# Supplementary material for: Gaussian Basis Sets for All-Electron Excited-State Calculations of Large Molecules
Source: J Chem Theory Comput. 2025 Dec 22;22(1):540–57. doi: 10.1021/acs.jctc.5c01386 (PMC12805578; doi:10.1021/acs.jctc.5c01386)
Supplement: Supplementary file 1 [file ct5c01386_si_001.pdf]

Supporting Information to "Gaussian basis sets for all-electron excited-state calculations of large molecules"

Rémi Pasquier, Maximilian Graml, and Jan Wilhelm
Institute of Theoretical Physics and Regensburg Center for Ultrafast Nanoscopy (RUN), University of Regensburg, 93053 Regensburg, Germany
(Dated: December 2, 2025)

In this supporting information, we provide additional benchmark calculations to assess the numerical precision of our developed all-electron augmented MOLOPT basis sets. We define another benchmark set containing 123 small molecules in Sec. S1 for benchmarking the augmented MOLOPT basis sets of Li, Be, B, Na, Ca, Al, Si, P, which are only rare in the *GW*5000 subset used in the main text. We report PBE0 and *GW* HOMO-LUMO gap and Bethe-Salpeter and TDDFT excitation energies in Sec. S2 computed with the augmented MOLOPT basis sets and compared to the complete basis set limit. We show additional results on the *GW*5000 subset with molecules with a LUMO energy below −2 eV (Sec. S3). We also list excitation gaps obtained with BSE and TDDFT (Sec. S4). We provide all the newly generated orbital (Sec. S5) and auxiliary RI (Sec. S6) basis sets in the CP2K basis set file format.

S1. MOLECULAR TEST SET CONTAINING Li, Be, B, Na, Ca, Al, Si, P ELEMENTS

The molecular set containing Li, Be, B, Na, Ca, Al, Si, P was built from the GMTKN55 set [1], by combining the W4-11 [2], ALK8 [3] and ALKBDE10 [4] subsets and removing all open-shell molecules. It contains 123 molecules, mostly of size between 2 and 9 atoms (with 2 molecules of size bigger than 10 atoms), with the most common elements being H (59 %) and C (45 %), the less present elements are O (33 %), F (21 %), N (20 %), Li (11 %), Cl (9 %), S (8 %), B (7 %), Al (5 %), Be (4 %), Si (4 %), Na (3 %), P (2 %) and Mg (2 %). The molecules in this set are therefore much smaller and hence have on average a higher, more diffuse LUMO than the *GW*5000 set. As such, we expect the basis-set convergence to the complete basis set limit to be slower than with the *GW*5000.

S2. HOMO-LUMO GAPS AND EXCITATION ENERGIES OF MOLECULES WITH Li, Be, B, Na, Ca, Al, Si, P

In this section, we present the basis-set convergence for the small molecule set, see Fig. S1. As before, Fig. S1a compares PBE0 HOMO–LUMO gaps across the four basis set families. The aug-MOLOPT-ae basis sets show a systematic improvement to the complete-basis-set (CBS) limit, the largest aug-TZVP-MOLOPT-ae basis gives a mean absolute deviation (MAD) of 73 meV to the CBS. If we only consider molecules with LUMO < -2 eV with less diffuse LUMO, the MAD of aug-TZVP-MOLOPT-ae to the CBS reduces to 21 meV. The aug-cc-pVXZ basis sets show a faster convergence on the whole set than their aug-MOLOPT-ae counterpart, with aug-cc-pVTZ having a MAD of 21 meV, showing the importance of very diffuse functions to describe the LUMO especially for very small molecules. This can also be seen in the non-augmented cc-pVXZ and MOLOPT basis sets, which exhibit very slow convergence unless molecules with higher LUMO energies are removed.

In Fig. S1b, we show the basis set convergence of the four basis set families for G_0W_0@PBE0 HOMO–LUMO gaps. The aug-MOLOPT-ae basis sets exhibits good convergence to the CBS. The MAD of the aug-DZVP-MOLOPT-ae basis is 242 meV, slightly smaller than the corresponding aug-cc-pVDZ value of 268 meV. On the other hand, the aug-TZVP-MOLOPT-ae calculation shows a MAD of 167 meV, which is larger than the corresponding aug-cc-pVTZ values of 105 meV. As such, in the context of very small molecules, the aug-MOLOPT-ae basis sets are also a good choice for the calculation of *GW* HOMO-LUMO gaps in small molecules. Just like for *GW*5000, non-augmented cc-pVXZ and MOLOPT basis sets exhibit very slow comvergence to the CBS for G_0W_0@PBE0 HOMO–LUMO gaps. Here, for example, the MAD of the large cc-pV5Z basis is 627 meV, but this value decreases to 97 meV once molecules with large LUMO energies are removed from the set.

In Figure S1c, we show the basis set convergence of the four basis set families for the first ten BSE@G_0W_0@PBE0 excitation energies. The MAD of the aug-TZVP-MOLOPT-ae basis set is 210 meV, larger than the corresponding value for aug-cc-pVTZ at 104 meV, so that the aug-cc-pVXZ basis set family seems to outperform the aug-MOLOPT-ae basis sets for small molecules. The non-augmented cc-pVXZ and MOLOPT basis sets show huge errors; for example, the MAD of the large cc-pV5Z basis is 808 meV— almost four times the error of aug-TZVP-MOLOPT-ae, so that augmentation appears to be an absolute necessity to properly converge excitation energies of small molecules. Note that this value does not decrease significantly when removing molecules with large LUMO energies, with a MAD of 583 meV in that case.

Finally, Figure S1d shows the basis set convergence of the first ten excitation energies computed with TDDFT (PBE0). As before, the aug-MOLOPT-ae basis sets exhibit systematic improvement with increasing basis size. The aug-cc-pVXZ basis sets again outperform the aug-MOLOPT-ae family: the MAD of aug-TZVP-MOLOPT-ae is 221 meV, whereas aug-cc-pVTZ has a

MAD of 137 meV. Once again, the non-augmented basis set families show extremely slow convergence to the CBS, with the cc-pV5Z basis set showing a MAD of 784 meV. Once again, this value is decreased to 583 meV after removing molecules with higher LUMO energies.

S3. HOMO-LUMO GAPS AND EXCITATION ENERGIES OF *GW*5000 SUBSET WITH LUMO < – 2 eV

In this section, we report the HOMO-LUMO gaps and excitation energies for the molecules in the *GW*5000 subset with a LUMO energy below – 2 eV in Fig. S2. We observe that removing the molecules with LUMO energy above – 2 eV leads to improvements of the MAD to the CBS. These improvements, however, a strongly dependent on the computation method and the basis set familiy. The improvement is generally stronger for non-augmented basis sets than augmented ones, with the MAD values being decreased by a factor up to ≈ 3 for example for *GW* gaps using aug-cc-pVDZ (from 218 meV to 71 meV), whereas we observe reductions up to one order of magnitude for non-augmented bases as we have already emphasized in the main article, with for example the cc-pV5Z MAD shrinking from 78 meV to 7 meV. This is a clear indicator of the fact that non-augmented basis set families lack more diffuse basis set functions and as a result, very slowly converge to the CBS limit for molecules with larger LUMO energies.

S4. *GW*+BSE & TDDFT EXCITATION ENERGIES GAPS OF *GW*5000 SUBSET

In this section, we report differences (i.e., gaps) of excited-state energies computed from *GW*+BSE and TDDFT in Fig. S3 for the *GW*5000 subset defined in the main text. Excited-state energy differences are important, because these energy differences are required to carry simulations of excited-state dynamics since essential quantities such as the rate for cascade transition processes are strongly dependent on the level spacings between excitation energies.

In Figure S3a, we show the basis set convergence of the four basis set families for the first nine BSE@G_0W_0@PBE0 excitation gaps. The MAD of the aug-SZV-MOLOPT-ae basis set is 83 meV, better than the larger aug-cc-pVDZ basis set at 91 meV. Already at the level of the very small aug-SZV-MOLOPT-mini-ae basis set, the MAD is somewhat close to these values at 117 meV. The MAD of the aug-TZVP-MOLOPT-ae basis set is 39 meV, slightly smaller than the corresponding value for aug-cc-pVTZ at 43 meV, so that the aug-cc-pVXZ basis set family seems to perform slightly worse than the aug-MOLOPT-ae basis. The non-augmented cc-pVXZ and MOLOPT basis sets show very slow convergence: the MAD of the cc-pV5Z basis set is 91 meV, worse than the aug-SZV-MOLOPT-ae basis set result, although the difference with the augmented basis set isn't as drastic as for the previous cases. Interestingly, we note that in this case removing molecule with larger LUMO values seems to have a strong impact on both augmented and non-augmented basis set families (although the effect is still stronger for non-augmented basis sets).

Figure S3b shows the basis set convergence of the first nine excitation gaps computed with TDDFT (PBE0). In this case, the aug-cc-pVXZ basis sets show slightly better convergence than the aug-MOLOPT-ae basis set: the aug-TZVP-MOLOPT-ae basis set shows a MAD of 15 meV, slightly worse than the equivalent aug-cc-pVTZ value of 11 meV. As such, unlike BSE, the aug-cc-pVXZ family converges faster to the CBS than the aug-MOLOPT-ae family. Once again, the non-augmented basis set families show slow convergence to the CBS as compared to the augmented basis set families, with the cc-pV5Z basis set showing a MAD of 67 meV. Similarly to BSE calculations, removing molecules with higher LUMO values from the set greatly reduces the MAD values across all the basis sets.

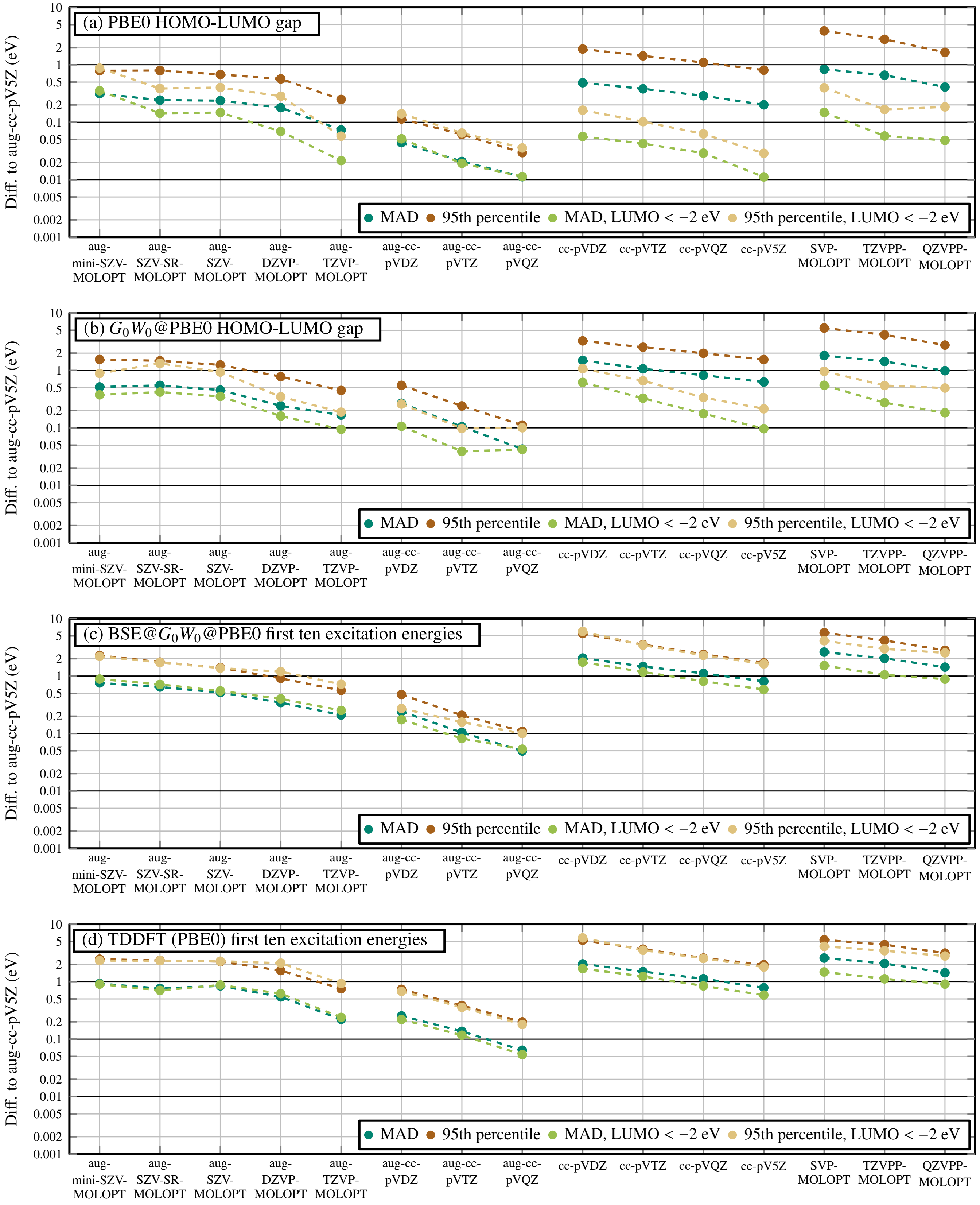

FIG. S1. Basis set convergence of excited-state energies for a subset of 123 molecules from the GMTKN55 set. We report the mean absolute deviation (MAD) and 95th percentile error (95PE) relative to the aug-cc-pV5Z basis for the aug-MOLOPT-ae basis sets developed in this work, aug-cc-pVXZ [5], cc-pVXZ [6] and all-electron MOLOPT basis sets [7, 8]. Panels show (a) PBE0 HOMO–LUMO gaps, (b) G_0W_0@PBE0 HOMO–LUMO gaps, (c) first ten excitation energies computed from BSE@G_0W_0@PBE0, and (d) from TDDFT (PBE0).

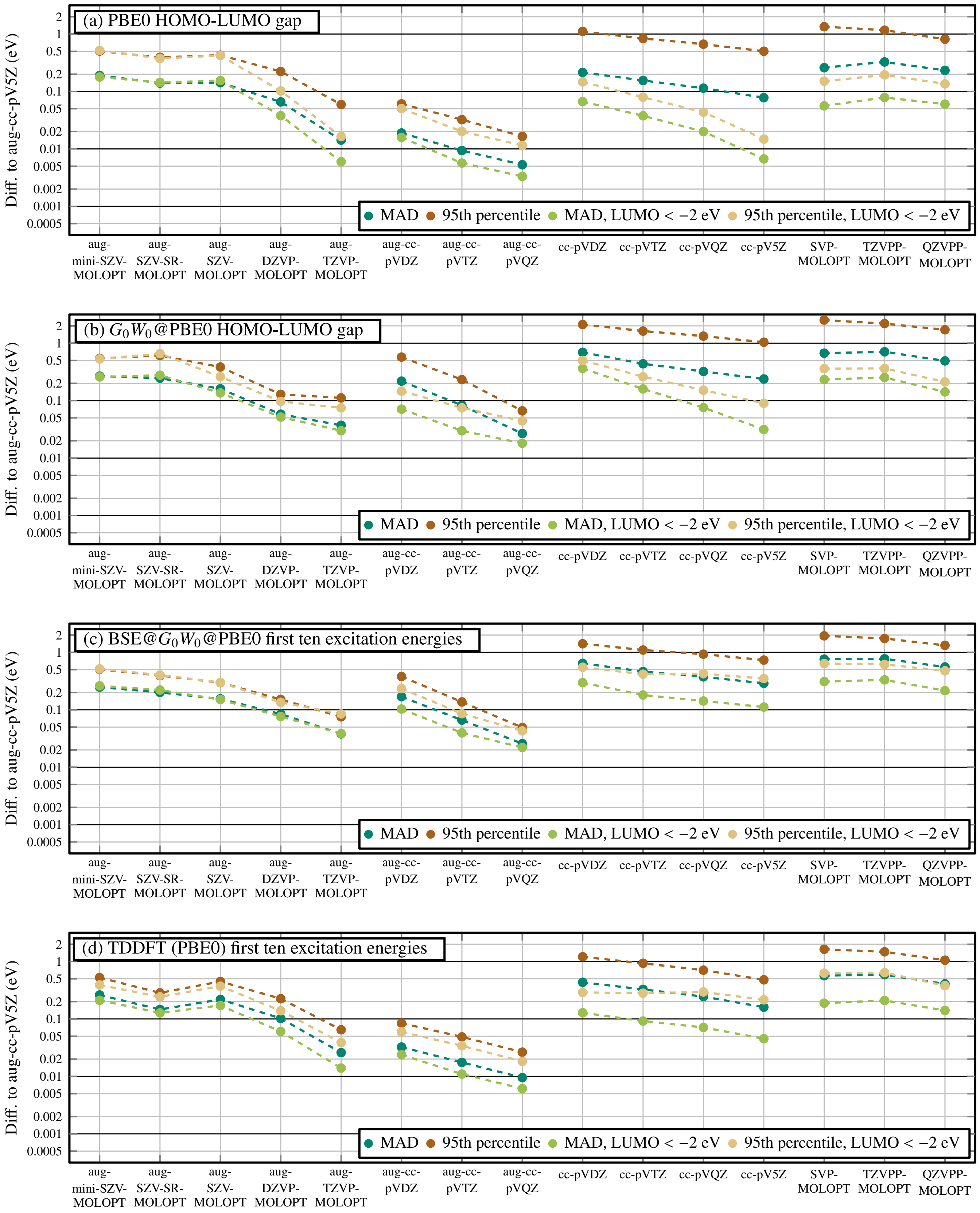

FIG. S2. Basis set convergence of excited-state energies for a subset of 247 molecules from the *GW*5000 benchmark set, including results for molecules with a LUMO<-2 eV

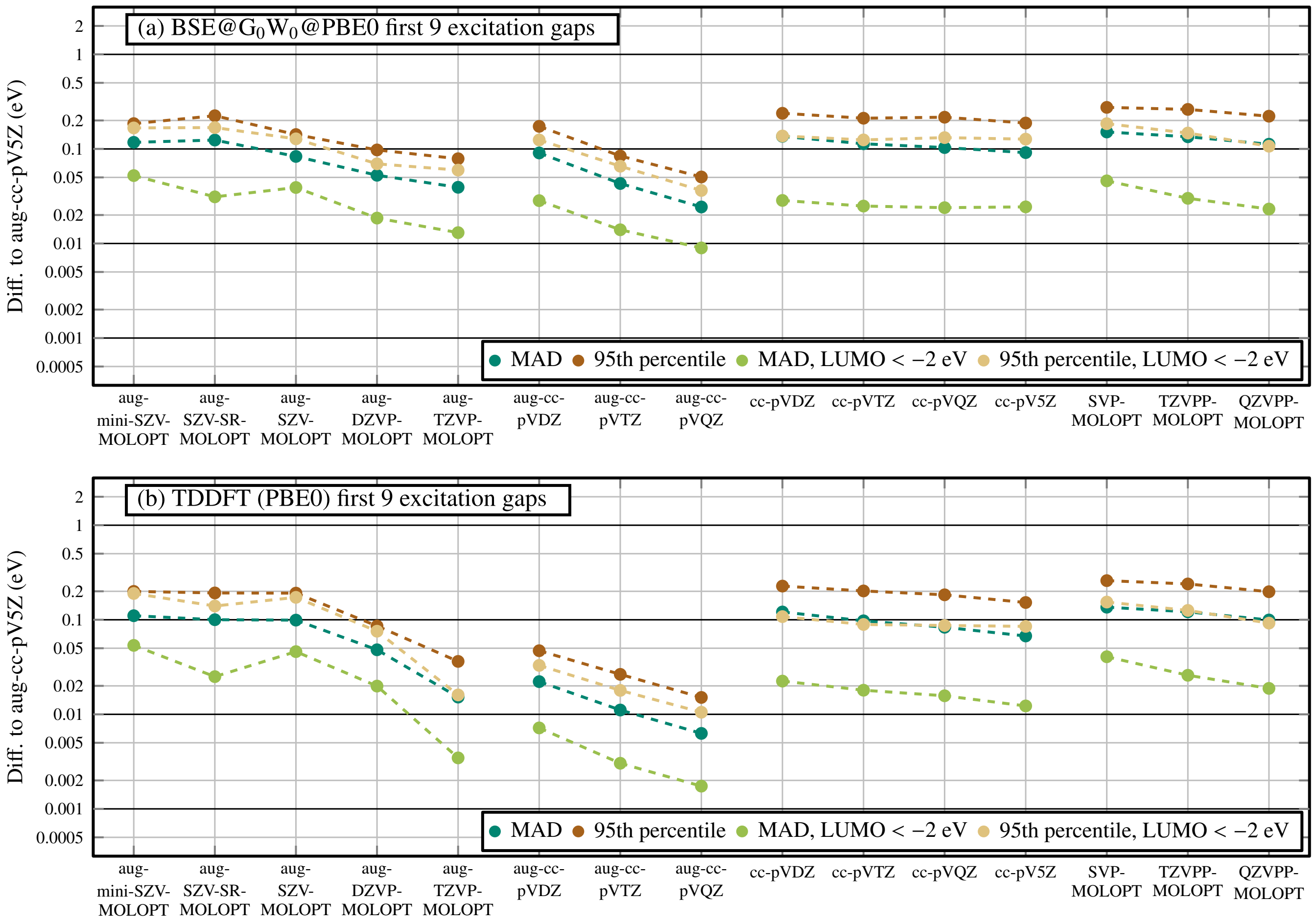

FIG. S3. Basis set convergence of the first 9 excited-state energy gaps for BSE@G_0W_0@PBE0 (a) and TDDFT (PBE0) (b) for a subset of 247 molecules from the *GW*5000 benchmark set, including results for molecules with a LUMO<-2 eV

S5. COMPLETE LIST OF GENERATED BASIS SETS

```
H  aug-SZV-MOLOPT-ae-SR
                1
2 0 1 4 3 1
 0.065428  0.000000 -0.612124  3.026281  0.585988
 0.168855  0.444635  1.176287 -2.032217 -0.102977
 0.623914  0.535328  0.137395 -0.019539  0.041334
 3.425251  0.154329 -0.010529  0.005118  0.001491

H  aug-SZV-MOLOPT-ae
       1
1 0 1 7 3 1
 0.065428  0.000000  4.851857  1.746076  0.476202
 0.100112  0.130334  0.405435 -5.736391 -0.111030
 0.243077  0.416492  0.054771 -0.329666  0.000050
 0.625955  0.370563  0.143656  0.109888  0.008399
 1.822143  0.168538  0.000000  0.000000  0.000000
 6.513144  0.049361  0.000000  0.000000  0.000000
35.523221  0.009164  0.000000  0.000000  0.000000

H aug-DZVP-MOLOPT-ae
          1
 2 0 1 5 3 2
 0.035000  0.000000  0.000000  1.585565  0.000000  3.171889
 0.111920  0.091223  0.981290 -1.494757  0.099890  3.190534
 0.374948  0.827248  0.020102 -0.001112  0.856402  0.411491
 1.446686  0.546801 -0.189239 -0.000055  0.505675  0.095052
12.248855  0.091360 -0.029223  0.000000 -0.029854  0.000000

H aug-TZVP-MOLOPT-ae
       1
 1 0 2 6 4 3 2
 0.030000  0.000000  0.000000  0.000000  2.025871  0.000000  0.000000  0.097083  0.000000  6.563029
 0.091371 -0.305476 -0.354607  0.868704 -0.527389  0.131942 -0.166958  0.038224 -0.201435  1.798424
 0.293823 -0.133308  0.819166  0.366069 -2.071514 -0.026498  0.979894 -0.010515  0.287655  3.448935
 1.065127  0.814717  0.211440  0.296545 -0.246941  0.976473  0.063472  0.001932  0.935175  1.020424
```

```
 4.947701 -0.083813  0.228656 -0.022813  0.000000  0.135570 -0.047852  0.000000 -0.044240  0.000000
34.177738 -0.467039  0.325931 -0.151283  0.000000 -0.100054  0.074932  0.000000  0.012969  0.000000

He aug-SZV-MOLOPT-ae
      1
1 0 1 7 2 1
 0.090000  0.000000  4.429372  2.183671
 0.185959  0.130334 -0.229042 -0.856279
 0.451516  0.416492  0.093049 -0.015621
 1.162715  0.370563  0.173642  0.045970
 3.384640  0.168538  0.000000  0.000000
12.098198  0.049361  0.000000  0.000000
65.984568  0.009164  0.000000  0.000000

He aug-DZVP-MOLOPT-ae
      1
1 0 2 6 3 2 1
 0.060000  0.000000  0.000000  0.745142  0.000000 -0.654292 -0.054702
 0.110000  0.000000  0.000000  1.374477  0.000000  0.025698  0.329772
 0.217321  0.578202  0.815195  0.100000  0.156837  0.100000  0.100000
 0.873488  0.743305 -0.528086  0.100000  0.983426  0.100000  0.100000
 4.185521  0.333322 -0.226554  0.000000  0.021403  0.000000  0.000000
38.837462  0.045572 -0.072494  0.000000  0.088422  0.000000  0.000000

 He aug-TZVPP-MOLOPT-PBE-ae
      2
1 0 0 3 1
98.119800  0.084471
14.434417  0.764705
 3.868555  0.638820
1 0 3 5 3 3 2 1
 0.050000  0.000000  0.000000 -0.307658  0.000000  0.000000  0.058910  0.000000 -0.631727  0.025691
 0.100000  0.000000  0.000000  0.100000  0.000000  0.000000  0.100000  0.000000  0.100000  0.100000
 0.197567  0.620058  0.741159  0.100000 -0.031037 -0.003721  0.100000 -0.262397  0.100000  0.100000
 0.700286  0.692952 -0.671161  0.100000 -0.218168  0.975906  0.100000  0.480410  0.100000  0.100000
 2.525067  0.367893  0.015005  0.000000  0.975417  0.218159  0.000000  0.836872  0.000000  0.000000

Li aug-SZV-MOLOPT-ae
   2
1 0 0 6 1
167.175846  0.009164
30.651508  0.049361
 8.575187  0.168538
 2.945808  0.370563
 1.143944  0.416492
 0.471139  0.130334
1 0 2 6 2 2 1
 0.031084  0.240706 841.480841  0.101708 -0.261789  0.212384
 0.067814  0.595117 -1.488165  0.425860  5.336068  0.734068
 0.156146  0.250242 -1.212976  0.418036 -0.029867  0.954102
 0.405851 -0.033785  0.100000  0.173897  0.100000  0.100000
 1.305830 -0.046992  0.100000  0.037679  0.100000  0.100000
 6.597564 -0.013253  0.000000  0.003760  0.000000  0.000000

Li aug-DZVP-MOLOPT-ae
   2
1 0 0 7 3
266.127173  0.011547  0.019322 -0.010295
30.791085  0.111439 -0.177203  0.056533
 6.513909  0.405297  0.335514 -0.224340
 1.822796  0.728172 -0.295961 -0.103657
 0.563425  0.447754 -0.004949 -0.244566
 0.074745  0.273052  0.655786  0.665251
 0.024840  0.133817 -0.581364  0.658220
1 0 2 5 1 3 1
 0.029000 33.124066  0.000000  0.000000 -0.706586  0.139558
 0.048794 -25.961564  0.699566  0.711005 -0.347504  7.572431
 0.100000 -4.365085  0.000000  0.000000  0.007044  2.884261
 0.223369  1.167885  0.698543 -0.659464  0.170593  3.853951
 1.213190  0.195071  0.150484 -0.244088  0.084547  0.252432

Li aug-TZVP-MOLOPT-ae
   2
1 0 0 6 1
6269.059024  0.000494
940.419254  0.003727
214.089215  0.019515
60.725519  0.081988
18.086742  0.330849
 5.657040  0.939905
1 0 3 5 5 4 2 1
 0.024646 -0.540924  0.215672  0.662872  0.076161 26.182012  0.028026 -0.799598  0.023664 -1.543642 -0.076993 -0.248145 -1.439282
 0.071007  0.667156  0.351697  0.426174  0.493845 -2.753986 -0.008045 -0.456513  0.622436  1.935165  0.492954 375.952136  2.048668
 0.237636 -0.294238  0.139882  0.340505 -0.129413 -6.415364  0.156340  0.369598  0.758288  0.441454  0.851731  0.100000 -0.141917
 0.680928  0.313096  0.449139  0.111412 -0.818886  0.474386 -0.655725  0.114949  0.191751  0.100548  0.148000 -1.018677 -0.062383
 1.933558 -0.278745  0.780066 -0.500620  0.250987  0.114267  0.738060  0.049222  0.015620  0.091762 -0.060987 -1.066114  0.022877

Be aug-SVP-MOLOPT-ae
   2
1 0 0 6 1
312.870494  0.009164
57.364463  0.049361
16.048509  0.168538
 5.513096  0.370563
 2.140897  0.416492
 0.881739  0.130334
1 0 2 6 2 2 1
 0.064233  0.240706 47.595456  0.101708 28.522682 -1.053541
 0.140131  0.595117 -13.675393  0.425860  3.461984  1.581876
 0.322660  0.250242 -3.324948  0.418036 -0.406003  1.756679
 0.838653 -0.033785  0.289101  0.173897 -0.132274 -0.398239
 2.698375 -0.046992  0.000000  0.037679  0.000000  0.000000
13.633247 -0.013253  0.000000  0.003760  0.000000  0.000000

Be aug-DZVP-MOLOPT-ae
   2
1 0 0 6 1
4700.379494  0.001432
704.895908  0.010001
160.564268  0.054123
45.293874  0.208339
```

```
14.656253  0.614295
 6.001848  0.759082
1 0 3 6 5 4 2 1
 0.035000  0.000000  0.000000  0.000000  0.000000  4.336292  0.000000  0.000000  0.000000 44.754099  0.000000 -0.038306 -14.891476
 0.058229  0.075536  0.332027  0.078739  0.934044 -3.365033 -0.031763  0.591411  0.477181 -26.314611  0.073155 117.690982  0.477966
 0.180196  0.264992  0.480992  0.790875 -0.251280 -0.524755 -0.560690  0.046061  0.631436 -0.581450  0.438934  0.514709  1.065123
 0.504171 -0.230699  0.803764 -0.468357 -0.239756  1.259558 -0.820684  0.000788 -0.472249  1.718034  0.885000 -0.622600  0.101894
 1.327711  0.598641  0.103526 -0.352462  0.000575  0.068231 -0.080602  0.109567 -0.089719  0.046891 -0.130107 -0.380829  0.100020
 3.215821  0.715878 -0.040631 -0.157253 -0.083285  0.000000 -0.067818 -0.797562  0.377515  0.000000  0.042812  0.000000  0.000000

Be aug-TZVP-MOLOPT-ae
   2
1 0 0 8 1
29646.682404  0.001139
4428.689934  0.001377
1005.571385  0.036217
284.153173  0.068796
92.635632  0.481731
33.244368  0.150189
13.025638  0.582151
 5.404143  0.632796
1 0 4 7 7 5 3 2 1
 0.042000 -0.252407  0.407129 -0.224200  0.429076 -0.117017  0.460181 -0.151735  0.156715 -0.002487  0.071115  0.970931  1.695754 -0.214407  0.353761  0.034888 -0.104499 26.179418 -0.024147
 0.065000  0.000000  0.000000  0.000000  0.000000  0.000000  0.000000  0.353646  0.000000  0.000000  0.000000  0.000000 -1.518034  0.000000  0.000000 18.974283  0.000000 -5.680467 12.455704
 0.114363 -0.064917  0.201814  0.350553  0.289018  0.816858  0.171238 -0.272089  0.107190  0.105176  0.928079 -0.050597 -0.206206  0.051097  0.522668  0.100000  0.204195  0.100000  0.100000
 0.289873  0.011904  0.129975  0.212709  0.484015 -0.052767 -0.793251  0.100000  0.888736  0.001676 -0.023025 -0.170695  0.100000  0.949766  0.208431  0.100000  0.970556  0.100000  0.100000
 0.898109  0.011359 -0.280974  0.835628  0.026986 -0.295976  0.289901  0.100000  0.372282 -0.150287 -0.067280 -0.004415  0.100000 -0.166831  0.715105  0.100000 -0.059353  0.100000  0.100000
 2.358409 -0.118014  0.803207  0.283003 -0.337616 -0.268576 -0.071316  0.000000  0.023885 -0.141271  0.050258 -0.147654  0.000000 -0.068945  0.037176  0.000000  0.040508  0.000000  0.000000
 6.728135  0.938856  0.205476 -0.023778  0.205198 -0.083122  0.158406  0.000000  0.039918  0.971797 -0.087505 -0.016612  0.000000  0.119899 -0.175018  0.000000 -0.015502  0.000000  0.000000
23.115291  0.190861  0.101564  0.051690 -0.584186  0.386810 -0.124269  0.000000  0.182597  0.044675 -0.344052  0.059150  0.000000  0.049003 -0.121818  0.000000 -0.001016  0.000000  0.000000

B aug-SVP-MOLOPT-ae
   2
1 0 0 6 1
506.011837  0.009164
92.776716  0.049361
25.955582  0.168538
 8.916443  0.370563
 3.462516  0.416492
 1.426055  0.130334
1 0 2 7 2 2 1
 0.109280  0.240706 -0.259247  0.101708  7.061007  0.313287
 0.155613  0.000000 -0.485319  0.000000  2.601650  0.346061
 0.238409  0.595117 -0.074370  0.425860  0.654894  8.878371
 0.548949  0.250242  0.089153  0.418036 -0.046084  1.792249
 1.426820 -0.033785  0.101784  0.173897  0.100000  0.100000
 4.590809 -0.046992  0.000000  0.037679  0.000000  0.000000
23.194561 -0.013253  0.000000  0.003760  0.000000  0.000000

B aug-DZVP-MOLOPT-ae
   2
1 0 0 7 3
839.324562 -0.008085  0.000542 -0.011314
126.174643 -0.064117 -0.005126  0.002304
28.339532 -0.291110 -0.022897 -0.069906
 7.329111 -0.726250 -0.108395 -0.086717
 2.208031 -0.568885 -0.226861 -0.027617
 0.410209 -0.115808  0.775219 -0.608610
 0.151694 -0.215867  0.579025  0.785042
2 0 3 5 1 3 2 1
 0.082134 100.786823 -0.107696  0.820575  0.112100  0.024466  8.611727 -0.096793
 0.140000 39.265376  0.000000  0.000000  0.178722  0.000000 -5.082317  0.765548
 0.279625 -14.467020 -0.724173  0.320342 -0.052384  0.791419 -0.242747  0.445998
 0.881274  4.381849 -0.663755 -0.470276  0.096147  0.610604 -0.861764  0.068238
 4.510873  0.000000 -0.152986 -0.053652  0.000000 -0.014877  0.000000  0.000000

B aug-TZVP-MOLOPT-ae
   2
1 0 0 6 1
8564.805310  0.001007
1284.266148  0.009214
292.317852  0.042162
82.773089  0.190050
26.955680  0.341703
 9.937437  0.919378
1 0 4 8 5 4 3 2 1
 0.037000  0.000000  0.000000  0.000000  0.000000 55.333899  0.000000  0.000000  0.000000  0.109631  0.000000  0.000000  6.680313  0.000000 32.176408  3.280828
 0.055000  0.000000  0.000000  0.000000  0.000000 -48.469154  0.000000  0.000000  0.000000 -3.440413  0.000000  0.000000 32.644826  0.000000 16.302139  1.291731
 0.081527  0.055726  0.265332  0.034872  0.857189  0.916508  0.039189 -0.043335  0.963883 -1.936783  0.135178 -0.117988  3.093239 -0.225109  1.558452 13.954102
 0.231037  0.026180  0.401371  0.880466 -0.035360 -0.910465  0.050573  0.977439  0.049821 -0.466707  0.115113  0.784635 -1.819112  0.415784 -0.900000  0.253907
 0.627002 -0.091939  0.857064 -0.356947 -0.323612  1.100000  0.842584  0.051169  0.010320  0.296972  0.685289  0.375882  0.100000  0.868307  0.100000  0.100000
 1.676500  0.507137  0.159803 -0.251296  0.227852  0.100000  0.475178 -0.104640 -0.160187  0.100000  0.650277 -0.398358  0.100000  0.148891  0.100000  0.100000
 4.312240  0.840577 -0.047240  0.109545 -0.264275  0.000000  0.225251 -0.166840  0.170636  0.000000 -0.099016  0.074426  0.000000 -0.008796  0.000000  0.000000
22.230570  0.154915  0.078565 -0.144925  0.193637  0.000000  0.097083 -0.036504 -0.116468  0.000000 -0.257275  0.254778  0.000000  0.015723  0.000000  0.000000

C aug-SZV-MOLOPT-ae-SR
             2
1 0 0 3 1
71.616837  0.154329
13.045096  0.535328
 3.530512  0.444635
2 0 2 4 2 2 1
 0.114559  0.000000  1.641269  0.000000  0.811821  0.026539
 0.222290  0.700115  1.667360  0.391957 -0.862449  0.200443
 0.683483  0.399513 -0.467157  0.607684  0.356215  0.033815
 2.941249 -0.099967  0.076264  0.155916 -0.021797  0.008057

C aug-SZV-MOLOPT-ae
       2
1 0 0 6 1
742.737049  0.009164
136.180025  0.049361
38.098264  0.168538
13.087782  0.370563
 5.082369  0.416492
 2.093200  0.130334
2 0 2 7 2 2 1
 0.070000  0.000000 -31.050837  0.000000  0.293729  0.171948
 0.143687  0.240706 36.758105  0.101708 -0.134549  0.143038
 0.313471  0.595117  0.734526  0.425860  0.006029  0.361380
 0.721783  0.250242  6.675481  0.418036  0.026990  0.053989
```

```
 1.876046 -0.033785  0.000000  0.173897  0.000000  0.000000
 6.036200 -0.046992  0.000000  0.037679  0.000000  0.000000
30.497240 -0.013253  0.000000  0.003760  0.000000  0.000000

C aug-DZVP-MOLOPT-ae
         2
 2 1 3 5 3 2 1
 0.050000  0.000000  0.000000  0.151423  0.000000 -0.059937 -0.002447
 0.096935  0.097878  0.899922 -0.188697  0.058721  0.092684  0.025053
 0.300899  0.640599  0.269763 -0.009629  0.678270 -0.057369  0.000961
 0.937529  0.723207 -0.326894  0.007878  0.730958  0.000000  0.000000
 4.145546  0.238798 -0.102515  0.000000  0.046916  0.000000  0.000000
 1 0 0 8 4
 0.060000  0.000000  0.000000  0.000000 -0.733195
 0.161278  0.194025  0.569392  0.796229 -0.123048
 0.523772 -0.096783  0.818843 -0.558118  1.297796
 3.176528  0.570205 -0.060279 -0.116302  0.045130
10.501907  0.736614 -0.010355 -0.150059  0.000000
40.805234  0.282730  0.039122 -0.120910  0.000000
186.270922  0.072758 -0.003870  0.031268  0.000000
1238.394264 -0.004784  0.001271 -0.053695  0.000000

C aug-TZVP-MOLOPT-ae
       2
 1 0 0 6 1
13575.369584  0.000458
2035.237517  0.012134
463.111112  0.039477
131.318553  0.227675
42.899593  0.183999
15.855102  0.955302
 1 0 4 7 5 4 3 2 1
 0.040000  0.000000  0.000000  0.000000  0.000000  0.088606  0.000000  0.000000  0.000000  0.086814  0.000000  0.000000 16.186294  0.000000  0.095389  0.033773
 0.097098  0.223019  0.314774  0.365074  0.759044 -0.076753  0.086904  0.099383  0.969387 -0.024439  0.069953  0.048444 -1.773328 -0.126955  0.212390  0.349000
 0.283960  0.142304  0.459233  0.588311 -0.615167 -0.025870  0.143786  0.946250 -0.053915 -0.023431  0.117179  0.844846  2.930809  0.598728 -1.188411  0.052239
 0.806764  0.008727  0.801078 -0.400910  0.075110  0.002813  0.943473 -0.164395 -0.059384 -0.001283  0.664978  0.314837  0.046531  0.781790  0.001042 -0.002231
 2.441624  0.459594 -0.100655 -0.137654 -0.019356  0.000000  0.211052  0.199160 -0.181773  0.000000  0.658920 -0.351582  0.000000  0.116047  0.000000  0.000000
 6.739673  0.842949 -0.129849 -0.054453 -0.067193  0.000000  0.191278 -0.166915  0.105142  0.000000  0.125840 -0.104282  0.000000 -0.023169  0.000000  0.000000
34.417212  0.090261  0.145960 -0.581350 -0.186792  0.000000  0.022400  0.013511 -0.098785  0.000000  0.298612 -0.224237  0.000000  0.014412  0.000000  0.000000

N aug-SZV-MOLOPT-ae-SR
       2
1 0 0 3 1
99.106169  0.154329
18.052312  0.535328
 4.885660  0.444635
1 0 2 4 2 2 1
 0.112012  0.000000  0.038235  0.000000  0.039958 -0.001006
 0.285714  0.700115 -0.042894  0.391957 -0.025036  0.099998
 0.878497  0.399513 -0.045881  0.607684  0.001604 -0.003370
 3.780456 -0.099967 -0.000068  0.155916  0.000028  0.010631

N aug-SZV-MOLOPT-ae
       2
1 0 0 6 1
1027.828458  0.009164
188.451223  0.049361
52.721861  0.168538
18.111382  0.370563
 7.033180  0.416492
 2.896652  0.130334
1 0 2 7 2 2 1
 0.090000  0.000000 -155.729656  0.000000 -0.084093 -0.096605
 0.184684  0.240706 61.812669  0.101708 -0.085905 -0.131499
 0.402911  0.595117 -69.869801  0.425860  0.044939  0.068145
 0.927724  0.250242 -5.554590  0.418036  0.150796 -0.119146
 2.411326 -0.033785  0.000000  0.173897  0.000000  0.000000
 7.758467 -0.046992  0.000000  0.037679  0.000000  0.000000
39.198808 -0.013253  0.000000  0.003760  0.000000  0.000000

N aug-DZVP-MOLOPT-ae
       2
 1 0 0 7 3
 0.191012 -0.018407  0.497263  0.867362
 0.666629  0.036018  0.864889 -0.494509
 4.403487 -0.581552  0.005464 -0.020046
14.651380 -0.756096  0.000103 -0.010627
57.692026 -0.291057  0.066982 -0.050367
257.997554 -0.060703 -0.012912  0.009619
1713.054433 -0.009023  0.003547 -0.000848
 2 0 3 5 1 3 2 1
 0.060000 -0.112088  0.000000  0.000000 25.236101  0.000000 -0.075158  0.241728
 0.166384  0.020261 -0.287697  0.957131 15.592722  0.333387 -0.043511  4.579733
 0.634073 -0.011016 -0.834182 -0.233858 -2.139130  0.918261 -0.008583  5.859281
 2.325354  0.000000 -0.460187 -0.168910  0.000000  0.213621  0.000000  1.618034
12.268174  0.000000 -0.097971 -0.026066  0.000000 -0.003947  0.000000  0.000000

N aug-TZVP-MOLOPT-ae
    2
 1 0 0 6 1
19730.758242  0.000723
2958.006020  0.008109
673.387518  0.032294
190.675174  0.162882
62.366448  0.292109
22.833858  0.941824
 1 0 4 7 5 4 3 2 1
 0.045000  0.000000  0.000000  0.000000  0.000000  8.372960  0.000000  0.000000  0.000000  0.230132  0.000000  0.000000 -0.760836  0.000000  0.439735  0.526255
 0.113512  0.135763  0.339285  0.274575  0.779513 -2.777238  0.094756  0.115124  0.988042 -3.275799  0.267525 -0.196165  0.355861  0.037198  0.951866  1.717754
 0.322032  0.154514  0.393935  0.801001 -0.389361 -0.831463  0.164439  0.966294 -0.122235  0.415750  0.021183  0.857338  4.021778  0.269003 14.441572  0.399187
 0.930550 -0.119116  0.849739 -0.481864 -0.177596  1.000000  0.870197 -0.114639 -0.083062  0.618034  0.779832  0.302948  1.939327  0.957010  6.463948  0.145898
 2.987378  0.431666  0.049052 -0.113212  0.249961  0.000000  0.437091 -0.132674 -0.007822  0.000000  0.504713 -0.280541  0.000000  0.086664  0.000000  0.000000
 8.608625  0.866560 -0.024829 -0.193670 -0.170341  0.000000  0.085696  0.017344 -0.017006  0.000000 -0.252571  0.232698  0.000000 -0.044563  0.000000  0.000000
49.270190  0.079015 -0.067983 -0.022077 -0.343104  0.000000  0.091354 -0.148245  0.039776  0.000000 -0.036273  0.043146  0.000000  0.029812  0.000000  0.000000

O aug-SZV-MOLOPT-ae-SR
       2
1 0 0 3 1
130.709321  0.154329
 23.808866  0.535328
  6.443608  0.444635
```

```
1 0 2 4 2 2 1
 0.162492  0.000000  3.462113  0.000000  0.144311 -0.049716
 0.380389  0.700115  1.990000  0.391957 16.479501  0.112570
 1.169596  0.399513 -0.000269  0.607684  0.007424  0.008654
 5.033151 -0.099967 -0.195350  0.155916  0.006001  0.003894

O aug-SZV-MOLOPT-ae
       2
1 0 0 6 1
1355.584234  0.009164
248.544886  0.049361
69.533902  0.168538
23.886772  0.370563
 9.275933  0.416492
 3.820341  0.130334
1 0 1 7 2 2 1
 0.123000  0.000000 -68.535898  0.000000 13.668314 -0.771118
 0.245881  0.240706 30.092081  0.101708 30.455761  1.222295
 0.536420  0.595117  5.697724  0.425860 -24.097881  0.846995
 1.235135  0.250242  3.592084  0.418036 -2.419873  1.231367
 3.210345 -0.033785  0.000000  0.173897  0.000000  0.000000
10.329320 -0.046992  0.000000  0.037679  0.000000  0.000000
52.187762 -0.013253  0.000000  0.003760  0.000000  0.000000

O aug-DZVP-MOLOPT-ae
       2
 1 0 0 7 3
 0.293338 -0.007817  0.606131  0.794669
 0.941883  0.158317  0.783140 -0.591846
 5.946538 -0.584021  0.067645 -0.065515
19.692604 -0.746654  0.072751 -0.047785
76.928143 -0.268399  0.096636 -0.103150
340.903325 -0.064969 -0.008386  0.027897
2266.118827 -0.006532  0.004315 -0.015026
 2 0 3 5 1 3 2 1
 0.090000  3.896033  0.000000  0.000000  1.260041  0.000000 -0.272693  1.920557
 0.191936 -0.356438  0.173620  0.984764  2.946650  0.408031  0.134912  2.031777
 0.750068  0.391428  0.835859 -0.143392  0.424446  0.894420  0.611891  1.106812
 2.907977  0.000000  0.510733 -0.094994  0.000000  0.182905  0.000518 -0.454438
16.368686  0.000000  0.101724 -0.025586  0.000000  0.008337  0.000000  0.000000

O aug-TZVP-MOLOPT-ae
    2
 1 0 0 6 1
27032.341107  0.000735
4052.439160  0.008668
922.349653  0.033461
261.277359  0.168915
85.309424  0.328334
31.080802  0.928692
 1 0 4 7 5 4 3 2 1
 0.050000  0.000000  0.000000  0.000000  0.000000 16.433776  0.000000  0.000000  0.000000 -0.045112  0.000000  0.000000 -0.157989  0.000000 -0.101416  0.106738
 0.148931  0.071989  0.261293  0.414519  0.867396 -4.719347  0.020761  0.069906  0.996563  0.328013  0.257701 -0.110362  0.127312  0.089946  0.054371  0.481966
 0.454433  0.093292  0.231107  0.841721 -0.478471  0.385786 -0.036115  0.980776 -0.061268  0.974668  0.114853  0.835162  7.775561  0.294962  5.236068  3.456499
 1.335408  0.009216  0.933077 -0.327551 -0.121459 -1.472136  0.900080  0.045047 -0.019750  0.502427  0.553666  0.394485  0.618034  0.940648 -0.290908  0.001917
 4.395399  0.465742 -0.064034 -0.030034 -0.003981  0.000000  0.391422  0.052638 -0.030952  0.000000  0.770731 -0.353323  0.000000 -0.134770  0.000000  0.000000
12.309450  0.869241 -0.014246 -0.106600 -0.024760  0.000000  0.145932 -0.060524  0.017364  0.000000 -0.035845  0.047669  0.000000  0.043884  0.000000  0.000000
62.914956  0.116333 -0.058137  0.011194  0.057511  0.000000 -0.116673  0.157254 -0.038193  0.000000  0.136167 -0.087158  0.000000  0.000321  0.000000  0.000000

F aug-SVP-MOLOPT-ae
   2
1 0 0 6 1
1728.626574  0.009164
316.941790  0.049361
88.668891  0.168538
30.460157  0.370563
11.828570  0.416492
 4.871659  0.130334
1 0 2 7 2 2 1
 0.155133  0.000000 -187.198809  0.000000 77.663239  0.014359
 0.315820  0.240706 -9.931690  0.101708  6.003516  0.024879
 0.689002  0.595117 -20.203807  0.425860 -22.490088  0.145356
 1.586463  0.250242 88.505253  0.418036 -8.781211  0.630974
 4.123510 -0.033785  0.000000  0.173897  0.000000  0.000000
13.267438 -0.046992  0.000000  0.037679  0.000000  0.000000
67.032281 -0.013253  0.000000  0.003760  0.000000  0.000000

F aug-DZVP-MOLOPT-ae
   2
1 0 0 7 3
2894.857604 -0.018923 -0.016924  0.156429
435.720063 -0.054772  0.004359 -0.079843
98.526357 -0.273685  0.103734 -0.035091
25.521650 -0.737742  0.090985  0.011903
 7.740335 -0.597666  0.075355 -0.004083
 1.290275  0.118959  0.803005 -0.567629
 0.396917  0.078222  0.574595  0.803469
2 0 3 5 1 3 2 1
 0.110000 -0.467172  0.000000  0.000000  0.066650  0.000000  0.391373 -0.049550
 0.221407  0.229255 -0.161777  0.982507 -0.093893  0.402856 -1.015283  0.099388
 0.856285  0.106536 -0.826329 -0.181030  0.100609  0.903974 -0.048399  0.069658
 3.472802  0.000000 -0.528022 -0.008769  0.000000  0.142075  0.000000  0.000000
20.861065  0.000000 -0.110460 -0.042793  0.000000  0.018777  0.000000  0.000000

F aug-TZVP-MOLOPT-ae
   2
1 0 0 6 1
35479.028788  0.000673
5318.563130  0.008201
1210.532078  0.034158
342.851437  0.167981
112.046988  0.340180
40.784918  0.924568
1 0 4 8 5 4 3 2 1
 0.050000  0.000000  0.000000  0.000000  0.000000  3.721224  0.000000  0.000000  0.000000 -0.042045  0.000000  0.000000  0.214256  0.000000  0.006007  0.008025
 0.100000  0.000000  0.000000  0.000000  0.000000 -0.853358  0.000000  0.000000  0.000000  0.062065  0.000000  0.000000  0.200293  0.000000  0.073521 -0.020178
 0.194582  0.009396  0.125787  0.501269  0.855856  0.074692 -0.048568 -0.096683  0.987882  0.377408  0.155723 -0.110074  0.184821 -0.065872  0.088925 -0.005397
 0.613429 -0.178123  0.116155  0.834961 -0.505013 -0.002909  0.002358  0.952591  0.078611  2.100000  0.021487  0.958447  0.226215  0.567195  0.058136  0.043818
 1.800486  0.005422  0.984951 -0.165338 -0.048225 -0.005346  0.882036  0.022969  0.095499  0.300374  0.596805  0.206884  0.090723  0.791345 -0.078844  1.100000
 5.778011  0.475828  0.009069  0.045628 -0.034223  0.000000  0.385472  0.108619 -0.038656  0.000000  0.778220 -0.158938  0.000000 -0.190739  0.000000  0.000000
16.196356  0.853271  0.013851  0.147304 -0.094461  0.000000 -0.104049  0.157592  0.057016  0.000000  0.056511  0.004459  0.000000  0.105681  0.000000  0.000000
```

```
79.879018  0.116918 -0.016815 -0.021282 -0.007262  0.000000 -0.245425  0.214619  0.063589  0.000000  0.101451 -0.034357  0.000000 -0.013187  0.000000  0.000000

Ne aug-SVP-MOLOPT-ae
   2
1 0 0 6 1
2146.955475  0.009164
393.641936  0.049361
110.126828  0.168538
37.831538  0.370563
14.691093  0.416492
 6.050603  0.130334
1 0 2 7 2 2 1
 0.200000  0.000000 519.483919  0.000000 -0.118562  0.798343
 0.402851  0.240706  0.148177  0.101708  0.101720  0.097625
 0.878871  0.595117  0.186261  0.425860  0.149181  0.100000
 2.023646  0.250242  0.000000  0.418036  0.000000  0.000000
 5.259829 -0.033785  0.000000  0.173897  0.000000  0.000000
16.923558 -0.046992  0.000000  0.037679  0.000000  0.000000
85.504429 -0.013253  0.000000  0.003760  0.000000  0.000000

Ne aug-DZVP-MOLOPT-ae
   2
1 0 1 7 1 1
3598.723056 -0.008853  0.000045
541.377061 -0.061566 -0.001395
122.577688 -0.286486 -0.013281
34.344225 -0.690810 -0.091751
11.862251 -0.652710 -0.318598
 4.352450 -0.102970 -0.938434
 3.000251 -0.014335 -0.096131
1 0 3 4 3 2 2 1
 0.100000  0.000000  0.000000 73.487352  0.000000 -12.521440  0.000000 37.977328 18.868492
 0.150878  0.237663  0.722307 -29.320556  0.176980 23.379368 -0.034706 43.634481 -4.656560
 0.431798  0.411606 -0.680503  0.167893  0.600292  2.587296  0.779196  3.474725  1.578583
 1.385195  0.879828  0.123244 -1.150859  0.779954  4.474582  0.625819  7.116081  0.970759

Ne aug-TZVP-MOLOPT-ae
   3
1 0 0 6 1
45069.563080  0.000991
6755.976770  0.006208
1537.582966  0.034684
435.609766  0.135357
142.111689  0.419250
51.715998  0.897031
1 0 1 4 1 1
99.424302  0.024266  0.049593
21.924191  0.626033  0.299650
 8.052049  0.523306  0.681578
 1.812539 -0.577620  0.665734
1 0 4 5 4 3 3 2 1
 0.100000  0.000000  0.000000  0.000000  3.903934  0.000000  0.000000 42.647159  0.000000  0.000000 -0.055539  0.000000 -0.379349 -0.508250
 0.199299 -0.278299 -0.421655  0.860034 -0.058134  0.462599  0.844762 -5.283491  0.175662 -0.010062  0.074530 -0.136833  2.274061 -0.923563
 0.622392  0.433361  0.745838  0.498984  0.116366  0.659525 -0.485586 -4.699239  0.041467  0.659358  0.094630  0.047842  0.546054 -1.597865
 1.940818  0.847891 -0.502239  0.041798  0.092213  0.523211 -0.194658  0.070714  0.205571  0.729285  0.097882  0.320031 -0.013091  0.140883
 4.656439 -0.125814  0.116998  0.098030  0.000000  0.277990  0.112658  0.000000  0.961854 -0.182454  0.000000  0.936252  0.000000  0.000000

Na aug-SVP-MOLOPT-ae
   3
1 0 0 6 1
2600.756771  0.009164
476.845907  0.049361
133.404301  0.168538
45.827978  0.370563
17.796345  0.416492
 7.329518  0.130334
1 0 1 6 1 1
124.842404 -0.013253  0.003760
24.709570 -0.046992  0.037679
 7.679716 -0.033785  0.173897
 2.954664  0.250242  0.418036
 1.283212  0.595117  0.425860
 0.588190  0.240706  0.101708
1 0 2 7 2 2 1
 0.050000  0.000000 90.695687  0.000000 44.746227 141.219206
 0.108147  0.276059 -1.483592  0.105882 -5.948327  0.114440
 0.209845  0.735491  0.098191  0.488962  3.666153 -1.330880
 0.424059  0.151064 -4.266305  0.414510 -0.140511 -1.452150
 0.947368 -0.178503  0.099990  0.076216  0.478146 -1.209458
 2.526244 -0.071003  0.000000 -0.018293  0.000000  0.000000
 9.433006 -0.007943  0.000000 -0.007139  0.000000  0.000000

Na aug-DZVP-MOLOPT-ae
   3
1 0 0 5 1
4098.270759 -0.009259
616.747254 -0.070009
138.821600 -0.304384
40.548805 -0.697319
14.109896 -0.645066
1 0 0 5 3
19.238720  0.186427 -0.049661  0.038226
 5.090226 -0.593290  0.731461 -0.290686
 1.696440 -0.616910 -0.548468  0.023719
 0.050328 -0.192087  0.223694  0.954849
 0.553126 -0.442466 -0.334128 -0.041718
1 0 3 7 1 3 2 1
 0.045196 -2.310284  0.169376  0.946740  6.699438  0.827194 -0.107147 37.201983
 0.100000  0.201125  0.000000  0.000000 -0.987779  0.000000  2.110352  0.959411
 0.305164  0.094298  0.278766  0.192517  0.100193  0.548116  0.101403  0.100000
 1.013846  0.100135  0.655431 -0.242877  0.099903 -0.022355  0.106077  0.100000
 3.279754  0.000000  0.633221 -0.062497  0.000000  0.110184  0.000000  0.000000
12.049726  0.000000  0.248963 -0.060987  0.000000 -0.050265  0.000000  0.000000
75.335905  0.000000  0.032554 -0.002300  0.000000  0.012333  0.000000  0.000000

Na aug-TZVP-MOLOPT-ae
   3
1 0 0 7 1
26041.211692  0.000997
3906.215052  0.007200
889.014603  0.038121
```

```
251.476040  0.143642
81.689666  0.416424
28.448251  0.744357
10.256970  0.500385
1 0 1 5 1 1
138.067005 -0.005216  0.008677
32.126148  0.152961  0.078175
 8.852389  0.253270  0.334122
 3.035254 -0.804963  0.582899
 0.925242  0.514262  0.736481
1 0 3 6 4 4 4 1
 0.024986 -0.169607  0.801883  0.054655  8.926008  0.290511  0.875060 -0.178346 28.478589 -0.081649  0.029952  0.561296  7.954102 -0.260188
 0.042000  0.000000  0.000000  0.000000  1.111106  0.000000  0.000000  0.000000 -16.580115  0.000000  0.000000  0.000000 -6.606032  0.100000
 0.071573  0.284223  0.109717  0.938941  0.100000  0.426396  0.174075  0.784518  0.100000  0.150175  0.292583  0.781420  0.100000  0.100000
 0.317258  0.333295 -0.485233  0.085595  0.100000  0.709674 -0.451166 -0.017186  0.100000 -0.067811  0.935796 -0.256630  0.100000  0.100000
 0.895326  0.845523  0.328114 -0.326247  0.100000 -0.206226 -0.002514  0.074705  0.100000  0.442928 -0.162309 -0.025663  0.100000  0.100000
 1.873645  0.253883 -0.042858 -0.040483  0.100000  0.433156 -0.020262 -0.588938  0.100000  0.877495  0.106958 -0.088383  0.100000  0.100000

Mg aug-SVP-MOLOPT-ae
   3
1 0 0 6 1
3103.386324  0.009164
569.002485  0.049361
159.186391  0.168538
54.684822  0.370563
21.235716  0.416492
 8.746041  0.130334
1 0 1 6 1 1
156.795232 -0.013253  0.003760
31.033868 -0.046992  0.037679
 9.645303 -0.033785  0.173897
 3.710896  0.250242  0.418036
 1.611645  0.595117  0.425860
 0.738735  0.240706  0.101708
1 0 2 7 2 2 1
 0.050000  0.000000 -0.453104  0.000000 416.892309  3.960342
 0.102056  0.276059  6.276573  0.105882 104.911857  7.563685
 0.198026  0.735491 -3.049442  0.488962  3.368429  1.849246
 0.400174  0.151064  0.501107  0.414510 -99.375002  0.049818
 0.894006 -0.178503  0.333016  0.076216 -10.284186  0.276459
 2.383949 -0.071003  0.000000 -0.018293  0.000000  0.000000
 8.901678 -0.007943  0.000000 -0.007139  0.000000  0.000000

Mg aug-DZVP-MOLOPT-ae
   3
1 0 0 5 1
4954.047690 -0.009174
745.091200 -0.062439
169.138165 -0.294905
45.390154 -0.745322
14.013742 -0.594595
1 0 0 5 3
24.820315  0.107773  0.041287 -0.007699
 2.496961 -0.687673 -0.493260  0.507377
 0.850548 -0.559016 -0.075345 -0.818394
 0.117509 -0.399029  0.699256  0.269703
 0.045075 -0.209206  0.510249  0.000653
1 0 3 8 1 4 2 1
 0.060437 223.043845 -0.208880  0.643916  0.700561  2.718034  0.682569  0.118861  4.073808
 0.110000 -241.939105  0.000000  0.000000  0.000000 -0.676368  0.000000  9.572136  0.100000
 0.229420  0.100000 -0.305708  0.671579 -0.632873  0.100000  0.705191  0.100000  0.100000
 0.778278  0.100000 -0.432100 -0.153627 -0.227287  0.100000  0.188522  0.100000  0.100000
 2.211078  0.000000 -0.671790 -0.279264  0.232304  0.000000  0.028370  0.000000  0.000000
 6.646124  0.000000 -0.453045 -0.171703 -0.036075  0.000000  0.020543  0.000000  0.000000
22.627201  0.000000 -0.138700 -0.056827  0.041517  0.000000 -0.005473  0.000000  0.000000
97.890192  0.000000 -0.020294 -0.007332 -0.006030  0.000000  0.002598  0.000000  0.000000

Mg aug-TZVP-MOLOPT-ae
   3
1 0 0 7 1
31438.408440  0.000971
4715.551058  0.007166
1073.121768  0.037417
303.646056  0.142891
98.439313  0.412095
34.863642  0.729010
12.946643  0.526166
1 0 1 5 1 1
179.629327  0.001600  0.009071
42.016549  0.191892  0.069602
12.311363  0.394845  0.305664
 4.184588 -0.843197  0.623403
 1.316573  0.310307  0.716249
1 0 3 6 4 4 4 1
 0.046225  0.238637  0.642095  0.718758 -0.094209 -0.414473 -0.157895  0.692235 16.426238 -0.176477  0.264945  0.933054 32.254177 -1.360829
 0.070000  0.000000  0.000000  0.000000  0.033564  0.000000  0.000000  0.000000  0.299976  0.000000  0.000000  0.000000  0.103198  5.596857
 0.134753  0.277594  0.666876 -0.689916  0.071462 -0.087757  0.804173  0.445405 -2.366132  0.267717  0.700676 -0.030586 -63.453336  1.397219
 0.461937  0.189989 -0.175060 -0.062538  5.283707  0.789642 -0.004757  0.337727 -0.155238 -0.280060  0.660207 -0.346882 80.036634 -0.135339
 1.234435  0.860078 -0.320698  0.048733  0.100000 -0.281744 -0.455189  0.252398  0.100000 -0.332750  0.005086 -0.062273  0.100000  0.100000
 2.432121  0.300273 -0.097455 -0.033430  0.000000  0.342923 -0.348072  0.380343  0.000000  0.841444  0.054387  0.065342  0.000000  0.000000

Al aug-SVP-MOLOPT-ae-SR
  3
1 0 0 6 1
3644.586388  0.009164
668.230925  0.049361
186.946932  0.168538
64.221317  0.370563
24.939017  0.416492
10.271264  0.130334
1 0 1 6 1 1
195.964144 -0.013253  0.003760
38.786418 -0.046992  0.037679
12.054790 -0.033785  0.173897
 4.637912  0.250242  0.418036
 2.014248  0.595117  0.425860
 0.923277  0.240706  0.101708
1 0 2 6 2 2 1
 0.102056  0.276059 -1.758622  0.105882  9.752990  0.587631
 0.198026  0.735491  0.645717  0.488962 -1.322655  0.324993
 0.400174  0.151064  0.203500  0.414510  1.958622  0.134808
```

```
 0.894006 -0.178503  0.219418  0.076216 -0.234276  0.085183
 2.383949 -0.071003  0.000000 -0.018293  0.000000  0.000000
 8.901678 -0.007943  0.000000 -0.007139  0.000000  0.000000

Al aug-SVP-MOLOPT-ae
   3
1 0 0 6 1
3644.586388  0.009164
668.230925  0.049361
186.946932  0.168538
64.221317  0.370563
24.939017  0.416492
10.271264  0.130334
1 0 1 6 1 1
195.964144 -0.013253  0.003760
38.786418 -0.046992  0.037679
12.054790 -0.033785  0.173897
 4.637912  0.250242  0.418036
 2.014248  0.595117  0.425860
 0.923277  0.240706  0.101708
1 0 2 7 2 2 1
 0.050000  0.000000 119.859557  0.000000 29.866340 -0.012273
 0.102056  0.276059 32.068019  0.105882 26.165454  0.256402
 0.198026  0.735491 -56.097165  0.488962 -0.793066  0.468325
 0.400174  0.151064 -2.598654  0.414510  3.842264  0.283099
 0.894006 -0.178503 -1.057614  0.076216 -0.518034  0.100000
 2.383949 -0.071003  0.000000 -0.018293  0.000000  0.000000
 8.901678 -0.007943  0.000000 -0.007139  0.000000  0.000000

Al aug-DZVP-MOLOPT-ae-SR
   3
1 0 0 5 1
5887.765650  0.008083
885.617931  0.064661
201.176620  0.273826
56.172899  0.731654
16.856998  0.620850
1 0 0 5 3
29.250065  0.115111  0.003546 -0.029429
 3.027549 -0.724058 -0.273522  0.358786
 1.086592 -0.675452  0.186793 -0.385986
 0.227406 -0.078708  0.932463  0.051567
 0.093278  0.007683  0.144204  0.847799
1 0 3 8 1 4 2 1
 0.071899 26.257580  0.087131  0.066194  0.969831 43.307584  0.297590 22.309051  5.566539
 0.140000 -5.351606  0.000000  0.000000  0.000000  0.207837  0.000000  0.098161  0.101569
 0.280430  0.100000  0.050606  0.988366 -0.041747  0.100000  0.906701  0.100000  0.100000
 1.054693  0.100000  0.489491  0.074233 -0.206708  0.100000  0.297306  0.100000  0.100000
 3.275178  0.000000  0.761624 -0.113823  0.074195  0.000000 -0.009869  0.000000  0.000000
10.287012  0.000000  0.397854 -0.009934 -0.096822  0.000000  0.028601  0.000000  0.000000
33.685547  0.000000  0.107745 -0.013651  0.008023  0.000000 -0.004557  0.000000  0.000000
145.052984  0.000000  0.016669 -0.000420 -0.003587  0.000000 -0.002660  0.000000  0.000000

Al aug-DZVP-MOLOPT-ae
   3
1 0 0 5 1
5887.765650  0.008083
885.617931  0.064661
201.176620  0.273826
56.172899  0.731654
16.856998  0.620850
1 0 0 5 3
29.250065  0.115111  0.003546 -0.029429
 3.027549 -0.724058 -0.273522  0.358786
 1.086592 -0.675452  0.186793 -0.385986
 0.227406 -0.078708  0.932463  0.051567
 0.093278  0.007683  0.144204  0.847799
1 0 3 8 1 4 2 1
 0.050000  9.500359  0.000000  0.000000  0.000000 247.400227  0.000000 -0.530932 37.646944
 0.071899  0.758689  0.087131  0.066194  0.969831  6.354700  0.297590 -4.386889 13.067107
 0.280430 -0.915857  0.050606  0.988366 -0.041747 -4.932951  0.906701  0.002004  1.739716
 1.054693 -0.105825  0.489491  0.074233 -0.206708 -2.002472  0.297306  0.043910 -1.330072
 3.275178  0.139633  0.761624 -0.113823  0.074195 -0.923996 -0.009869  0.004294  0.138615
10.287012  0.000000  0.397854 -0.009934 -0.096822  0.000000  0.028601  0.000000  0.000000
33.685547  0.000000  0.107745 -0.013651  0.008023  0.000000 -0.004557  0.000000  0.000000
145.052984  0.000000  0.016669 -0.000420 -0.003587  0.000000 -0.002660  0.000000  0.000000

Al aug-TZVP-MOLOPT-ae
   3
1 0 0 7 1
37792.562568  0.000909
5668.107384  0.007105
1289.982926  0.036123
364.877998  0.140425
118.653376  0.401302
42.008686  0.730450
15.356196  0.533221
1 0 1 5 1 1
452.528436  0.005693  0.002543
107.119023  0.046547  0.030558
34.047655  0.189716  0.114480
12.525255  0.211067  0.435472
 4.333798 -0.957737  0.892367
1 0 4 6 4 5 4 2 1
 0.064494  0.001612  0.045967  0.970013 10.604582 -0.038721  0.018194 -0.073004  0.995756 -0.006650 -0.061025 -0.011351  0.229145  1.100000 -0.075928  5.677413  0.100383
 0.110000  0.000000  0.000000  0.000000 -7.515229  0.000000  0.000000  0.000000  0.000000  0.413321  0.000000  0.000000  0.000000  0.072240  0.000000  1.718034  0.132786
 0.201490  0.046383  0.997323 -0.039102  0.155756  0.101174  0.247034  0.958917  0.072030  0.099886 -0.095984 -0.067376  0.958591 -0.289600  0.868955 -0.749018  2.100000
 0.613444  0.252689  0.010239 -0.231961  0.322438 -0.105869  0.965599 -0.233172 -0.039642  0.093021  0.029643  0.996860  0.072983  1.323707  0.488882  0.094609  0.086652
 1.609191  0.964447 -0.052041  0.061140  0.161604  0.988162  0.078990 -0.127671  0.028143  0.098518  0.992245 -0.036312  0.099947  0.099953  0.004686  0.099981  0.100000
 4.148366 -0.061941 -0.020518  0.001656  0.000000  0.024342 -0.004797  0.066928 -0.030288  0.000000  0.040415 -0.016812  0.115244  0.000000 -0.011172  0.000000  0.000000

Si aug-SVP-MOLOPT-ae-SR
   3
1 0 0 6 1
4229.261737  0.009164
775.430510  0.049361
216.937513  0.168538
74.523891  0.370563
28.939808  0.416492
11.919011  0.130334
1 0 1 6 1 1
```

```
240.490485 -0.013253  0.003760
47.599343 -0.046992  0.037679
14.793840 -0.033785  0.173897
 5.691724  0.250242  0.418036
 2.471919  0.595117  0.425860
 1.133062  0.240706  0.101708
1 0 2 6 2 2 1
 0.108147  0.276059 188.707875  0.105882 194.295508  0.389592
 0.209845  0.735491 -43.000376  0.488962 27.361652  0.231623
 0.424059  0.151064  6.047305  0.414510 71.223696  0.103694
 0.947368 -0.178503 -0.043191  0.076216 -2.670489  0.100000
 2.526244 -0.071003  0.000000 -0.018293  0.000000  0.000000
 9.433006 -0.007943  0.000000 -0.007139  0.000000  0.000000

Si aug-SVP-MOLOPT-ae
   3
1 0 0 6 1
4229.261737  0.009164
775.430510  0.049361
216.937513  0.168538
74.523891  0.370563
28.939808  0.416492
11.919011  0.130334
1 0 1 6 1 1
240.490485 -0.013253  0.003760
47.599343 -0.046992  0.037679
14.793840 -0.033785  0.173897
 5.691724  0.250242  0.418036
 2.471919  0.595117  0.425860
 1.133062  0.240706  0.101708
1 0 2 7 2 2 1
 0.050000  0.000000 355.890247  0.000000 67.471003 -0.067509
 0.108147  0.276059 -104.082871  0.105882 39.415820  0.301676
 0.209845  0.735491 -2.616436  0.488962  5.486054  0.321514
 0.424059  0.151064 -3.116258  0.414510  1.244080  0.205447
 0.947368 -0.178503 -0.470796  0.076216 -0.545309  0.100000
 2.526244 -0.071003  0.000000 -0.018293  0.000000  0.000000
 9.433006 -0.007943  0.000000 -0.007139  0.000000  0.000000

Si aug-DZVP-MOLOPT-ae-SR
   3
1 0 0 5 1
6903.595265  0.007904
1038.479710  0.063722
235.942605  0.270806
66.091579  0.727061
19.821530  0.627633
1 0 0 5 3
34.220232  0.119411 -0.009335 -0.011802
 3.601959 -0.752601 -0.118832  0.213707
 1.334327 -0.636977 -0.036082 -0.303574
 0.333383 -0.104711  0.976318 -0.151299
 0.146508 -0.051271  0.176899  0.916047
1 0 3 8 1 4 2 1
 0.100481 41.412214 -0.120919  0.020833  0.977664 27.516409  0.164501 34.825756  0.954102
 0.180000 -19.526072  0.000000  0.000000  0.000000  5.401298  0.000000  1.247372  1.500431
 0.360587  1.643143 -0.051325  0.991817 -0.007646 -8.061090  0.951090  2.772145  0.354662
 1.403852  0.260084  0.526179  0.120628 -0.064339  0.137626  0.260629 -4.136068  0.100000
 4.231639  0.000000  0.735765 -0.026834  0.199359  0.000000  0.005353  0.000000  0.000000
12.916447  0.000000  0.392364  0.021982  0.009823  0.000000  0.017155  0.000000  0.000000
41.955626  0.000000  0.101652  0.010533  0.010493  0.000000 -0.009671  0.000000  0.000000
179.802308  0.000000  0.015741  0.000031  0.004897  0.000000  0.004861  0.000000  0.000000

Si aug-DZVP-MOLOPT-ae
   3
1 0 0 5 1
6903.595265  0.007904
1038.479710  0.063722
235.942605  0.270806
66.091579  0.727061
19.821530  0.627633
1 0 0 5 3
34.220232  0.119411 -0.009335 -0.011802
 3.601959 -0.752601 -0.118832  0.213707
 1.334327 -0.636977 -0.036082 -0.303574
 0.333383 -0.104711  0.976318 -0.151299
 0.146508 -0.051271  0.176899  0.916047
1 0 3 9 1 4 2 1
 0.060000 50.917798  0.000000  0.000000  0.000000  2.280340  0.000000 -4.136068 -0.042218
 0.100481 -25.125685 -0.120919  0.020833  0.977664  0.619855  0.164501 -153.802044  0.011192
 0.180000  2.859087  0.000000  0.000000  0.000000  1.079503  0.000000 -184.682372  1.100000
 0.360587  0.481966 -0.051325  0.991817 -0.007646  0.481966  0.951090  0.100000  0.100000
 1.403852  0.100000  0.526179  0.120628 -0.064339  0.100000  0.260629  0.100000  0.100000
 4.231639  0.000000  0.735765 -0.026834  0.199359  0.000000  0.005353  0.000000  0.000000
12.916447  0.000000  0.392364  0.021982  0.009823  0.000000  0.017155  0.000000  0.000000
41.955626  0.000000  0.101652  0.010533  0.010493  0.000000 -0.009671  0.000000  0.000000
179.802308  0.000000  0.015741  0.000031  0.004897  0.000000  0.004861  0.000000  0.000000

Si aug-TZVP-MOLOPT-ae
   3
1 0 0 7 1
44773.460346  0.000909
6717.203689  0.006763
1528.869958  0.035652
432.532950  0.136273
140.584919  0.396770
49.878252  0.722820
18.295805  0.547921
1 0 1 5 1 1
394.440960  0.015374  0.005682
93.116343  0.071105  0.046751
29.607340  0.251672  0.201465
10.791845  0.026712  0.560691
 4.281689 -0.964705  0.801760
1 0 4 7 4 5 4 2 1
 0.045000  0.000000  0.000000  0.000000  6.650703  0.000000  0.000000  0.000000  0.000000  2.539232  0.000000  0.000000  0.000000 -0.019320  0.000000 12.337785 67.898227
 0.079418  0.100476  0.090631  0.990652 -2.621832  0.007260 -0.010791  0.020096  0.999000 -0.959773 -0.101355  0.061665  0.112266  0.065238 -0.027974  1.499468  2.338952
 0.135000  0.000000  0.000000  0.000000  0.104313  0.000000  0.000000  0.000000  0.000000  1.059804  0.000000  0.000000  0.000000  1.097651  0.000000 -1.913304 -0.438496
 0.253847 -0.005475  0.994604 -0.090333 -1.322403  0.101433  0.010725  0.992575 -0.018179  1.114449 -0.022837  0.025924  0.987886  0.099218  0.936833  0.237774  0.572160
 0.818669  0.315328  0.033282 -0.024635  0.099862 -0.063918  0.996634 -0.007146  0.013230  0.154133  0.004007  0.997623 -0.034271  0.094083  0.311251  0.001476 -0.900051
 1.861308  0.940722 -0.017691 -0.096156  0.188944  0.991702  0.060774 -0.104806 -0.002820  0.106183  0.991917  0.001659  0.027183  0.096741  0.143457  0.026923  0.419520
```

```
 4.867591 -0.074065 -0.033574  0.024403  0.000000  0.045824  0.052884  0.057924 -0.038548  0.000000  0.072738  0.016479  0.097798  0.000000 -0.064069  0.000000  0.000000

P aug-SVP-MOLOPT-ae
   3
1 0 0 6 1
4857.412371  0.009164
890.601241  0.049361
249.158133  0.168538
85.592543  0.370563
33.238089  0.416492
13.689281  0.130334
1 0 1 6 1 1
290.664959 -0.013253  0.003760
57.530181 -0.046992  0.037679
17.880337 -0.033785  0.173897
 6.879210  0.250242  0.418036
 2.987646  0.595117  0.425860
 1.369457  0.240706  0.101708
1 0 2 7 2 2 1
 0.072000  0.000000  2.674609  0.000000 54.208827  0.301264
 0.127481  0.276059 1175.932372  0.105882 -2.905477  0.436349
 0.247361  0.735491 -307.918621  0.488962 18.442408  0.863079
 0.499871  0.151064 -4.861046  0.414510  8.855318  0.513459
 1.116734 -0.178503  1.052769  0.076216  0.644430  0.198751
 2.977874 -0.071003 40.091742 -0.018293  0.762111  0.091302
11.119397 -0.007943  0.000000 -0.007139  0.000000  0.000000

P aug-DZVP-MOLOPT-ae
   3
1 0 0 5 1
8002.481553  0.007944
1203.619999  0.062506
273.354631  0.268040
76.535938  0.726336
22.798786  0.629778
1 0 0 5 3
39.914622  0.116692 -0.026585  0.001229
 4.194333 -0.757266  0.105153 -0.003055
 1.574628 -0.639741 -0.046375 -0.040667
 0.347185  0.056489  0.985683 -0.122754
 0.115581 -0.021721  0.120476  0.991598
1 0 3 8 1 4 2 1
 0.074000  5.445391  0.000000  0.000000  0.000000 -36.039557  0.000000 34.213594  0.157570
 0.127557 -6.027849 -0.034616  0.058450  0.995021 15.717781  0.117319 49.847964  4.775371
 0.443884 38.944212 -0.068364  0.987155 -0.050595 -0.860500  0.949584  3.744865  5.814973
 1.776662 -6.685561  0.521262  0.141951 -0.043684  0.315507  0.290064 -2.515180  0.039353
 5.266330 -2.518034  0.755007 -0.024680  0.073452 -0.126387 -0.002376  0.602683  0.033188
15.965140  0.000000  0.377426  0.036327 -0.005711  0.000000  0.017401  0.000000  0.000000
51.313725  0.000000  0.098570  0.003872  0.005095  0.000000 -0.008632  0.000000  0.000000
219.560856  0.000000  0.014564  0.003847 -0.003255  0.000000  0.002620  0.000000  0.000000

P aug-TZVP-MOLOPT-ae
   3
1 0 0 7 1
52427.011820  0.000772
7863.314290  0.006699
1789.606582  0.034464
506.366489  0.133567
164.622579  0.388407
58.421506  0.719833
21.516978  0.558486
1 0 1 5 1 1
472.277513 -0.001085  0.006002
111.671172  0.023602  0.043931
35.448363  0.270985  0.206339
12.887584  0.051783  0.557273
 5.350546 -0.960899  0.803060
1 0 4 6 4 5 4 2 1
 0.074000  0.000000  0.000000  0.000000  3.195725  0.000000  0.000000  0.000000  0.000000  0.087836  0.000000  0.000000  0.000000  0.286369  0.000000 11.607092  0.354009
 0.094240  0.087449  0.138293  0.981804 -3.781261  0.006623  0.003957  0.099595  0.992936  0.065567  0.002820  0.007485  0.107722  0.370953 -0.066684 -5.769132  0.098279
 0.293985  0.031987  0.987004 -0.133722  0.584395 -0.079913 -0.009192  0.988068 -0.092526  0.097152 -0.036016  0.006703  0.989778  0.108203  0.918614  0.415910  0.489102
 0.953573  0.292027  0.053185 -0.126112  0.109706 -0.045273  0.998951  0.005058 -0.004053 -0.034148 -0.067977  0.996250 -0.014329  0.099694  0.384852 -0.122670  0.057567
 2.325184  0.948199 -0.062046 -0.046131  0.000000  0.995713  0.044642  0.078100 -0.013576  0.000000  0.994044  0.071992  0.040999  0.000000  0.045116  0.000000  0.000000
 5.019530  0.083479 -0.004360 -0.012109  0.000000  0.008721  0.001705  0.087624 -0.072910  0.000000 -0.077145  0.046925  0.082756  0.000000 -0.039426  0.000000  0.000000

S aug-SVP-MOLOPT-ae
   3
1 0 0 6 1
5529.038289  0.009164
1013.743118  0.049361
283.608793  0.168538
97.427275  0.370563
37.833862  0.416492
15.582074  0.130334
1 0 1 6 1 1
345.589679 -0.013253  0.003760
68.401217 -0.046992  0.037679
21.259047 -0.033785  0.173897
 8.179122  0.250242  0.418036
 3.552198  0.595117  0.425860
 1.628232  0.240706  0.101708
1 0 2 7 2 2 1
 0.072000  0.000000 467.861244  0.000000 -1.942811  0.069678
 0.148404  0.276059 3002.942894  0.105882  0.596352  0.269845
 0.287959  0.735491 -1935.160935  0.488962 -0.362699  0.241104
 0.581913  0.151064  5.677435  0.414510  0.342372  0.142020
 1.300021 -0.178503 102.128177  0.076216 -0.044255  0.103062
 3.466625 -0.071003  0.000000 -0.018293  0.000000  0.000000
12.944394 -0.007943  0.000000 -0.007139  0.000000  0.000000

S aug-DZVP-MOLOPT-ae
   3
1 0 0 5 1
9184.968607 -0.007718
1381.514008 -0.062404
313.892819 -0.264955
87.514579 -0.729865
25.931396 -0.627010
1 0 0 5 3
45.482850 -0.117171 -0.031668  0.007982
 4.876333  0.761444  0.127228 -0.028018
```

```
 1.864066  0.626717 -0.010132 -0.072618
 0.430250 -0.111972  0.938663 -0.317639
 0.152872  0.034089  0.318777  0.944978
1 0 3 8 1 4 2 1
 0.070000 73.110205  0.000000  0.000000  0.000000 -0.039361  0.000000  0.243142  0.050596
 0.132026 -30.140563 -0.077140  0.135030  0.987631 -0.108174  0.259682  0.510705  0.050595
 0.461903 -7.157956  0.055730  0.972480 -0.125790  0.229154  0.917114  0.073933  0.099934
 2.043261  0.000000 -0.483922  0.173438 -0.070001  0.000000  0.300876  0.000000  0.000000
 6.030247  0.000000 -0.773455 -0.068115 -0.040760  0.000000 -0.022008  0.000000  0.000000
18.817576  0.000000 -0.386400  0.034707 -0.046462  0.000000  0.020171  0.000000  0.000000
61.243244  0.000000 -0.095007 -0.009977  0.002545  0.000000 -0.006549  0.000000  0.000000
262.037180  0.000000 -0.014078  0.005052 -0.005920  0.000000  0.002736  0.000000  0.000000

S aug-TZVP-MOLOPT-ae
   3
1 0 0 7 1
60700.943664  0.000862
9102.630460  0.006581
2071.464789  0.033761
585.934823  0.131917
190.479130  0.383016
67.668075  0.719277
24.765148  0.563341
1 0 1 5 1 1
564.299947 -0.001006  0.005440
133.435938  0.019580  0.040141
42.458538  0.179492  0.194774
15.602047  0.076002  0.512907
 6.412455 -0.980623  0.835074
1 0 4 6 4 5 4 2 1
 0.070000  0.000000  0.000000  0.000000  1.296559  0.000000  0.000000  0.000000  0.000000 -0.460870  0.000000  0.000000  0.000000 -0.232788  0.000000  1.909034  0.016902
 0.108835  0.108719  0.305589  0.944524 -1.246252 -0.033340  0.088666 -0.010173  0.995245  0.224960 -0.086923  0.178316  0.179025  2.361811  0.052133  0.945211  0.135580
 0.337374  0.042449  0.942544 -0.302973  0.121718 -0.033606 -0.039580  0.992635  0.014753  0.096021  0.000700 -0.056554  0.979623  0.519678  0.908394  1.551672  0.100478
 0.960895  0.208479  0.084602 -0.104338 -0.063145  0.056905  0.965810  0.066607 -0.088086  0.065918  0.079411  0.979537  0.028261 -1.286287  0.412245  0.189551  0.114579
 2.588982  0.962071 -0.099235 -0.067190  0.000000  0.997047 -0.048659  0.027326  0.038744  0.000000  0.988168 -0.058435  0.004184  0.000000  0.032167  0.000000  0.000000
 6.979742 -0.131656 -0.034944  0.026075  0.000000 -0.020468  0.235405 -0.096895 -0.002976  0.000000  0.098290 -0.045809  0.086444  0.000000 -0.033491  0.000000  0.000000

Cl aug-SVP-MOLOPT-ae
   3
1 0 0 6 1
6236.545525  0.009164
1143.463795  0.049361
319.899964  0.168538
109.894271  0.370563
42.675161  0.416492
17.575988  0.130334
1 0 1 6 1 1
403.972966 -0.013253  0.003760
79.956793 -0.046992  0.037679
24.850512 -0.033785  0.173897
 9.560888  0.250242  0.418036
 4.152300  0.595117  0.425860
 1.903303  0.240706  0.101708
1 0 2 7 2 2 1
 0.077000  0.000000 1628.424936  0.000000  1.389004  0.359474
 0.155732  0.276059 -12.034434  0.105882  0.412356  0.054033
 0.302177  0.735491 -170.169929  0.488962  0.062609  1.055343
 0.610646  0.151064  2.864808  0.414510 -0.060482  0.253992
 1.364210 -0.178503 -1.083690  0.076216  0.102208  0.500781
 3.637791 -0.071003 -5.177528 -0.018293  0.081826  0.100310
13.583529 -0.007943  0.000000 -0.007139  0.000000  0.000000

Cl aug-DZVP-MOLOPT-ae
   3
1 0 0 5 1
10449.738660  0.007930
1571.781775  0.062168
357.173295  0.269577
100.086675  0.719173
30.610026  0.637330
1 0 0 5 3
51.912451 -0.118096 -0.064038  0.038963
 5.604127  0.774484 -0.194653  0.574322
 2.190751  0.612386  0.385229 -0.660064
 0.564844 -0.050221  0.597599  0.462893
 0.202002 -0.093217  0.672669  0.136681
1 0 3 8 1 4 2 1
 0.077000 -0.311165  0.000000  0.000000  0.000000  3.973789  0.000000  0.564242 -0.252424
 0.154068 -0.497418 -0.034858  0.003829  0.997387  3.446014  0.443437  2.473713 -0.360879
 0.543592  0.457735  0.001773  0.986199 -0.013612  3.779106  0.860345  1.091486  1.660139
 2.471054 -0.114868 -0.507191  0.139408  0.028168  0.040810  0.251018  0.004897  0.350772
 7.247821 -0.018039 -0.768325 -0.088971 -0.063254  0.000301 -0.008307  0.045511 -0.041983
22.555186  0.000000 -0.378387 -0.000950  0.002337  0.000000  0.007213  0.000000  0.000000
72.147284  0.000000 -0.088528 -0.004945 -0.014710  0.000000 -0.003606  0.000000  0.000000
307.662624  0.000000 -0.014186  0.004537 -0.004237  0.000000  0.005124  0.000000  0.000000

Cl aug-TZVP-MOLOPT-ae
   3
1 0 0 7 1
69508.004502  0.000855
10426.074049  0.006366
2373.213726  0.033227
671.480826  0.129171
218.359533  0.378680
77.681940  0.712973
28.635695  0.574840
1 0 1 5 1 1
666.452681  0.001438  0.005818
157.540739  0.026872  0.042998
50.271480  0.223253  0.208198
18.561818  0.126050  0.559199
 7.676985 -0.966202  0.801291
1 0 4 5 4 5 4 2 1
 0.130056  0.137215  0.095926  0.985764  0.145978  0.045025 -0.047151  0.140088  0.987849  0.093127 -0.019575  0.255137  0.203731  7.602159 -0.021328 -5.757401 -2.053735
 0.412964 -0.014590  0.989285 -0.093558 -0.550269 -0.217443  0.024986  0.966833 -0.125325 -0.108829 -0.036555  0.032481  0.974024  2.854164  0.980784 -2.920918  1.888058
 1.261473  0.242813  0.104956 -0.052362 20.908293 -0.007824  0.992946 -0.016713  0.048319 -0.060800 -0.088273  0.957740 -0.091988  0.600362  0.180157  0.265678  0.426206
 3.213593  0.959550 -0.025995 -0.129366 -0.388504  0.967664  0.002777  0.212406 -0.075999 -0.049156  0.993190  0.096797  0.032687  0.148343  0.071082 -0.086276  0.118909
 7.922916 -0.035571 -0.020534 -0.006212 -1.453627  0.119404  0.105841 -0.014612  0.018345 -0.117422 -0.063740  0.084943  0.015542  0.106196  0.009927  0.025742  0.016695
```

S6. COMPLETE LIST OF GENERATED RI BASIS SETS

```
    # RI basis set for H (all-electron) relative DI metric: 1.5e-02
H  RI_aug-SZV-MOLOPT-ae-SR_N_RI_002_s_p_d_f_g_h_i_2_0_0_0_0_0_0_error_1.5e-02
   2
     1   0   0   1   1
      0.6364837502    1.0000000000
     2   0   0   1   1
      2.4579249207    1.0000000000

# RI basis set for H (all-electron) relative DI metric: 3.0e-03
H  RI_aug-SZV-MOLOPT-ae-SR_N_RI_005_s_p_d_f_g_h_i_2_1_0_0_0_0_0_error_3.0e-03
   3
     1   0   0   1   1
      0.2552786857    1.0000000000
     2   0   0   1   1
      3.6198122915    1.0000000000
     3   1   1   1   1
      0.7007968815    1.0000000000

# RI basis set for H (all-electron) relative DI metric: 2.7e-04
H  RI_aug-SZV-MOLOPT-ae-SR_N_RI_006_s_p_d_f_g_h_i_3_1_0_0_0_0_0_error_2.7e-04
   4
     1   0   0   1   1
      0.3105445218    1.0000000000
     2   0   0   1   1
      0.9651256425    1.0000000000
     3   0   0   1   1
      3.0949772213    1.0000000000
     4   1   1   1   1
      0.5432074796    1.0000000000

# RI basis set for H (all-electron) relative DI metric: 1.3e-04
H  RI_aug-SZV-MOLOPT-ae-SR_N_RI_008_s_p_d_f_g_h_i_5_1_0_0_0_0_0_error_1.3e-04
   6
     1   0   0   1   1
      0.2785826321    1.0000000000
     2   0   0   1   1
      0.5879680481    1.0000000000
     3   0   0   1   1
      1.2072643744    1.0000000000
     4   0   0   1   1
      2.6693395645    1.0000000000
     5   0   0   1   1
      5.2394990941    1.0000000000
     6   1   1   1   1
      0.4900456184    1.0000000000

# RI basis set for H (all-electron) relative DI metric: 1.3e-06
H  RI_aug-SZV-MOLOPT-ae-SR_N_RI_011_s_p_d_f_g_h_i_5_2_0_0_0_0_0_error_1.3e-06
   7
     1   0   0   1   1
      0.2200602014    1.0000000000
     2   0   0   1   1
      0.4399905525    1.0000000000
     3   0   0   1   1
      1.2502261621    1.0000000000
     4   0   0   1   1
      3.6298498522    1.0000000000
     5   0   0   1   1
      7.0326865470    1.0000000000
     6   1   1   1   1
      0.2492167327    1.0000000000
     7   1   1   1   1
      1.5273474371    1.0000000000

# RI basis set for H (all-electron) relative DI metric: 4.7e-07
H  RI_aug-SZV-MOLOPT-ae-SR_N_RI_018_s_p_d_f_g_h_i_6_4_0_0_0_0_0_error_4.7e-07
   10
     1   0   0   1   1
      0.2369109995    1.0000000000
     2   0   0   1   1
      0.5060539998    1.0000000000
     3   0   0   1   1
      0.9942179999    1.0000000000
     4   0   0   1   1
      1.7751000000    1.0000000000
     5   0   0   1   1
      4.9229699991    1.0000000000
     6   0   0   1   1
     23.3214999998    1.0000000000
     7   1   1   1   1
      0.3478919970    1.0000000000
     8   1   1   1   1
      0.6223361128    1.0000000000
     9   1   1   1   1
      1.1132829667    1.0000000000
    10   1   1   1   1
      1.9915266663    1.0000000000

# RI basis set for H (all-electron) relative DI metric: 3.5e-08
H  RI_aug-SZV-MOLOPT-ae-SR_N_RI_025_s_p_d_f_g_h_i_7_6_0_0_0_0_0_error_3.5e-08
   13
     1   0   0   1   1
      0.2369110000    1.0000000000
     2   0   0   1   1
      0.4238968856    1.0000000000
     3   0   0   1   1
      0.7584644429    1.0000000000
     4   0   0   1   1
      1.3570949226    1.0000000000
     5   0   0   1   1
      2.4282043094    1.0000000000
     6   0   0   1   1
      4.3447043169    1.0000000000
     7   0   0   1   1
      7.7738333333    1.0000000000
     8   1   1   1   1
      0.3478920000    1.0000000000
```

```
     9   1   1   1   1
      0.4931656038    1.0000000000
    10   1   1   1   1
      0.6991029192    1.0000000000
    11   1   1   1   1
      0.9910360493    1.0000000000
    12   1   1   1   1
      1.4048753396    1.0000000000
    13   1   1   1   1
      1.9915266667    1.0000000000

# RI basis set for H (all-electron) relative DI metric: 1.3e-08
H  RI_aug-SZV-MOLOPT-ae-SR_N_RI_035_s_p_d_f_g_h_i_7_6_2_0_0_0_0_error_1.3e-08
   15
     1   0   0   1   1
      0.2369110000    1.0000000000
     2   0   0   1   1
      0.4238968856    1.0000000000
     3   0   0   1   1
      0.7584644429    1.0000000000
     4   0   0   1   1
      1.3570949226    1.0000000000
     5   0   0   1   1
      2.4282043094    1.0000000000
     6   0   0   1   1
      4.3447043169    1.0000000000
     7   0   0   1   1
      7.7738333333    1.0000000000
     8   1   1   1   1
      0.3478920000    1.0000000000
     9   1   1   1   1
      0.4931656038    1.0000000000
    10   1   1   1   1
      0.6991029192    1.0000000000
    11   1   1   1   1
      0.9910360493    1.0000000000
    12   1   1   1   1
      1.4048753396    1.0000000000
    13   1   1   1   1
      1.9915266667    1.0000000000
    14   2   2   1   1
      0.6253660000    1.0000000000
    15   2   2   1   1
      1.2860533333    1.0000000000

# RI basis set for H (all-electron) relative DI metric: 1.3e-02
H  RI_aug-SZV-MOLOPT-ae_N_RI_002_s_p_d_f_g_h_i_2_0_0_0_0_0_0_error_1.3e-02
   2
     1   0   0   1   1
      0.4805569653    1.0000000000
     2   0   0   1   1
      2.1834666323    1.0000000000

# RI basis set for H (all-electron) relative DI metric: 1.3e-04
H  RI_aug-SZV-MOLOPT-ae_N_RI_006_s_p_d_f_g_h_i_3_1_0_0_0_0_0_error_1.3e-04
   4
     1   0   0   1   1
      0.4327270288    1.0000000000
     2   0   0   1   1
      1.0802448276    1.0000000000
     3   0   0   1   1
      4.1159522843    1.0000000000
     4   1   1   1   1
      0.3741982228    1.0000000000

# RI basis set for H (all-electron) relative DI metric: 4.4e-05
H  RI_aug-SZV-MOLOPT-ae_N_RI_007_s_p_d_f_g_h_i_4_1_0_0_0_0_0_error_4.4e-05
   5
     1   0   0   1   1
      0.1487770976    1.0000000000
     2   0   0   1   1
      0.5105034533    1.0000000000
     3   0   0   1   1
      1.1063661205    1.0000000000
     4   0   0   1   1
      5.9552917212    1.0000000000
     5   1   1   1   1
      0.3674521516    1.0000000000

# RI basis set for H (all-electron) relative DI metric: 2.4e-06
H  RI_aug-SZV-MOLOPT-ae_N_RI_010_s_p_d_f_g_h_i_4_2_0_0_0_0_0_error_2.4e-06
   6
     1   0   0   1   1
      0.1764834857    1.0000000000
     2   0   0   1   1
      0.4925801649    1.0000000000
     3   0   0   1   1
      1.1727895186    1.0000000000
     4   0   0   1   1
      4.9135650575    1.0000000000
     5   1   1   1   1
      0.2711921775    1.0000000000
     6   1   1   1   1
      1.6074447029    1.0000000000

# RI basis set for H (all-electron) relative DI metric: 9.8e-07
H  RI_aug-SZV-MOLOPT-ae_N_RI_011_s_p_d_f_g_h_i_5_2_0_0_0_0_0_error_9.8e-07
   7
     1   0   0   1   1
      0.2170009784    1.0000000000
     2   0   0   1   1
      0.4410485534    1.0000000000
     3   0   0   1   1
      1.0542154011    1.0000000000
     4   0   0   1   1
      2.7792232468    1.0000000000
     5   0   0   1   1
      8.8765570199    1.0000000000
     6   1   1   1   1
      0.2325795252    1.0000000000
     7   1   1   1   1
```

```
      1.3163036644    1.0000000000

# RI basis set for H (all-electron) relative DI metric: 3.4e-07
H  RI_aug-SZV-MOLOPT-ae_N_RI_020_s_p_d_f_g_h_i_6_3_1_0_0_0_0_error_3.4e-07
   10
     1   0   0   1   1
      0.2350618650    1.0000000000
     2   0   0   1   1
      0.5052340022    1.0000000000
     3   0   0   1   1
      0.9936556058    1.0000000000
     4   0   0   1   1
      1.7749267927    1.0000000000
     5   0   0   1   1
      4.9213708088    1.0000000000
     6   0   0   1   1
     23.3219919414    1.0000000000
     7   1   1   1   1
      0.2635391247    1.0000000000
     8   1   1   1   1
      0.8270918706    1.0000000000
     9   1   1   1   1
      1.9826522887    1.0000000000
    10   2   2   1   1
      0.9794814801    1.0000000000

# RI basis set for H (all-electron) relative DI metric: 1.1e-07
H  RI_aug-SZV-MOLOPT-ae_N_RI_026_s_p_d_f_g_h_i_7_3_2_0_0_0_0_error_1.1e-07
   12
     1   0   0   1   1
      0.2241441298    1.0000000000
     2   0   0   1   1
      0.4188097859    1.0000000000
     3   0   0   1   1
      0.7544730669    1.0000000000
     4   0   0   1   1
      1.3556095300    1.0000000000
     5   0   0   1   1
      2.4300856355    1.0000000000
     6   0   0   1   1
      4.3510092418    1.0000000000
     7   0   0   1   1
      7.8363248142    1.0000000000
     8   1   1   1   1
      0.1298778793    1.0000000000
     9   1   1   1   1
      0.4403713398    1.0000000000
    10   1   1   1   1
      1.4931481731    1.0000000000
    11   2   2   1   1
      0.6205955943    1.0000000000
    12   2   2   1   1
      1.2340233898    1.0000000000

# RI basis set for H (all-electron) relative DI metric: 5.1e-08
H  RI_aug-SZV-MOLOPT-ae_N_RI_035_s_p_d_f_g_h_i_7_6_2_0_0_0_0_error_5.1e-08
   15
     1   0   0   1   1
      0.2369109999    1.0000000000
     2   0   0   1   1
      0.4238968856    1.0000000000
     3   0   0   1   1
      0.7584644429    1.0000000000
     4   0   0   1   1
      1.3570949225    1.0000000000
     5   0   0   1   1
      2.4282043094    1.0000000000
     6   0   0   1   1
      4.3447043169    1.0000000000
     7   0   0   1   1
      7.7738333335    1.0000000000
     8   1   1   1   1
      0.3478920000    1.0000000000
     9   1   1   1   1
      0.4931656038    1.0000000000
    10   1   1   1   1
      0.6991029192    1.0000000000
    11   1   1   1   1
      0.9910360493    1.0000000000
    12   1   1   1   1
      1.4048753396    1.0000000000
    13   1   1   1   1
      1.9915266667    1.0000000000
    14   2   2   1   1
      0.6253660000    1.0000000000
    15   2   2   1   1
      1.2860533333    1.0000000000

# RI basis set for H (all-electron) relative DI metric: 2.9e-03
H  RI_aug-DZVP-MOLOPT-ae_N_RI_005_s_p_d_f_g_h_i_2_1_0_0_0_0_0_error_2.9e-03
   3
     1   0   0   1   1
      0.6020328442    1.0000000000
     2   0   0   1   1
      2.0551121420    1.0000000000
     3   1   1   1   1
      1.1065929596    1.0000000000

# RI basis set for H (all-electron) relative DI metric: 4.4e-05
H  RI_aug-DZVP-MOLOPT-ae_N_RI_009_s_p_d_f_g_h_i_3_2_0_0_0_0_0_error_4.4e-05
   5
     1   0   0   1   1
      0.3735675702    1.0000000000
     2   0   0   1   1
      0.8864765188    1.0000000000
     3   0   0   1   1
      5.0288475332    1.0000000000
     4   1   1   1   1
      0.3303148160    1.0000000000
     5   1   1   1   1
      1.4513187711    1.0000000000
```

```
# RI basis set for H (all-electron) relative DI metric: 1.6e-05
H  RI_aug-DZVP-MOLOPT-ae_N_RI_013_s_p_d_f_g_h_i_4_3_0_0_0_0_0_error_1.6e-05
   7
     1   0   0   1   1
      0.2819663791    1.0000000000
     2   0   0   1   1
      0.6323106124    1.0000000000
     3   0   0   1   1
      1.7000274384    1.0000000000
     4   0   0   1   1
      9.3565662629    1.0000000000
     5   1   1   1   1
      0.2117538520    1.0000000000
     6   1   1   1   1
      0.6280621919    1.0000000000
     7   1   1   1   1
      1.8253246023    1.0000000000

# RI basis set for H (all-electron) relative DI metric: 6.3e-07
H  RI_aug-DZVP-MOLOPT-ae_N_RI_018_s_p_d_f_g_h_i_4_3_1_0_0_0_0_error_6.3e-07
   8
     1   0   0   1   1
      0.2427161444    1.0000000000
     2   0   0   1   1
      0.5773799365    1.0000000000
     3   0   0   1   1
      1.7983762709    1.0000000000
     4   0   0   1   1
      9.5769277682    1.0000000000
     5   1   1   1   1
      0.2318801459    1.0000000000
     6   1   1   1   1
      0.6342948358    1.0000000000
     7   1   1   1   1
      1.9313895689    1.0000000000
     8   2   2   1   1
      1.2035077028    1.0000000000

# RI basis set for H (all-electron) relative DI metric: 1.5e-07
H  RI_aug-DZVP-MOLOPT-ae_N_RI_019_s_p_d_f_g_h_i_5_3_1_0_0_0_0_error_1.5e-07
   9
     1   0   0   1   1
      0.1541784933    1.0000000000
     2   0   0   1   1
      0.4872638522    1.0000000000
     3   0   0   1   1
      1.2896085706    1.0000000000
     4   0   0   1   1
      2.7708466656    1.0000000000
     5   0   0   1   1
      9.1784327278    1.0000000000
     6   1   1   1   1
      0.2591148909    1.0000000000
     7   1   1   1   1
      0.6397858506    1.0000000000
     8   1   1   1   1
      1.9839356512    1.0000000000
     9   2   2   1   1
      1.5281545369    1.0000000000

# RI basis set for H (all-electron) relative DI metric: 6.1e-08
H  RI_aug-DZVP-MOLOPT-ae_N_RI_031_s_p_d_f_g_h_i_5_3_2_1_0_0_0_error_6.1e-08
   11
     1   0   0   1   1
      0.1629095425    1.0000000000
     2   0   0   1   1
      0.4154636073    1.0000000000
     3   0   0   1   1
      1.2377267256    1.0000000000
     4   0   0   1   1
      3.1601335660    1.0000000000
     5   0   0   1   1
      9.9061915067    1.0000000000
     6   1   1   1   1
      0.2266362479    1.0000000000
     7   1   1   1   1
      0.6650698011    1.0000000000
     8   1   1   1   1
      1.9905989356    1.0000000000
     9   2   2   1   1
      0.8366461724    1.0000000000
    10   2   2   1   1
      1.3744872974    1.0000000000
    11   3   3   1   1
      1.3312038025    1.0000000000

# RI basis set for H (all-electron) relative DI metric: 2.9e-08
H  RI_aug-DZVP-MOLOPT-ae_N_RI_047_s_p_d_f_g_h_i_7_6_3_1_0_0_0_error_2.9e-08
   17
     1   0   0   1   1
      0.2369110000    1.0000000000
     2   0   0   1   1
      0.4238968856    1.0000000000
     3   0   0   1   1
      0.7584644429    1.0000000000
     4   0   0   1   1
      1.3570949226    1.0000000000
     5   0   0   1   1
      2.4282043095    1.0000000000
     6   0   0   1   1
      4.3447043169    1.0000000000
     7   0   0   1   1
      7.7738333335    1.0000000000
     8   1   1   1   1
      0.3478920000    1.0000000000
     9   1   1   1   1
      0.4931656038    1.0000000000
    10   1   1   1   1
      0.6991029192    1.0000000000
    11   1   1   1   1
```

```
      0.9910360493    1.0000000000
    12   1   1   1   1
      1.4048753396    1.0000000000
    13   1   1   1   1
      1.9915266666    1.0000000000
    14   2   2   1   1
      0.6253660000    1.0000000000
    15   2   2   1   1
      0.8968021124    1.0000000000
    16   2   2   1   1
      1.2860533334    1.0000000000
    17   3   3   1   1
      1.1981100000    1.0000000000

# RI basis set for H (all-electron) relative DI metric: 9.0e-03
H  RI_aug-TZVP-MOLOPT-ae_N_RI_011_s_p_d_f_g_h_i_3_1_1_0_0_0_0_error_9.0e-03
   5
     1   0   0   1   1
      0.4920300024    1.0000000000
     2   0   0   1   1
      1.6049088854    1.0000000000
     3   0   0   1   1
      9.4056393035    1.0000000000
     4   1   1   1   1
      1.2220981421    1.0000000000
     5   2   2   1   1
      1.2033086289    1.0000000000

# RI basis set for H (all-electron) relative DI metric: 5.9e-04
H  RI_aug-TZVP-MOLOPT-ae_N_RI_015_s_p_d_f_g_h_i_4_2_1_0_0_0_0_error_5.9e-04
   7
     1   0   0   1   1
      0.4222122864    1.0000000000
     2   0   0   1   1
      0.9645053830    1.0000000000
     3   0   0   1   1
      3.4608046993    1.0000000000
     4   0   0   1   1
     10.6963281183    1.0000000000
     5   1   1   1   1
      0.5289683271    1.0000000000
     6   1   1   1   1
      2.1250573245    1.0000000000
     7   2   2   1   1
      1.3115890895    1.0000000000

# RI basis set for H (all-electron) relative DI metric: 1.0e-04
H  RI_aug-TZVP-MOLOPT-ae_N_RI_020_s_p_d_f_g_h_i_4_2_2_0_0_0_0_error_1.0e-04
   8
     1   0   0   1   1
      0.3227056137    1.0000000000
     2   0   0   1   1
      1.1861118630    1.0000000000
     3   0   0   1   1
      4.3595819219    1.0000000000
     4   0   0   1   1
     16.0237478615    1.0000000000
     5   1   1   1   1
      0.5037358136    1.0000000000
     6   1   1   1   1
      1.8993450245    1.0000000000
     7   2   2   1   1
      0.4561915998    1.0000000000
     8   2   2   1   1
      1.5673930803    1.0000000000

# RI basis set for H (all-electron) relative DI metric: 4.6e-05
H  RI_aug-TZVP-MOLOPT-ae_N_RI_023_s_p_d_f_g_h_i_4_3_2_0_0_0_0_error_4.6e-05
   9
     1   0   0   1   1
      0.4870324295    1.0000000000
     2   0   0   1   1
      1.7744452734    1.0000000000
     3   0   0   1   1
      6.4649822623    1.0000000000
     4   0   0   1   1
     23.5544011224    1.0000000000
     5   1   1   1   1
      0.1997035395    1.0000000000
     6   1   1   1   1
      0.5877622634    1.0000000000
     7   1   1   1   1
      2.1620970442    1.0000000000
     8   2   2   1   1
      0.3710609969    1.0000000000
     9   2   2   1   1
      1.4863697565    1.0000000000

# RI basis set for H (all-electron) relative DI metric: 4.5e-06
H  RI_aug-TZVP-MOLOPT-ae_N_RI_031_s_p_d_f_g_h_i_6_5_2_0_0_0_0_error_4.5e-06
   13
     1   0   0   1   1
      0.2249316285    1.0000000000
     2   0   0   1   1
      0.4979081683    1.0000000000
     3   0   0   1   1
      0.9872873263    1.0000000000
     4   0   0   1   1
      1.7846470216    1.0000000000
     5   0   0   1   1
      5.1187494220    1.0000000000
     6   0   0   1   1
     26.8039600727    1.0000000000
     7   1   1   1   1
      0.2794776723    1.0000000000
     8   1   1   1   1
      0.5310113443    1.0000000000
     9   1   1   1   1
      1.5185390663    1.0000000000
    10   1   1   1   1
      6.8263430324    1.0000000000
```

```
    11   1   1   1   1
     18.2662499465    1.0000000000
    12   2   2   1   1
      0.4693442359    1.0000000000
    13   2   2   1   1
      1.4730352119    1.0000000000

# RI basis set for He (all-electron) relative DI metric: 4.2e-03
He  RI_aug-SZV-MOLOPT-ae_N_RI_003_s_p_d_f_g_h_i_3_0_0_0_0_0_0_error_4.2e-03
   3
     1   0   0   1   1
      0.2928573659    1.0000000000
     2   0   0   1   1
      2.6608694544    1.0000000000
     3   0   0   1   1
     11.9714078591    1.0000000000

# RI basis set for He (all-electron) relative DI metric: 2.5e-06
He  RI_aug-SZV-MOLOPT-ae_N_RI_009_s_p_d_f_g_h_i_6_1_0_0_0_0_0_error_2.5e-06
   7
     1   0   0   1   1
      0.2767048707    1.0000000000
     2   0   0   1   1
      0.8362575869    1.0000000000
     3   0   0   1   1
      2.0077680651    1.0000000000
     4   0   0   1   1
      4.9747970507    1.0000000000
     5   0   0   1   1
     15.6006625991    1.0000000000
     6   0   0   1   1
     69.8876707484    1.0000000000
     7   1   1   1   1
      0.3001402433    1.0000000000

# RI basis set for He (all-electron) relative DI metric: 7.2e-07
He  RI_aug-SZV-MOLOPT-ae_N_RI_018_s_p_d_f_g_h_i_6_4_0_0_0_0_0_error_7.2e-07
   10
     1   0   0   1   1
      0.3281284314    1.0000000000
     2   0   0   1   1
      0.8455612806    1.0000000000
     3   0   0   1   1
      2.0026886479    1.0000000000
     4   0   0   1   1
      5.0111238866    1.0000000000
     5   0   0   1   1
     15.5809589082    1.0000000000
     6   0   0   1   1
     69.9393812976    1.0000000000
     7   1   1   1   1
      0.3663977236    1.0000000000
     8   1   1   1   1
      0.8930771227    1.0000000000
     9   1   1   1   1
      1.9514158980    1.0000000000
    10   1   1   1   1
      4.1899471264    1.0000000000

# RI basis set for He (all-electron) relative DI metric: 1.4e-07
He  RI_aug-SZV-MOLOPT-ae_N_RI_031_s_p_d_f_g_h_i_7_4_1_1_0_0_0_error_1.4e-07
   13
     1   0   0   1   1
      0.3443410323    1.0000000000
     2   0   0   1   1
      0.7411806536    1.0000000000
     3   0   0   1   1
      1.4800690631    1.0000000000
     4   0   0   1   1
      2.9448035261    1.0000000000
     5   0   0   1   1
      5.8770659493    1.0000000000
     6   0   0   1   1
     11.7021618371    1.0000000000
     7   0   0   1   1
     23.3290181415    1.0000000000
     8   1   1   1   1
      0.3187967579    1.0000000000
     9   1   1   1   1
      0.7176523604    1.0000000000
    10   1   1   1   1
      1.6155274402    1.0000000000
    11   1   1   1   1
      3.6367593196    1.0000000000
    12   2   2   1   1
      3.5200600000    1.0000000000
    13   3   3   1   1
      5.4484895350    1.0000000000

# RI basis set for He (all-electron) relative DI metric: 8.4e-03
He  RI_aug-DZVP-MOLOPT-ae_N_RI_005_s_p_d_f_g_h_i_2_1_0_0_0_0_0_error_8.4e-03
   3
     1   0   0   1   1
      0.5800765063    1.0000000000
     2   0   0   1   1
      4.9791481247    1.0000000000
     3   1   1   1   1
      1.5526672041    1.0000000000

# RI basis set for He (all-electron) relative DI metric: 1.8e-04
He  RI_aug-DZVP-MOLOPT-ae_N_RI_011_s_p_d_f_g_h_i_3_1_1_0_0_0_0_error_1.8e-04
   5
     1   0   0   1   1
      0.5282943733    1.0000000000
     2   0   0   1   1
      2.8184144556    1.0000000000
     3   0   0   1   1
     22.4734319396    1.0000000000
     4   1   1   1   1
      1.5711890881    1.0000000000
     5   2   2   1   1
```

```
      0.8890133475    1.0000000000

# RI basis set for He (all-electron) relative DI metric: 4.5e-05
He  RI_aug-DZVP-MOLOPT-ae_N_RI_014_s_p_d_f_g_h_i_3_2_1_0_0_0_0_error_4.5e-05
   6
     1   0   0   1   1
      0.6195053070    1.0000000000
     2   0   0   1   1
      1.7594147045    1.0000000000
     3   0   0   1   1
     12.2639524681    1.0000000000
     4   1   1   1   1
      0.8901940527    1.0000000000
     5   1   1   1   1
      2.5082462491    1.0000000000
     6   2   2   1   1
      0.9644685161    1.0000000000

# RI basis set for He (all-electron) relative DI metric: 9.7e-06
He  RI_aug-DZVP-MOLOPT-ae_N_RI_018_s_p_d_f_g_h_i_4_3_1_0_0_0_0_error_9.7e-06
   8
     1   0   0   1   1
      0.2638845757    1.0000000000
     2   0   0   1   1
      0.5413964588    1.0000000000
     3   0   0   1   1
      3.3104898593    1.0000000000
     4   0   0   1   1
     21.3700049021    1.0000000000
     5   1   1   1   1
      0.4792470960    1.0000000000
     6   1   1   1   1
      1.4306470355    1.0000000000
     7   1   1   1   1
      6.1955237895    1.0000000000
     8   2   2   1   1
      0.8998154095    1.0000000000

# RI basis set for He (all-electron) relative DI metric: 1.3e-06
He  RI_aug-DZVP-MOLOPT-ae_N_RI_024_s_p_d_f_g_h_i_5_3_2_0_0_0_0_error_1.3e-06
   10
     1   0   0   1   1
      0.4365368485    1.0000000000
     2   0   0   1   1
      0.9183589121    1.0000000000
     3   0   0   1   1
      2.7589628897    1.0000000000
     4   0   0   1   1
      8.2365058627    1.0000000000
     5   0   0   1   1
     25.0808309776    1.0000000000
     6   1   1   1   1
      0.3914492770    1.0000000000
     7   1   1   1   1
      1.4970236906    1.0000000000
     8   1   1   1   1
     10.6487657292    1.0000000000
     9   2   2   1   1
      0.6531873035    1.0000000000
    10   2   2   1   1
      2.9127472641    1.0000000000

# RI basis set for He (all-electron) relative DI metric: 2.2e-07
He  RI_aug-DZVP-MOLOPT-ae_N_RI_031_s_p_d_f_g_h_i_6_5_2_0_0_0_0_error_2.2e-07
   13
     1   0   0   1   1
      0.3916064848    1.0000000000
     2   0   0   1   1
      0.7983076335    1.0000000000
     3   0   0   1   1
      1.9161374414    1.0000000000
     4   0   0   1   1
      5.1148113183    1.0000000000
     5   0   0   1   1
     15.4457405077    1.0000000000
     6   0   0   1   1
     69.2530691074    1.0000000000
     7   1   1   1   1
      0.4225747359    1.0000000000
     8   1   1   1   1
      1.1801707253    1.0000000000
     9   1   1   1   1
      3.0262184761    1.0000000000
    10   1   1   1   1
      7.3548905738    1.0000000000
    11   1   1   1   1
     21.6252263653    1.0000000000
    12   2   2   1   1
      0.6824457002    1.0000000000
    13   2   2   1   1
      2.6660108059    1.0000000000

# RI basis set for He (all-electron) relative DI metric: 5.9e-08
He  RI_aug-DZVP-MOLOPT-ae_N_RI_036_s_p_d_f_g_h_i_6_5_3_0_0_0_0_error_5.9e-08
   14
     1   0   0   1   1
      0.3897135965    1.0000000000
     2   0   0   1   1
      0.7778372153    1.0000000000
     3   0   0   1   1
      1.8828863133    1.0000000000
     4   0   0   1   1
      5.1492641924    1.0000000000
     5   0   0   1   1
     15.4159046642    1.0000000000
     6   0   0   1   1
     68.7694127854    1.0000000000
     7   1   1   1   1
      0.4275319008    1.0000000000
     8   1   1   1   1
      1.1759845528    1.0000000000
```

```
     9   1   1   1   1
      3.0069168037    1.0000000000
    10   1   1   1   1
      7.3081681638    1.0000000000
    11   1   1   1   1
     21.3050249152    1.0000000000
    12   2   2   1   1
      0.5049976236    1.0000000000
    13   2   2   1   1
      1.5193023142    1.0000000000
    14   2   2   1   1
      3.5925551628    1.0000000000

# RI basis set for He (all-electron) relative DI metric: 3.7e-02
He  RI_aug-TZVP-MOLOPT-ae_N_RI_011_s_p_d_f_g_h_i_3_1_1_0_0_0_0_error_3.7e-02
   5
     1   0   0   1   1
      0.4580211668    1.0000000000
     2   0   0   1   1
      1.6811714090    1.0000000000
     3   0   0   1   1
     13.0932111484    1.0000000000
     4   1   1   1   1
      2.4213666598    1.0000000000
     5   2   2   1   1
      2.8667761206    1.0000000000

# RI basis set for He (all-electron) relative DI metric: 1.7e-03
He  RI_aug-TZVP-MOLOPT-ae_N_RI_014_s_p_d_f_g_h_i_3_2_1_0_0_0_0_error_1.7e-03
   6
     1   0   0   1   1
      0.4643512619    1.0000000000
     2   0   0   1   1
      1.7719289689    1.0000000000
     3   0   0   1   1
     12.7109646723    1.0000000000
     4   1   1   1   1
      1.0729188442    1.0000000000
     5   1   1   1   1
      3.7462593856    1.0000000000
     6   2   2   1   1
      2.8517757422    1.0000000000

# RI basis set for He (all-electron) relative DI metric: 4.3e-04
He  RI_aug-TZVP-MOLOPT-ae_N_RI_021_s_p_d_f_g_h_i_3_2_1_1_0_0_0_error_4.3e-04
   7
     1   0   0   1   1
      0.4472018813    1.0000000000
     2   0   0   1   1
      1.9938195056    1.0000000000
     3   0   0   1   1
     15.3573344407    1.0000000000
     4   1   1   1   1
      1.0669120878    1.0000000000
     5   1   1   1   1
      3.7064460128    1.0000000000
     6   2   2   1   1
      2.8788531349    1.0000000000
     7   3   3   1   1
      1.1880376992    1.0000000000

# RI basis set for He (all-electron) relative DI metric: 3.9e-05
He  RI_aug-TZVP-MOLOPT-ae_N_RI_025_s_p_d_f_g_h_i_4_3_1_1_0_0_0_error_3.9e-05
   9
     1   0   0   1   1
      0.4872773998    1.0000000000
     2   0   0   1   1
      1.2585594729    1.0000000000
     3   0   0   1   1
      4.8845478311    1.0000000000
     4   0   0   1   1
     21.1818885779    1.0000000000
     5   1   1   1   1
      0.4414845041    1.0000000000
     6   1   1   1   1
      1.2626597190    1.0000000000
     7   1   1   1   1
      4.0152980628    1.0000000000
     8   2   2   1   1
      2.8684021807    1.0000000000
     9   3   3   1   1
      0.8764308672    1.0000000000

# RI basis set for He (all-electron) relative DI metric: 3.9e-06
He  RI_aug-TZVP-MOLOPT-ae_N_RI_030_s_p_d_f_g_h_i_4_3_2_1_0_0_0_error_3.9e-06
   10
     1   0   0   1   1
      0.4836629894    1.0000000000
     2   0   0   1   1
      0.9303353671    1.0000000000
     3   0   0   1   1
      2.5301957601    1.0000000000
     4   0   0   1   1
     17.5846943901    1.0000000000
     5   1   1   1   1
      0.4944364031    1.0000000000
     6   1   1   1   1
      1.1188370757    1.0000000000
     7   1   1   1   1
      4.0527542391    1.0000000000
     8   2   2   1   1
      1.3294446263    1.0000000000
     9   2   2   1   1
      3.6676568402    1.0000000000
    10   3   3   1   1
      0.9253742267    1.0000000000

# RI basis set for He (all-electron) relative DI metric: 8.1e-07
He  RI_aug-TZVP-MOLOPT-ae_N_RI_038_s_p_d_f_g_h_i_5_3_2_2_0_0_0_error_8.1e-07
   12
     1   0   0   1   1
```

```
      0.4189913763    1.0000000000
     2   0   0   1   1
      0.9059352217    1.0000000000
     3   0   0   1   1
      2.5115961909    1.0000000000
     4   0   0   1   1
      8.0063470036    1.0000000000
     5   0   0   1   1
     23.9720187989    1.0000000000
     6   1   1   1   1
      0.3956710565    1.0000000000
     7   1   1   1   1
      1.1755188147    1.0000000000
     8   1   1   1   1
      4.0218764859    1.0000000000
     9   2   2   1   1
      1.3294769183    1.0000000000
    10   2   2   1   1
      3.6681727074    1.0000000000
    11   3   3   1   1
      0.6094842030    1.0000000000
    12   3   3   1   1
      1.9854582875    1.0000000000

# RI basis set for He (all-electron) relative DI metric: 1.7e-07
He  RI_aug-TZVP-MOLOPT-ae_N_RI_042_s_p_d_f_g_h_i_6_4_2_2_0_0_0_error_1.7e-07
   14
     1   0   0   1   1
      0.2924788290    1.0000000000
     2   0   0   1   1
      0.6876110148    1.0000000000
     3   0   0   1   1
      1.6523223972    1.0000000000
     4   0   0   1   1
      5.0465646470    1.0000000000
     5   0   0   1   1
     15.5242006611    1.0000000000
     6   0   0   1   1
     69.4810839591    1.0000000000
     7   1   1   1   1
      0.3811631732    1.0000000000
     8   1   1   1   1
      0.9449083361    1.0000000000
     9   1   1   1   1
      1.9428203927    1.0000000000
    10   1   1   1   1
      4.6179679381    1.0000000000
    11   2   2   1   1
      1.2924316314    1.0000000000
    12   2   2   1   1
      3.6667957210    1.0000000000
    13   3   3   1   1
      0.5468297685    1.0000000000
    14   3   3   1   1
      1.4760179520    1.0000000000

# RI basis set for Li (all-electron) relative DI metric: 4.7e-04
Li  RI_aug-SZV-MOLOPT-ae-mini_N_RI_009_s_p_d_f_g_h_i_3_2_0_0_0_0_0_error_4.7e-04
   5
     1   0   0   1   1
      0.3496297613    1.0000000000
     2   0   0   1   1
      2.3116122905    1.0000000000
     3   0   0   1   1
     15.3170386416    1.0000000000
     4   1   1   1   1
      0.1457880413    1.0000000000
     5   1   1   1   1
      1.4263325185    1.0000000000

# RI basis set for Li (all-electron) relative DI metric: 9.8e-05
Li  RI_aug-SZV-MOLOPT-ae-mini_N_RI_012_s_p_d_f_g_h_i_6_2_0_0_0_0_0_error_9.8e-05
   8
     1   0   0   1   1
      0.1266926627    1.0000000000
     2   0   0   1   1
      0.3848526156    1.0000000000
     3   0   0   1   1
      0.9557526311    1.0000000000
     4   0   0   1   1
      2.5299606082    1.0000000000
     5   0   0   1   1
      8.5663489658    1.0000000000
     6   0   0   1   1
     27.1841685368    1.0000000000
     7   1   1   1   1
      0.1587638527    1.0000000000
     8   1   1   1   1
      1.4399827773    1.0000000000

# RI basis set for Li (all-electron) relative DI metric: 9.2e-07
Li  RI_aug-SZV-MOLOPT-ae-mini_N_RI_015_s_p_d_f_g_h_i_6_3_0_0_0_0_0_error_9.2e-07
   9
     1   0   0   1   1
      0.1245879747    1.0000000000
     2   0   0   1   1
      0.3786907139    1.0000000000
     3   0   0   1   1
      0.9664745050    1.0000000000
     4   0   0   1   1
      2.6282373054    1.0000000000
     5   0   0   1   1
      8.7516610522    1.0000000000
     6   0   0   1   1
     27.0343284342    1.0000000000
     7   1   1   1   1
      0.1175657934    1.0000000000
     8   1   1   1   1
      0.9935990820    1.0000000000
     9   1   1   1   1
      4.0128832489    1.0000000000
```

```
# RI basis set for Li (all-electron) relative DI metric: 4.8e-02
Li  RI_aug-SZV-MOLOPT-ae_N_RI_005_s_p_d_f_g_h_i_2_1_0_0_0_0_0_error_4.8e-02
   3
    1   0   0   1   1
      6.2084121040    1.0000000000
    2   0   0   1   1
     22.4170259204    1.0000000000
    3   1   1   1   1
      1.3737602394    1.0000000000

# RI basis set for Li (all-electron) relative DI metric: 1.0e-02
Li  RI_aug-SZV-MOLOPT-ae_N_RI_006_s_p_d_f_g_h_i_3_1_0_0_0_0_0_error_1.0e-02
   4
    1   0   0   1   1
      0.5918107477    1.0000000000
    2   0   0   1   1
      5.8214114237    1.0000000000
    3   0   0   1   1
     23.4647171688    1.0000000000
    4   1   1   1   1
      1.3997738808    1.0000000000

# RI basis set for Li (all-electron) relative DI metric: 3.8e-03
Li  RI_aug-SZV-MOLOPT-ae_N_RI_011_s_p_d_f_g_h_i_3_1_1_0_0_0_0_error_3.8e-03
   5
    1   0   0   1   1
      0.5505135941    1.0000000000
    2   0   0   1   1
      7.9839775055    1.0000000000
    3   0   0   1   1
     23.4943215127    1.0000000000
    4   1   1   1   1
      1.3745763138    1.0000000000
    5   2   2   1   1
      0.7672789281    1.0000000000

# RI basis set for Li (all-electron) relative DI metric: 1.1e-04
Li  RI_aug-SZV-MOLOPT-ae_N_RI_014_s_p_d_f_g_h_i_3_2_1_0_0_0_0_error_1.1e-04
   6
    1   0   0   1   1
      0.5137523807    1.0000000000
    2   0   0   1   1
      8.3363890613    1.0000000000
    3   0   0   1   1
     18.2925532795    1.0000000000
    4   1   1   1   1
      0.3242299383    1.0000000000
    5   1   1   1   1
      1.6429407750    1.0000000000
    6   2   2   1   1
      0.7673096620    1.0000000000

# RI basis set for Li (all-electron) relative DI metric: 2.2e-05
Li  RI_aug-SZV-MOLOPT-ae_N_RI_018_s_p_d_f_g_h_i_4_3_1_0_0_0_0_error_2.2e-05
   8
    1   0   0   1   1
      0.2536890794    1.0000000000
    2   0   0   1   1
      0.7427866114    1.0000000000
    3   0   0   1   1
      6.1836155034    1.0000000000
    4   0   0   1   1
     31.3087310167    1.0000000000
    5   1   1   1   1
      0.2574008809    1.0000000000
    6   1   1   1   1
      1.0838890909    1.0000000000
    7   1   1   1   1
      4.4515528124    1.0000000000
    8   2   2   1   1
      0.7686702585    1.0000000000

# RI basis set for Li (all-electron) relative DI metric: 3.5e-06
Li  RI_aug-SZV-MOLOPT-ae_N_RI_023_s_p_d_f_g_h_i_4_3_2_0_0_0_0_error_3.5e-06
   9
    1   0   0   1   1
      0.2660733242    1.0000000000
    2   0   0   1   1
      0.7440750558    1.0000000000
    3   0   0   1   1
      6.1921754131    1.0000000000
    4   0   0   1   1
     31.7801278422    1.0000000000
    5   1   1   1   1
      0.2473099670    1.0000000000
    6   1   1   1   1
      1.0016277195    1.0000000000
    7   1   1   1   1
      3.8463034719    1.0000000000
    8   2   2   1   1
      0.4581833795    1.0000000000
    9   2   2   1   1
      2.5158456830    1.0000000000

# RI basis set for Li (all-electron) relative DI metric: 8.0e-07
Li  RI_aug-SZV-MOLOPT-ae_N_RI_028_s_p_d_f_g_h_i_4_3_3_0_0_0_0_error_8.0e-07
   10
    1   0   0   1   1
      0.3245560788    1.0000000000
    2   0   0   1   1
      1.1372932793    1.0000000000
    3   0   0   1   1
      5.4284432155    1.0000000000
    4   0   0   1   1
     30.5874725057    1.0000000000
    5   1   1   1   1
      0.2529770330    1.0000000000
    6   1   1   1   1
      1.0620002655    1.0000000000
    7   1   1   1   1
```

```
      4.3266577118    1.0000000000
     8   2   2   1   1
      0.1092880641    1.0000000000
     9   2   2   1   1
      0.4801563819    1.0000000000
    10   2   2   1   1
      2.3378216974    1.0000000000

# RI basis set for Li (all-electron) relative DI metric: 3.9e-07
Li  RI_aug-SZV-MOLOPT-ae_N_RI_104_s_p_d_f_g_h_i_5_4_4_2_2_2_1_error_3.9e-07
   20
     1   0   0   1   1
      0.2370942763    1.0000000000
     2   0   0   1   1
      0.9205269073    1.0000000000
     3   0   0   1   1
      2.6339689852    1.0000000000
     4   0   0   1   1
      8.4191419856    1.0000000000
     5   0   0   1   1
     37.6123928513    1.0000000000
     6   1   1   1   1
      0.1306881328    1.0000000000
     7   1   1   1   1
      0.3415343691    1.0000000000
     8   1   1   1   1
      1.3350258999    1.0000000000
     9   1   1   1   1
      6.4906102699    1.0000000000
    10   2   2   1   1
      0.1007081297    1.0000000000
    11   2   2   1   1
      0.2874290819    1.0000000000
    12   2   2   1   1
      0.8160639196    1.0000000000
    13   2   2   1   1
      2.3286914870    1.0000000000
    14   3   3   1   1
      0.1670531148    1.0000000000
    15   3   3   1   1
      1.0729094546    1.0000000000
    16   4   4   1   1
      0.1112100753    1.0000000000
    17   4   4   1   1
      0.4536139815    1.0000000000
    18   5   5   1   1
      0.3952389732    1.0000000000
    19   5   5   1   1
      0.4272495728    1.0000000000
    20   6   6   1   1
      0.7349241171    1.0000000000

# RI basis set for Li (all-electron) relative DI metric: 1.1e-02
Li  RI_aug-DZVP-MOLOPT-ae_N_RI_006_s_p_d_f_g_h_i_3_1_0_0_0_0_0_error_1.1e-02
   4
     1   0   0   1   1
      2.4916607417    1.0000000000
     2   0   0   1   1
      9.3921918903    1.0000000000
     3   0   0   1   1
     31.3463671159    1.0000000000
     4   1   1   1   1
      1.7928072978    1.0000000000

# RI basis set for Li (all-electron) relative DI metric: 3.5e-03
Li  RI_aug-DZVP-MOLOPT-ae_N_RI_009_s_p_d_f_g_h_i_3_2_0_0_0_0_0_error_3.5e-03
   5
     1   0   0   1   1
      1.4390401105    1.0000000000
     2   0   0   1   1
     12.0535427197    1.0000000000
     3   0   0   1   1
     50.8623685541    1.0000000000
     4   1   1   1   1
      0.7706698602    1.0000000000
     5   1   1   1   1
      2.7844011814    1.0000000000

# RI basis set for Li (all-electron) relative DI metric: 1.2e-03
Li  RI_aug-DZVP-MOLOPT-ae_N_RI_010_s_p_d_f_g_h_i_4_2_0_0_0_0_0_error_1.2e-03
   6
     1   0   0   1   1
      0.3684678753    1.0000000000
     2   0   0   1   1
      2.4959284032    1.0000000000
     3   0   0   1   1
     11.3425295715    1.0000000000
     4   0   0   1   1
     37.2086870325    1.0000000000
     5   1   1   1   1
      0.7146750996    1.0000000000
     6   1   1   1   1
      2.6158194473    1.0000000000

# RI basis set for Li (all-electron) relative DI metric: 2.7e-04
Li  RI_aug-DZVP-MOLOPT-ae_N_RI_015_s_p_d_f_g_h_i_4_2_1_0_0_0_0_error_2.7e-04
   7
     1   0   0   1   1
      0.3084103587    1.0000000000
     2   0   0   1   1
      2.2523286129    1.0000000000
     3   0   0   1   1
     13.4336085244    1.0000000000
     4   0   0   1   1
     53.2740420415    1.0000000000
     5   1   1   1   1
      0.8863937322    1.0000000000
     6   1   1   1   1
      3.0735731667    1.0000000000
     7   2   2   1   1
      0.7742805827    1.0000000000
```

```
# RI basis set for Li (all-electron) relative DI metric: 1.1e-05
Li  RI_aug-DZVP-MOLOPT-ae_N_RI_018_s_p_d_f_g_h_i_4_3_1_0_0_0_0_error_1.1e-05
   8
    1   0   0   1   1
      0.6182489890    1.0000000000
    2   0   0   1   1
      3.0921771346    1.0000000000
    3   0   0   1   1
     11.7413733387    1.0000000000
    4   0   0   1   1
     31.8419081199    1.0000000000
    5   1   1   1   1
      0.4913032870    1.0000000000
    6   1   1   1   1
      0.8580572738    1.0000000000
    7   1   1   1   1
      3.1346871993    1.0000000000
    8   2   2   1   1
      0.9071235587    1.0000000000

# RI basis set for Li (all-electron) relative DI metric: 4.6e-06
Li  RI_aug-DZVP-MOLOPT-ae_N_RI_022_s_p_d_f_g_h_i_5_4_1_0_0_0_0_error_4.6e-06
   10
    1   0   0   1   1
      0.1004011863    1.0000000000
    2   0   0   1   1
      0.6182046384    1.0000000000
    3   0   0   1   1
      3.1641938056    1.0000000000
    4   0   0   1   1
     11.4580289039    1.0000000000
    5   0   0   1   1
     32.3111193567    1.0000000000
    6   1   1   1   1
      0.1034701188    1.0000000000
    7   1   1   1   1
      0.4363539797    1.0000000000
    8   1   1   1   1
      1.3001591911    1.0000000000
    9   1   1   1   1
      4.4487690422    1.0000000000
   10   2   2   1   1
      0.9121088287    1.0000000000

# RI basis set for Li (all-electron) relative DI metric: 1.0e-06
Li  RI_aug-DZVP-MOLOPT-ae_N_RI_080_s_p_d_f_g_h_i_5_4_3_3_3_0_0_error_1.0e-06
   18
    1   0   0   1   1
      0.1621932177    1.0000000000
    2   0   0   1   1
      0.6559690291    1.0000000000
    3   0   0   1   1
      2.8945204305    1.0000000000
    4   0   0   1   1
     11.0162110986    1.0000000000
    5   0   0   1   1
     33.1927957253    1.0000000000
    6   1   1   1   1
      0.1812113386    1.0000000000
    7   1   1   1   1
      0.5691845889    1.0000000000
    8   1   1   1   1
      1.4299923686    1.0000000000
    9   1   1   1   1
      4.7437927480    1.0000000000
   10   2   2   1   1
      0.1572603012    1.0000000000
   11   2   2   1   1
      0.4926538517    1.0000000000
   12   2   2   1   1
      2.2582517084    1.0000000000
   13   3   3   1   1
      0.1146274170    1.0000000000
   14   3   3   1   1
      0.3780434270    1.0000000000
   15   3   3   1   1
      0.9673984334    1.0000000000
   16   4   4   1   1
      0.1625425774    1.0000000000
   17   4   4   1   1
      0.3465576506    1.0000000000
   18   4   4   1   1
      0.6179790344    1.0000000000

# RI basis set for Li (all-electron) relative DI metric: 1.5e-02
Li  RI_aug-TZVP-MOLOPT-ae_N_RI_006_s_p_d_f_g_h_i_3_1_0_0_0_0_0_error_1.5e-02
   4
    1   0   0   1   1
      1.2418833637    1.0000000000
    2   0   0   1   1
      4.3156365677    1.0000000000
    3   0   0   1   1
     15.1652330873    1.0000000000
    4   1   1   1   1
      3.9377584450    1.0000000000

# RI basis set for Li (all-electron) relative DI metric: 1.8e-03
Li  RI_aug-TZVP-MOLOPT-ae_N_RI_010_s_p_d_f_g_h_i_4_2_0_0_0_0_0_error_1.8e-03
   6
    1   0   0   1   1
      0.5479199871    1.0000000000
    2   0   0   1   1
      1.7531264214    1.0000000000
    3   0   0   1   1
      4.0967941017    1.0000000000
    4   0   0   1   1
     16.3127939881    1.0000000000
    5   1   1   1   1
      1.1101786392    1.0000000000
    6   1   1   1   1
```

```
      4.5563339803    1.0000000000

# RI basis set for Li (all-electron) relative DI metric: 9.9e-05
Li  RI_aug-TZVP-MOLOPT-ae_N_RI_015_s_p_d_f_g_h_i_4_2_1_0_0_0_0_error_9.9e-05
   7
     1   0   0   1   1
      0.4235919563    1.0000000000
     2   0   0   1   1
      1.4926835069    1.0000000000
     3   0   0   1   1
      5.2600218427    1.0000000000
     4   0   0   1   1
     18.5356440205    1.0000000000
     5   1   1   1   1
      1.0956527484    1.0000000000
     6   1   1   1   1
      4.6029400974    1.0000000000
     7   2   2   1   1
      0.9143737140    1.0000000000

# RI basis set for Li (all-electron) relative DI metric: 2.0e-05
Li  RI_aug-TZVP-MOLOPT-ae_N_RI_018_s_p_d_f_g_h_i_4_3_1_0_0_0_0_error_2.0e-05
   8
     1   0   0   1   1
      0.5173015905    1.0000000000
     2   0   0   1   1
      1.6827082266    1.0000000000
     3   0   0   1   1
      4.3378108034    1.0000000000
     4   0   0   1   1
     16.4696466889    1.0000000000
     5   1   1   1   1
      0.4179946941    1.0000000000
     6   1   1   1   1
      1.3153495542    1.0000000000
     7   1   1   1   1
      4.4216545744    1.0000000000
     8   2   2   1   1
      0.9125372407    1.0000000000

# RI basis set for Li (all-electron) relative DI metric: 9.2e-06
Li  RI_aug-TZVP-MOLOPT-ae_N_RI_020_s_p_d_f_g_h_i_6_3_1_0_0_0_0_error_9.2e-06
   10
     1   0   0   1   1
      0.1437569362    1.0000000000
     2   0   0   1   1
      0.4550956506    1.0000000000
     3   0   0   1   1
      1.5408238918    1.0000000000
     4   0   0   1   1
      3.6081169910    1.0000000000
     5   0   0   1   1
      7.5386485970    1.0000000000
     6   0   0   1   1
     29.6707035855    1.0000000000
     7   1   1   1   1
      0.4857784130    1.0000000000
     8   1   1   1   1
      1.4003979068    1.0000000000
     9   1   1   1   1
      4.2695109787    1.0000000000
    10   2   2   1   1
      0.9338389900    1.0000000000

# RI basis set for Li (all-electron) relative DI metric: 4.5e-06
Li  RI_aug-TZVP-MOLOPT-ae_N_RI_027_s_p_d_f_g_h_i_7_5_1_0_0_0_0_error_4.5e-06
   13
     1   0   0   1   1
      0.0939884541    1.0000000000
     2   0   0   1   1
      0.2018873357    1.0000000000
     3   0   0   1   1
      0.6208393015    1.0000000000
     4   0   0   1   1
      1.8400036469    1.0000000000
     5   0   0   1   1
      3.5716956538    1.0000000000
     6   0   0   1   1
      9.7900971608    1.0000000000
     7   0   0   1   1
     43.8134113976    1.0000000000
     8   1   1   1   1
      0.1282589900    1.0000000000
     9   1   1   1   1
      0.3270046536    1.0000000000
    10   1   1   1   1
      0.7810135491    1.0000000000
    11   1   1   1   1
      1.9758292575    1.0000000000
    12   1   1   1   1
      4.5275082551    1.0000000000
    13   2   2   1   1
      0.9131331057    1.0000000000

# RI basis set for Li (all-electron) relative DI metric: 6.6e-07
Li  RI_aug-TZVP-MOLOPT-ae_N_RI_032_s_p_d_f_g_h_i_7_5_2_0_0_0_0_error_6.6e-07
   14
     1   0   0   1   1
      0.0974841892    1.0000000000
     2   0   0   1   1
      0.2041084070    1.0000000000
     3   0   0   1   1
      0.6251476931    1.0000000000
     4   0   0   1   1
      1.8887974908    1.0000000000
     5   0   0   1   1
      3.4717874960    1.0000000000
     6   0   0   1   1
      9.7098594162    1.0000000000
     7   0   0   1   1
     43.2612865518    1.0000000000
```

```
     8   1   1   1   1
      0.1273369045    1.0000000000
     9   1   1   1   1
      0.3244017243    1.0000000000
    10   1   1   1   1
      0.7831570468    1.0000000000
    11   1   1   1   1
      1.9723558204    1.0000000000
    12   1   1   1   1
      4.5271833522    1.0000000000
    13   2   2   1   1
      0.6800528315    1.0000000000
    14   2   2   1   1
      1.3997999782    1.0000000000

# RI basis set for Li (all-electron) relative DI metric: 3.2e-07
Li  RI_aug-TZVP-MOLOPT-ae_N_RI_056_s_p_d_f_g_h_i_7_5_4_2_0_0_0_error_3.2e-07
   18
     1   0   0   1   1
      0.0981591594    1.0000000000
     2   0   0   1   1
      0.2048827613    1.0000000000
     3   0   0   1   1
      0.6270122905    1.0000000000
     4   0   0   1   1
      1.8443128321    1.0000000000
     5   0   0   1   1
      3.4690352943    1.0000000000
     6   0   0   1   1
      9.7063404448    1.0000000000
     7   0   0   1   1
     43.9353123774    1.0000000000
     8   1   1   1   1
      0.1236639637    1.0000000000
     9   1   1   1   1
      0.3224179706    1.0000000000
    10   1   1   1   1
      0.7728976675    1.0000000000
    11   1   1   1   1
      1.9552599060    1.0000000000
    12   1   1   1   1
      4.5461526000    1.0000000000
    13   2   2   1   1
      0.1131038401    1.0000000000
    14   2   2   1   1
      0.2895785960    1.0000000000
    15   2   2   1   1
      0.8355139674    1.0000000000
    16   2   2   1   1
      2.2882230273    1.0000000000
    17   3   3   1   1
      0.1336070947    1.0000000000
    18   3   3   1   1
      1.0835696307    1.0000000000

# RI basis set for Be (all-electron) relative DI metric: 1.3e-02
Be  RI_aug-SZV-MOLOPT-ae-mini_N_RI_009_s_p_d_f_g_h_i_3_2_0_0_0_0_0_error_1.3e-02
   5
     1   0   0   1   1
      0.7764010800    1.0000000000
     2   0   0   1   1
      7.1505325961    1.0000000000
     3   0   0   1   1
     72.3147648020    1.0000000000
     4   1   1   1   1
      0.2770890543    1.0000000000
     5   1   1   1   1
      0.4903269990    1.0000000000

# RI basis set for Be (all-electron) relative DI metric: 5.2e-06
Be  RI_aug-SZV-MOLOPT-ae-mini_N_RI_016_s_p_d_f_g_h_i_7_3_0_0_0_0_0_error_5.2e-06
   10
     1   0   0   1   1
      0.1096822461    1.0000000000
     2   0   0   1   1
      0.3146507837    1.0000000000
     3   0   0   1   1
      0.9849871068    1.0000000000
     4   0   0   1   1
      2.7089836765    1.0000000000
     5   0   0   1   1
      7.1980201003    1.0000000000
     6   0   0   1   1
     19.0078189412    1.0000000000
     7   0   0   1   1
     50.2591771488    1.0000000000
     8   1   1   1   1
      0.1552253143    1.0000000000
     9   1   1   1   1
      0.4826696273    1.0000000000
    10   1   1   1   1
      3.3446943618    1.0000000000

# RI basis set for Be (all-electron) relative DI metric: 4.2e-02
Be  RI_aug-SZV-MOLOPT-ae_N_RI_010_s_p_d_f_g_h_i_2_1_1_0_0_0_0_error_4.2e-02
   4
     1   0   0   1   1
      1.0969320829    1.0000000000
     2   0   0   1   1
     12.7765171623    1.0000000000
     3   1   1   1   1
      0.2887799304    1.0000000000
     4   2   2   1   1
      0.3346038588    1.0000000000

# RI basis set for Be (all-electron) relative DI metric: 1.2e-02
Be  RI_aug-SZV-MOLOPT-ae_N_RI_020_s_p_d_f_g_h_i_5_1_1_1_0_0_0_error_1.2e-02
   8
     1   0   0   1   1
      0.1162739708    1.0000000000
     2   0   0   1   1
```

```
      0.3336047865    1.0000000000
     3   0   0   1   1
      1.9546901430    1.0000000000
     4   0   0   1   1
     10.2644204478    1.0000000000
     5   0   0   1   1
     52.0670897630    1.0000000000
     6   1   1   1   1
      0.3676728182    1.0000000000
     7   2   2   1   1
      0.3343569831    1.0000000000
     8   3   3   1   1
      0.1442126443    1.0000000000

# RI basis set for Be (all-electron) relative DI metric: 5.5e-03
Be  RI_aug-SZV-MOLOPT-ae_N_RI_024_s_p_d_f_g_h_i_6_2_1_1_0_0_0_error_5.5e-03
   10
     1   0   0   1   1
      0.1163150776    1.0000000000
     2   0   0   1   1
      0.3285248018    1.0000000000
     3   0   0   1   1
      1.1502904587    1.0000000000
     4   0   0   1   1
      4.0868099234    1.0000000000
     5   0   0   1   1
     14.5468289874    1.0000000000
     6   0   0   1   1
     51.8324499125    1.0000000000
     7   1   1   1   1
      0.2315435272    1.0000000000
     8   1   1   1   1
      1.1145091776    1.0000000000
     9   2   2   1   1
      0.2902896713    1.0000000000
    10   3   3   1   1
      1.3313985884    1.0000000000

# RI basis set for Be (all-electron) relative DI metric: 4.1e-06
Be  RI_aug-SZV-MOLOPT-ae_N_RI_027_s_p_d_f_g_h_i_6_3_1_1_0_0_0_error_4.1e-06
   11
     1   0   0   1   1
      0.1203001266    1.0000000000
     2   0   0   1   1
      0.3303015784    1.0000000000
     3   0   0   1   1
      1.1525134696    1.0000000000
     4   0   0   1   1
      4.0848918101    1.0000000000
     5   0   0   1   1
     14.5072486120    1.0000000000
     6   0   0   1   1
     51.8762391715    1.0000000000
     7   1   1   1   1
      0.1379226490    1.0000000000
     8   1   1   1   1
      0.4441245927    1.0000000000
     9   1   1   1   1
      3.8255393417    1.0000000000
    10   2   2   1   1
      0.3343636816    1.0000000000
    11   3   3   1   1
      0.5216014710    1.0000000000

# RI basis set for Be (all-electron) relative DI metric: 4.8e-07
Be  RI_aug-SZV-MOLOPT-ae_N_RI_035_s_p_d_f_g_h_i_6_4_2_1_0_0_0_error_4.8e-07
   13
     1   0   0   1   1
      0.1188267923    1.0000000000
     2   0   0   1   1
      0.3320087332    1.0000000000
     3   0   0   1   1
      1.1614903191    1.0000000000
     4   0   0   1   1
      4.0777271577    1.0000000000
     5   0   0   1   1
     14.2911118322    1.0000000000
     6   0   0   1   1
     52.1325431967    1.0000000000
     7   1   1   1   1
      0.1364956209    1.0000000000
     8   1   1   1   1
      0.4147703536    1.0000000000
     9   1   1   1   1
      2.4052799662    1.0000000000
    10   1   1   1   1
      6.6324776370    1.0000000000
    11   2   2   1   1
      0.3476984882    1.0000000000
    12   2   2   1   1
      2.0306278019    1.0000000000
    13   3   3   1   1
      0.4962152098    1.0000000000

# RI basis set for Be (all-electron) relative DI metric: 2.2e-07
Be  RI_aug-SZV-MOLOPT-ae_N_RI_038_s_p_d_f_g_h_i_6_5_2_1_0_0_0_error_2.2e-07
   14
     1   0   0   1   1
      0.1241785093    1.0000000000
     2   0   0   1   1
      0.3375851649    1.0000000000
     3   0   0   1   1
      1.1718281073    1.0000000000
     4   0   0   1   1
      4.0598250771    1.0000000000
     5   0   0   1   1
     14.1100658911    1.0000000000
     6   0   0   1   1
     52.3133931096    1.0000000000
     7   1   1   1   1
      0.1272141107    1.0000000000
```

```
     8   1   1   1   1
      0.2988603582    1.0000000000
     9   1   1   1   1
      0.7379864384    1.0000000000
    10   1   1   1   1
      2.7921786623    1.0000000000
    11   1   1   1   1
      9.8199888918    1.0000000000
    12   2   2   1   1
      0.3502253661    1.0000000000
    13   2   2   1   1
      1.9331712918    1.0000000000
    14   3   3   1   1
      0.4980086817    1.0000000000

# RI basis set for Be (all-electron) relative DI metric: 6.2e-08
Be  RI_aug-SZV-MOLOPT-ae_N_RI_048_s_p_d_f_g_h_i_6_5_4_1_0_0_0_error_6.2e-08
   16
     1   0   0   1   1
      0.1225660465    1.0000000000
     2   0   0   1   1
      0.3295049378    1.0000000000
     3   0   0   1   1
      1.1503567196    1.0000000000
     4   0   0   1   1
      4.0841110637    1.0000000000
     5   0   0   1   1
     14.5274163477    1.0000000000
     6   0   0   1   1
     51.8511881076    1.0000000000
     7   1   1   1   1
      0.1386836024    1.0000000000
     8   1   1   1   1
      0.3817246510    1.0000000000
     9   1   1   1   1
      1.2396943852    1.0000000000
    10   1   1   1   1
      3.4766497167    1.0000000000
    11   1   1   1   1
      9.7144319908    1.0000000000
    12   2   2   1   1
      0.1399463761    1.0000000000
    13   2   2   1   1
      0.3511424504    1.0000000000
    14   2   2   1   1
      1.1196655217    1.0000000000
    15   2   2   1   1
      3.2549751676    1.0000000000
    16   3   3   1   1
      0.4982903298    1.0000000000

# RI basis set for Be (all-electron) relative DI metric: 2.9e-08
Be  RI_aug-SZV-MOLOPT-ae_N_RI_063_s_p_d_f_g_h_i_7_5_5_1_1_0_0_error_2.9e-08
   19
     1   0   0   1   1
      0.1197814148    1.0000000000
     2   0   0   1   1
      0.2669704181    1.0000000000
     3   0   0   1   1
      0.7463987749    1.0000000000
     4   0   0   1   1
      2.1578519457    1.0000000000
     5   0   0   1   1
      6.2404460089    1.0000000000
     6   0   0   1   1
     17.9965325035    1.0000000000
     7   0   0   1   1
     51.8140965260    1.0000000000
     8   1   1   1   1
      0.1294098831    1.0000000000
     9   1   1   1   1
      0.3661819458    1.0000000000
    10   1   1   1   1
      1.2482207007    1.0000000000
    11   1   1   1   1
      3.4892287599    1.0000000000
    12   1   1   1   1
      9.6985294681    1.0000000000
    13   2   2   1   1
      0.1369009410    1.0000000000
    14   2   2   1   1
      0.3024852287    1.0000000000
    15   2   2   1   1
      0.6680227180    1.0000000000
    16   2   2   1   1
      1.4753766262    1.0000000000
    17   2   2   1   1
      3.2586449593    1.0000000000
    18   3   3   1   1
      0.4991852113    1.0000000000
    19   4   4   1   1
      0.5156063055    1.0000000000

# RI basis set for Be (all-electron) relative DI metric: 7.3e-09
Be  RI_aug-SZV-MOLOPT-ae_N_RI_117_s_p_d_f_g_h_i_7_6_6_5_3_0_0_error_7.3e-09
   27
     1   0   0   1   1
      0.1225575334    1.0000000000
     2   0   0   1   1
      0.2659044433    1.0000000000
     3   0   0   1   1
      0.7438608774    1.0000000000
     4   0   0   1   1
      2.1535602804    1.0000000000
     5   0   0   1   1
      6.2371673986    1.0000000000
     6   0   0   1   1
     17.9986800169    1.0000000000
     7   0   0   1   1
     51.8090911778    1.0000000000
     8   1   1   1   1
```

```
      0.1095529187    1.0000000000
     9   1   1   1   1
      0.1951024723    1.0000000000
    10   1   1   1   1
      0.5149136540    1.0000000000
    11   1   1   1   1
      1.4116400215    1.0000000000
    12   1   1   1   1
      3.7137780979    1.0000000000
    13   1   1   1   1
      9.7326451726    1.0000000000
    14   2   2   1   1
      0.1369498480    1.0000000000
    15   2   2   1   1
      0.2581458049    1.0000000000
    16   2   2   1   1
      0.4865941315    1.0000000000
    17   2   2   1   1
      0.9171878545    1.0000000000
    18   2   2   1   1
      1.7288251943    1.0000000000
    19   2   2   1   1
      3.2587000000    1.0000000000
    20   3   3   1   1
      0.2055270886    1.0000000000
    21   3   3   1   1
      0.3295494446    1.0000000000
    22   3   3   1   1
      0.5289064732    1.0000000000
    23   3   3   1   1
      0.8490444610    1.0000000000
    24   3   3   1   1
      1.3631103378    1.0000000000
    25   4   4   1   1
      0.3810209324    1.0000000000
    26   4   4   1   1
      0.5468838517    1.0000000000
    27   4   4   1   1
      0.7857014957    1.0000000000

# RI basis set for Be (all-electron) relative DI metric: 2.6e-03
Be  RI_aug-DZVP-MOLOPT-ae_N_RI_019_s_p_d_f_g_h_i_5_3_1_0_0_0_0_error_2.6e-03
   9
     1   0   0   1   1
      0.1198707147    1.0000000000
     2   0   0   1   1
      0.3254757997    1.0000000000
     3   0   0   1   1
      1.1457928926    1.0000000000
     4   0   0   1   1
      5.7015562658    1.0000000000
     5   0   0   1   1
     23.9486351711    1.0000000000
     6   1   1   1   1
      0.2231118190    1.0000000000
     7   1   1   1   1
      0.5523255671    1.0000000000
     8   1   1   1   1
      6.6972274731    1.0000000000
     9   2   2   1   1
      0.3886492407    1.0000000000

# RI basis set for Be (all-electron) relative DI metric: 5.7e-04
Be  RI_aug-DZVP-MOLOPT-ae_N_RI_026_s_p_d_f_g_h_i_7_3_2_0_0_0_0_error_5.7e-04
   12
     1   0   0   1   1
      0.1091644292    1.0000000000
     2   0   0   1   1
      0.2631433042    1.0000000000
     3   0   0   1   1
      0.7403394424    1.0000000000
     4   0   0   1   1
      2.1404593934    1.0000000000
     5   0   0   1   1
      6.2021703199    1.0000000000
     6   0   0   1   1
     17.9181310146    1.0000000000
     7   0   0   1   1
     51.8416536139    1.0000000000
     8   1   1   1   1
      0.1930994601    1.0000000000
     9   1   1   1   1
      0.4881078158    1.0000000000
    10   1   1   1   1
      7.5994984116    1.0000000000
    11   2   2   1   1
      0.1918665211    1.0000000000
    12   2   2   1   1
      0.7771889181    1.0000000000

# RI basis set for Be (all-electron) relative DI metric: 1.3e-04
Be  RI_aug-DZVP-MOLOPT-ae_N_RI_029_s_p_d_f_g_h_i_7_4_2_0_0_0_0_error_1.3e-04
   13
     1   0   0   1   1
      0.1326320521    1.0000000000
     2   0   0   1   1
      0.2361394797    1.0000000000
     3   0   0   1   1
      0.6206749778    1.0000000000
     4   0   0   1   1
      1.8822829757    1.0000000000
     5   0   0   1   1
      5.7330340727    1.0000000000
     6   0   0   1   1
     16.9622307974    1.0000000000
     7   0   0   1   1
     52.3606261638    1.0000000000
     8   1   1   1   1
      0.1858441860    1.0000000000
     9   1   1   1   1
      0.3874522392    1.0000000000
```

```
    10   1   1   1   1
      1.2535607486    1.0000000000
    11   1   1   1   1
      6.6628211122    1.0000000000
    12   2   2   1   1
      0.1462626809    1.0000000000
    13   2   2   1   1
      0.4744052978    1.0000000000

# RI basis set for Be (all-electron) relative DI metric: 5.7e-05
Be  RI_aug-DZVP-MOLOPT-ae_N_RI_039_s_p_d_f_g_h_i_7_5_2_1_0_0_0_error_5.7e-05
   15
     1   0   0   1   1
      0.1014525092    1.0000000000
     2   0   0   1   1
      0.2370988269    1.0000000000
     3   0   0   1   1
      0.6786159070    1.0000000000
     4   0   0   1   1
      2.1856352842    1.0000000000
     5   0   0   1   1
      6.5717724827    1.0000000000
     6   0   0   1   1
     18.6856135032    1.0000000000
     7   0   0   1   1
     53.7675277281    1.0000000000
     8   1   1   1   1
      0.1308632403    1.0000000000
     9   1   1   1   1
      0.2417037867    1.0000000000
    10   1   1   1   1
      0.7034589586    1.0000000000
    11   1   1   1   1
      2.1950437215    1.0000000000
    12   1   1   1   1
      7.4680114409    1.0000000000
    13   2   2   1   1
      0.1399642511    1.0000000000
    14   2   2   1   1
      0.4440961539    1.0000000000
    15   3   3   1   1
      0.3562115345    1.0000000000

# RI basis set for Be (all-electron) relative DI metric: 2.1e-05
Be  RI_aug-DZVP-MOLOPT-ae_N_RI_075_s_p_d_f_g_h_i_7_5_5_4_0_0_0_error_2.1e-05
   21
     1   0   0   1   1
      0.1060202334    1.0000000000
     2   0   0   1   1
      0.2768363781    1.0000000000
     3   0   0   1   1
      0.8480252293    1.0000000000
     4   0   0   1   1
      2.6348430545    1.0000000000
     5   0   0   1   1
      7.5426145341    1.0000000000
     6   0   0   1   1
     20.8007199984    1.0000000000
     7   0   0   1   1
     57.1643594382    1.0000000000
     8   1   1   1   1
      0.1021916652    1.0000000000
     9   1   1   1   1
      0.2537391170    1.0000000000
    10   1   1   1   1
      0.6800787343    1.0000000000
    11   1   1   1   1
      2.4616499447    1.0000000000
    12   1   1   1   1
      7.5195956449    1.0000000000
    13   2   2   1   1
      0.1317062989    1.0000000000
    14   2   2   1   1
      0.3023965844    1.0000000000
    15   2   2   1   1
      0.6681022770    1.0000000000
    16   2   2   1   1
      1.4751826616    1.0000000000
    17   2   2   1   1
      3.2616090078    1.0000000000
    18   3   3   1   1
      0.1300362526    1.0000000000
    19   3   3   1   1
      0.3428480667    1.0000000000
    20   3   3   1   1
      0.6709593480    1.0000000000
    21   3   3   1   1
      1.2898409224    1.0000000000

# RI basis set for Be (all-electron) relative DI metric: 2.1e-02
Be  RI_aug-TZVP-MOLOPT-ae_N_RI_019_s_p_d_f_g_h_i_5_3_1_0_0_0_0_error_2.1e-02
   9
     1   0   0   1   1
      0.1870555794    1.0000000000
     2   0   0   1   1
      0.2932248365    1.0000000000
     3   0   0   1   1
      2.7941764838    1.0000000000
     4   0   0   1   1
     14.2014704718    1.0000000000
     5   0   0   1   1
     36.0370522688    1.0000000000
     6   1   1   1   1
      0.2151540236    1.0000000000
     7   1   1   1   1
      0.5959733511    1.0000000000
     8   1   1   1   1
     11.4985054482    1.0000000000
     9   2   2   1   1
      0.3705402825    1.0000000000
```

```
# RI basis set for Be (all-electron) relative DI metric: 5.7e-03
Be  RI_aug-TZVP-MOLOPT-ae_N_RI_029_s_p_d_f_g_h_i_5_4_1_1_0_0_0_error_5.7e-03
   11
    1   0   0   1   1
      0.1774342505    1.0000000000
    2   0   0   1   1
      0.3111843177    1.0000000000
    3   0   0   1   1
      2.5577684354    1.0000000000
    4   0   0   1   1
     13.5055251484    1.0000000000
    5   0   0   1   1
     40.5085389935    1.0000000000
    6   1   1   1   1
      0.2198595722    1.0000000000
    7   1   1   1   1
      0.5326574240    1.0000000000
    8   1   1   1   1
      3.0339484500    1.0000000000
    9   1   1   1   1
     12.2140323684    1.0000000000
   10   2   2   1   1
      0.3705155199    1.0000000000
   11   3   3   1   1
      0.3272528852    1.0000000000

# RI basis set for Be (all-electron) relative DI metric: 2.3e-03
Be  RI_aug-TZVP-MOLOPT-ae_N_RI_034_s_p_d_f_g_h_i_5_4_2_1_0_0_0_error_2.3e-03
   12
    1   0   0   1   1
      0.1474559457    1.0000000000
    2   0   0   1   1
      0.4218923632    1.0000000000
    3   0   0   1   1
      2.6215398637    1.0000000000
    4   0   0   1   1
     12.5267691122    1.0000000000
    5   0   0   1   1
     38.7373161979    1.0000000000
    6   1   1   1   1
      0.2243626406    1.0000000000
    7   1   1   1   1
      0.5261490554    1.0000000000
    8   1   1   1   1
      2.7911114549    1.0000000000
    9   1   1   1   1
     12.2923551765    1.0000000000
   10   2   2   1   1
      0.2765289841    1.0000000000
   11   2   2   1   1
      0.6806371634    1.0000000000
   12   3   3   1   1
      0.3471314856    1.0000000000

# RI basis set for Be (all-electron) relative DI metric: 9.3e-04
Be  RI_aug-TZVP-MOLOPT-ae_N_RI_041_s_p_d_f_g_h_i_7_4_3_1_0_0_0_error_9.3e-04
   15
    1   0   0   1   1
      0.1251653393    1.0000000000
    2   0   0   1   1
      0.2477193025    1.0000000000
    3   0   0   1   1
      0.6185492738    1.0000000000
    4   0   0   1   1
      1.7915841586    1.0000000000
    5   0   0   1   1
      5.3969925817    1.0000000000
    6   0   0   1   1
     16.4977603871    1.0000000000
    7   0   0   1   1
     54.1690233305    1.0000000000
    8   1   1   1   1
      0.2192458032    1.0000000000
    9   1   1   1   1
      0.5542747049    1.0000000000
   10   1   1   1   1
      2.8525734383    1.0000000000
   11   1   1   1   1
     11.6224311224    1.0000000000
   12   2   2   1   1
      0.1719014034    1.0000000000
   13   2   2   1   1
      0.4275258226    1.0000000000
   14   2   2   1   1
      1.0585552117    1.0000000000
   15   3   3   1   1
      0.3289432139    1.0000000000

# RI basis set for Be (all-electron) relative DI metric: 4.3e-04
Be  RI_aug-TZVP-MOLOPT-ae_N_RI_049_s_p_d_f_g_h_i_7_5_4_1_0_0_0_error_4.3e-04
   17
    1   0   0   1   1
      0.1281322053    1.0000000000
    2   0   0   1   1
      0.2233997882    1.0000000000
    3   0   0   1   1
      0.5632364748    1.0000000000
    4   0   0   1   1
      1.6203212704    1.0000000000
    5   0   0   1   1
      4.8017506176    1.0000000000
    6   0   0   1   1
     14.7567942064    1.0000000000
    7   0   0   1   1
     58.6787256784    1.0000000000
    8   1   1   1   1
      0.1108794740    1.0000000000
    9   1   1   1   1
      0.2364345855    1.0000000000
   10   1   1   1   1
      0.6858646432    1.0000000000
```

```
    11   1   1   1   1
      3.3743512426    1.0000000000
    12   1   1   1   1
     11.9836318840    1.0000000000
    13   2   2   1   1
      0.1829245769    1.0000000000
    14   2   2   1   1
      0.6454397254    1.0000000000
    15   2   2   1   1
      2.3266627020    1.0000000000
    16   2   2   1   1
      8.3921164943    1.0000000000
    17   3   3   1   1
      0.3536736931    1.0000000000

# RI basis set for Be (all-electron) relative DI metric: 1.5e-04
Be  RI_aug-TZVP-MOLOPT-ae_N_RI_058_s_p_d_f_g_h_i_7_5_4_1_1_0_0_error_1.5e-04
   18
     1   0   0   1   1
      0.1399358111    1.0000000000
     2   0   0   1   1
      0.2275133343    1.0000000000
     3   0   0   1   1
      0.5656105950    1.0000000000
     4   0   0   1   1
      1.6291491274    1.0000000000
     5   0   0   1   1
      4.8392086696    1.0000000000
     6   0   0   1   1
     14.9278652608    1.0000000000
     7   0   0   1   1
     58.9595185139    1.0000000000
     8   1   1   1   1
      0.1127554502    1.0000000000
     9   1   1   1   1
      0.2318756824    1.0000000000
    10   1   1   1   1
      0.6820254920    1.0000000000
    11   1   1   1   1
      3.3877210643    1.0000000000
    12   1   1   1   1
     12.0060347323    1.0000000000
    13   2   2   1   1
      0.2124705501    1.0000000000
    14   2   2   1   1
      0.3498892404    1.0000000000
    15   2   2   1   1
      1.5605393383    1.0000000000
    16   2   2   1   1
      8.8372953922    1.0000000000
    17   3   3   1   1
      0.3715200433    1.0000000000
    18   4   4   1   1
      0.1369188316    1.0000000000

# RI basis set for Be (all-electron) relative DI metric: 6.6e-05
Be  RI_aug-TZVP-MOLOPT-ae_N_RI_063_s_p_d_f_g_h_i_7_5_5_1_1_0_0_error_6.6e-05
   19
     1   0   0   1   1
      0.1158841857    1.0000000000
     2   0   0   1   1
      0.2025966865    1.0000000000
     3   0   0   1   1
      0.5452636265    1.0000000000
     4   0   0   1   1
      1.5658945512    1.0000000000
     5   0   0   1   1
      4.5631324597    1.0000000000
     6   0   0   1   1
     13.6312997217    1.0000000000
     7   0   0   1   1
     63.0468497053    1.0000000000
     8   1   1   1   1
      0.1105607661    1.0000000000
     9   1   1   1   1
      0.2298000164    1.0000000000
    10   1   1   1   1
      0.6547022920    1.0000000000
    11   1   1   1   1
      3.6036789345    1.0000000000
    12   1   1   1   1
     12.0103858263    1.0000000000
    13   2   2   1   1
      0.1173634209    1.0000000000
    14   2   2   1   1
      0.3591563309    1.0000000000
    15   2   2   1   1
      1.0920707252    1.0000000000
    16   2   2   1   1
      3.3324036866    1.0000000000
    17   2   2   1   1
     10.1947129487    1.0000000000
    18   3   3   1   1
      0.3526519108    1.0000000000
    19   4   4   1   1
      0.1576113299    1.0000000000

# RI basis set for Be (all-electron) relative DI metric: 2.1e-05
Be  RI_aug-TZVP-MOLOPT-ae_N_RI_093_s_p_d_f_g_h_i_7_5_5_4_2_0_0_error_2.1e-05
   23
     1   0   0   1   1
      0.1168437801    1.0000000000
     2   0   0   1   1
      0.1981447580    1.0000000000
     3   0   0   1   1
      0.5458946432    1.0000000000
     4   0   0   1   1
      1.5657311674    1.0000000000
     5   0   0   1   1
      4.5571801046    1.0000000000
     6   0   0   1   1
```

```
      13.5091479487    1.0000000000
      7   0   0   1   1
      63.9251322318    1.0000000000
      8   1   1   1   1
       0.1230808212    1.0000000000
      9   1   1   1   1
       0.2087985094    1.0000000000
     10   1   1   1   1
       0.6515376746    1.0000000000
     11   1   1   1   1
       4.2055711855    1.0000000000
     12   1   1   1   1
      12.1483185924    1.0000000000
     13   2   2   1   1
       0.1346246712    1.0000000000
     14   2   2   1   1
       0.3959135282    1.0000000000
     15   2   2   1   1
       1.1802436195    1.0000000000
     16   2   2   1   1
       3.4705494523    1.0000000000
     17   2   2   1   1
      10.4634289747    1.0000000000
     18   3   3   1   1
       0.1380217807    1.0000000000
     19   3   3   1   1
       0.3533670144    1.0000000000
     20   3   3   1   1
       0.6975153228    1.0000000000
     21   3   3   1   1
       1.3508238423    1.0000000000
     22   4   4   1   1
       0.1738343968    1.0000000000
     23   4   4   1   1
       0.6724171726    1.0000000000

# RI basis set for Be (all-electron) relative DI metric: 9.1e-06
Be  RI_aug-TZVP-MOLOPT-ae_N_RI_112_s_p_d_f_g_h_i_7_6_5_5_3_0_0_error_9.1e-06
   26
      1   0   0   1   1
       0.1143529973    1.0000000000
      2   0   0   1   1
       0.4117242119    1.0000000000
      3   0   0   1   1
       0.7513436070    1.0000000000
      4   0   0   1   1
       1.9115115953    1.0000000000
      5   0   0   1   1
       4.7816706729    1.0000000000
      6   0   0   1   1
      12.5678861425    1.0000000000
      7   0   0   1   1
      71.8344621696    1.0000000000
      8   1   1   1   1
       0.1147719514    1.0000000000
      9   1   1   1   1
       0.2470098577    1.0000000000
     10   1   1   1   1
       0.6402742942    1.0000000000
     11   1   1   1   1
       1.6332128386    1.0000000000
     12   1   1   1   1
       4.4128305246    1.0000000000
     13   1   1   1   1
      12.1244242727    1.0000000000
     14   2   2   1   1
       0.1407702354    1.0000000000
     15   2   2   1   1
       0.3874456634    1.0000000000
     16   2   2   1   1
       1.0144554849    1.0000000000
     17   2   2   1   1
       3.2583356502    1.0000000000
     18   2   2   1   1
      10.3252064136    1.0000000000
     19   3   3   1   1
       0.1535205182    1.0000000000
     20   3   3   1   1
       0.2901096106    1.0000000000
     21   3   3   1   1
       0.4889628210    1.0000000000
     22   3   3   1   1
       0.8141034745    1.0000000000
     23   3   3   1   1
       1.3393348464    1.0000000000
     24   4   4   1   1
       0.1938105962    1.0000000000
     25   4   4   1   1
       0.3526269336    1.0000000000
     26   4   4   1   1
       0.5742579315    1.0000000000


# RI basis set for B (all-electron) relative DI metric: 2.0e-02
B  RI_aug-SZV-MOLOPT-ae_N_RI_017_s_p_d_f_g_h_i_2_1_1_1_0_0_0_error_2.0e-02
   5
      1   0   0   1   1
       1.9536140826    1.0000000000
      2   0   0   1   1
      41.2931581775    1.0000000000
      3   1   1   1   1
       0.5966229391    1.0000000000
      4   2   2   1   1
       0.5394017949    1.0000000000
      5   3   3   1   1
       0.2266443541    1.0000000000

# RI basis set for B (all-electron) relative DI metric: 5.8e-07
B  RI_aug-SZV-MOLOPT-ae-mini_N_RI_009_s_p_d_f_g_h_i_3_2_0_0_0_0_0_error_5.8e-07
   5
```

```
     1   0   0   1   1
      0.2856215636    1.0000000000
     2   0   0   1   1
      0.8126732788    1.0000000000
     3   0   0   1   1
      3.0529684504    1.0000000000
     4   1   1   1   1
      0.3264938753    1.0000000000
     5   1   1   1   1
      0.8635069462    1.0000000000

# RI basis set for B (all-electron) relative DI metric: 1.2e-07
B  RI_aug-SZV-MOLOPT-ae-mini_N_RI_014_s_p_d_f_g_h_i_5_3_0_0_0_0_0_error_1.2e-07
   8
     1   0   0   1   1
      0.3789426331    1.0000000000
     2   0   0   1   1
      0.6873959454    1.0000000000
     3   0   0   1   1
      1.2314603527    1.0000000000
     4   0   0   1   1
      2.1430160263    1.0000000000
     5   0   0   1   1
      3.7010209697    1.0000000000
     6   1   1   1   1
      0.3541247654    1.0000000000
     7   1   1   1   1
      0.7345081003    1.0000000000
     8   1   1   1   1
      3.3360177769    1.0000000000

# RI basis set for B (all-electron) relative DI metric: 1.9e-08
B  RI_aug-SZV-MOLOPT-ae-mini_N_RI_025_s_p_d_f_g_h_i_7_6_0_0_0_0_0_error_1.9e-08
   13
     1   0   0   1   1
      0.4226550000    1.0000000000
     2   0   0   1   1
      0.6075369656    1.0000000000
     3   0   0   1   1
      0.8732918447    1.0000000000
     4   0   0   1   1
      1.2552958736    1.0000000000
     5   0   0   1   1
      1.8043999149    1.0000000000
     6   0   0   1   1
      2.5936985227    1.0000000000
     7   0   0   1   1
      3.7282600000    1.0000000000
     8   1   1   1   1
      0.4226549998    1.0000000000
     9   1   1   1   1
      0.6532659874    1.0000000000
    10   1   1   1   1
      1.0097040149    1.0000000000
    11   1   1   1   1
      1.5606234172    1.0000000000
    12   1   1   1   1
      2.4121380270    1.0000000000
    13   1   1   1   1
      3.7282599999    1.0000000000

# RI basis set for B (all-electron) relative DI metric: 7.4e-06
B  RI_aug-SZV-MOLOPT-ae_N_RI_025_s_p_d_f_g_h_i_4_3_1_1_0_0_0_error_7.4e-06
   9
     1   0   0   1   1
      0.2503405201    1.0000000000
     2   0   0   1   1
      0.7878125425    1.0000000000
     3   0   0   1   1
      5.9745224766    1.0000000000
     4   0   0   1   1
     47.4325949225    1.0000000000
     5   1   1   1   1
      0.2732835355    1.0000000000
     6   1   1   1   1
      0.7371828437    1.0000000000
     7   1   1   1   1
      7.2472997167    1.0000000000
     8   2   2   1   1
      0.4552986304    1.0000000000
     9   3   3   1   1
      0.6759737321    1.0000000000

# RI basis set for B (all-electron) relative DI metric: 2.2e-06
B  RI_aug-SZV-MOLOPT-ae_N_RI_032_s_p_d_f_g_h_i_6_3_2_1_0_0_0_error_2.2e-06
   12
     1   0   0   1   1
      0.2096188111    1.0000000000
     2   0   0   1   1
      0.6181885944    1.0000000000
     3   0   0   1   1
      1.8231208880    1.0000000000
     4   0   0   1   1
      5.3767102492    1.0000000000
     5   0   0   1   1
     15.8568248146    1.0000000000
     6   0   0   1   1
     46.7643265056    1.0000000000
     7   1   1   1   1
      0.2791733779    1.0000000000
     8   1   1   1   1
      0.7597034155    1.0000000000
     9   1   1   1   1
      8.0104160700    1.0000000000
    10   2   2   1   1
      0.4386074548    1.0000000000
    11   2   2   1   1
      2.2728344217    1.0000000000
    12   3   3   1   1
      0.5504494161    1.0000000000
```

```
# RI basis set for B (all-electron) relative DI metric: 8.4e-07
B  RI_aug-SZV-MOLOPT-ae_N_RI_035_s_p_d_f_g_h_i_6_4_2_1_0_0_0_error_8.4e-07
  13
    1   0   0   1   1
     0.2795590646    1.0000000000
    2   0   0   1   1
     0.6490525811    1.0000000000
    3   0   0   1   1
     1.3641673187    1.0000000000
    4   0   0   1   1
     4.7327099132    1.0000000000
    5   0   0   1   1
    15.3866247994    1.0000000000
    6   0   0   1   1
    46.7578724023    1.0000000000
    7   1   1   1   1
     0.2667829069    1.0000000000
    8   1   1   1   1
     0.7006018892    1.0000000000
    9   1   1   1   1
     2.4338232209    1.0000000000
   10   1   1   1   1
    11.9303964003    1.0000000000
   11   2   2   1   1
     0.4388107433    1.0000000000
   12   2   2   1   1
     1.9054843743    1.0000000000
   13   3   3   1   1
     0.5245098254    1.0000000000

# RI basis set for B (all-electron) relative DI metric: 2.0e-02
B  RI_aug-DZVP-MOLOPT-ae_N_RI_019_s_p_d_f_g_h_i_5_3_1_0_0_0_0_error_2.0e-02
   9
    1   0   0   1   1
     0.2564726306    1.0000000000
    2   0   0   1   1
     0.5597857422    1.0000000000
    3   0   0   1   1
     1.7043028242    1.0000000000
    4   0   0   1   1
     8.9210964200    1.0000000000
    5   0   0   1   1
    46.0715304658    1.0000000000
    6   1   1   1   1
     0.3394107448    1.0000000000
    7   1   1   1   1
     0.8007981726    1.0000000000
    8   1   1   1   1
     4.7671779140    1.0000000000
    9   2   2   1   1
     0.7924889026    1.0000000000

# RI basis set for B (all-electron) relative DI metric: 2.3e-03
B  RI_aug-DZVP-MOLOPT-ae_N_RI_026_s_p_d_f_g_h_i_5_3_1_1_0_0_0_error_2.3e-03
  10
    1   0   0   1   1
     0.2531919204    1.0000000000
    2   0   0   1   1
     0.5567015753    1.0000000000
    3   0   0   1   1
     1.6880530896    1.0000000000
    4   0   0   1   1
     8.8434280515    1.0000000000
    5   0   0   1   1
    45.9906164530    1.0000000000
    6   1   1   1   1
     0.3078115919    1.0000000000
    7   1   1   1   1
     0.7055911376    1.0000000000
    8   1   1   1   1
     1.6287629371    1.0000000000
    9   2   2   1   1
     0.6590424988    1.0000000000
   10   3   3   1   1
     0.5130681309    1.0000000000

# RI basis set for B (all-electron) relative DI metric: 4.1e-05
B  RI_aug-DZVP-MOLOPT-ae_N_RI_035_s_p_d_f_g_h_i_6_4_2_1_0_0_0_error_4.1e-05
  13
    1   0   0   1   1
     0.1552970154    1.0000000000
    2   0   0   1   1
     0.3482034165    1.0000000000
    3   0   0   1   1
     1.1145969782    1.0000000000
    4   0   0   1   1
     3.9529246997    1.0000000000
    5   0   0   1   1
    13.7870973928    1.0000000000
    6   0   0   1   1
    47.4916354174    1.0000000000
    7   1   1   1   1
     0.2801076159    1.0000000000
    8   1   1   1   1
     0.4443360202    1.0000000000
    9   1   1   1   1
     1.4008888782    1.0000000000
   10   1   1   1   1
     6.6588111268    1.0000000000
   11   2   2   1   1
     0.1911013608    1.0000000000
   12   2   2   1   1
     0.7616868087    1.0000000000
   13   3   3   1   1
     0.4017000541    1.0000000000

# RI basis set for B (all-electron) relative DI metric: 1.5e-05
B  RI_aug-DZVP-MOLOPT-ae_N_RI_045_s_p_d_f_g_h_i_6_5_2_2_0_0_0_error_1.5e-05
  15
    1   0   0   1   1
     0.1718701132    1.0000000000
```

```
     2   0   0   1   1
      0.3480828739    1.0000000000
     3   0   0   1   1
      1.0648940759    1.0000000000
     4   0   0   1   1
      3.8839828905    1.0000000000
     5   0   0   1   1
     13.7366807467    1.0000000000
     6   0   0   1   1
     47.4452029724    1.0000000000
     7   1   1   1   1
      0.2282231194    1.0000000000
     8   1   1   1   1
      0.4695895530    1.0000000000
     9   1   1   1   1
      1.3610136161    1.0000000000
    10   1   1   1   1
      4.0023213190    1.0000000000
    11   1   1   1   1
     12.2019647697    1.0000000000
    12   2   2   1   1
      0.1614610529    1.0000000000
    13   2   2   1   1
      0.7726964308    1.0000000000
    14   3   3   1   1
      0.3422593493    1.0000000000
    15   3   3   1   1
      1.5468148392    1.0000000000

# RI basis set for B (all-electron) relative DI metric: 5.2e-06
B  RI_aug-DZVP-MOLOPT-ae_N_RI_060_s_p_d_f_g_h_i_6_5_5_2_0_0_0_error_5.2e-06
   18
     1   0   0   1   1
      0.2044863231    1.0000000000
     2   0   0   1   1
      0.3366459763    1.0000000000
     3   0   0   1   1
      0.8423151354    1.0000000000
     4   0   0   1   1
      3.2832130041    1.0000000000
     5   0   0   1   1
     13.2191036972    1.0000000000
     6   0   0   1   1
     47.0152130709    1.0000000000
     7   1   1   1   1
      0.2278202832    1.0000000000
     8   1   1   1   1
      0.5191913562    1.0000000000
     9   1   1   1   1
      1.2704821526    1.0000000000
    10   1   1   1   1
      3.8238353693    1.0000000000
    11   1   1   1   1
     12.1110876725    1.0000000000
    12   2   2   1   1
      0.1633837895    1.0000000000
    13   2   2   1   1
      0.3548407526    1.0000000000
    14   2   2   1   1
      0.7734409840    1.0000000000
    15   2   2   1   1
      1.6552406213    1.0000000000
    16   2   2   1   1
      3.5941425639    1.0000000000
    17   3   3   1   1
      0.3299952743    1.0000000000
    18   3   3   1   1
      1.3274783395    1.0000000000

# RI basis set for B (all-electron) relative DI metric: 9.1e-07
B  RI_aug-DZVP-MOLOPT-ae_N_RI_070_s_p_d_f_g_h_i_7_5_5_2_1_0_0_error_9.1e-07
   20
     1   0   0   1   1
      0.1773659770    1.0000000000
     2   0   0   1   1
      0.3175366721    1.0000000000
     3   0   0   1   1
      0.7074629922    1.0000000000
     4   0   0   1   1
      2.0199307728    1.0000000000
     5   0   0   1   1
      5.9270669433    1.0000000000
     6   0   0   1   1
     16.8628249356    1.0000000000
     7   0   0   1   1
     47.4043896805    1.0000000000
     8   1   1   1   1
      0.2235997374    1.0000000000
     9   1   1   1   1
      0.5239921141    1.0000000000
    10   1   1   1   1
      1.2791701953    1.0000000000
    11   1   1   1   1
      4.0687944057    1.0000000000
    12   1   1   1   1
     11.7068863231    1.0000000000
    13   2   2   1   1
      0.1524853889    1.0000000000
    14   2   2   1   1
      0.3552298298    1.0000000000
    15   2   2   1   1
      0.7711794526    1.0000000000
    16   2   2   1   1
      1.6605429017    1.0000000000
    17   2   2   1   1
      3.5985481333    1.0000000000
    18   3   3   1   1
      0.3187151452    1.0000000000
    19   3   3   1   1
      1.0339908044    1.0000000000
    20   4   4   1   1
```

```
      0.6493287535    1.0000000000

# RI basis set for B (all-electron) relative DI metric: 3.5e-07
B  RI_aug-DZVP-MOLOPT-ae_N_RI_115_s_p_d_f_g_h_i_7_5_5_5_1_1_1_error_3.5e-07
   25
     1   0   0   1   1
      0.1734220838    1.0000000000
     2   0   0   1   1
      0.3208947428    1.0000000000
     3   0   0   1   1
      0.7440076161    1.0000000000
     4   0   0   1   1
      2.0677296718    1.0000000000
     5   0   0   1   1
      5.9715250288    1.0000000000
     6   0   0   1   1
     16.9083520127    1.0000000000
     7   0   0   1   1
     47.4517765035    1.0000000000
     8   1   1   1   1
      0.2202878947    1.0000000000
     9   1   1   1   1
      0.4941093091    1.0000000000
    10   1   1   1   1
      1.4390234887    1.0000000000
    11   1   1   1   1
      4.0054845836    1.0000000000
    12   1   1   1   1
     12.2025491582    1.0000000000
    13   2   2   1   1
      0.1706233174    1.0000000000
    14   2   2   1   1
      0.3559161656    1.0000000000
    15   2   2   1   1
      0.7736745534    1.0000000000
    16   2   2   1   1
      1.6668166219    1.0000000000
    17   2   2   1   1
      3.6009638067    1.0000000000
    18   3   3   1   1
      0.2385153640    1.0000000000
    19   3   3   1   1
      0.3870522027    1.0000000000
    20   3   3   1   1
      0.6276442351    1.0000000000
    21   3   3   1   1
      1.0136901365    1.0000000000
    22   3   3   1   1
      1.6360602693    1.0000000000
    23   4   4   1   1
      0.6039468505    1.0000000000
    24   5   5   1   1
      1.0389702720    1.0000000000
    25   6   6   1   1
      1.1280327774    1.0000000000

# RI basis set for B (all-electron) relative DI metric: 3.8e-03
B  RI_aug-TZVP-MOLOPT-ae_N_RI_029_s_p_d_f_g_h_i_5_4_1_1_0_0_0_error_3.8e-03
   11
     1   0   0   1   1
      0.2524202319    1.0000000000
     2   0   0   1   1
      0.4265722672    1.0000000000
     3   0   0   1   1
      1.4824592982    1.0000000000
     4   0   0   1   1
      7.7968477178    1.0000000000
     5   0   0   1   1
     43.8112330272    1.0000000000
     6   1   1   1   1
      0.2976680716    1.0000000000
     7   1   1   1   1
      0.8089852537    1.0000000000
     8   1   1   1   1
      2.7907723928    1.0000000000
     9   1   1   1   1
      9.1572445847    1.0000000000
    10   2   2   1   1
      0.7024476962    1.0000000000
    11   3   3   1   1
      0.8247722601    1.0000000000

# RI basis set for B (all-electron) relative DI metric: 5.1e-04
B  RI_aug-TZVP-MOLOPT-ae_N_RI_034_s_p_d_f_g_h_i_5_4_2_1_0_0_0_error_5.1e-04
   12
     1   0   0   1   1
      0.1848683764    1.0000000000
     2   0   0   1   1
      0.3933770513    1.0000000000
     3   0   0   1   1
      1.1147311334    1.0000000000
     4   0   0   1   1
      5.6007277177    1.0000000000
     5   0   0   1   1
     35.1186194522    1.0000000000
     6   1   1   1   1
      0.2969247069    1.0000000000
     7   1   1   1   1
      0.4421497519    1.0000000000
     8   1   1   1   1
      1.9949113190    1.0000000000
     9   1   1   1   1
     10.5475889670    1.0000000000
    10   2   2   1   1
      0.3911041448    1.0000000000
    11   2   2   1   1
      0.9375856244    1.0000000000
    12   3   3   1   1
      0.7666416517    1.0000000000

# RI basis set for B (all-electron) relative DI metric: 2.6e-04
```

```
B  RI_aug-TZVP-MOLOPT-ae_N_RI_043_s_p_d_f_g_h_i_6_5_3_1_0_0_0_error_2.6e-04
   15
    1   0   0   1   1
      0.1880618703    1.0000000000
    2   0   0   1   1
      0.3123483443    1.0000000000
    3   0   0   1   1
      0.9753573507    1.0000000000
    4   0   0   1   1
      3.4317307741    1.0000000000
    5   0   0   1   1
     12.7641328041    1.0000000000
    6   0   0   1   1
     46.2395909795    1.0000000000
    7   1   1   1   1
      0.1838853606    1.0000000000
    8   1   1   1   1
      0.4924576967    1.0000000000
    9   1   1   1   1
      1.1923760727    1.0000000000
   10   1   1   1   1
      3.9446023358    1.0000000000
   11   1   1   1   1
     12.2286111122    1.0000000000
   12   2   2   1   1
      0.2538024037    1.0000000000
   13   2   2   1   1
      0.5576538341    1.0000000000
   14   2   2   1   1
      1.1491110018    1.0000000000
   15   3   3   1   1
      0.7813893372    1.0000000000

# RI basis set for B (all-electron) relative DI metric: 4.2e-05
B  RI_aug-TZVP-MOLOPT-ae_N_RI_059_s_p_d_f_g_h_i_6_5_3_2_1_0_0_error_4.2e-05
   17
    1   0   0   1   1
      0.2020070642    1.0000000000
    2   0   0   1   1
      0.2962409718    1.0000000000
    3   0   0   1   1
      0.9348951526    1.0000000000
    4   0   0   1   1
      3.3635597346    1.0000000000
    5   0   0   1   1
     12.6675671079    1.0000000000
    6   0   0   1   1
     46.1313627817    1.0000000000
    7   1   1   1   1
      0.1883716547    1.0000000000
    8   1   1   1   1
      0.4829326176    1.0000000000
    9   1   1   1   1
      1.1669707875    1.0000000000
   10   1   1   1   1
      3.8752642566    1.0000000000
   11   1   1   1   1
     12.1625382358    1.0000000000
   12   2   2   1   1
      0.1927728654    1.0000000000
   13   2   2   1   1
      0.5438829563    1.0000000000
   14   2   2   1   1
      1.1626953624    1.0000000000
   15   3   3   1   1
      0.2205426254    1.0000000000
   16   3   3   1   1
      0.8059823055    1.0000000000
   17   4   4   1   1
      0.5089116277    1.0000000000

# RI basis set for B (all-electron) relative DI metric: 1.2e-05
B  RI_aug-TZVP-MOLOPT-ae_N_RI_073_s_p_d_f_g_h_i_6_5_4_2_2_0_0_error_1.2e-05
   19
    1   0   0   1   1
      0.1530229343    1.0000000000
    2   0   0   1   1
      0.3803950221    1.0000000000
    3   0   0   1   1
      0.9297305065    1.0000000000
    4   0   0   1   1
      3.1408411688    1.0000000000
    5   0   0   1   1
     12.0601336716    1.0000000000
    6   0   0   1   1
     45.3274746487    1.0000000000
    7   1   1   1   1
      0.1599145932    1.0000000000
    8   1   1   1   1
      0.4890508250    1.0000000000
    9   1   1   1   1
      1.1115500104    1.0000000000
   10   1   1   1   1
      3.8173807021    1.0000000000
   11   1   1   1   1
     12.0636785241    1.0000000000
   12   2   2   1   1
      0.1807472194    1.0000000000
   13   2   2   1   1
      0.5306696038    1.0000000000
   14   2   2   1   1
      1.3769613680    1.0000000000
   15   2   2   1   1
      3.6971991940    1.0000000000
   16   3   3   1   1
      0.1875532938    1.0000000000
   17   3   3   1   1
      0.8372546215    1.0000000000
   18   4   4   1   1
      0.2488964568    1.0000000000
   19   4   4   1   1
```

```
      0.9679395434    1.0000000000

# RI basis set for B (all-electron) relative DI metric: 2.1e-06
B  RI_aug-TZVP-MOLOPT-ae_N_RI_100_s_p_d_f_g_h_i_6_5_4_3_3_1_0_error_2.1e-06
   22
     1   0   0   1   1
      0.1632978080    1.0000000000
     2   0   0   1   1
      0.3043203694    1.0000000000
     3   0   0   1   1
      1.0731289130    1.0000000000
     4   0   0   1   1
      3.6192731436    1.0000000000
     5   0   0   1   1
     13.0601773480    1.0000000000
     6   0   0   1   1
     46.5839292943    1.0000000000
     7   1   1   1   1
      0.1621382301    1.0000000000
     8   1   1   1   1
      0.4546183294    1.0000000000
     9   1   1   1   1
      1.1014930363    1.0000000000
    10   1   1   1   1
      3.9693787168    1.0000000000
    11   1   1   1   1
     12.2154888474    1.0000000000
    12   2   2   1   1
      0.1620114045    1.0000000000
    13   2   2   1   1
      0.5413195777    1.0000000000
    14   2   2   1   1
      1.4872996869    1.0000000000
    15   2   2   1   1
      3.9298507145    1.0000000000
    16   3   3   1   1
      0.1836690476    1.0000000000
    17   3   3   1   1
      0.6313504996    1.0000000000
    18   3   3   1   1
      1.6049622806    1.0000000000
    19   4   4   1   1
      0.1930818476    1.0000000000
    20   4   4   1   1
      0.4118707386    1.0000000000
    21   4   4   1   1
      0.9358813053    1.0000000000
    22   5   5   1   1
      1.0470452584    1.0000000000

# RI basis set for C (all-electron) relative DI metric: 4.2e-03
C  RI_aug-SZV-MOLOPT-ae-mini_N_RI_011_s_p_d_f_g_h_i_5_2_0_0_0_0_0_error_4.2e-03
   7
     1   0   0   1   1
      0.9165410533    1.0000000000
     2   0   0   1   1
      1.4278985792    1.0000000000
     3   0   0   1   1
      2.2042490497    1.0000000000
     4   0   0   1   1
      3.4333033407    1.0000000000
     5   0   0   1   1
      5.3971423888    1.0000000000
     6   1   1   1   1
      0.5144405644    1.0000000000
     7   1   1   1   1
      1.5558271972    1.0000000000

# RI basis set for C (all-electron) relative DI metric: 7.2e-04
C  RI_aug-SZV-MOLOPT-ae-mini_N_RI_017_s_p_d_f_g_h_i_6_2_1_0_0_0_0_error_7.2e-04
   9
     1   0   0   1   1
      0.4616654402    1.0000000000
     2   0   0   1   1
      0.7017742317    1.0000000000
     3   0   0   1   1
      1.0817323319    1.0000000000
     4   0   0   1   1
      1.6888472776    1.0000000000
     5   0   0   1   1
      2.6403290997    1.0000000000
     6   0   0   1   1
      4.1302286700    1.0000000000
     7   1   1   1   1
      0.4113237826    1.0000000000
     8   1   1   1   1
      1.1163282358    1.0000000000
     9   2   2   1   1
      2.8337941285    1.0000000000

# RI basis set for C (all-electron) relative DI metric: 1.4e-06
C  RI_aug-SZV-MOLOPT-ae-mini_N_RI_022_s_p_d_f_g_h_i_6_2_2_0_0_0_0_error_1.4e-06
   10
     1   0   0   1   1
      0.3573036368    1.0000000000
     2   0   0   1   1
      0.5859195274    1.0000000000
     3   0   0   1   1
      0.9625494398    1.0000000000
     4   0   0   1   1
      1.5830931274    1.0000000000
     5   0   0   1   1
      2.6028272414    1.0000000000
     6   0   0   1   1
      4.2787846013    1.0000000000
     7   1   1   1   1
      0.4130399935    1.0000000000
     8   1   1   1   1
      1.1752889367    1.0000000000
     9   2   2   1   1
      0.3194886190    1.0000000000
```

```
    10   2   2   1   1
      1.9687565083    1.0000000000

# RI basis set for C (all-electron) relative DI metric: 6.8e-07
C  RI_aug-SZV-MOLOPT-ae-mini_N_RI_025_s_p_d_f_g_h_i_6_3_2_0_0_0_0_error_6.8e-07
   11
     1   0   0   1   1
      0.2865589661    1.0000000000
     2   0   0   1   1
      0.5054104333    1.0000000000
     3   0   0   1   1
      0.8917487424    1.0000000000
     4   0   0   1   1
      1.5739361979    1.0000000000
     5   0   0   1   1
      2.7775633148    1.0000000000
     6   0   0   1   1
      4.9013678368    1.0000000000
     7   1   1   1   1
      0.4277251216    1.0000000000
     8   1   1   1   1
      1.0549895554    1.0000000000
     9   1   1   1   1
      4.5821662285    1.0000000000
    10   2   2   1   1
      0.3092837680    1.0000000000
    11   2   2   1   1
      1.9084499044    1.0000000000

# RI basis set for C (all-electron) relative DI metric: 2.1e-07
C  RI_aug-SZV-MOLOPT-ae-mini_N_RI_040_s_p_d_f_g_h_i_7_6_3_0_0_0_0_error_2.1e-07
   16
     1   0   0   1   1
      0.2864002389    1.0000000000
     2   0   0   1   1
      0.4597640659    1.0000000000
     3   0   0   1   1
      0.7380803313    1.0000000000
     4   0   0   1   1
      1.1848770535    1.0000000000
     5   0   0   1   1
      1.9021383792    1.0000000000
     6   0   0   1   1
      3.0535906080    1.0000000000
     7   0   0   1   1
      4.9020648570    1.0000000000
     8   1   1   1   1
      0.2863950499    1.0000000000
     9   1   1   1   1
      0.5054152627    1.0000000000
    10   1   1   1   1
      0.8919313814    1.0000000000
    11   1   1   1   1
      1.5740353863    1.0000000000
    12   1   1   1   1
      2.7777779108    1.0000000000
    13   1   1   1   1
      4.9020816667    1.0000000000
    14   2   2   1   1
      0.3266817287    1.0000000000
    15   2   2   1   1
      1.3251221295    1.0000000000
    16   2   2   1   1
      4.6198641560    1.0000000000

# RI basis set for C (all-electron) relative DI metric: 1.3e-10
C  RI_aug-SZV-MOLOPT-ae-mini_N_RI_050_s_p_d_f_g_h_i_7_6_5_0_0_0_0_error_1.3e-10
   18
     1   0   0   1   1
      0.2863950000    1.0000000000
     2   0   0   1   1
      0.4597636339    1.0000000000
     3   0   0   1   1
      0.7380806195    1.0000000000
     4   0   0   1   1
      1.1848762294    1.0000000000
     5   0   0   1   1
      1.9021386578    1.0000000000
     6   0   0   1   1
      3.0535944462    1.0000000000
     7   0   0   1   1
      4.9020816667    1.0000000000
     8   1   1   1   1
      0.2863950000    1.0000000000
     9   1   1   1   1
      0.5054153802    1.0000000000
    10   1   1   1   1
      0.8919314462    1.0000000000
    11   1   1   1   1
      1.5740354092    1.0000000000
    12   1   1   1   1
      2.7777779108    1.0000000000
    13   1   1   1   1
      4.9020816667    1.0000000000
    14   2   2   1   1
      0.2863950000    1.0000000000
    15   2   2   1   1
      0.5825312247    1.0000000000
    16   2   2   1   1
      1.1848762294    1.0000000000
    17   2   2   1   1
      2.4100539499    1.0000000000
    18   2   2   1   1
      4.9020816667    1.0000000000

# RI basis set for C (all-electron) relative DI metric: 2.0e-11
C  RI_aug-SZV-MOLOPT-ae-mini_N_RI_055_s_p_d_f_g_h_i_7_6_6_0_0_0_0_error_2.0e-11
   19
     1   0   0   1   1
      0.2863950000    1.0000000000
     2   0   0   1   1
```

```
      0.4597636339    1.0000000000
     3   0   0   1   1
      0.7380806195    1.0000000000
     4   0   0   1   1
      1.1848762294    1.0000000000
     5   0   0   1   1
      1.9021386578    1.0000000000
     6   0   0   1   1
      3.0535944462    1.0000000000
     7   0   0   1   1
      4.9020816667    1.0000000000
     8   1   1   1   1
      0.2863950000    1.0000000000
     9   1   1   1   1
      0.5054153802    1.0000000000
    10   1   1   1   1
      0.8919314462    1.0000000000
    11   1   1   1   1
      1.5740354092    1.0000000000
    12   1   1   1   1
      2.7777779108    1.0000000000
    13   1   1   1   1
      4.9020816667    1.0000000000
    14   2   2   1   1
      0.2863950000    1.0000000000
    15   2   2   1   1
      0.5054153802    1.0000000000
    16   2   2   1   1
      0.8919314462    1.0000000000
    17   2   2   1   1
      1.5740354092    1.0000000000
    18   2   2   1   1
      2.7777779108    1.0000000000
    19   2   2   1   1
      4.9020816667    1.0000000000

# RI basis set for C (all-electron) relative DI metric: 1.2e-02
C  RI_aug-SZV-MOLOPT-ae-SR_N_RI_010_s_p_d_f_g_h_i_2_1_1_0_0_0_0_error_1.2e-02
   4
     1   0   0   1   1
      2.8934416376    1.0000000000
     2   0   0   1   1
      8.9886817802    1.0000000000
     3   1   1   1   1
      0.8321629877    1.0000000000
     4   2   2   1   1
      0.7411049496    1.0000000000

# RI basis set for C (all-electron) relative DI metric: 3.2e-03
C  RI_aug-SZV-MOLOPT-ae-SR_N_RI_018_s_p_d_f_g_h_i_4_3_1_0_0_0_0_error_3.2e-03
   8
     1   0   0   1   1
      0.3217228640    1.0000000000
     2   0   0   1   1
      0.8553597430    1.0000000000
     3   0   0   1   1
      8.3280146579    1.0000000000
     4   0   0   1   1
     81.3804831356    1.0000000000
     5   1   1   1   1
      1.0347513413    1.0000000000
     6   1   1   1   1
      2.1392175445    1.0000000000
     7   1   1   1   1
      4.5792995891    1.0000000000
     8   2   2   1   1
      0.5456040832    1.0000000000

# RI basis set for C (all-electron) relative DI metric: 1.0e-04
C  RI_aug-SZV-MOLOPT-ae-SR_N_RI_029_s_p_d_f_g_h_i_5_4_1_1_0_0_0_error_1.0e-04
   11
     1   0   0   1   1
      0.2759929116    1.0000000000
     2   0   0   1   1
      1.0923842149    1.0000000000
     3   0   0   1   1
      4.9019866910    1.0000000000
     4   0   0   1   1
     20.2730739459    1.0000000000
     5   0   0   1   1
     80.8351403533    1.0000000000
     6   1   1   1   1
      0.6587945534    1.0000000000
     7   1   1   1   1
      2.3331948867    1.0000000000
     8   1   1   1   1
      6.3481850853    1.0000000000
     9   1   1   1   1
     17.6378720562    1.0000000000
    10   2   2   1   1
      0.5873038702    1.0000000000
    11   3   3   1   1
      1.0356515402    1.0000000000

# RI basis set for C (all-electron) relative DI metric: 3.3e-05
C  RI_aug-SZV-MOLOPT-ae-SR_N_RI_045_s_p_d_f_g_h_i_6_5_2_2_0_0_0_error_3.3e-05
   15
     1   0   0   1   1
      0.3356260130    1.0000000000
     2   0   0   1   1
      1.0121926865    1.0000000000
     3   0   0   1   1
      3.1155677619    1.0000000000
     4   0   0   1   1
      9.2320465028    1.0000000000
     5   0   0   1   1
     27.1304876148    1.0000000000
     6   0   0   1   1
     79.5971566998    1.0000000000
     7   1   1   1   1
      0.4304308067    1.0000000000
```

```
     8   1   1   1   1
      0.5579024388    1.0000000000
     9   1   1   1   1
      1.5959551455    1.0000000000
    10   1   1   1   1
      5.0691126601    1.0000000000
    11   1   1   1   1
     15.1997043408    1.0000000000
    12   2   2   1   1
      0.5220831846    1.0000000000
    13   2   2   1   1
      2.5228041102    1.0000000000
    14   3   3   1   1
      0.5883496293    1.0000000000
    15   3   3   1   1
      2.5197345063    1.0000000000

# RI basis set for C (all-electron) relative DI metric: 9.8e-06
C  RI_aug-SZV-MOLOPT-ae-SR_N_RI_054_s_p_d_f_g_h_i_6_5_2_2_1_0_0_error_9.8e-06
   16
     1   0   0   1   1
      0.3149722647    1.0000000000
     2   0   0   1   1
      0.9498328706    1.0000000000
     3   0   0   1   1
      2.3588023813    1.0000000000
     4   0   0   1   1
      7.6219014679    1.0000000000
     5   0   0   1   1
     25.0528864944    1.0000000000
     6   0   0   1   1
     80.2666991396    1.0000000000
     7   1   1   1   1
      0.2803225733    1.0000000000
     8   1   1   1   1
      0.4728186015    1.0000000000
     9   1   1   1   1
      1.4360019609    1.0000000000
    10   1   1   1   1
      5.0091423988    1.0000000000
    11   1   1   1   1
     16.4123995279    1.0000000000
    12   2   2   1   1
      0.4638938776    1.0000000000
    13   2   2   1   1
      2.6350441175    1.0000000000
    14   3   3   1   1
      0.6520959885    1.0000000000
    15   3   3   1   1
      2.3460470961    1.0000000000
    16   4   4   1   1
      1.1084873393    1.0000000000

# RI basis set for C (all-electron) relative DI metric: 9.1e-07
C  RI_aug-SZV-MOLOPT-ae-SR_N_RI_075_s_p_d_f_g_h_i_6_5_3_3_2_0_0_error_9.1e-07
   19
     1   0   0   1   1
      0.3069938763    1.0000000000
     2   0   0   1   1
      0.9468618000    1.0000000000
     3   0   0   1   1
      1.2688573114    1.0000000000
     4   0   0   1   1
      4.9251778562    1.0000000000
     5   0   0   1   1
     21.6436483878    1.0000000000
     6   0   0   1   1
     78.5004035044    1.0000000000
     7   1   1   1   1
      0.2807876683    1.0000000000
     8   1   1   1   1
      0.4776988648    1.0000000000
     9   1   1   1   1
      1.3998003965    1.0000000000
    10   1   1   1   1
      4.8178395166    1.0000000000
    11   1   1   1   1
     16.2028897637    1.0000000000
    12   2   2   1   1
      0.4258849991    1.0000000000
    13   2   2   1   1
      1.2882452526    1.0000000000
    14   2   2   1   1
      2.3353791373    1.0000000000
    15   3   3   1   1
      0.4414571449    1.0000000000
    16   3   3   1   1
      0.9016876340    1.0000000000
    17   3   3   1   1
      2.5321100326    1.0000000000
    18   4   4   1   1
      0.9344096601    1.0000000000
    19   4   4   1   1
      1.5355888317    1.0000000000

# RI basis set for C (all-electron) relative DI metric: 1.1e-02
C  RI_aug-SZV-MOLOPT-ae_N_RI_010_s_p_d_f_g_h_i_2_1_1_0_0_0_0_error_1.1e-02
   4
     1   0   0   1   1
      2.6191396176    1.0000000000
     2   0   0   1   1
     13.3812594461    1.0000000000
     3   1   1   1   1
      0.8555145853    1.0000000000
     4   2   2   1   1
      0.6928828507    1.0000000000

# RI basis set for C (all-electron) relative DI metric: 6.7e-04
C  RI_aug-SZV-MOLOPT-ae_N_RI_023_s_p_d_f_g_h_i_5_2_1_1_0_0_0_error_6.7e-04
   9
     1   0   0   1   1
```

```
      0.2056033452    1.0000000000
     2   0   0   1   1
      0.8049620781    1.0000000000
     3   0   0   1   1
      4.6057255680    1.0000000000
     4   0   0   1   1
     20.1691260708    1.0000000000
     5   0   0   1   1
     81.6618981877    1.0000000000
     6   1   1   1   1
      0.2376347921    1.0000000000
     7   1   1   1   1
      1.0237565019    1.0000000000
     8   2   2   1   1
      0.5817360740    1.0000000000
     9   3   3   1   1
      1.1168326622    1.0000000000

# RI basis set for C (all-electron) relative DI metric: 1.7e-04
C  RI_aug-SZV-MOLOPT-ae_N_RI_047_s_p_d_f_g_h_i_5_3_2_2_1_0_0_error_1.7e-04
   13
     1   0   0   1   1
      0.2159960852    1.0000000000
     2   0   0   1   1
      0.5588459320    1.0000000000
     3   0   0   1   1
      3.1133491855    1.0000000000
     4   0   0   1   1
     16.0358596352    1.0000000000
     5   0   0   1   1
     82.3413151158    1.0000000000
     6   1   1   1   1
      0.1880256744    1.0000000000
     7   1   1   1   1
      0.8850463414    1.0000000000
     8   1   1   1   1
     15.6318792293    1.0000000000
     9   2   2   1   1
      0.4931594824    1.0000000000
    10   2   2   1   1
      3.1394161483    1.0000000000
    11   3   3   1   1
      0.6068637805    1.0000000000
    12   3   3   1   1
      2.6402812005    1.0000000000
    13   4   4   1   1
      1.0891285069    1.0000000000

# RI basis set for C (all-electron) relative DI metric: 8.4e-05
C  RI_aug-SZV-MOLOPT-ae_N_RI_048_s_p_d_f_g_h_i_6_3_2_2_1_0_0_error_8.4e-05
   14
     1   0   0   1   1
      0.1821321734    1.0000000000
     2   0   0   1   1
      0.4339049118    1.0000000000
     3   0   0   1   1
      1.6206925128    1.0000000000
     4   0   0   1   1
      5.9996611038    1.0000000000
     5   0   0   1   1
     22.2249632951    1.0000000000
     6   0   0   1   1
     82.3297694046    1.0000000000
     7   1   1   1   1
      0.2135936218    1.0000000000
     8   1   1   1   1
      0.9158565360    1.0000000000
     9   1   1   1   1
      9.2848609404    1.0000000000
    10   2   2   1   1
      0.3811247059    1.0000000000
    11   2   2   1   1
      1.5814884974    1.0000000000
    12   3   3   1   1
      0.5244458254    1.0000000000
    13   3   3   1   1
      2.6771569161    1.0000000000
    14   4   4   1   1
      1.2176655648    1.0000000000

# RI basis set for C (all-electron) relative DI metric: 3.9e-02
C  RI_aug-DZVP-MOLOPT-ae_N_RI_010_s_p_d_f_g_h_i_2_1_1_0_0_0_0_error_3.9e-02
   4
     1   0   0   1   1
      3.5834788500    1.0000000000
     2   0   0   1   1
      8.3106857686    1.0000000000
     3   1   1   1   1
      0.8997380223    1.0000000000
     4   2   2   1   1
      1.0578164454    1.0000000000

# RI basis set for C (all-electron) relative DI metric: 9.3e-03
C  RI_aug-DZVP-MOLOPT-ae_N_RI_018_s_p_d_f_g_h_i_4_3_1_0_0_0_0_error_9.3e-03
   8
     1   0   0   1   1
      0.2711703797    1.0000000000
     2   0   0   1   1
      1.5223072417    1.0000000000
     3   0   0   1   1
     14.5066017156    1.0000000000
     4   0   0   1   1
    102.3848656322    1.0000000000
     5   1   1   1   1
      0.2265737154    1.0000000000
     6   1   1   1   1
      1.6588365523    1.0000000000
     7   1   1   1   1
     23.8479103620    1.0000000000
     8   2   2   1   1
      1.0207681204    1.0000000000
```

```
# RI basis set for C (all-electron) relative DI metric: 2.6e-03
C  RI_aug-DZVP-MOLOPT-ae_N_RI_029_s_p_d_f_g_h_i_5_4_1_1_0_0_0_error_2.6e-03
   11
    1   0   0   1   1
     0.0733635045    1.0000000000
    2   0   0   1   1
     0.4365138690    1.0000000000
    3   0   0   1   1
     2.2504763586    1.0000000000
    4   0   0   1   1
    11.7965818644    1.0000000000
    5   0   0   1   1
    68.6282556583    1.0000000000
    6   1   1   1   1
     0.1825549481    1.0000000000
    7   1   1   1   1
     0.7011234039    1.0000000000
    8   1   1   1   1
     3.0030147576    1.0000000000
    9   1   1   1   1
    13.6375596795    1.0000000000
   10   2   2   1   1
     0.9597351493    1.0000000000
   11   3   3   1   1
     1.0420807849    1.0000000000

# RI basis set for C (all-electron) relative DI metric: 4.2e-04
C  RI_aug-DZVP-MOLOPT-ae_N_RI_035_s_p_d_f_g_h_i_6_4_2_1_0_0_0_error_4.2e-04
   13
    1   0   0   1   1
     0.1345134311    1.0000000000
    2   0   0   1   1
     0.3916635522    1.0000000000
    3   0   0   1   1
     1.4042738635    1.0000000000
    4   0   0   1   1
     5.6058181254    1.0000000000
    5   0   0   1   1
    21.9005336645    1.0000000000
    6   0   0   1   1
    82.0221874732    1.0000000000
    7   1   1   1   1
     0.3453422395    1.0000000000
    8   1   1   1   1
     0.7276422870    1.0000000000
    9   1   1   1   1
     3.0218755068    1.0000000000
   10   1   1   1   1
    16.0345515256    1.0000000000
   11   2   2   1   1
     0.4364735168    1.0000000000
   12   2   2   1   1
     1.3304225057    1.0000000000
   13   3   3   1   1
     0.9496592034    1.0000000000

# RI basis set for C (all-electron) relative DI metric: 1.2e-04
C  RI_aug-DZVP-MOLOPT-ae_N_RI_047_s_p_d_f_g_h_i_6_4_3_2_0_0_0_error_1.2e-04
   15
    1   0   0   1   1
     0.2548416823    1.0000000000
    2   0   0   1   1
     0.4225888673    1.0000000000
    3   0   0   1   1
     1.2992930855    1.0000000000
    4   0   0   1   1
     5.6847231578    1.0000000000
    5   0   0   1   1
    22.0161608401    1.0000000000
    6   0   0   1   1
    82.1039673190    1.0000000000
    7   1   1   1   1
     0.2993248895    1.0000000000
    8   1   1   1   1
     0.9395696949    1.0000000000
    9   1   1   1   1
     3.3571598066    1.0000000000
   10   1   1   1   1
    16.3652385615    1.0000000000
   11   2   2   1   1
     0.2819367454    1.0000000000
   12   2   2   1   1
     0.8119306759    1.0000000000
   13   2   2   1   1
     2.9312130473    1.0000000000
   14   3   3   1   1
     0.3398155817    1.0000000000
   15   3   3   1   1
     1.3511942136    1.0000000000

# RI basis set for C (all-electron) relative DI metric: 4.4e-05
C  RI_aug-DZVP-MOLOPT-ae_N_RI_060_s_p_d_f_g_h_i_7_5_3_2_1_0_0_error_4.4e-05
   18
    1   0   0   1   1
     0.1390291015    1.0000000000
    2   0   0   1   1
     0.3509347299    1.0000000000
    3   0   0   1   1
     1.0271052919    1.0000000000
    4   0   0   1   1
     3.1151077202    1.0000000000
    5   0   0   1   1
     9.2926918292    1.0000000000
    6   0   0   1   1
    27.6625493769    1.0000000000
    7   0   0   1   1
    82.3450417359    1.0000000000
    8   1   1   1   1
     0.2637372023    1.0000000000
    9   1   1   1   1
```

```
      0.7115755304    1.0000000000
    10   1   1   1   1
      1.8135481674    1.0000000000
    11   1   1   1   1
      5.5722279923    1.0000000000
    12   1   1   1   1
     16.9742997541    1.0000000000
    13   2   2   1   1
      0.2607196960    1.0000000000
    14   2   2   1   1
      0.7220373754    1.0000000000
    15   2   2   1   1
      2.0606421767    1.0000000000
    16   3   3   1   1
      0.2950192530    1.0000000000
    17   3   3   1   1
      1.2286389294    1.0000000000
    18   4   4   1   1
      0.7351949451    1.0000000000

# RI basis set for C (all-electron) relative DI metric: 8.9e-06
C  RI_aug-DZVP-MOLOPT-ae_N_RI_067_s_p_d_f_g_h_i_7_5_3_3_1_0_0_error_8.9e-06
   19
     1   0   0   1   1
      0.1392308281    1.0000000000
     2   0   0   1   1
      0.3594750654    1.0000000000
     3   0   0   1   1
      1.0490847875    1.0000000000
     4   0   0   1   1
      3.1159061908    1.0000000000
     5   0   0   1   1
      9.2968462872    1.0000000000
     6   0   0   1   1
     27.6818435712    1.0000000000
     7   0   0   1   1
     82.3646151150    1.0000000000
     8   1   1   1   1
      0.1841999814    1.0000000000
     9   1   1   1   1
      0.5435907574    1.0000000000
    10   1   1   1   1
      1.4445226480    1.0000000000
    11   1   1   1   1
      5.4334740731    1.0000000000
    12   1   1   1   1
     16.9478360702    1.0000000000
    13   2   2   1   1
      0.2245613307    1.0000000000
    14   2   2   1   1
      0.6869642339    1.0000000000
    15   2   2   1   1
      1.9876949896    1.0000000000
    16   3   3   1   1
      0.2549923466    1.0000000000
    17   3   3   1   1
      0.8369349474    1.0000000000
    18   3   3   1   1
      2.5905900447    1.0000000000
    19   4   4   1   1
      0.2503300668    1.0000000000

# RI basis set for C (all-electron) relative DI metric: 1.4e-06
C  RI_aug-DZVP-MOLOPT-ae_N_RI_077_s_p_d_f_g_h_i_7_5_5_3_1_0_0_error_1.4e-06
   21
     1   0   0   1   1
      0.1955832419    1.0000000000
     2   0   0   1   1
      0.3052133491    1.0000000000
     3   0   0   1   1
      0.9119275529    1.0000000000
     4   0   0   1   1
      3.0250187367    1.0000000000
     5   0   0   1   1
      9.1842143255    1.0000000000
     6   0   0   1   1
     27.5333685150    1.0000000000
     7   0   0   1   1
     82.2125736520    1.0000000000
     8   1   1   1   1
      0.2070043555    1.0000000000
     9   1   1   1   1
      0.4914244786    1.0000000000
    10   1   1   1   1
      1.2856283304    1.0000000000
    11   1   1   1   1
      5.2297462358    1.0000000000
    12   1   1   1   1
     16.5076285357    1.0000000000
    13   2   2   1   1
      0.2796186251    1.0000000000
    14   2   2   1   1
      0.4629816850    1.0000000000
    15   2   2   1   1
      0.9633760652    1.0000000000
    16   2   2   1   1
      1.9751437949    1.0000000000
    17   2   2   1   1
      4.3178639365    1.0000000000
    18   3   3   1   1
      0.2869154674    1.0000000000
    19   3   3   1   1
      0.7525257679    1.0000000000
    20   3   3   1   1
      2.2791356262    1.0000000000
    21   4   4   1   1
      0.2837293445    1.0000000000

# RI basis set for C (all-electron) relative DI metric: 6.7e-07
C  RI_aug-DZVP-MOLOPT-ae_N_RI_102_s_p_d_f_g_h_i_7_5_5_4_3_0_0_error_6.7e-07
   24
```

```
     1   0   0   1   1
      0.1767727781    1.0000000000
     2   0   0   1   1
      0.2827685508    1.0000000000
     3   0   0   1   1
      0.8566204523    1.0000000000
     4   0   0   1   1
      2.9871605018    1.0000000000
     5   0   0   1   1
      9.1588633448    1.0000000000
     6   0   0   1   1
     27.4926875641    1.0000000000
     7   0   0   1   1
     82.1681561206    1.0000000000
     8   1   1   1   1
      0.1968071375    1.0000000000
     9   1   1   1   1
      0.4781569745    1.0000000000
    10   1   1   1   1
      1.1628213623    1.0000000000
    11   1   1   1   1
      4.4736836509    1.0000000000
    12   1   1   1   1
     16.1955118487    1.0000000000
    13   2   2   1   1
      0.3101728219    1.0000000000
    14   2   2   1   1
      0.4755210547    1.0000000000
    15   2   2   1   1
      0.9607454810    1.0000000000
    16   2   2   1   1
      1.9215223724    1.0000000000
    17   2   2   1   1
      4.3452027987    1.0000000000
    18   3   3   1   1
      0.2389632979    1.0000000000
    19   3   3   1   1
      0.6159237674    1.0000000000
    20   3   3   1   1
      1.2146585960    1.0000000000
    21   3   3   1   1
      2.3777185464    1.0000000000
    22   4   4   1   1
      0.4154542817    1.0000000000
    23   4   4   1   1
      0.7741176275    1.0000000000
    24   4   4   1   1
      1.3562318230    1.0000000000

# RI basis set for C (all-electron) relative DI metric: 3.4e-02
C  RI_aug-TZVP-MOLOPT-ae_N_RI_018_s_p_d_f_g_h_i_3_1_1_1_0_0_0_error_3.4e-02
   6
     1   0   0   1   1
      3.1502690553    1.0000000000
     2   0   0   1   1
      7.6484813809    1.0000000000
     3   0   0   1   1
     18.9617076378    1.0000000000
     4   1   1   1   1
      0.8847028649    1.0000000000
     5   2   2   1   1
      1.1047708862    1.0000000000
     6   3   3   1   1
      1.3318123137    1.0000000000

# RI basis set for C (all-electron) relative DI metric: 1.2e-02
C  RI_aug-TZVP-MOLOPT-ae_N_RI_022_s_p_d_f_g_h_i_4_2_1_1_0_0_0_error_1.2e-02
   8
     1   0   0   1   1
      0.3146504172    1.0000000000
     2   0   0   1   1
      1.8480009838    1.0000000000
     3   0   0   1   1
     10.8544133630    1.0000000000
     4   0   0   1   1
     63.7534153533    1.0000000000
     5   1   1   1   1
      0.6532518785    1.0000000000
     6   1   1   1   1
      1.4132944048    1.0000000000
     7   2   2   1   1
      1.0394040421    1.0000000000
     8   3   3   1   1
      1.1497038199    1.0000000000

# RI basis set for C (all-electron) relative DI metric: 4.6e-03
C  RI_aug-TZVP-MOLOPT-ae_N_RI_029_s_p_d_f_g_h_i_6_2_2_1_0_0_0_error_4.6e-03
   11
     1   0   0   1   1
      0.0922372318    1.0000000000
     2   0   0   1   1
      0.4332285265    1.0000000000
     3   0   0   1   1
      1.5881140243    1.0000000000
     4   0   0   1   1
      5.8139558174    1.0000000000
     5   0   0   1   1
     21.9618430614    1.0000000000
     6   0   0   1   1
     82.0218695250    1.0000000000
     7   1   1   1   1
      0.6422257434    1.0000000000
     8   1   1   1   1
      1.2971227977    1.0000000000
     9   2   2   1   1
      0.7931472045    1.0000000000
    10   2   2   1   1
      2.6128011914    1.0000000000
    11   3   3   1   1
      1.0957881873    1.0000000000
```

```
# RI basis set for C (all-electron) relative DI metric: 2.3e-03
C  RI_aug-TZVP-MOLOPT-ae_N_RI_041_s_p_d_f_g_h_i_6_3_2_1_1_0_0_error_2.3e-03
  13
    1   0   0   1   1
     0.1301411497    1.0000000000
    2   0   0   1   1
     0.4143152929    1.0000000000
    3   0   0   1   1
     1.4140293514    1.0000000000
    4   0   0   1   1
     5.5423831131    1.0000000000
    5   0   0   1   1
    21.6007934991    1.0000000000
    6   0   0   1   1
    81.5907587316    1.0000000000
    7   1   1   1   1
     0.5844974760    1.0000000000
    8   1   1   1   1
     1.2231289523    1.0000000000
    9   1   1   1   1
    11.9736958952    1.0000000000
   10   2   2   1   1
     0.7847099972    1.0000000000
   11   2   2   1   1
     2.2270150443    1.0000000000
   12   3   3   1   1
     1.1041613775    1.0000000000
   13   4   4   1   1
     1.1590919683    1.0000000000

# RI basis set for C (all-electron) relative DI metric: 6.0e-04
C  RI_aug-TZVP-MOLOPT-ae_N_RI_048_s_p_d_f_g_h_i_6_3_2_2_1_0_0_error_6.0e-04
  14
    1   0   0   1   1
     0.2456758592    1.0000000000
    2   0   0   1   1
     0.4244099874    1.0000000000
    3   0   0   1   1
     1.2409992889    1.0000000000
    4   0   0   1   1
     5.1873610724    1.0000000000
    5   0   0   1   1
    21.0862483820    1.0000000000
    6   0   0   1   1
    80.9594150705    1.0000000000
    7   1   1   1   1
     0.5229370070    1.0000000000
    8   1   1   1   1
     1.7293501734    1.0000000000
    9   1   1   1   1
    10.1113416740    1.0000000000
   10   2   2   1   1
     0.6850969105    1.0000000000
   11   2   2   1   1
     2.0071949515    1.0000000000
   12   3   3   1   1
     0.5522346945    1.0000000000
   13   3   3   1   1
     1.8297097626    1.0000000000
   14   4   4   1   1
     1.0099496878    1.0000000000

# RI basis set for C (all-electron) relative DI metric: 2.8e-04
C  RI_aug-TZVP-MOLOPT-ae_N_RI_051_s_p_d_f_g_h_i_6_4_2_2_1_0_0_error_2.8e-04
  15
    1   0   0   1   1
     0.2393535841    1.0000000000
    2   0   0   1   1
     0.4775796096    1.0000000000
    3   0   0   1   1
     1.4795968593    1.0000000000
    4   0   0   1   1
     5.6948037427    1.0000000000
    5   0   0   1   1
    21.8200938338    1.0000000000
    6   0   0   1   1
    81.8483680819    1.0000000000
    7   1   1   1   1
     0.4572971321    1.0000000000
    8   1   1   1   1
     1.0703773320    1.0000000000
    9   1   1   1   1
     3.2149656981    1.0000000000
   10   1   1   1   1
    16.2979482614    1.0000000000
   11   2   2   1   1
     0.6858802217    1.0000000000
   12   2   2   1   1
     1.9650556217    1.0000000000
   13   3   3   1   1
     0.6264377365    1.0000000000
   14   3   3   1   1
     1.6115990986    1.0000000000
   15   4   4   1   1
     1.0062094734    1.0000000000

# RI basis set for C (all-electron) relative DI metric: 1.0e-04
C  RI_aug-TZVP-MOLOPT-ae_N_RI_065_s_p_d_f_g_h_i_6_4_3_2_2_0_0_error_1.0e-04
  17
    1   0   0   1   1
     0.2553243297    1.0000000000
    2   0   0   1   1
     0.5190956392    1.0000000000
    3   0   0   1   1
     1.3854298055    1.0000000000
    4   0   0   1   1
     5.3861610044    1.0000000000
    5   0   0   1   1
    21.3951993307    1.0000000000
    6   0   0   1   1
    81.3672493566    1.0000000000
```

```
      7   1   1   1   1
       0.3283794717    1.0000000000
      8   1   1   1   1
       0.8032542751    1.0000000000
      9   1   1   1   1
       2.7016325675    1.0000000000
     10   1   1   1   1
      13.5533669435    1.0000000000
     11   2   2   1   1
       0.4344577581    1.0000000000
     12   2   2   1   1
       1.0554467672    1.0000000000
     13   2   2   1   1
       3.6086562132    1.0000000000
     14   3   3   1   1
       0.5330765737    1.0000000000
     15   3   3   1   1
       1.5752779669    1.0000000000
     16   4   4   1   1
       0.6204974632    1.0000000000
     17   4   4   1   1
       1.5043818154    1.0000000000

# RI basis set for C (all-electron) relative DI metric: 4.5e-05
C  RI_aug-TZVP-MOLOPT-ae_N_RI_073_s_p_d_f_g_h_i_7_4_3_3_2_0_0_error_4.5e-05
   19
      1   0   0   1   1
       0.0918159897    1.0000000000
      2   0   0   1   1
       0.2871747535    1.0000000000
      3   0   0   1   1
       0.8110529957    1.0000000000
      4   0   0   1   1
       2.6310511956    1.0000000000
      5   0   0   1   1
       8.6342127738    1.0000000000
      6   0   0   1   1
      27.1352682871    1.0000000000
      7   0   0   1   1
      81.8481250191    1.0000000000
      8   1   1   1   1
       0.3914546745    1.0000000000
      9   1   1   1   1
       0.7926471525    1.0000000000
     10   1   1   1   1
       2.7357356707    1.0000000000
     11   1   1   1   1
      14.1328788527    1.0000000000
     12   2   2   1   1
       0.4531991776    1.0000000000
     13   2   2   1   1
       1.0508314697    1.0000000000
     14   2   2   1   1
       3.5314239833    1.0000000000
     15   3   3   1   1
       0.4935957141    1.0000000000
     16   3   3   1   1
       1.0944165437    1.0000000000
     17   3   3   1   1
       2.2479699281    1.0000000000
     18   4   4   1   1
       0.5526779684    1.0000000000
     19   4   4   1   1
       1.6172510149    1.0000000000

# RI basis set for C (all-electron) relative DI metric: 2.0e-05
C  RI_aug-TZVP-MOLOPT-ae_N_RI_076_s_p_d_f_g_h_i_7_5_3_3_2_0_0_error_2.0e-05
   20
      1   0   0   1   1
       0.2921370058    1.0000000000
      2   0   0   1   1
       0.6801182066    1.0000000000
      3   0   0   1   1
       1.4987697876    1.0000000000
      4   0   0   1   1
       4.0284301435    1.0000000000
      5   0   0   1   1
      11.1650300514    1.0000000000
      6   0   0   1   1
      30.3522867455    1.0000000000
      7   0   0   1   1
      81.6709361233    1.0000000000
      8   1   1   1   1
       0.3930677715    1.0000000000
      9   1   1   1   1
       0.6102702938    1.0000000000
     10   1   1   1   1
       1.7527838356    1.0000000000
     11   1   1   1   1
       5.8743527677    1.0000000000
     12   1   1   1   1
      15.4414492584    1.0000000000
     13   2   2   1   1
       0.3924950599    1.0000000000
     14   2   2   1   1
       1.0472644196    1.0000000000
     15   2   2   1   1
       3.5391789208    1.0000000000
     16   3   3   1   1
       0.5282375045    1.0000000000
     17   3   3   1   1
       1.1503554926    1.0000000000
     18   3   3   1   1
       3.0803726175    1.0000000000
     19   4   4   1   1
       0.4850018364    1.0000000000
     20   4   4   1   1
       1.5606725484    1.0000000000

# RI basis set for C (all-electron) relative DI metric: 6.6e-06
C  RI_aug-TZVP-MOLOPT-ae_N_RI_084_s_p_d_f_g_h_i_7_6_4_3_2_0_0_error_6.6e-06
```

```
   22
     1   0   0   1   1
      0.1475763200    1.0000000000
     2   0   0   1   1
      0.2735512852    1.0000000000
     3   0   0   1   1
      0.7613873413    1.0000000000
     4   0   0   1   1
      2.3137161906    1.0000000000
     5   0   0   1   1
      7.8973936221    1.0000000000
     6   0   0   1   1
     26.7995019274    1.0000000000
     7   0   0   1   1
     81.4850808681    1.0000000000
     8   1   1   1   1
      0.3445629898    1.0000000000
     9   1   1   1   1
      0.4913651387    1.0000000000
    10   1   1   1   1
      1.1111614743    1.0000000000
    11   1   1   1   1
      2.8095573455    1.0000000000
    12   1   1   1   1
      7.0571881063    1.0000000000
    13   1   1   1   1
     17.0380536816    1.0000000000
    14   2   2   1   1
      0.2279443797    1.0000000000
    15   2   2   1   1
      0.4312587874    1.0000000000
    16   2   2   1   1
      1.2430028245    1.0000000000
    17   2   2   1   1
      3.7705245424    1.0000000000
    18   3   3   1   1
      0.5355551805    1.0000000000
    19   3   3   1   1
      1.3457995671    1.0000000000
    20   3   3   1   1
      3.7420977382    1.0000000000
    21   4   4   1   1
      0.5467181097    1.0000000000
    22   4   4   1   1
      1.7384751607    1.0000000000

# RI basis set for C (all-electron) relative DI metric: 3.2e-06
C  RI_aug-TZVP-MOLOPT-ae_N_RI_094_s_p_d_f_g_h_i_7_6_6_3_2_0_0_error_3.2e-06
   24
     1   0   0   1   1
      0.1823749974    1.0000000000
     2   0   0   1   1
      0.2786247028    1.0000000000
     3   0   0   1   1
      0.7533443142    1.0000000000
     4   0   0   1   1
      2.2472189038    1.0000000000
     5   0   0   1   1
      7.1637658207    1.0000000000
     6   0   0   1   1
     24.8066644242    1.0000000000
     7   0   0   1   1
     79.3320585465    1.0000000000
     8   1   1   1   1
      0.3505431095    1.0000000000
     9   1   1   1   1
      0.4925200015    1.0000000000
    10   1   1   1   1
      1.0634823891    1.0000000000
    11   1   1   1   1
      2.6824263758    1.0000000000
    12   1   1   1   1
      6.9909328723    1.0000000000
    13   1   1   1   1
     16.9666942531    1.0000000000
    14   2   2   1   1
      0.2161262158    1.0000000000
    15   2   2   1   1
      0.3873638660    1.0000000000
    16   2   2   1   1
      0.7151141494    1.0000000000
    17   2   2   1   1
      1.3134627766    1.0000000000
    18   2   2   1   1
      2.4175214579    1.0000000000
    19   2   2   1   1
      4.5730455738    1.0000000000
    20   3   3   1   1
      0.5308746655    1.0000000000
    21   3   3   1   1
      1.3208422005    1.0000000000
    22   3   3   1   1
      3.8822698538    1.0000000000
    23   4   4   1   1
      0.5473649493    1.0000000000
    24   4   4   1   1
      1.5483278866    1.0000000000

# RI basis set for N (all-electron) relative DI metric: 2.7e-02
N  RI_aug-SZV-MOLOPT-ae-mini_N_RI_006_s_p_d_f_g_h_i_3_1_0_0_0_0_0_error_2.7e-02
   4
     1   0   0   1   1
      0.9554107134    1.0000000000
     2   0   0   1   1
      1.6511801180    1.0000000000
     3   0   0   1   1
      2.8482258727    1.0000000000
     4   1   1   1   1
      4.0271620921    1.0000000000

# RI basis set for N (all-electron) relative DI metric: 6.9e-04
```

```
N  RI_aug-SZV-MOLOPT-ae-mini_N_RI_018_s_p_d_f_g_h_i_4_3_1_0_0_0_0_error_6.9e-04
   8
     1   0   0   1   1
      0.4093569870    1.0000000000
     2   0   0   1   1
      1.2514964904    1.0000000000
     3   0   0   1   1
      3.0013349844    1.0000000000
     4   0   0   1   1
      5.5611333166    1.0000000000
     5   1   1   1   1
      0.2443110997    1.0000000000
     6   1   1   1   1
      1.1895082980    1.0000000000
     7   1   1   1   1
      8.2457522637    1.0000000000
     8   2   2   1   1
      2.9780390367    1.0000000000

# RI basis set for N (all-electron) relative DI metric: 1.0e-06
N  RI_aug-SZV-MOLOPT-ae-mini_N_RI_023_s_p_d_f_g_h_i_4_3_2_0_0_0_0_error_1.0e-06
   9
     1   0   0   1   1
      0.2699823251    1.0000000000
     2   0   0   1   1
      0.7738294534    1.0000000000
     3   0   0   1   1
      2.2285298905    1.0000000000
     4   0   0   1   1
      6.2958666392    1.0000000000
     5   1   1   1   1
      0.2902924101    1.0000000000
     6   1   1   1   1
      1.3370551211    1.0000000000
     7   1   1   1   1
      6.2062418173    1.0000000000
     8   2   2   1   1
      0.2474730899    1.0000000000
     9   2   2   1   1
      2.3684737679    1.0000000000

# RI basis set for N (all-electron) relative DI metric: 2.8e-07
N  RI_aug-SZV-MOLOPT-ae-mini_N_RI_037_s_p_d_f_g_h_i_5_4_4_0_0_0_0_error_2.8e-07
   13
     1   0   0   1   1
      0.2800299976    1.0000000000
     2   0   0   1   1
      0.6098901574    1.0000000000
     3   0   0   1   1
      1.3283077039    1.0000000000
     4   0   0   1   1
      2.8929821689    1.0000000000
     5   0   0   1   1
      6.3007583322    1.0000000000
     6   1   1   1   1
      0.2800300019    1.0000000000
     7   1   1   1   1
      0.7905584489    1.0000000000
     8   1   1   1   1
      2.2318417556    1.0000000000
     9   1   1   1   1
      6.3007583319    1.0000000000
    10   2   2   1   1
      0.2800299987    1.0000000000
    11   2   2   1   1
      0.7905584420    1.0000000000
    12   2   2   1   1
      2.2318417829    1.0000000000
    13   2   2   1   1
      6.3007583275    1.0000000000

# RI basis set for N (all-electron) relative DI metric: 1.3e-07
N  RI_aug-SZV-MOLOPT-ae-mini_N_RI_038_s_p_d_f_g_h_i_6_4_4_0_0_0_0_error_1.3e-07
   14
     1   0   0   1   1
      0.2800299997    1.0000000000
     2   0   0   1   1
      0.5219659975    1.0000000000
     3   0   0   1   1
      0.9729261250    1.0000000000
     4   0   0   1   1
      1.8134998234    1.0000000000
     5   0   0   1   1
      3.3802994135    1.0000000000
     6   0   0   1   1
      6.3007583334    1.0000000000
     7   1   1   1   1
      0.2800300016    1.0000000000
     8   1   1   1   1
      0.7905584486    1.0000000000
     9   1   1   1   1
      2.2318417569    1.0000000000
    10   1   1   1   1
      6.3007583319    1.0000000000
    11   2   2   1   1
      0.2800299995    1.0000000000
    12   2   2   1   1
      0.7905584425    1.0000000000
    13   2   2   1   1
      2.2318417764    1.0000000000
    14   2   2   1   1
      6.3007583311    1.0000000000

# RI basis set for N (all-electron) relative DI metric: 3.0e-08
N  RI_aug-SZV-MOLOPT-ae-mini_N_RI_041_s_p_d_f_g_h_i_6_5_4_0_0_0_0_error_3.0e-08
   15
     1   0   0   1   1
      0.2800299997    1.0000000000
     2   0   0   1   1
      0.5219659976    1.0000000000
     3   0   0   1   1
```

```
      0.9729261250    1.0000000000
     4   0   0   1   1
      1.8134998234    1.0000000000
     5   0   0   1   1
      3.3802994135    1.0000000000
     6   0   0   1   1
      6.3007583335    1.0000000000
     7   1   1   1   1
      0.2800300000    1.0000000000
     8   1   1   1   1
      0.6098901592    1.0000000000
     9   1   1   1   1
      1.3283077039    1.0000000000
    10   1   1   1   1
      2.8929821697    1.0000000000
    11   1   1   1   1
      6.3007583333    1.0000000000
    12   2   2   1   1
      0.2800299995    1.0000000000
    13   2   2   1   1
      0.7905584425    1.0000000000
    14   2   2   1   1
      2.2318417764    1.0000000000
    15   2   2   1   1
      6.3007583311    1.0000000000

# RI basis set for N (all-electron) relative DI metric: 6.8e-09
N  RI_aug-SZV-MOLOPT-ae-mini_N_RI_047_s_p_d_f_g_h_i_7_5_5_0_0_0_0_error_6.8e-09
   17
     1   0   0   1   1
      0.2800300000    1.0000000000
     2   0   0   1   1
      0.4705104470    1.0000000000
     3   0   0   1   1
      0.7905584428    1.0000000000
     4   0   0   1   1
      1.3283077038    1.0000000000
     5   0   0   1   1
      2.2318417723    1.0000000000
     6   0   0   1   1
      3.7499727526    1.0000000000
     7   0   0   1   1
      6.3007583333    1.0000000000
     8   1   1   1   1
      0.2800300000    1.0000000000
     9   1   1   1   1
      0.6098901592    1.0000000000
    10   1   1   1   1
      1.3283077039    1.0000000000
    11   1   1   1   1
      2.8929821697    1.0000000000
    12   1   1   1   1
      6.3007583333    1.0000000000
    13   2   2   1   1
      0.2800300000    1.0000000000
    14   2   2   1   1
      0.6098901592    1.0000000000
    15   2   2   1   1
      1.3283077038    1.0000000000
    16   2   2   1   1
      2.8929821698    1.0000000000
    17   2   2   1   1
      6.3007583332    1.0000000000

# RI basis set for N (all-electron) relative DI metric: 4.8e-02
N  RI_aug-SZV-MOLOPT-ae-SR_N_RI_007_s_p_d_f_g_h_i_4_1_0_0_0_0_0_error_4.8e-02
   5
     1   0   0   1   1
      0.5437273984    1.0000000000
     2   0   0   1   1
      2.1724878395    1.0000000000
     3   0   0   1   1
     14.6903227432    1.0000000000
     4   0   0   1   1
     77.0699050271    1.0000000000
     5   1   1   1   1
      1.0160173719    1.0000000000

# RI basis set for N (all-electron) relative DI metric: 6.3e-03
N  RI_aug-SZV-MOLOPT-ae-SR_N_RI_012_s_p_d_f_g_h_i_4_1_1_0_0_0_0_error_6.3e-03
   6
     1   0   0   1   1
      0.4251430139    1.0000000000
     2   0   0   1   1
      1.6056707754    1.0000000000
     3   0   0   1   1
     12.9086620539    1.0000000000
     4   0   0   1   1
     90.6638704283    1.0000000000
     5   1   1   1   1
      1.1195764353    1.0000000000
     6   2   2   1   1
      0.7486022890    1.0000000000

# RI basis set for N (all-electron) relative DI metric: 2.0e-04
N  RI_aug-SZV-MOLOPT-ae-SR_N_RI_025_s_p_d_f_g_h_i_4_3_1_1_0_0_0_error_2.0e-04
   9
     1   0   0   1   1
      0.3660430277    1.0000000000
     2   0   0   1   1
      1.3385215588    1.0000000000
     3   0   0   1   1
     12.3219488493    1.0000000000
     4   0   0   1   1
    109.5981674344    1.0000000000
     5   1   1   1   1
      0.4387856332    1.0000000000
     6   1   1   1   1
      1.6380057544    1.0000000000
     7   1   1   1   1
     21.3475786772    1.0000000000
```

```
     8   2   2   1   1
      0.6717026775    1.0000000000
     9   3   3   1   1
      0.6870253810    1.0000000000

# RI basis set for N (all-electron) relative DI metric: 7.1e-05
N  RI_aug-SZV-MOLOPT-ae-SR_N_RI_036_s_p_d_f_g_h_i_5_3_3_1_0_0_0_error_7.1e-05
   12
     1   0   0   1   1
      0.3621141077    1.0000000000
     2   0   0   1   1
      0.6316342217    1.0000000000
     3   0   0   1   1
      2.9789466462    1.0000000000
     4   0   0   1   1
     18.3420516463    1.0000000000
     5   0   0   1   1
    106.3089583557    1.0000000000
     6   1   1   1   1
      0.4996964383    1.0000000000
     7   1   1   1   1
      0.8833959321    1.0000000000
     8   1   1   1   1
      4.4077456353    1.0000000000
     9   2   2   1   1
      0.5886591695    1.0000000000
    10   2   2   1   1
      1.1539459089    1.0000000000
    11   2   2   1   1
      5.6918834247    1.0000000000
    12   3   3   1   1
      0.7366054611    1.0000000000

# RI basis set for N (all-electron) relative DI metric: 1.9e-05
N  RI_aug-SZV-MOLOPT-ae-SR_N_RI_044_s_p_d_f_g_h_i_6_3_3_2_0_0_0_error_1.9e-05
   14
     1   0   0   1   1
      0.3445147905    1.0000000000
     2   0   0   1   1
      0.6420248017    1.0000000000
     3   0   0   1   1
      2.0632391955    1.0000000000
     4   0   0   1   1
      8.1313932030    1.0000000000
     5   0   0   1   1
     30.2419817321    1.0000000000
     6   0   0   1   1
    110.1047185739    1.0000000000
     7   1   1   1   1
      0.4094066903    1.0000000000
     8   1   1   1   1
      1.2224583728    1.0000000000
     9   1   1   1   1
      7.7161882548    1.0000000000
    10   2   2   1   1
      0.6071734694    1.0000000000
    11   2   2   1   1
      0.8491719256    1.0000000000
    12   2   2   1   1
      5.8098795328    1.0000000000
    13   3   3   1   1
      0.6445900563    1.0000000000
    14   3   3   1   1
      3.9566890997    1.0000000000

# RI basis set for N (all-electron) relative DI metric: 2.1e-06
N  RI_aug-SZV-MOLOPT-ae-SR_N_RI_050_s_p_d_f_g_h_i_6_5_3_2_0_0_0_error_2.1e-06
   16
     1   0   0   1   1
      0.3609201500    1.0000000000
     2   0   0   1   1
      0.6750674925    1.0000000000
     3   0   0   1   1
      1.9647064424    1.0000000000
     4   0   0   1   1
      8.0564086166    1.0000000000
     5   0   0   1   1
     30.2136718519    1.0000000000
     6   0   0   1   1
    110.0722584980    1.0000000000
     7   1   1   1   1
      0.3549994408    1.0000000000
     8   1   1   1   1
      0.8651404647    1.0000000000
     9   1   1   1   1
      2.6452371665    1.0000000000
    10   1   1   1   1
      7.6635290625    1.0000000000
    11   1   1   1   1
     22.3167872648    1.0000000000
    12   2   2   1   1
      0.5242047877    1.0000000000
    13   2   2   1   1
      1.2479798128    1.0000000000
    14   2   2   1   1
      5.7524136451    1.0000000000
    15   3   3   1   1
      0.6048957981    1.0000000000
    16   3   3   1   1
      3.0267596576    1.0000000000

# RI basis set for N (all-electron) relative DI metric: 7.2e-07
N  RI_aug-SZV-MOLOPT-ae-SR_N_RI_057_s_p_d_f_g_h_i_6_5_3_3_0_0_0_error_7.2e-07
   17
     1   0   0   1   1
      0.3688965063    1.0000000000
     2   0   0   1   1
      0.6864355261    1.0000000000
     3   0   0   1   1
      1.7492346017    1.0000000000
     4   0   0   1   1
```

```
      7.6818406016    1.0000000000
     5   0   0   1   1
     30.0601523465    1.0000000000
     6   0   0   1   1
    109.9791587620    1.0000000000
     7   1   1   1   1
      0.3271271441    1.0000000000
     8   1   1   1   1
      0.8572018536    1.0000000000
     9   1   1   1   1
      2.4027958967    1.0000000000
    10   1   1   1   1
      7.4796290287    1.0000000000
    11   1   1   1   1
     22.1585213504    1.0000000000
    12   2   2   1   1
      0.5227525898    1.0000000000
    13   2   2   1   1
      1.2831404516    1.0000000000
    14   2   2   1   1
      5.6078176972    1.0000000000
    15   3   3   1   1
      0.4856017677    1.0000000000
    16   3   3   1   1
      1.2361958224    1.0000000000
    17   3   3   1   1
      3.4529556155    1.0000000000

# RI basis set for N (all-electron) relative DI metric: 1.5e-07
N  RI_aug-SZV-MOLOPT-ae-SR_N_RI_089_s_p_d_f_g_h_i_6_5_4_3_3_0_0_error_1.5e-07
   21
     1   0   0   1   1
      0.2675019515    1.0000000000
     2   0   0   1   1
      0.4703449573    1.0000000000
     3   0   0   1   1
      1.6082178401    1.0000000000
     4   0   0   1   1
      7.3481487307    1.0000000000
     5   0   0   1   1
     29.8956732465    1.0000000000
     6   0   0   1   1
    109.7791665284    1.0000000000
     7   1   1   1   1
      0.3631686075    1.0000000000
     8   1   1   1   1
      0.6360078805    1.0000000000
     9   1   1   1   1
      2.5379095518    1.0000000000
    10   1   1   1   1
      7.3968406671    1.0000000000
    11   1   1   1   1
     21.5085008916    1.0000000000
    12   2   2   1   1
      0.3695171557    1.0000000000
    13   2   2   1   1
      0.5736298636    1.0000000000
    14   2   2   1   1
      2.0879181319    1.0000000000
    15   2   2   1   1
      5.3667195220    1.0000000000
    16   3   3   1   1
      0.5161189175    1.0000000000
    17   3   3   1   1
      1.1546474805    1.0000000000
    18   3   3   1   1
      3.6284034545    1.0000000000
    19   4   4   1   1
      0.8708591071    1.0000000000
    20   4   4   1   1
      1.2478476380    1.0000000000
    21   4   4   1   1
      1.8203166985    1.0000000000

# RI basis set for N (all-electron) relative DI metric: 3.1e-02
N  RI_aug-SZV-MOLOPT-ae_N_RI_010_s_p_d_f_g_h_i_2_1_1_0_0_0_0_error_3.1e-02
   4
     1   0   0   1   1
      2.4025167189    1.0000000000
     2   0   0   1   1
     10.1393897410    1.0000000000
     3   1   1   1   1
      1.0654461177    1.0000000000
     4   2   2   1   1
      1.1902917242    1.0000000000

# RI basis set for N (all-electron) relative DI metric: 8.1e-03
N  RI_aug-SZV-MOLOPT-ae_N_RI_019_s_p_d_f_g_h_i_4_1_1_1_0_0_0_error_8.1e-03
   7
     1   0   0   1   1
      0.3123515208    1.0000000000
     2   0   0   1   1
      2.0783921931    1.0000000000
     3   0   0   1   1
     12.7176806031    1.0000000000
     4   0   0   1   1
    112.3647860911    1.0000000000
     5   1   1   1   1
      1.3806301601    1.0000000000
     6   2   2   1   1
      1.2416012478    1.0000000000
     7   3   3   1   1
      0.3604259987    1.0000000000

# RI basis set for N (all-electron) relative DI metric: 2.3e-03
N  RI_aug-SZV-MOLOPT-ae_N_RI_033_s_p_d_f_g_h_i_6_5_1_1_0_0_0_error_2.3e-03
   13
     1   0   0   1   1
      0.6068131992    1.0000000000
     2   0   0   1   1
      1.8169899039    1.0000000000
```

```
      3   0   0   1   1
       4.6697401244    1.0000000000
      4   0   0   1   1
      12.9893976075    1.0000000000
      5   0   0   1   1
      38.7614688329    1.0000000000
      6   0   0   1   1
     117.7766022022    1.0000000000
      7   1   1   1   1
       0.5590053395    1.0000000000
      8   1   1   1   1
       1.4848299634    1.0000000000
      9   1   1   1   1
       2.9877077459    1.0000000000
     10   1   1   1   1
       7.6017060524    1.0000000000
     11   1   1   1   1
      18.4576390056    1.0000000000
     12   2   2   1   1
       1.1047660361    1.0000000000
     13   3   3   1   1
       1.3064331409    1.0000000000

# RI basis set for N (all-electron) relative DI metric: 2.2e-05
N  RI_aug-SZV-MOLOPT-ae_N_RI_043_s_p_d_f_g_h_i_6_5_3_1_0_0_0_error_2.2e-05
   15
      1   0   0   1   1
       0.3488811538    1.0000000000
      2   0   0   1   1
       0.6042559787    1.0000000000
      3   0   0   1   1
       2.1157261719    1.0000000000
      4   0   0   1   1
       8.1019254260    1.0000000000
      5   0   0   1   1
      30.2074743369    1.0000000000
      6   0   0   1   1
     110.0780369594    1.0000000000
      7   1   1   1   1
       0.5654298479    1.0000000000
      8   1   1   1   1
       0.7363840894    1.0000000000
      9   1   1   1   1
       3.0579978651    1.0000000000
     10   1   1   1   1
       9.2426226532    1.0000000000
     11   1   1   1   1
      24.5338249142    1.0000000000
     12   2   2   1   1
       0.4158986316    1.0000000000
     13   2   2   1   1
       1.3391448868    1.0000000000
     14   2   2   1   1
       5.4063406985    1.0000000000
     15   3   3   1   1
       1.1978072490    1.0000000000

# RI basis set for N (all-electron) relative DI metric: 1.0e-06
N  RI_aug-SZV-MOLOPT-ae_N_RI_051_s_p_d_f_g_h_i_7_5_3_2_0_0_0_error_1.0e-06
   17
      1   0   0   1   1
       0.3088837141    1.0000000000
      2   0   0   1   1
       0.8451552729    1.0000000000
      3   0   0   1   1
       2.2780884387    1.0000000000
      4   0   0   1   1
       6.0084815626    1.0000000000
      5   0   0   1   1
      15.8311841775    1.0000000000
      6   0   0   1   1
      41.7231306103    1.0000000000
      7   0   0   1   1
     109.9691726721    1.0000000000
      8   1   1   1   1
       0.3723247906    1.0000000000
      9   1   1   1   1
       1.0776389078    1.0000000000
     10   1   1   1   1
       2.2377500420    1.0000000000
     11   1   1   1   1
       7.6271649510    1.0000000000
     12   1   1   1   1
      22.3169370744    1.0000000000
     13   2   2   1   1
       0.4011466484    1.0000000000
     14   2   2   1   1
       1.4414205470    1.0000000000
     15   2   2   1   1
       5.2803588958    1.0000000000
     16   3   3   1   1
       0.6136024375    1.0000000000
     17   3   3   1   1
       2.0818956914    1.0000000000

# RI basis set for N (all-electron) relative DI metric: 4.2e-07
N  RI_aug-SZV-MOLOPT-ae_N_RI_054_s_p_d_f_g_h_i_7_6_3_2_0_0_0_error_4.2e-07
   18
      1   0   0   1   1
       0.2277209174    1.0000000000
      2   0   0   1   1
       0.5103911276    1.0000000000
      3   0   0   1   1
       1.4510222987    1.0000000000
      4   0   0   1   1
       4.3632375872    1.0000000000
      5   0   0   1   1
      12.8636150561    1.0000000000
      6   0   0   1   1
      37.6680087660    1.0000000000
      7   0   0   1   1
```

```
    110.2424673764    1.0000000000
     8   1   1   1   1
      0.3789487168    1.0000000000
     9   1   1   1   1
      0.7196498314    1.0000000000
    10   1   1   1   1
      1.7775728371    1.0000000000
    11   1   1   1   1
      4.0940538071    1.0000000000
    12   1   1   1   1
      9.5256112941    1.0000000000
    13   1   1   1   1
     22.3494126721    1.0000000000
    14   2   2   1   1
      0.3918460188    1.0000000000
    15   2   2   1   1
      1.4556190928    1.0000000000
    16   2   2   1   1
      5.5566406406    1.0000000000
    17   3   3   1   1
      0.4833168716    1.0000000000
    18   3   3   1   1
      2.3342893057    1.0000000000

# RI basis set for N (all-electron) relative DI metric: 1.8e-07
N  RI_aug-SZV-MOLOPT-ae_N_RI_059_s_p_d_f_g_h_i_7_6_4_2_0_0_0_error_1.8e-07
   19
     1   0   0   1   1
      0.2753240332    1.0000000000
     2   0   0   1   1
      0.5115753207    1.0000000000
     3   0   0   1   1
      1.3677725119    1.0000000000
     4   0   0   1   1
      4.2732665299    1.0000000000
     5   0   0   1   1
     12.8010392407    1.0000000000
     6   0   0   1   1
     37.6019151399    1.0000000000
     7   0   0   1   1
    110.1717860237    1.0000000000
     8   1   1   1   1
      0.3395473111    1.0000000000
     9   1   1   1   1
      0.7134050198    1.0000000000
    10   1   1   1   1
      1.8068039487    1.0000000000
    11   1   1   1   1
      4.1276003657    1.0000000000
    12   1   1   1   1
      9.5354362670    1.0000000000
    13   1   1   1   1
     22.3569050202    1.0000000000
    14   2   2   1   1
      0.2921677417    1.0000000000
    15   2   2   1   1
      0.8075758781    1.0000000000
    16   2   2   1   1
      1.8162896360    1.0000000000
    17   2   2   1   1
      5.5901829862    1.0000000000
    18   3   3   1   1
      0.4722452116    1.0000000000
    19   3   3   1   1
      2.1764165813    1.0000000000

# RI basis set for N (all-electron) relative DI metric: 8.3e-08
N  RI_aug-SZV-MOLOPT-ae_N_RI_078_s_p_d_f_g_h_i_7_6_5_4_0_0_0_error_8.3e-08
   22
     1   0   0   1   1
      0.3186179571    1.0000000000
     2   0   0   1   1
      0.5028126171    1.0000000000
     3   0   0   1   1
      1.2423577644    1.0000000000
     4   0   0   1   1
      4.1017620715    1.0000000000
     5   0   0   1   1
     12.6710382019    1.0000000000
     6   0   0   1   1
     37.4653215997    1.0000000000
     7   0   0   1   1
    110.0278252602    1.0000000000
     8   1   1   1   1
      0.3210979812    1.0000000000
     9   1   1   1   1
      0.7348489340    1.0000000000
    10   1   1   1   1
      1.5858313586    1.0000000000
    11   1   1   1   1
      3.9642515363    1.0000000000
    12   1   1   1   1
      9.4534847432    1.0000000000
    13   1   1   1   1
     22.2755383613    1.0000000000
    14   2   2   1   1
      0.2913617779    1.0000000000
    15   2   2   1   1
      0.5939891009    1.0000000000
    16   2   2   1   1
      1.2537663619    1.0000000000
    17   2   2   1   1
      2.7053808523    1.0000000000
    18   2   2   1   1
      5.7108379444    1.0000000000
    19   3   3   1   1
      0.4874594538    1.0000000000
    20   3   3   1   1
      0.9083545544    1.0000000000
    21   3   3   1   1
      1.7615934583    1.0000000000
```

```
    22   3   3   1   1
      3.3878483320    1.0000000000

# RI basis set for N (all-electron) relative DI metric: 3.2e-02
N  RI_aug-DZVP-MOLOPT-ae_N_RI_019_s_p_d_f_g_h_i_4_1_1_1_0_0_0_error_3.2e-02
   7
     1   0   0   1   1
      0.6337220731    1.0000000000
     2   0   0   1   1
      2.4888620430    1.0000000000
     3   0   0   1   1
     12.3865456560    1.0000000000
     4   0   0   1   1
     55.4796719544    1.0000000000
     5   1   1   1   1
      1.2753414258    1.0000000000
     6   2   2   1   1
      1.4694326146    1.0000000000
     7   3   3   1   1
      0.7435641067    1.0000000000

# RI basis set for N (all-electron) relative DI metric: 3.5e-03
N  RI_aug-DZVP-MOLOPT-ae_N_RI_031_s_p_d_f_g_h_i_4_2_1_1_1_0_0_error_3.5e-03
   9
     1   0   0   1   1
      0.7877470285    1.0000000000
     2   0   0   1   1
      4.3096680771    1.0000000000
     3   0   0   1   1
     21.1630494118    1.0000000000
     4   0   0   1   1
    110.2266600801    1.0000000000
     5   1   1   1   1
      0.5881620256    1.0000000000
     6   1   1   1   1
      1.8301870067    1.0000000000
     7   2   2   1   1
      1.4920485746    1.0000000000
     8   3   3   1   1
      1.0521726037    1.0000000000
     9   4   4   1   1
      0.5883866521    1.0000000000

# RI basis set for N (all-electron) relative DI metric: 9.2e-04
N  RI_aug-DZVP-MOLOPT-ae_N_RI_039_s_p_d_f_g_h_i_4_3_2_1_1_0_0_error_9.2e-04
   11
     1   0   0   1   1
      0.3622921711    1.0000000000
     2   0   0   1   1
      1.6354963080    1.0000000000
     3   0   0   1   1
     12.7639396884    1.0000000000
     4   0   0   1   1
    110.0543942234    1.0000000000
     5   1   1   1   1
      0.6270722879    1.0000000000
     6   1   1   1   1
      1.2592391587    1.0000000000
     7   1   1   1   1
     13.7353229588    1.0000000000
     8   2   2   1   1
      0.7610417678    1.0000000000
     9   2   2   1   1
      3.4659460097    1.0000000000
    10   3   3   1   1
      1.0837990674    1.0000000000
    11   4   4   1   1
      1.2910568292    1.0000000000

# RI basis set for N (all-electron) relative DI metric: 3.5e-04
N  RI_aug-DZVP-MOLOPT-ae_N_RI_043_s_p_d_f_g_h_i_5_4_2_1_1_0_0_error_3.5e-04
   13
     1   0   0   1   1
      0.4774483883    1.0000000000
     2   0   0   1   1
      0.6726701107    1.0000000000
     3   0   0   1   1
      3.0353306658    1.0000000000
     4   0   0   1   1
     19.0591267866    1.0000000000
     5   0   0   1   1
    106.5417909780    1.0000000000
     6   1   1   1   1
      0.5703561299    1.0000000000
     7   1   1   1   1
      0.8919639409    1.0000000000
     8   1   1   1   1
      3.7010959109    1.0000000000
     9   1   1   1   1
     20.2508685042    1.0000000000
    10   2   2   1   1
      0.6789894290    1.0000000000
    11   2   2   1   1
      1.9698804689    1.0000000000
    12   3   3   1   1
      1.1840866026    1.0000000000
    13   4   4   1   1
      1.0016913950    1.0000000000

# RI basis set for N (all-electron) relative DI metric: 1.6e-04
N  RI_aug-DZVP-MOLOPT-ae_N_RI_051_s_p_d_f_g_h_i_6_4_2_2_1_0_0_error_1.6e-04
   15
     1   0   0   1   1
      0.2783158228    1.0000000000
     2   0   0   1   1
      0.5380358598    1.0000000000
     3   0   0   1   1
      1.9069630279    1.0000000000
     4   0   0   1   1
      7.7414876947    1.0000000000
     5   0   0   1   1
```

```
     29.8474403973    1.0000000000
     6   0   0   1   1
    109.7910431053    1.0000000000
     7   1   1   1   1
      0.4301472803    1.0000000000
     8   1   1   1   1
      1.0306055181    1.0000000000
     9   1   1   1   1
      3.6880896959    1.0000000000
    10   1   1   1   1
     20.0563452996    1.0000000000
    11   2   2   1   1
      0.6598514115    1.0000000000
    12   2   2   1   1
      2.0088880905    1.0000000000
    13   3   3   1   1
      0.7357954895    1.0000000000
    14   3   3   1   1
      2.2996062252    1.0000000000
    15   4   4   1   1
      1.0178044814    1.0000000000

# RI basis set for N (all-electron) relative DI metric: 6.6e-05
N  RI_aug-DZVP-MOLOPT-ae_N_RI_056_s_p_d_f_g_h_i_6_4_3_2_1_0_0_error_6.6e-05
   16
     1   0   0   1   1
      0.2949790136    1.0000000000
     2   0   0   1   1
      0.7785123249    1.0000000000
     3   0   0   1   1
      2.4077176827    1.0000000000
     4   0   0   1   1
      9.0620020650    1.0000000000
     5   0   0   1   1
     31.5803321143    1.0000000000
     6   0   0   1   1
    106.8824935458    1.0000000000
     7   1   1   1   1
      0.3687995805    1.0000000000
     8   1   1   1   1
      1.0537994381    1.0000000000
     9   1   1   1   1
      3.9145827044    1.0000000000
    10   1   1   1   1
     20.6989803748    1.0000000000
    11   2   2   1   1
      0.6759504032    1.0000000000
    12   2   2   1   1
      0.8727684173    1.0000000000
    13   2   2   1   1
      4.4005869673    1.0000000000
    14   3   3   1   1
      0.6555017925    1.0000000000
    15   3   3   1   1
      2.3267347662    1.0000000000
    16   4   4   1   1
      1.3371746309    1.0000000000

# RI basis set for N (all-electron) relative DI metric: 3.1e-05
N  RI_aug-DZVP-MOLOPT-ae_N_RI_059_s_p_d_f_g_h_i_6_5_3_2_1_0_0_error_3.1e-05
   17
     1   0   0   1   1
      0.3731029845    1.0000000000
     2   0   0   1   1
      0.6182386393    1.0000000000
     3   0   0   1   1
      1.7456897436    1.0000000000
     4   0   0   1   1
      7.6216208759    1.0000000000
     5   0   0   1   1
     29.8143933546    1.0000000000
     6   0   0   1   1
    109.6768583408    1.0000000000
     7   1   1   1   1
      0.4150661970    1.0000000000
     8   1   1   1   1
      0.8817944706    1.0000000000
     9   1   1   1   1
      2.3611160326    1.0000000000
    10   1   1   1   1
      7.5219437369    1.0000000000
    11   1   1   1   1
     22.2129391888    1.0000000000
    12   2   2   1   1
      0.4415892513    1.0000000000
    13   2   2   1   1
      0.8846731406    1.0000000000
    14   2   2   1   1
      3.5662320160    1.0000000000
    15   3   3   1   1
      0.6643853280    1.0000000000
    16   3   3   1   1
      2.2087835061    1.0000000000
    17   4   4   1   1
      1.3020622384    1.0000000000

# RI basis set for N (all-electron) relative DI metric: 9.7e-06
N  RI_aug-DZVP-MOLOPT-ae_N_RI_068_s_p_d_f_g_h_i_6_5_3_2_2_0_0_error_9.7e-06
   18
     1   0   0   1   1
      0.3165071392    1.0000000000
     2   0   0   1   1
      0.5722077846    1.0000000000
     3   0   0   1   1
      1.9269510127    1.0000000000
     4   0   0   1   1
      8.0366046608    1.0000000000
     5   0   0   1   1
     30.1911144685    1.0000000000
     6   0   0   1   1
    110.0609699459    1.0000000000
```

```
    7   1   1   1   1
     0.4113721857    1.0000000000
    8   1   1   1   1
     0.8092548399    1.0000000000
    9   1   1   1   1
     2.3286469947    1.0000000000
   10   1   1   1   1
     7.4771990298    1.0000000000
   11   1   1   1   1
    22.1708647917    1.0000000000
   12   2   2   1   1
     0.4203212864    1.0000000000
   13   2   2   1   1
     1.1167275027    1.0000000000
   14   2   2   1   1
     4.3524086894    1.0000000000
   15   3   3   1   1
     0.6299936330    1.0000000000
   16   3   3   1   1
     1.8607856226    1.0000000000
   17   4   4   1   1
     0.6927359127    1.0000000000
   18   4   4   1   1
     2.0011179420    1.0000000000

# RI basis set for N (all-electron) relative DI metric: 2.7e-06
N  RI_aug-DZVP-MOLOPT-ae_N_RI_075_s_p_d_f_g_h_i_6_5_3_3_2_0_0_error_2.7e-06
   19
    1   0   0   1   1
     0.3591070304    1.0000000000
    2   0   0   1   1
     0.4865362652    1.0000000000
    3   0   0   1   1
     1.6390402008    1.0000000000
    4   0   0   1   1
     7.4039596248    1.0000000000
    5   0   0   1   1
    29.7670457880    1.0000000000
    6   0   0   1   1
   109.6997495514    1.0000000000
    7   1   1   1   1
     0.3330383247    1.0000000000
    8   1   1   1   1
     0.6673384734    1.0000000000
    9   1   1   1   1
     1.9388704770    1.0000000000
   10   1   1   1   1
     7.3869934120    1.0000000000
   11   1   1   1   1
    22.1219138829    1.0000000000
   12   2   2   1   1
     0.4024394074    1.0000000000
   13   2   2   1   1
     1.0222849475    1.0000000000
   14   2   2   1   1
     3.9142917190    1.0000000000
   15   3   3   1   1
     0.4551067667    1.0000000000
   16   3   3   1   1
     1.1609166338    1.0000000000
   17   3   3   1   1
     3.5132663400    1.0000000000
   18   4   4   1   1
     0.7345268692    1.0000000000
   19   4   4   1   1
     2.1367859617    1.0000000000

# RI basis set for N (all-electron) relative DI metric: 1.0e-06
N  RI_aug-DZVP-MOLOPT-ae_N_RI_084_s_p_d_f_g_h_i_7_6_4_3_2_0_0_error_1.0e-06
   22
    1   0   0   1   1
     0.2574076637    1.0000000000
    2   0   0   1   1
     0.4892081731    1.0000000000
    3   0   0   1   1
     1.4417080922    1.0000000000
    4   0   0   1   1
     4.3835750306    1.0000000000
    5   0   0   1   1
    12.8638910455    1.0000000000
    6   0   0   1   1
    37.6677667429    1.0000000000
    7   0   0   1   1
   110.2464508427    1.0000000000
    8   1   1   1   1
     0.3108518762    1.0000000000
    9   1   1   1   1
     0.6845875897    1.0000000000
   10   1   1   1   1
     1.6165364374    1.0000000000
   11   1   1   1   1
     4.0519934629    1.0000000000
   12   1   1   1   1
     9.4916608581    1.0000000000
   13   1   1   1   1
    22.3005490078    1.0000000000
   14   2   2   1   1
     0.3790654550    1.0000000000
   15   2   2   1   1
     0.7605758519    1.0000000000
   16   2   2   1   1
     2.0093282334    1.0000000000
   17   2   2   1   1
     5.6769357170    1.0000000000
   18   3   3   1   1
     0.3979363188    1.0000000000
   19   3   3   1   1
     1.1072873728    1.0000000000
   20   3   3   1   1
     3.4991425994    1.0000000000
   21   4   4   1   1
```

```
      0.6213987031    1.0000000000
    22   4   4   1   1
      1.9847660952    1.0000000000

# RI basis set for N (all-electron) relative DI metric: 4.5e-07
N  RI_aug-DZVP-MOLOPT-ae_N_RI_089_s_p_d_f_g_h_i_7_6_5_3_2_0_0_error_4.5e-07
   23
     1   0   0   1   1
      0.3083642064    1.0000000000
     2   0   0   1   1
      0.3900660902    1.0000000000
     3   0   0   1   1
      1.2436811890    1.0000000000
     4   0   0   1   1
      4.3013257694    1.0000000000
     5   0   0   1   1
     12.7630999338    1.0000000000
     6   0   0   1   1
     37.5545867769    1.0000000000
     7   0   0   1   1
    110.1289417591    1.0000000000
     8   1   1   1   1
      0.2128335779    1.0000000000
     9   1   1   1   1
      0.5818385031    1.0000000000
    10   1   1   1   1
      1.3798997486    1.0000000000
    11   1   1   1   1
      4.0919828016    1.0000000000
    12   1   1   1   1
      9.4598902733    1.0000000000
    13   1   1   1   1
     22.2212108205    1.0000000000
    14   2   2   1   1
      0.3642814436    1.0000000000
    15   2   2   1   1
      0.5517099031    1.0000000000
    16   2   2   1   1
      1.3602076208    1.0000000000
    17   2   2   1   1
      2.6468895760    1.0000000000
    18   2   2   1   1
      5.6370529153    1.0000000000
    19   3   3   1   1
      0.3686439491    1.0000000000
    20   3   3   1   1
      0.9856099335    1.0000000000
    21   3   3   1   1
      3.2061667961    1.0000000000
    22   4   4   1   1
      0.7076664781    1.0000000000
    23   4   4   1   1
      2.1268127230    1.0000000000

# RI basis set for N (all-electron) relative DI metric: 2.7e-02
N  RI_aug-TZVP-MOLOPT-ae_N_RI_023_s_p_d_f_g_h_i_5_2_1_1_0_0_0_error_2.7e-02
   9
     1   0   0   1   1
      0.7070649969    1.0000000000
     2   0   0   1   1
      2.4870463204    1.0000000000
     3   0   0   1   1
      8.8030349073    1.0000000000
     4   0   0   1   1
     31.1296524299    1.0000000000
     5   0   0   1   1
    109.9178056587    1.0000000000
     6   1   1   1   1
      0.9555337177    1.0000000000
     7   1   1   1   1
      2.6400378242    1.0000000000
     8   2   2   1   1
      1.3655786143    1.0000000000
     9   3   3   1   1
      1.5383058496    1.0000000000

# RI basis set for N (all-electron) relative DI metric: 8.5e-03
N  RI_aug-TZVP-MOLOPT-ae_N_RI_031_s_p_d_f_g_h_i_5_3_2_1_0_0_0_error_8.5e-03
   11
     1   0   0   1   1
      0.4057855853    1.0000000000
     2   0   0   1   1
      1.4398864411    1.0000000000
     3   0   0   1   1
      6.5997817510    1.0000000000
     4   0   0   1   1
     27.1507516417    1.0000000000
     5   0   0   1   1
    106.4489147975    1.0000000000
     6   1   1   1   1
      0.8417204108    1.0000000000
     7   1   1   1   1
      1.6327959469    1.0000000000
     8   1   1   1   1
     15.3521371962    1.0000000000
     9   2   2   1   1
      0.7844464596    1.0000000000
    10   2   2   1   1
      2.6654025015    1.0000000000
    11   3   3   1   1
      1.4085901621    1.0000000000

# RI basis set for N (all-electron) relative DI metric: 3.7e-03
N  RI_aug-TZVP-MOLOPT-ae_N_RI_044_s_p_d_f_g_h_i_6_4_2_1_1_0_0_error_3.7e-03
   14
     1   0   0   1   1
      0.0924350268    1.0000000000
     2   0   0   1   1
      0.4592275360    1.0000000000
     3   0   0   1   1
      1.6094381114    1.0000000000
```

```
    4   0   0   1   1
     6.5036335540    1.0000000000
    5   0   0   1   1
    26.5949090230    1.0000000000
    6   0   0   1   1
   110.2886429351    1.0000000000
    7   1   1   1   1
     0.1739697641    1.0000000000
    8   1   1   1   1
     0.9146694686    1.0000000000
    9   1   1   1   1
     3.7351940695    1.0000000000
   10   1   1   1   1
    19.2314816948    1.0000000000
   11   2   2   1   1
     0.8998948907    1.0000000000
   12   2   2   1   1
     2.5401226409    1.0000000000
   13   3   3   1   1
     1.3678504526    1.0000000000
   14   4   4   1   1
     1.2961955778    1.0000000000

# RI basis set for N (all-electron) relative DI metric: 9.9e-04
N  RI_aug-TZVP-MOLOPT-ae_N_RI_055_s_p_d_f_g_h_i_7_5_2_2_1_0_0_error_9.9e-04
   17
    1   0   0   1   1
     0.0852784338    1.0000000000
    2   0   0   1   1
     0.2808132756    1.0000000000
    3   0   0   1   1
     0.9246862034    1.0000000000
    4   0   0   1   1
     3.0448872113    1.0000000000
    5   0   0   1   1
    10.0264828544    1.0000000000
    6   0   0   1   1
    33.0161469742    1.0000000000
    7   0   0   1   1
   108.7186374273    1.0000000000
    8   1   1   1   1
     0.3940530906    1.0000000000
    9   1   1   1   1
     0.8320492104    1.0000000000
   10   1   1   1   1
     2.3739404395    1.0000000000
   11   1   1   1   1
     7.3095279425    1.0000000000
   12   1   1   1   1
    22.0012271745    1.0000000000
   13   2   2   1   1
     0.8400542597    1.0000000000
   14   2   2   1   1
     2.1806038242    1.0000000000
   15   3   3   1   1
     0.8160494412    1.0000000000
   16   3   3   1   1
     2.1564793118    1.0000000000
   17   4   4   1   1
     1.2498956211    1.0000000000

# RI basis set for N (all-electron) relative DI metric: 4.1e-04
N  RI_aug-TZVP-MOLOPT-ae_N_RI_060_s_p_d_f_g_h_i_7_5_3_2_1_0_0_error_4.1e-04
   18
    1   0   0   1   1
     0.0945528768    1.0000000000
    2   0   0   1   1
     0.3898031726    1.0000000000
    3   0   0   1   1
     0.7715285110    1.0000000000
    4   0   0   1   1
     1.8552453133    1.0000000000
    5   0   0   1   1
     7.9851050827    1.0000000000
    6   0   0   1   1
    31.1361135343    1.0000000000
    7   0   0   1   1
   107.6422077786    1.0000000000
    8   1   1   1   1
     0.4549916422    1.0000000000
    9   1   1   1   1
     0.8258554462    1.0000000000
   10   1   1   1   1
     2.0690369781    1.0000000000
   11   1   1   1   1
     6.4662857949    1.0000000000
   12   1   1   1   1
    21.6870091007    1.0000000000
   13   2   2   1   1
     0.8763019852    1.0000000000
   14   2   2   1   1
     1.5324257723    1.0000000000
   15   2   2   1   1
     5.8658674369    1.0000000000
   16   3   3   1   1
     0.7915999005    1.0000000000
   17   3   3   1   1
     2.1163298544    1.0000000000
   18   4   4   1   1
     1.3981231793    1.0000000000

# RI basis set for N (all-electron) relative DI metric: 1.7e-04
N  RI_aug-TZVP-MOLOPT-ae_N_RI_069_s_p_d_f_g_h_i_7_5_3_2_2_0_0_error_1.7e-04
   19
    1   0   0   1   1
     0.1332741678    1.0000000000
    2   0   0   1   1
     0.5181526975    1.0000000000
    3   0   0   1   1
     1.4260137566    1.0000000000
    4   0   0   1   1
```

```
      4.0648526631    1.0000000000
     5   0   0   1   1
     12.4409216140    1.0000000000
     6   0   0   1   1
     37.2869696332    1.0000000000
     7   0   0   1   1
    109.8484862881    1.0000000000
     8   1   1   1   1
      0.4883680646    1.0000000000
     9   1   1   1   1
      0.8562735637    1.0000000000
    10   1   1   1   1
      2.0953335829    1.0000000000
    11   1   1   1   1
      6.5933514752    1.0000000000
    12   1   1   1   1
     21.6928426703    1.0000000000
    13   2   2   1   1
      0.6169412394    1.0000000000
    14   2   2   1   1
      1.5462647407    1.0000000000
    15   2   2   1   1
      5.2089416276    1.0000000000
    16   3   3   1   1
      0.7398619034    1.0000000000
    17   3   3   1   1
      2.0925417807    1.0000000000
    18   4   4   1   1
      0.7897770203    1.0000000000
    19   4   4   1   1
      2.1464845239    1.0000000000

# RI basis set for N (all-electron) relative DI metric: 5.6e-05
N  RI_aug-TZVP-MOLOPT-ae_N_RI_081_s_p_d_f_g_h_i_7_5_4_3_2_0_0_error_5.6e-05
   21
     1   0   0   1   1
      0.3469575256    1.0000000000
     2   0   0   1   1
      0.7473109451    1.0000000000
     3   0   0   1   1
      1.5419936569    1.0000000000
     4   0   0   1   1
      4.7007373314    1.0000000000
     5   0   0   1   1
     14.4861957686    1.0000000000
     6   0   0   1   1
     40.2789959300    1.0000000000
     7   0   0   1   1
    107.8356854843    1.0000000000
     8   1   1   1   1
      0.4144342736    1.0000000000
     9   1   1   1   1
      1.0518806976    1.0000000000
    10   1   1   1   1
      3.0486123225    1.0000000000
    11   1   1   1   1
      8.4729723782    1.0000000000
    12   1   1   1   1
     21.8813835703    1.0000000000
    13   2   2   1   1
      0.4647633969    1.0000000000
    14   2   2   1   1
      1.2059616719    1.0000000000
    15   2   2   1   1
      2.4503051774    1.0000000000
    16   2   2   1   1
      7.9115907838    1.0000000000
    17   3   3   1   1
      0.6462244198    1.0000000000
    18   3   3   1   1
      1.0970712765    1.0000000000
    19   3   3   1   1
      2.6318605023    1.0000000000
    20   4   4   1   1
      0.5531996972    1.0000000000
    21   4   4   1   1
      1.7472098325    1.0000000000

# RI basis set for N (all-electron) relative DI metric: 2.2e-05
N  RI_aug-TZVP-MOLOPT-ae_N_RI_091_s_p_d_f_g_h_i_7_6_4_4_2_0_0_error_2.2e-05
   23
     1   0   0   1   1
      0.3770570899    1.0000000000
     2   0   0   1   1
      0.6686337432    1.0000000000
     3   0   0   1   1
      1.3930870484    1.0000000000
     4   0   0   1   1
      3.9581892855    1.0000000000
     5   0   0   1   1
     12.7894666410    1.0000000000
     6   0   0   1   1
     38.6001088391    1.0000000000
     7   0   0   1   1
    106.0590189442    1.0000000000
     8   1   1   1   1
      0.4167133074    1.0000000000
     9   1   1   1   1
      0.5937659471    1.0000000000
    10   1   1   1   1
      1.3271485005    1.0000000000
    11   1   1   1   1
      4.7745899253    1.0000000000
    12   1   1   1   1
     11.7789577401    1.0000000000
    13   1   1   1   1
     26.7762533284    1.0000000000
    14   2   2   1   1
      0.4400150321    1.0000000000
    15   2   2   1   1
      1.2918844349    1.0000000000
```

```
    16   2   2   1   1
      2.4341041256    1.0000000000
    17   2   2   1   1
      8.2315998859    1.0000000000
    18   3   3   1   1
      0.6728288981    1.0000000000
    19   3   3   1   1
      1.4851576806    1.0000000000
    20   3   3   1   1
      2.9834231895    1.0000000000
    21   3   3   1   1
      5.4903987655    1.0000000000
    22   4   4   1   1
      0.5959617108    1.0000000000
    23   4   4   1   1
      1.7777103546    1.0000000000

# RI basis set for N (all-electron) relative DI metric: 8.5e-06
N  RI_aug-TZVP-MOLOPT-ae_N_RI_112_s_p_d_f_g_h_i_7_6_6_4_2_1_0_error_8.5e-06
   26
     1   0   0   1   1
      0.2605142943    1.0000000000
     2   0   0   1   1
      0.7149120125    1.0000000000
     3   0   0   1   1
      1.3961230988    1.0000000000
     4   0   0   1   1
      3.9672157097    1.0000000000
     5   0   0   1   1
     12.8697912194    1.0000000000
     6   0   0   1   1
     38.6407000547    1.0000000000
     7   0   0   1   1
    105.4476503492    1.0000000000
     8   1   1   1   1
      0.3390610860    1.0000000000
     9   1   1   1   1
      0.5956399603    1.0000000000
    10   1   1   1   1
      1.3422832655    1.0000000000
    11   1   1   1   1
      4.7828796428    1.0000000000
    12   1   1   1   1
     11.7922770223    1.0000000000
    13   1   1   1   1
     26.9370630580    1.0000000000
    14   2   2   1   1
      0.2923766308    1.0000000000
    15   2   2   1   1
      0.5120536666    1.0000000000
    16   2   2   1   1
      0.9377737869    1.0000000000
    17   2   2   1   1
      1.7202621079    1.0000000000
    18   2   2   1   1
      3.2057022068    1.0000000000
    19   2   2   1   1
      6.1345170815    1.0000000000
    20   3   3   1   1
      0.5440126172    1.0000000000
    21   3   3   1   1
      1.2540791602    1.0000000000
    22   3   3   1   1
      3.0132268005    1.0000000000
    23   3   3   1   1
      6.8259402449    1.0000000000
    24   4   4   1   1
      0.4909597453    1.0000000000
    25   4   4   1   1
      1.9088292110    1.0000000000
    26   5   5   1   1
      0.7186725960    1.0000000000

# RI basis set for O (all-electron) relative DI metric: 4.8e-02
O  RI_aug-SZV-MOLOPT-ae-mini_N_RI_006_s_p_d_f_g_h_i_3_1_0_0_0_0_0_error_4.8e-02
   4
     1   0   0   1   1
      0.3719261910    1.0000000000
     2   0   0   1   1
      2.6236134276    1.0000000000
     3   0   0   1   1
     18.5072230466    1.0000000000
     4   1   1   1   1
      4.1669751099    1.0000000000

# RI basis set for O (all-electron) relative DI metric: 2.1e-02
O  RI_aug-SZV-MOLOPT-ae-mini_N_RI_011_s_p_d_f_g_h_i_3_1_1_0_0_0_0_error_2.1e-02
   5
     1   0   0   1   1
      0.8812418072    1.0000000000
     2   0   0   1   1
      1.4422904706    1.0000000000
     3   0   0   1   1
      2.3571863493    1.0000000000
     4   1   1   1   1
      4.1269642016    1.0000000000
     5   2   2   1   1
      4.3887768259    1.0000000000

# RI basis set for O (all-electron) relative DI metric: 2.9e-03
O  RI_aug-SZV-MOLOPT-ae-mini_N_RI_018_s_p_d_f_g_h_i_4_3_1_0_0_0_0_error_2.9e-03
   8
     1   0   0   1   1
      0.6659998294    1.0000000000
     2   0   0   1   1
      1.3219302659    1.0000000000
     3   0   0   1   1
      2.6328639073    1.0000000000
     4   0   0   1   1
      5.0890329646    1.0000000000
     5   1   1   1   1
```

```
      0.6345784442    1.0000000000
     6   1   1   1   1
      1.5667625222    1.0000000000
     7   1   1   1   1
      3.5512182878    1.0000000000
     8   2   2   1   1
      4.2358612041    1.0000000000

# RI basis set for O (all-electron) relative DI metric: 1.0e-06
O  RI_aug-SZV-MOLOPT-ae-mini_N_RI_028_s_p_d_f_g_h_i_4_3_3_0_0_0_0_error_1.0e-06
   10
     1   0   0   1   1
      0.3574894797    1.0000000000
     2   0   0   1   1
      1.0547527060    1.0000000000
     3   0   0   1   1
      3.0261945404    1.0000000000
     4   0   0   1   1
      8.3616103574    1.0000000000
     5   1   1   1   1
      0.4097478642    1.0000000000
     6   1   1   1   1
      1.7670212628    1.0000000000
     7   1   1   1   1
      8.4220623483    1.0000000000
     8   2   2   1   1
      0.4438790730    1.0000000000
     9   2   2   1   1
      2.1388351180    1.0000000000
    10   2   2   1   1
      8.0124493305    1.0000000000

# RI basis set for O (all-electron) relative DI metric: 4.0e-07
O  RI_aug-SZV-MOLOPT-ae-mini_N_RI_032_s_p_d_f_g_h_i_5_4_3_0_0_0_0_error_4.0e-07
   12
     1   0   0   1   1
      0.3700943529    1.0000000000
     2   0   0   1   1
      0.8375589417    1.0000000000
     3   0   0   1   1
      1.8493352639    1.0000000000
     4   0   0   1   1
      3.9333459393    1.0000000000
     5   0   0   1   1
      8.3743933127    1.0000000000
     6   1   1   1   1
      0.4452795331    1.0000000000
     7   1   1   1   1
      1.1847536603    1.0000000000
     8   1   1   1   1
      2.8358057607    1.0000000000
     9   1   1   1   1
      8.2256142484    1.0000000000
    10   2   2   1   1
      0.4501358715    1.0000000000
    11   2   2   1   1
      2.1142769217    1.0000000000
    12   2   2   1   1
      8.1013192044    1.0000000000

# RI basis set for O (all-electron) relative DI metric: 1.6e-07
O  RI_aug-SZV-MOLOPT-ae-mini_N_RI_042_s_p_d_f_g_h_i_7_5_4_0_0_0_0_error_1.6e-07
   16
     1   0   0   1   1
      0.4062275001    1.0000000000
     2   0   0   1   1
      0.6728566542    1.0000000000
     3   0   0   1   1
      1.1144889920    1.0000000000
     4   0   0   1   1
      1.8459886005    1.0000000000
     5   0   0   1   1
      3.0576111004    1.0000000000
     6   0   0   1   1
      5.0644872013    1.0000000000
     7   0   0   1   1
      8.3885850001    1.0000000000
     8   1   1   1   1
      0.4062274998    1.0000000000
     9   1   1   1   1
      0.8659626632    1.0000000000
    10   1   1   1   1
      1.8459886004    1.0000000000
    11   1   1   1   1
      3.9351279882    1.0000000000
    12   1   1   1   1
      8.3885850000    1.0000000000
    13   2   2   1   1
      0.4062275055    1.0000000000
    14   2   2   1   1
      1.1144889998    1.0000000000
    15   2   2   1   1
      3.0576110487    1.0000000000
    16   2   2   1   1
      8.3885850057    1.0000000000

# RI basis set for O (all-electron) relative DI metric: 2.2e-08
O  RI_aug-SZV-MOLOPT-ae-mini_N_RI_047_s_p_d_f_g_h_i_7_5_5_0_0_0_0_error_2.2e-08
   17
     1   0   0   1   1
      0.4062274998    1.0000000000
     2   0   0   1   1
      0.6728566540    1.0000000000
     3   0   0   1   1
      1.1144889919    1.0000000000
     4   0   0   1   1
      1.8459886004    1.0000000000
     5   0   0   1   1
      3.0576111002    1.0000000000
     6   0   0   1   1
      5.0644872012    1.0000000000
```

```
     7   0   0   1   1
      8.3885849998    1.0000000000
     8   1   1   1   1
      0.4062274998    1.0000000000
     9   1   1   1   1
      0.8659626632    1.0000000000
    10   1   1   1   1
      1.8459886004    1.0000000000
    11   1   1   1   1
      3.9351279882    1.0000000000
    12   1   1   1   1
      8.3885850000    1.0000000000
    13   2   2   1   1
      0.4062275000    1.0000000000
    14   2   2   1   1
      0.8659626633    1.0000000000
    15   2   2   1   1
      1.8459886004    1.0000000000
    16   2   2   1   1
      3.9351279883    1.0000000000
    17   2   2   1   1
      8.3885850000    1.0000000000

# RI basis set for O (all-electron) relative DI metric: 1.6e-02
O  RI_aug-SZV-MOLOPT-ae-SR_N_RI_027_s_p_d_f_g_h_i_5_4_2_0_0_0_0_error_1.6e-02
   11
     1   0   0   1   1
      0.6794244566    1.0000000000
     2   0   0   1   1
      1.3780269227    1.0000000000
     3   0   0   1   1
      6.1706388639    1.0000000000
     4   0   0   1   1
     32.0807873840    1.0000000000
     5   0   0   1   1
    162.6313565592    1.0000000000
     6   1   1   1   1
      0.2508902204    1.0000000000
     7   1   1   1   1
      1.4861431123    1.0000000000
     8   1   1   1   1
      2.7286584268    1.0000000000
     9   1   1   1   1
     17.6530861099    1.0000000000
    10   2   2   1   1
      0.8566924904    1.0000000000
    11   2   2   1   1
      3.2736961110    1.0000000000

# RI basis set for O (all-electron) relative DI metric: 1.1e-05
O  RI_aug-SZV-MOLOPT-ae-SR_N_RI_034_s_p_d_f_g_h_i_5_4_2_1_0_0_0_error_1.1e-05
   12
     1   0   0   1   1
      0.4376260301    1.0000000000
     2   0   0   1   1
      1.9300281813    1.0000000000
     3   0   0   1   1
      7.0454620304    1.0000000000
     4   0   0   1   1
     33.5362950213    1.0000000000
     5   0   0   1   1
    158.8449858148    1.0000000000
     6   1   1   1   1
      0.9004932103    1.0000000000
     7   1   1   1   1
      1.5807262262    1.0000000000
     8   1   1   1   1
      7.1872339896    1.0000000000
     9   1   1   1   1
     31.2066130295    1.0000000000
    10   2   2   1   1
      0.8339111160    1.0000000000
    11   2   2   1   1
      3.3555768922    1.0000000000
    12   3   3   1   1
      1.2293462032    1.0000000000

# RI basis set for O (all-electron) relative DI metric: 5.0e-06
O  RI_aug-SZV-MOLOPT-ae-SR_N_RI_041_s_p_d_f_g_h_i_5_4_2_2_0_0_0_error_5.0e-06
   13
     1   0   0   1   1
      0.5785871820    1.0000000000
     2   0   0   1   1
      1.3622440795    1.0000000000
     3   0   0   1   1
      6.3008887582    1.0000000000
     4   0   0   1   1
     32.0408502092    1.0000000000
     5   0   0   1   1
    162.6018184732    1.0000000000
     6   1   1   1   1
      0.7802953711    1.0000000000
     7   1   1   1   1
      1.6469785097    1.0000000000
     8   1   1   1   1
      7.2427109901    1.0000000000
     9   1   1   1   1
     31.2205637814    1.0000000000
    10   2   2   1   1
      0.8337057372    1.0000000000
    11   2   2   1   1
      3.3521208276    1.0000000000
    12   3   3   1   1
      1.1090668614    1.0000000000
    13   3   3   1   1
      4.5931523228    1.0000000000

# RI basis set for O (all-electron) relative DI metric: 7.1e-07
O  RI_aug-SZV-MOLOPT-ae-SR_N_RI_046_s_p_d_f_g_h_i_5_4_3_2_0_0_0_error_7.1e-07
   14
     1   0   0   1   1
```

```
      0.6465893825    1.0000000000
     2   0   0   1   1
      1.4297429381    1.0000000000
     3   0   0   1   1
      6.1570352512    1.0000000000
     4   0   0   1   1
     31.7515425922    1.0000000000
     5   0   0   1   1
    162.3112135095    1.0000000000
     6   1   1   1   1
      0.7341687692    1.0000000000
     7   1   1   1   1
      1.8877449030    1.0000000000
     8   1   1   1   1
      9.3319205529    1.0000000000
     9   1   1   1   1
     30.7546461742    1.0000000000
    10   2   2   1   1
      0.8147505556    1.0000000000
    11   2   2   1   1
      2.2028261421    1.0000000000
    12   2   2   1   1
      6.0546644380    1.0000000000
    13   3   3   1   1
      1.0770858186    1.0000000000
    14   3   3   1   1
      4.4458153677    1.0000000000

# RI basis set for O (all-electron) relative DI metric: 2.9e-07
O  RI_aug-SZV-MOLOPT-ae-SR_N_RI_051_s_p_d_f_g_h_i_5_4_4_2_0_0_0_error_2.9e-07
   15
     1   0   0   1   1
      0.6170576381    1.0000000000
     2   0   0   1   1
      1.5127793672    1.0000000000
     3   0   0   1   1
      5.9038087679    1.0000000000
     4   0   0   1   1
     30.5897084192    1.0000000000
     5   0   0   1   1
    161.2105867258    1.0000000000
     6   1   1   1   1
      0.7351513529    1.0000000000
     7   1   1   1   1
      1.6400468624    1.0000000000
     8   1   1   1   1
      5.2960944591    1.0000000000
     9   1   1   1   1
     25.8341939083    1.0000000000
    10   2   2   1   1
      0.3285273147    1.0000000000
    11   2   2   1   1
      0.9010950370    1.0000000000
    12   2   2   1   1
      2.2646722769    1.0000000000
    13   2   2   1   1
      7.5730990765    1.0000000000
    14   3   3   1   1
      0.9905683564    1.0000000000
    15   3   3   1   1
      2.9203372730    1.0000000000

# RI basis set for O (all-electron) relative DI metric: 1.4e-07
O  RI_aug-SZV-MOLOPT-ae-SR_N_RI_063_s_p_d_f_g_h_i_7_5_4_3_0_0_0_error_1.4e-07
   19
     1   0   0   1   1
      0.3807351972    1.0000000000
     2   0   0   1   1
      0.6262456551    1.0000000000
     3   0   0   1   1
      1.6760360144    1.0000000000
     4   0   0   1   1
      6.1110248502    1.0000000000
     5   0   0   1   1
     18.7152735005    1.0000000000
     6   0   0   1   1
     55.1658197624    1.0000000000
     7   0   0   1   1
    162.6509934533    1.0000000000
     8   1   1   1   1
      0.7212143374    1.0000000000
     9   1   1   1   1
      1.5418543920    1.0000000000
    10   1   1   1   1
      4.9203531182    1.0000000000
    11   1   1   1   1
     12.3471430129    1.0000000000
    12   1   1   1   1
     31.1234273958    1.0000000000
    13   2   2   1   1
      0.3036782006    1.0000000000
    14   2   2   1   1
      0.8898516607    1.0000000000
    15   2   2   1   1
      2.5231684828    1.0000000000
    16   2   2   1   1
      7.3232352815    1.0000000000
    17   3   3   1   1
      0.7762163234    1.0000000000
    18   3   3   1   1
      1.5996298128    1.0000000000
    19   3   3   1   1
      4.6605621473    1.0000000000

# RI basis set for O (all-electron) relative DI metric: 5.0e-08
O  RI_aug-SZV-MOLOPT-ae-SR_N_RI_084_s_p_d_f_g_h_i_7_6_4_3_2_0_0_error_5.0e-08
   22
     1   0   0   1   1
      0.3654518487    1.0000000000
     2   0   0   1   1
      0.6418179890    1.0000000000
```

```
      3   0   0   1   1
       1.7962770493    1.0000000000
      4   0   0   1   1
       6.2244019185    1.0000000000
      5   0   0   1   1
      18.7761307143    1.0000000000
      6   0   0   1   1
      55.2613658429    1.0000000000
      7   0   0   1   1
     162.7541788074    1.0000000000
      8   1   1   1   1
       0.5121080385    1.0000000000
      9   1   1   1   1
       0.9122976074    1.0000000000
     10   1   1   1   1
       2.2084557679    1.0000000000
     11   1   1   1   1
       5.5444662537    1.0000000000
     12   1   1   1   1
      13.1858740758    1.0000000000
     13   1   1   1   1
      31.3567690240    1.0000000000
     14   2   2   1   1
       0.3039135307    1.0000000000
     15   2   2   1   1
       0.9016923088    1.0000000000
     16   2   2   1   1
       2.5133805222    1.0000000000
     17   2   2   1   1
       7.3337659528    1.0000000000
     18   3   3   1   1
       1.0730493831    1.0000000000
     19   3   3   1   1
       2.2979655421    1.0000000000
     20   3   3   1   1
       4.7981866778    1.0000000000
     21   4   4   1   1
       0.9607658177    1.0000000000
     22   4   4   1   1
       2.2587729939    1.0000000000

# RI basis set for O (all-electron) relative DI metric: 1.9e-08
O  RI_aug-SZV-MOLOPT-ae-SR_N_RI_094_s_p_d_f_g_h_i_7_6_6_3_2_0_0_error_1.9e-08
   24
      1   0   0   1   1
       0.3515826621    1.0000000000
      2   0   0   1   1
       0.6448754961    1.0000000000
      3   0   0   1   1
       1.7898649211    1.0000000000
      4   0   0   1   1
       6.2409470948    1.0000000000
      5   0   0   1   1
      18.7949111636    1.0000000000
      6   0   0   1   1
      55.2761722729    1.0000000000
      7   0   0   1   1
     162.7693146685    1.0000000000
      8   1   1   1   1
       0.6044455579    1.0000000000
      9   1   1   1   1
       0.8237183654    1.0000000000
     10   1   1   1   1
       2.0808831664    1.0000000000
     11   1   1   1   1
       5.5132934628    1.0000000000
     12   1   1   1   1
      13.1373170891    1.0000000000
     13   1   1   1   1
      31.2951304076    1.0000000000
     14   2   2   1   1
       0.3028548892    1.0000000000
     15   2   2   1   1
       0.5727442735    1.0000000000
     16   2   2   1   1
       1.0843478581    1.0000000000
     17   2   2   1   1
       2.0499403326    1.0000000000
     18   2   2   1   1
       3.8758323274    1.0000000000
     19   2   2   1   1
       7.3213978891    1.0000000000
     20   3   3   1   1
       1.0425120714    1.0000000000
     21   3   3   1   1
       2.2677711242    1.0000000000
     22   3   3   1   1
       4.7950512595    1.0000000000
     23   4   4   1   1
       0.9750148016    1.0000000000
     24   4   4   1   1
       2.2699013156    1.0000000000

# RI basis set for O (all-electron) relative DI metric: 4.2e-02
O  RI_aug-SZV-MOLOPT-ae_N_RI_013_s_p_d_f_g_h_i_5_1_1_0_0_0_0_error_4.2e-02
   7
      1   0   0   1   1
       0.5062811446    1.0000000000
      2   0   0   1   1
       2.5926629766    1.0000000000
      3   0   0   1   1
      15.7440006433    1.0000000000
      4   0   0   1   1
      86.8209063052    1.0000000000
      5   0   0   1   1
     467.9184862809    1.0000000000
      6   1   1   1   1
       1.3461991559    1.0000000000
      7   2   2   1   1
       1.7411130613    1.0000000000
```

```
# RI basis set for O (all-electron) relative DI metric: 1.6e-02
O  RI_aug-SZV-MOLOPT-ae_N_RI_024_s_p_d_f_g_h_i_6_2_1_1_0_0_0_error_1.6e-02
   10
    1   0   0   1   1
     0.4406276910    1.0000000000
    2   0   0   1   1
     1.8768923182    1.0000000000
    3   0   0   1   1
     6.3429773099    1.0000000000
    4   0   0   1   1
    25.6975388317    1.0000000000
    5   0   0   1   1
   110.9173062863    1.0000000000
    6   0   0   1   1
   480.6209323922    1.0000000000
    7   1   1   1   1
     1.6749670250    1.0000000000
    8   1   1   1   1
    10.8429804748    1.0000000000
    9   2   2   1   1
     1.8997640822    1.0000000000
   10   3   3   1   1
     0.4642475464    1.0000000000

# RI basis set for O (all-electron) relative DI metric: 1.8e-04
O  RI_aug-SZV-MOLOPT-ae_N_RI_041_s_p_d_f_g_h_i_7_4_3_1_0_0_0_error_1.8e-04
   15
    1   0   0   1   1
     0.2180759215    1.0000000000
    2   0   0   1   1
     0.7183068446    1.0000000000
    3   0   0   1   1
     2.1090794763    1.0000000000
    4   0   0   1   1
     8.8558762166    1.0000000000
    5   0   0   1   1
    34.3514833756    1.0000000000
    6   0   0   1   1
   129.5971856888    1.0000000000
    7   0   0   1   1
   486.2785765669    1.0000000000
    8   1   1   1   1
     0.4802631422    1.0000000000
    9   1   1   1   1
     1.5304361353    1.0000000000
   10   1   1   1   1
    15.4992755738    1.0000000000
   11   1   1   1   1
    87.8619456922    1.0000000000
   12   2   2   1   1
     0.2691413217    1.0000000000
   13   2   2   1   1
     1.0351757346    1.0000000000
   14   2   2   1   1
     2.1764464633    1.0000000000
   15   3   3   1   1
     1.1372702684    1.0000000000

# RI basis set for O (all-electron) relative DI metric: 5.3e-05
O  RI_aug-SZV-MOLOPT-ae_N_RI_054_s_p_d_f_g_h_i_7_6_3_2_0_0_0_error_5.3e-05
   18
    1   0   0   1   1
     0.4306239504    1.0000000000
    2   0   0   1   1
     0.8521884623    1.0000000000
    3   0   0   1   1
     3.0048875724    1.0000000000
    4   0   0   1   1
    10.9228841684    1.0000000000
    5   0   0   1   1
    38.9980478913    1.0000000000
    6   0   0   1   1
   138.1263795543    1.0000000000
    7   0   0   1   1
   488.4072189716    1.0000000000
    8   1   1   1   1
     0.4849605520    1.0000000000
    9   1   1   1   1
     1.2380592592    1.0000000000
   10   1   1   1   1
     3.9119911188    1.0000000000
   11   1   1   1   1
    11.2663991798    1.0000000000
   12   1   1   1   1
    32.5174990955    1.0000000000
   13   1   1   1   1
    93.4456009991    1.0000000000
   14   2   2   1   1
     0.5717318000    1.0000000000
   15   2   2   1   1
     1.7245232247    1.0000000000
   16   2   2   1   1
    18.3774541080    1.0000000000
   17   3   3   1   1
     1.0684357346    1.0000000000
   18   3   3   1   1
     6.0008377772    1.0000000000

# RI basis set for O (all-electron) relative DI metric: 7.1e-06
O  RI_aug-SZV-MOLOPT-ae_N_RI_059_s_p_d_f_g_h_i_7_6_4_2_0_0_0_error_7.1e-06
   19
    1   0   0   1   1
     0.4018141370    1.0000000000
    2   0   0   1   1
     0.8151176898    1.0000000000
    3   0   0   1   1
     2.9845135906    1.0000000000
    4   0   0   1   1
    10.8948352087    1.0000000000
    5   0   0   1   1
    38.9717670113    1.0000000000
```

```
     6   0   0   1   1
   138.0985237091    1.0000000000
     7   0   0   1   1
   488.3768448588    1.0000000000
     8   1   1   1   1
     0.3871827308    1.0000000000
     9   1   1   1   1
     0.9361603476    1.0000000000
    10   1   1   1   1
     3.8535343741    1.0000000000
    11   1   1   1   1
    11.1058914846    1.0000000000
    12   1   1   1   1
    32.3922740999    1.0000000000
    13   1   1   1   1
    93.0342360583    1.0000000000
    14   2   2   1   1
     0.4069747603    1.0000000000
    15   2   2   1   1
     1.3991202086    1.0000000000
    16   2   2   1   1
     4.8723885675    1.0000000000
    17   2   2   1   1
    21.8778375666    1.0000000000
    18   3   3   1   1
     0.7103161145    1.0000000000
    19   3   3   1   1
     2.9755479849    1.0000000000

# RI basis set for O (all-electron) relative DI metric: 4.1e-07
O  RI_aug-SZV-MOLOPT-ae_N_RI_066_s_p_d_f_g_h_i_7_6_4_3_0_0_0_error_4.1e-07
   20
     1   0   0   1   1
     0.3297155479    1.0000000000
     2   0   0   1   1
     0.8940696620    1.0000000000
     3   0   0   1   1
     3.0804002870    1.0000000000
     4   0   0   1   1
    10.9993033773    1.0000000000
     5   0   0   1   1
    39.0725258868    1.0000000000
     6   0   0   1   1
   138.2055622820    1.0000000000
     7   0   0   1   1
   488.4936374930    1.0000000000
     8   1   1   1   1
     0.3473668351    1.0000000000
     9   1   1   1   1
     1.2486865031    1.0000000000
    10   1   1   1   1
     3.6108931111    1.0000000000
    11   1   1   1   1
    10.7042664634    1.0000000000
    12   1   1   1   1
    31.8053629850    1.0000000000
    13   1   1   1   1
    94.1963194348    1.0000000000
    14   2   2   1   1
     0.4481890257    1.0000000000
    15   2   2   1   1
     1.5001354554    1.0000000000
    16   2   2   1   1
     4.5980115325    1.0000000000
    17   2   2   1   1
    21.8516061888    1.0000000000
    18   3   3   1   1
     0.5502182315    1.0000000000
    19   3   3   1   1
     2.0012627133    1.0000000000
    20   3   3   1   1
    13.9368062469    1.0000000000

# RI basis set for O (all-electron) relative DI metric: 1.8e-07
O  RI_aug-SZV-MOLOPT-ae_N_RI_108_s_p_d_f_g_h_i_7_6_6_5_2_0_0_error_1.8e-07
   26
     1   0   0   1   1
     0.3444894440    1.0000000000
     2   0   0   1   1
     0.9091556640    1.0000000000
     3   0   0   1   1
     3.1097707550    1.0000000000
     4   0   0   1   1
    11.0349994141    1.0000000000
     5   0   0   1   1
    39.1010203672    1.0000000000
     6   0   0   1   1
   138.2346100950    1.0000000000
     7   0   0   1   1
   488.5270529402    1.0000000000
     8   1   1   1   1
     0.3479884360    1.0000000000
     9   1   1   1   1
     1.2356196798    1.0000000000
    10   1   1   1   1
     3.6420252560    1.0000000000
    11   1   1   1   1
    10.7695892998    1.0000000000
    12   1   1   1   1
    31.8650126208    1.0000000000
    13   1   1   1   1
    94.2526242359    1.0000000000
    14   2   2   1   1
     0.3066131492    1.0000000000
    15   2   2   1   1
     0.7127228855    1.0000000000
    16   2   2   1   1
     1.6821932472    1.0000000000
    17   2   2   1   1
     3.9623688080    1.0000000000
    18   2   2   1   1
```

```
      9.3333592604    1.0000000000
    19   2   2   1   1
     21.9880801810    1.0000000000
    20   3   3   1   1
      0.4542116367    1.0000000000
    21   3   3   1   1
      1.3698133384    1.0000000000
    22   3   3   1   1
      2.9221467561    1.0000000000
    23   3   3   1   1
      6.4456689307    1.0000000000
    24   3   3   1   1
     14.0842067920    1.0000000000
    25   4   4   1   1
      0.8254236980    1.0000000000
    26   4   4   1   1
      7.0126131855    1.0000000000

# RI basis set for O (all-electron) relative DI metric: 3.1e-02
O  RI_aug-DZVP-MOLOPT-ae_N_RI_018_s_p_d_f_g_h_i_4_3_1_0_0_0_0_error_3.1e-02
   8
     1   0   0   1   1
      0.9328356312    1.0000000000
     2   0   0   1   1
      1.5506774909    1.0000000000
     3   0   0   1   1
     13.5314468592    1.0000000000
     4   0   0   1   1
    155.5873278195    1.0000000000
     5   1   1   1   1
      0.2181701263    1.0000000000
     6   1   1   1   1
      1.7755978336    1.0000000000
     7   1   1   1   1
      4.0055758664    1.0000000000
     8   2   2   1   1
      1.6346574773    1.0000000000

# RI basis set for O (all-electron) relative DI metric: 1.2e-02
O  RI_aug-DZVP-MOLOPT-ae_N_RI_028_s_p_d_f_g_h_i_7_3_1_1_0_0_0_error_1.2e-02
   12
     1   0   0   1   1
      0.1220793427    1.0000000000
     2   0   0   1   1
      0.4050741799    1.0000000000
     3   0   0   1   1
      1.3440781000    1.0000000000
     4   0   0   1   1
      4.4597909937    1.0000000000
     5   0   0   1   1
     14.7980742001    1.0000000000
     6   0   0   1   1
     49.1016267543    1.0000000000
     7   0   0   1   1
    162.9245717276    1.0000000000
     8   1   1   1   1
      0.6321473856    1.0000000000
     9   1   1   1   1
      1.4984171309    1.0000000000
    10   1   1   1   1
     13.7397470116    1.0000000000
    11   2   2   1   1
      1.4945787018    1.0000000000
    12   3   3   1   1
      1.6812064943    1.0000000000

# RI basis set for O (all-electron) relative DI metric: 1.5e-03
O  RI_aug-DZVP-MOLOPT-ae_N_RI_033_s_p_d_f_g_h_i_7_3_2_1_0_0_0_error_1.5e-03
   13
     1   0   0   1   1
      0.1497620584    1.0000000000
     2   0   0   1   1
      0.7329426345    1.0000000000
     3   0   0   1   1
      2.1960703561    1.0000000000
     4   0   0   1   1
      6.3851506859    1.0000000000
     5   0   0   1   1
     18.7927574683    1.0000000000
     6   0   0   1   1
     55.3329110940    1.0000000000
     7   0   0   1   1
    162.8361040391    1.0000000000
     8   1   1   1   1
      0.8535243743    1.0000000000
     9   1   1   1   1
      1.6127280837    1.0000000000
    10   1   1   1   1
     17.3021770677    1.0000000000
    11   2   2   1   1
      0.7678366833    1.0000000000
    12   2   2   1   1
      2.7382382304    1.0000000000
    13   3   3   1   1
      1.2487837087    1.0000000000

# RI basis set for O (all-electron) relative DI metric: 7.2e-04
O  RI_aug-DZVP-MOLOPT-ae_N_RI_041_s_p_d_f_g_h_i_7_4_3_1_0_0_0_error_7.2e-04
   15
     1   0   0   1   1
      0.3102637643    1.0000000000
     2   0   0   1   1
      0.6988249187    1.0000000000
     3   0   0   1   1
      1.9978906213    1.0000000000
     4   0   0   1   1
      6.2273960032    1.0000000000
     5   0   0   1   1
     18.6377528456    1.0000000000
     6   0   0   1   1
     55.1581526291    1.0000000000
```

```
      7   0   0   1   1
    162.6625249591    1.0000000000
      8   1   1   1   1
      0.5071855830    1.0000000000
      9   1   1   1   1
      0.8880256198    1.0000000000
     10   1   1   1   1
      3.5362762975    1.0000000000
     11   1   1   1   1
     13.7879736692    1.0000000000
     12   2   2   1   1
      0.6800692936    1.0000000000
     13   2   2   1   1
      1.3056672699    1.0000000000
     14   2   2   1   1
      5.9981300777    1.0000000000
     15   3   3   1   1
      1.4856489001    1.0000000000

# RI basis set for O (all-electron) relative DI metric: 3.4e-04
O  RI_aug-DZVP-MOLOPT-ae_N_RI_053_s_p_d_f_g_h_i_7_5_3_1_1_0_0_error_3.4e-04
   17
      1   0   0   1   1
      0.3718152050    1.0000000000
      2   0   0   1   1
      0.6674071413    1.0000000000
      3   0   0   1   1
      1.8694101918    1.0000000000
      4   0   0   1   1
      5.9838208307    1.0000000000
      5   0   0   1   1
     18.3626940132    1.0000000000
      6   0   0   1   1
     54.8761232308    1.0000000000
      7   0   0   1   1
    162.3682015385    1.0000000000
      8   1   1   1   1
      0.5824726315    1.0000000000
      9   1   1   1   1
      0.8936294489    1.0000000000
     10   1   1   1   1
      3.9008865880    1.0000000000
     11   1   1   1   1
     10.6489906088    1.0000000000
     12   1   1   1   1
     31.3584687298    1.0000000000
     13   2   2   1   1
      0.4664943564    1.0000000000
     14   2   2   1   1
      1.3889819744    1.0000000000
     15   2   2   1   1
      4.8690725358    1.0000000000
     16   3   3   1   1
      1.4699142238    1.0000000000
     17   4   4   1   1
      0.6192247138    1.0000000000

# RI basis set for O (all-electron) relative DI metric: 1.2e-04
O  RI_aug-DZVP-MOLOPT-ae_N_RI_060_s_p_d_f_g_h_i_7_5_3_2_1_0_0_error_1.2e-04
   18
      1   0   0   1   1
      0.3549500948    1.0000000000
      2   0   0   1   1
      0.6969874540    1.0000000000
      3   0   0   1   1
      1.9443079723    1.0000000000
      4   0   0   1   1
      6.1458644070    1.0000000000
      5   0   0   1   1
     18.5453917573    1.0000000000
      6   0   0   1   1
     55.0606031737    1.0000000000
      7   0   0   1   1
    162.5633421527    1.0000000000
      8   1   1   1   1
      0.3582466802    1.0000000000
      9   1   1   1   1
      0.9365667288    1.0000000000
     10   1   1   1   1
      2.9919506196    1.0000000000
     11   1   1   1   1
     10.1774147209    1.0000000000
     12   1   1   1   1
     30.9816754305    1.0000000000
     13   2   2   1   1
      0.5619392415    1.0000000000
     14   2   2   1   1
      1.1180265993    1.0000000000
     15   2   2   1   1
      4.7851538276    1.0000000000
     16   3   3   1   1
      1.1403391558    1.0000000000
     17   3   3   1   1
      5.4590850252    1.0000000000
     18   4   4   1   1
      0.6879130615    1.0000000000

# RI basis set for O (all-electron) relative DI metric: 4.9e-06
O  RI_aug-DZVP-MOLOPT-ae_N_RI_072_s_p_d_f_g_h_i_7_5_4_3_1_0_0_error_4.9e-06
   20
      1   0   0   1   1
      0.1496626807    1.0000000000
      2   0   0   1   1
      0.3912157152    1.0000000000
      3   0   0   1   1
      1.1732025741    1.0000000000
      4   0   0   1   1
      3.1434209637    1.0000000000
      5   0   0   1   1
     12.5166908479    1.0000000000
      6   0   0   1   1
```

```
      46.9427001845    1.0000000000
      7   0   0   1   1
     160.1633217714    1.0000000000
      8   1   1   1   1
       0.1836459248    1.0000000000
      9   1   1   1   1
       0.6817230727    1.0000000000
     10   1   1   1   1
       1.0774825187    1.0000000000
     11   1   1   1   1
       3.6017502772    1.0000000000
     12   1   1   1   1
      19.1150952675    1.0000000000
     13   2   2   1   1
       0.1540353273    1.0000000000
     14   2   2   1   1
       0.5763654079    1.0000000000
     15   2   2   1   1
       1.2748427615    1.0000000000
     16   2   2   1   1
       4.7632071044    1.0000000000
     17   3   3   1   1
       0.3750085912    1.0000000000
     18   3   3   1   1
       1.3372638464    1.0000000000
     19   3   3   1   1
       5.0641646561    1.0000000000
     20   4   4   1   1
       0.7109823428    1.0000000000

# RI basis set for O (all-electron) relative DI metric: 1.8e-06
O  RI_aug-DZVP-MOLOPT-ae_N_RI_095_s_p_d_f_g_h_i_7_5_5_3_3_0_0_error_1.8e-06
   23
      1   0   0   1   1
       0.1097027319    1.0000000000
      2   0   0   1   1
       0.3697189834    1.0000000000
      3   0   0   1   1
       1.2460230075    1.0000000000
      4   0   0   1   1
       4.1993335400    1.0000000000
      5   0   0   1   1
      14.1525495872    1.0000000000
      6   0   0   1   1
      47.6967733132    1.0000000000
      7   0   0   1   1
     160.7471618104    1.0000000000
      8   1   1   1   1
       0.3206021709    1.0000000000
      9   1   1   1   1
       0.8246135739    1.0000000000
     10   1   1   1   1
       2.1115719050    1.0000000000
     11   1   1   1   1
       8.0690812076    1.0000000000
     12   1   1   1   1
      26.2678310861    1.0000000000
     13   2   2   1   1
       0.3822312671    1.0000000000
     14   2   2   1   1
       0.6741166038    1.0000000000
     15   2   2   1   1
       1.5065321822    1.0000000000
     16   2   2   1   1
       3.3523482080    1.0000000000
     17   2   2   1   1
       7.5938737670    1.0000000000
     18   3   3   1   1
       0.3911089825    1.0000000000
     19   3   3   1   1
       1.2427639346    1.0000000000
     20   3   3   1   1
       4.1683816756    1.0000000000
     21   4   4   1   1
       0.5995007479    1.0000000000
     22   4   4   1   1
       1.4619082563    1.0000000000
     23   4   4   1   1
       2.5977990388    1.0000000000

# RI basis set for O (all-electron) relative DI metric: 4.7e-07
O  RI_aug-DZVP-MOLOPT-ae_N_RI_105_s_p_d_f_g_h_i_7_6_5_4_3_0_0_error_4.7e-07
   25
      1   0   0   1   1
       0.1959047066    1.0000000000
      2   0   0   1   1
       0.4069838382    1.0000000000
      3   0   0   1   1
       1.3148829934    1.0000000000
      4   0   0   1   1
       4.4312553421    1.0000000000
      5   0   0   1   1
      15.5342692827    1.0000000000
      6   0   0   1   1
      51.0052996300    1.0000000000
      7   0   0   1   1
     159.0935411803    1.0000000000
      8   1   1   1   1
       0.2824855427    1.0000000000
      9   1   1   1   1
       0.7563202893    1.0000000000
     10   1   1   1   1
       1.8455324715    1.0000000000
     11   1   1   1   1
       5.1735651800    1.0000000000
     12   1   1   1   1
      12.7023100962    1.0000000000
     13   1   1   1   1
      30.8973273790    1.0000000000
     14   2   2   1   1
       0.3528331722    1.0000000000
```

```
    15   2   2   1   1
      0.6448585305    1.0000000000
    16   2   2   1   1
      1.4712921351    1.0000000000
    17   2   2   1   1
      3.3233438010    1.0000000000
    18   2   2   1   1
      7.7399650681    1.0000000000
    19   3   3   1   1
      0.3458735307    1.0000000000
    20   3   3   1   1
      0.8720072153    1.0000000000
    21   3   3   1   1
      1.8965474790    1.0000000000
    22   3   3   1   1
      4.9420891606    1.0000000000
    23   4   4   1   1
      0.4745012550    1.0000000000
    24   4   4   1   1
      1.4818699783    1.0000000000
    25   4   4   1   1
      3.1881423137    1.0000000000

# RI basis set for O (all-electron) relative DI metric: 3.7e-02
O  RI_aug-TZVP-MOLOPT-ae_N_RI_025_s_p_d_f_g_h_i_4_3_1_1_0_0_0_error_3.7e-02
   9
     1   0   0   1   1
      0.3159497368    1.0000000000
     2   0   0   1   1
      1.9080094003    1.0000000000
     3   0   0   1   1
     12.0863536609    1.0000000000
     4   0   0   1   1
     85.4743209155    1.0000000000
     5   1   1   1   1
      1.1447289943    1.0000000000
     6   1   1   1   1
      1.7370362061    1.0000000000
     7   1   1   1   1
      5.8867329794    1.0000000000
     8   2   2   1   1
      1.7873960573    1.0000000000
     9   3   3   1   1
      1.9137454195    1.0000000000

# RI basis set for O (all-electron) relative DI metric: 1.5e-02
O  RI_aug-TZVP-MOLOPT-ae_N_RI_030_s_p_d_f_g_h_i_4_3_2_1_0_0_0_error_1.5e-02
   10
     1   0   0   1   1
      0.3836435741    1.0000000000
     2   0   0   1   1
      2.5594126600    1.0000000000
     3   0   0   1   1
      7.4632154556    1.0000000000
     4   0   0   1   1
     91.3175615408    1.0000000000
     5   1   1   1   1
      1.0206452060    1.0000000000
     6   1   1   1   1
      1.7214350144    1.0000000000
     7   1   1   1   1
     14.9951730104    1.0000000000
     8   2   2   1   1
      1.1316072317    1.0000000000
     9   2   2   1   1
      2.6036734885    1.0000000000
    10   3   3   1   1
      1.6294342555    1.0000000000

# RI basis set for O (all-electron) relative DI metric: 7.2e-03
O  RI_aug-TZVP-MOLOPT-ae_N_RI_043_s_p_d_f_g_h_i_5_3_3_2_0_0_0_error_7.2e-03
   13
     1   0   0   1   1
      0.3826551011    1.0000000000
     2   0   0   1   1
      0.9359524515    1.0000000000
     3   0   0   1   1
      4.0908552684    1.0000000000
     4   0   0   1   1
     25.4448271199    1.0000000000
     5   0   0   1   1
    124.9878386966    1.0000000000
     6   1   1   1   1
      0.8275527697    1.0000000000
     7   1   1   1   1
      1.4566302904    1.0000000000
     8   1   1   1   1
      5.0967727554    1.0000000000
     9   2   2   1   1
      0.7193864869    1.0000000000
    10   2   2   1   1
      0.9990507562    1.0000000000
    11   2   2   1   1
      4.9509653960    1.0000000000
    12   3   3   1   1
      1.2207689145    1.0000000000
    13   3   3   1   1
      2.3332781584    1.0000000000

# RI basis set for O (all-electron) relative DI metric: 1.9e-03
O  RI_aug-TZVP-MOLOPT-ae_N_RI_052_s_p_d_f_g_h_i_5_3_3_2_1_0_0_error_1.9e-03
   14
     1   0   0   1   1
      0.6224726532    1.0000000000
     2   0   0   1   1
      0.8864793139    1.0000000000
     3   0   0   1   1
      4.3930304324    1.0000000000
     4   0   0   1   1
     27.6990676288    1.0000000000
     5   0   0   1   1
```

```
   157.9594149001    1.0000000000
    6   1   1   1   1
     0.8869673958    1.0000000000
    7   1   1   1   1
     1.8096816585    1.0000000000
    8   1   1   1   1
     3.6895973143    1.0000000000
    9   2   2   1   1
     0.7347315053    1.0000000000
   10   2   2   1   1
     1.3053424402    1.0000000000
   11   2   2   1   1
     5.8324327057    1.0000000000
   12   3   3   1   1
     1.1767995319    1.0000000000
   13   3   3   1   1
     2.9446679376    1.0000000000
   14   4   4   1   1
     1.5453806960    1.0000000000

# RI basis set for O (all-electron) relative DI metric: 9.4e-04
O  RI_aug-TZVP-MOLOPT-ae_N_RI_066_s_p_d_f_g_h_i_7_4_3_2_2_0_0_error_9.4e-04
   18
    1   0   0   1   1
     0.3664566315    1.0000000000
    2   0   0   1   1
     0.7504542396    1.0000000000
    3   0   0   1   1
     2.0700645424    1.0000000000
    4   0   0   1   1
     6.1622128221    1.0000000000
    5   0   0   1   1
    18.4889317327    1.0000000000
    6   0   0   1   1
    54.9641046500    1.0000000000
    7   0   0   1   1
   162.4526354489    1.0000000000
    8   1   1   1   1
     0.6610249346    1.0000000000
    9   1   1   1   1
     1.3825461690    1.0000000000
   10   1   1   1   1
     4.7203891508    1.0000000000
   11   1   1   1   1
    14.1874530366    1.0000000000
   12   2   2   1   1
     0.7205460786    1.0000000000
   13   2   2   1   1
     1.6956656944    1.0000000000
   14   2   2   1   1
     6.6328419698    1.0000000000
   15   3   3   1   1
     0.9970544300    1.0000000000
   16   3   3   1   1
     2.6395400901    1.0000000000
   17   4   4   1   1
     0.9345856656    1.0000000000
   18   4   4   1   1
     2.5102684412    1.0000000000

# RI basis set for O (all-electron) relative DI metric: 3.1e-04
O  RI_aug-TZVP-MOLOPT-ae_N_RI_073_s_p_d_f_g_h_i_7_4_3_3_2_0_0_error_3.1e-04
   19
    1   0   0   1   1
     0.4028338608    1.0000000000
    2   0   0   1   1
     0.7770023228    1.0000000000
    3   0   0   1   1
     2.0395912220    1.0000000000
    4   0   0   1   1
     6.1296286199    1.0000000000
    5   0   0   1   1
    18.4739607257    1.0000000000
    6   0   0   1   1
    54.9397275452    1.0000000000
    7   0   0   1   1
   162.4346617851    1.0000000000
    8   1   1   1   1
     0.6479886354    1.0000000000
    9   1   1   1   1
     1.1025118304    1.0000000000
   10   1   1   1   1
     3.1557134131    1.0000000000
   11   1   1   1   1
    11.8634428380    1.0000000000
   12   2   2   1   1
     0.6123977259    1.0000000000
   13   2   2   1   1
     1.5855240672    1.0000000000
   14   2   2   1   1
     5.7342675391    1.0000000000
   15   3   3   1   1
     0.9349082079    1.0000000000
   16   3   3   1   1
     1.8949417286    1.0000000000
   17   3   3   1   1
     5.2598943277    1.0000000000
   18   4   4   1   1
     0.7509972444    1.0000000000
   19   4   4   1   1
     2.2698060698    1.0000000000

# RI basis set for O (all-electron) relative DI metric: 6.7e-05
O  RI_aug-TZVP-MOLOPT-ae_N_RI_084_s_p_d_f_g_h_i_7_4_3_3_2_1_0_error_6.7e-05
   20
    1   0   0   1   1
     0.3949766272    1.0000000000
    2   0   0   1   1
     0.7581774727    1.0000000000
    3   0   0   1   1
     2.0199862314    1.0000000000
```

```
     4   0   0   1   1
      6.1337338090    1.0000000000
     5   0   0   1   1
     18.5098503864    1.0000000000
     6   0   0   1   1
     54.9930713327    1.0000000000
     7   0   0   1   1
    162.4863814100    1.0000000000
     8   1   1   1   1
      0.5925925190    1.0000000000
     9   1   1   1   1
      1.3028561220    1.0000000000
    10   1   1   1   1
      4.3158054194    1.0000000000
    11   1   1   1   1
     16.1875328927    1.0000000000
    12   2   2   1   1
      0.6221928500    1.0000000000
    13   2   2   1   1
      1.5767629247    1.0000000000
    14   2   2   1   1
      5.8316696219    1.0000000000
    15   3   3   1   1
      0.7488202199    1.0000000000
    16   3   3   1   1
      1.8506053252    1.0000000000
    17   3   3   1   1
      5.2990354846    1.0000000000
    18   4   4   1   1
      0.7667622853    1.0000000000
    19   4   4   1   1
      2.2477344885    1.0000000000
    20   5   5   1   1
      0.7626379089    1.0000000000

# RI basis set for O (all-electron) relative DI metric: 2.7e-05
O  RI_aug-TZVP-MOLOPT-ae_N_RI_089_s_p_d_f_g_h_i_7_4_4_3_2_1_0_error_2.7e-05
   21
     1   0   0   1   1
      0.4017847294    1.0000000000
     2   0   0   1   1
      0.7444561861    1.0000000000
     3   0   0   1   1
      1.9469061335    1.0000000000
     4   0   0   1   1
      5.9134645827    1.0000000000
     5   0   0   1   1
     18.2203034196    1.0000000000
     6   0   0   1   1
     54.6292790459    1.0000000000
     7   0   0   1   1
    162.0797813885    1.0000000000
     8   1   1   1   1
      0.5278913903    1.0000000000
     9   1   1   1   1
      1.1421772582    1.0000000000
    10   1   1   1   1
      3.5062547744    1.0000000000
    11   1   1   1   1
     12.6388058988    1.0000000000
    12   2   2   1   1
      0.5329289356    1.0000000000
    13   2   2   1   1
      0.9321110145    1.0000000000
    14   2   2   1   1
      1.8623498347    1.0000000000
    15   2   2   1   1
      6.4023325580    1.0000000000
    16   3   3   1   1
      0.6849803652    1.0000000000
    17   3   3   1   1
      1.8640679255    1.0000000000
    18   3   3   1   1
      5.5952946013    1.0000000000
    19   4   4   1   1
      0.7204421703    1.0000000000
    20   4   4   1   1
      2.3840956509    1.0000000000
    21   5   5   1   1
      0.8731087504    1.0000000000

# RI basis set for O (all-electron) relative DI metric: 1.2e-05
O  RI_aug-TZVP-MOLOPT-ae_N_RI_100_s_p_d_f_g_h_i_7_6_5_3_2_1_0_error_1.2e-05
   24
     1   0   0   1   1
      0.1283798277    1.0000000000
     2   0   0   1   1
      0.4068917904    1.0000000000
     3   0   0   1   1
      1.1298130974    1.0000000000
     4   0   0   1   1
      3.4393321793    1.0000000000
     5   0   0   1   1
     12.5397358651    1.0000000000
     6   0   0   1   1
     42.3199373137    1.0000000000
     7   0   0   1   1
    137.8789285876    1.0000000000
     8   1   1   1   1
      0.5285339812    1.0000000000
     9   1   1   1   1
      0.9341915996    1.0000000000
    10   1   1   1   1
      2.2580857341    1.0000000000
    11   1   1   1   1
      6.0159515648    1.0000000000
    12   1   1   1   1
     13.9073871590    1.0000000000
    13   1   1   1   1
     32.5899309186    1.0000000000
    14   2   2   1   1
```

```
      0.3467431720    1.0000000000
    15   2   2   1   1
      0.5542540736    1.0000000000
    16   2   2   1   1
      1.5427172866    1.0000000000
    17   2   2   1   1
      3.2993340623    1.0000000000
    18   2   2   1   1
      7.7874555396    1.0000000000
    19   3   3   1   1
      0.6599880739    1.0000000000
    20   3   3   1   1
      1.9281436686    1.0000000000
    21   3   3   1   1
      5.7804841272    1.0000000000
    22   4   4   1   1
      0.7256545547    1.0000000000
    23   4   4   1   1
      2.4172382787    1.0000000000
    24   5   5   1   1
      0.7711588260    1.0000000000

# RI basis set for O (all-electron) relative DI metric: 5.1e-06
O  RI_aug-TZVP-MOLOPT-ae_N_RI_119_s_p_d_f_g_h_i_7_6_6_5_2_1_0_error_5.1e-06
   27
     1   0   0   1   1
      0.3131338435    1.0000000000
     2   0   0   1   1
      0.7963612792    1.0000000000
     3   0   0   1   1
      1.2625319900    1.0000000000
     4   0   0   1   1
      3.4547474958    1.0000000000
     5   0   0   1   1
     11.9711403724    1.0000000000
     6   0   0   1   1
     41.2099877111    1.0000000000
     7   0   0   1   1
    127.2855805943    1.0000000000
     8   1   1   1   1
      0.4811550391    1.0000000000
     9   1   1   1   1
      0.9146961399    1.0000000000
    10   1   1   1   1
      2.0907347044    1.0000000000
    11   1   1   1   1
      5.2554856193    1.0000000000
    12   1   1   1   1
     12.8345181734    1.0000000000
    13   1   1   1   1
     30.9368078987    1.0000000000
    14   2   2   1   1
      0.3036284851    1.0000000000
    15   2   2   1   1
      0.5697519005    1.0000000000
    16   2   2   1   1
      1.0839964710    1.0000000000
    17   2   2   1   1
      2.0567654326    1.0000000000
    18   2   2   1   1
      3.9245649170    1.0000000000
    19   2   2   1   1
      7.6652591959    1.0000000000
    20   3   3   1   1
      0.6184939858    1.0000000000
    21   3   3   1   1
      1.0221331961    1.0000000000
    22   3   3   1   1
      1.7166591598    1.0000000000
    23   3   3   1   1
      2.8843071836    1.0000000000
    24   3   3   1   1
      5.0938068974    1.0000000000
    25   4   4   1   1
      0.6932411426    1.0000000000
    26   4   4   1   1
      2.1791102198    1.0000000000
    27   5   5   1   1
      0.9226622435    1.0000000000

# RI basis set for F (all-electron) relative DI metric: 4.4e-02
F  RI_aug-SZV-MOLOPT-ae-mini_N_RI_006_s_p_d_f_g_h_i_3_1_0_0_0_0_0_error_4.4e-02
   4
     1   0   0   1   1
      0.3986587783    1.0000000000
     2   0   0   1   1
      3.0160515346    1.0000000000
     3   0   0   1   1
     22.8178693395    1.0000000000
     4   1   1   1   1
      4.3689624046    1.0000000000

# RI basis set for F (all-electron) relative DI metric: 4.7e-03
F  RI_aug-SZV-MOLOPT-ae-mini_N_RI_011_s_p_d_f_g_h_i_3_1_1_0_0_0_0_error_4.7e-03
   5
     1   0   0   1   1
      0.1279253598    1.0000000000
     2   0   0   1   1
      0.7319554190    1.0000000000
     3   0   0   1   1
      2.6436461934    1.0000000000
     4   1   1   1   1
      4.3046185902    1.0000000000
     5   2   2   1   1
      4.9651244178    1.0000000000

# RI basis set for F (all-electron) relative DI metric: 2.1e-03
F  RI_aug-SZV-MOLOPT-ae-mini_N_RI_018_s_p_d_f_g_h_i_4_3_1_0_0_0_0_error_2.1e-03
   8
     1   0   0   1   1
      0.4950434129    1.0000000000
```

```
      2   0   0   1   1
       1.3374532133    1.0000000000
      3   0   0   1   1
       3.7086032862    1.0000000000
      4   0   0   1   1
       9.3142678407    1.0000000000
      5   1   1   1   1
       0.2899636126    1.0000000000
      6   1   1   1   1
       0.7202797545    1.0000000000
      7   1   1   1   1
       1.8459653565    1.0000000000
      8   2   2   1   1
       5.0043452160    1.0000000000

# RI basis set for F (all-electron) relative DI metric: 4.7e-06
F  RI_aug-SZV-MOLOPT-ae-mini_N_RI_023_s_p_d_f_g_h_i_4_3_2_0_0_0_0_error_4.7e-06
    9
      1   0   0   1   1
       0.2764689957    1.0000000000
      2   0   0   1   1
       1.1287583543    1.0000000000
      3   0   0   1   1
       3.1187188899    1.0000000000
      4   0   0   1   1
      14.0999379137    1.0000000000
      5   1   1   1   1
       0.2765665388    1.0000000000
      6   1   1   1   1
       0.9651991371    1.0000000000
      7   1   1   1   1
       2.9083702575    1.0000000000
      8   2   2   1   1
       0.2992894257    1.0000000000
      9   2   2   1   1
       3.6943557033    1.0000000000

# RI basis set for F (all-electron) relative DI metric: 2.2e-06
F  RI_aug-SZV-MOLOPT-ae-mini_N_RI_024_s_p_d_f_g_h_i_5_3_2_0_0_0_0_error_2.2e-06
    10
      1   0   0   1   1
       0.1916936098    1.0000000000
      2   0   0   1   1
       0.5035308800    1.0000000000
      3   0   0   1   1
       1.3420008444    1.0000000000
      4   0   0   1   1
       3.8587634671    1.0000000000
      5   0   0   1   1
      10.4922469210    1.0000000000
      6   1   1   1   1
       0.2348395795    1.0000000000
      7   1   1   1   1
       0.6630414405    1.0000000000
      8   1   1   1   1
       2.7118112576    1.0000000000
      9   2   2   1   1
       0.3163212941    1.0000000000
     10   2   2   1   1
       3.6395284305    1.0000000000

# RI basis set for F (all-electron) relative DI metric: 1.0e-06
F  RI_aug-SZV-MOLOPT-ae-mini_N_RI_029_s_p_d_f_g_h_i_5_3_3_0_0_0_0_error_1.0e-06
    11
      1   0   0   1   1
       0.1867626678    1.0000000000
      2   0   0   1   1
       0.5123007160    1.0000000000
      3   0   0   1   1
       1.4054173519    1.0000000000
      4   0   0   1   1
       3.8574599408    1.0000000000
      5   0   0   1   1
      10.5837003415    1.0000000000
      6   1   1   1   1
       0.2258453930    1.0000000000
      7   1   1   1   1
       1.0012977094    1.0000000000
      8   1   1   1   1
       2.6237607561    1.0000000000
      9   2   2   1   1
       0.3569649907    1.0000000000
     10   2   2   1   1
       2.7725110964    1.0000000000
     11   2   2   1   1
       6.4569329891    1.0000000000

# RI basis set for F (all-electron) relative DI metric: 2.5e-07
F  RI_aug-SZV-MOLOPT-ae-mini_N_RI_033_s_p_d_f_g_h_i_6_4_3_0_0_0_0_error_2.5e-07
    13
      1   0   0   1   1
       0.2462927166    1.0000000000
      2   0   0   1   1
       0.5874459515    1.0000000000
      3   0   0   1   1
       1.2576438658    1.0000000000
      4   0   0   1   1
       2.4905891837    1.0000000000
      5   0   0   1   1
       5.2139451668    1.0000000000
      6   0   0   1   1
      10.8106111651    1.0000000000
      7   1   1   1   1
       0.2263238677    1.0000000000
      8   1   1   1   1
       0.8524051058    1.0000000000
      9   1   1   1   1
       2.3253111498    1.0000000000
     10   1   1   1   1
       9.6455125989    1.0000000000
     11   2   2   1   1
```

```
      0.3591757129    1.0000000000
    12   2   2   1   1
      2.8184792026    1.0000000000
    13   2   2   1   1
      6.3411194253    1.0000000000

# RI basis set for F (all-electron) relative DI metric: 5.6e-08
F  RI_aug-SZV-MOLOPT-ae-mini_N_RI_038_s_p_d_f_g_h_i_6_4_4_0_0_0_0_error_5.6e-08
   14
     1   0   0   1   1
      0.2315981922    1.0000000000
     2   0   0   1   1
      0.5555718186    1.0000000000
     3   0   0   1   1
      1.1820389897    1.0000000000
     4   0   0   1   1
      2.3379645952    1.0000000000
     5   0   0   1   1
      4.9296943760    1.0000000000
     6   0   0   1   1
     10.2666977424    1.0000000000
     7   1   1   1   1
      0.2328019585    1.0000000000
     8   1   1   1   1
      0.7321477130    1.0000000000
     9   1   1   1   1
      2.3233821814    1.0000000000
    10   1   1   1   1
      7.5282429909    1.0000000000
    11   2   2   1   1
      0.2892024598    1.0000000000
    12   2   2   1   1
      0.7946116868    1.0000000000
    13   2   2   1   1
      2.1898049193    1.0000000000
    14   2   2   1   1
      6.1676280497    1.0000000000

# RI basis set for F (all-electron) relative DI metric: 2.7e-08
F  RI_aug-SZV-MOLOPT-ae-mini_N_RI_055_s_p_d_f_g_h_i_7_6_6_0_0_0_0_error_2.7e-08
   19
     1   0   0   1   1
      0.2618568441    1.0000000000
     2   0   0   1   1
      0.5199828907    1.0000000000
     3   0   0   1   1
      0.9653180694    1.0000000000
     4   0   0   1   1
      1.7575091726    1.0000000000
     5   0   0   1   1
      3.2381875512    1.0000000000
     6   0   0   1   1
      5.9025492331    1.0000000000
     7   0   0   1   1
     10.7311638508    1.0000000000
     8   1   1   1   1
      0.2672708372    1.0000000000
     9   1   1   1   1
      0.5971999147    1.0000000000
    10   1   1   1   1
      1.2483042967    1.0000000000
    11   1   1   1   1
      2.5367771077    1.0000000000
    12   1   1   1   1
      5.2601292756    1.0000000000
    13   1   1   1   1
     10.7387089968    1.0000000000
    14   2   2   1   1
      0.3031706390    1.0000000000
    15   2   2   1   1
      0.6132576796    1.0000000000
    16   2   2   1   1
      1.2574188041    1.0000000000
    17   2   2   1   1
      2.5795362368    1.0000000000
    18   2   2   1   1
      5.2608491523    1.0000000000
    19   2   2   1   1
     10.7840477353    1.0000000000

# RI basis set for F (all-electron) relative DI metric: 3.9e-02
F  RI_aug-SZV-MOLOPT-ae_N_RI_012_s_p_d_f_g_h_i_4_1_1_0_0_0_0_error_3.9e-02
   6
     1   0   0   1   1
      3.5854059760    1.0000000000
     2   0   0   1   1
     20.8319571411    1.0000000000
     3   0   0   1   1
    112.1267364004    1.0000000000
     4   0   0   1   1
    585.5488448476    1.0000000000
     5   1   1   1   1
      3.0999836020    1.0000000000
     6   2   2   1   1
      2.1527687985    1.0000000000

# RI basis set for F (all-electron) relative DI metric: 5.2e-03
F  RI_aug-SZV-MOLOPT-ae_N_RI_019_s_p_d_f_g_h_i_4_1_1_1_0_0_0_error_5.2e-03
   7
     1   0   0   1   1
      3.8624774903    1.0000000000
     2   0   0   1   1
      9.8812079622    1.0000000000
     3   0   0   1   1
     98.8395944295    1.0000000000
     4   0   0   1   1
    628.5921928450    1.0000000000
     5   1   1   1   1
      1.9099929077    1.0000000000
     6   2   2   1   1
      2.4044416007    1.0000000000
```

```
     7   3   3   1   1
      3.0370656606    1.0000000000

# RI basis set for F (all-electron) relative DI metric: 1.0e-03
F  RI_aug-SZV-MOLOPT-ae_N_RI_022_s_p_d_f_g_h_i_7_1_1_1_0_0_0_error_1.0e-03
   10
     1   0   0   1   1
      0.2620157456    1.0000000000
     2   0   0   1   1
      0.9038810068    1.0000000000
     3   0   0   1   1
      3.6732689768    1.0000000000
     4   0   0   1   1
     13.2821749389    1.0000000000
     5   0   0   1   1
     48.2756333416    1.0000000000
     6   0   0   1   1
    176.7683639381    1.0000000000
     7   0   0   1   1
    648.3130266412    1.0000000000
     8   1   1   1   1
      2.0447469562    1.0000000000
     9   2   2   1   1
      2.8337282919    1.0000000000
    10   3   3   1   1
      3.2504863578    1.0000000000

# RI basis set for F (all-electron) relative DI metric: 2.4e-04
F  RI_aug-SZV-MOLOPT-ae_N_RI_034_s_p_d_f_g_h_i_7_5_1_1_0_0_0_error_2.4e-04
   14
     1   0   0   1   1
      0.2251974544    1.0000000000
     2   0   0   1   1
      1.2438582221    1.0000000000
     3   0   0   1   1
      4.9407129322    1.0000000000
     4   0   0   1   1
     16.2718480208    1.0000000000
     5   0   0   1   1
     53.1136264241    1.0000000000
     6   0   0   1   1
    183.1619845578    1.0000000000
     7   0   0   1   1
    649.1502088954    1.0000000000
     8   1   1   1   1
      0.1706281109    1.0000000000
     9   1   1   1   1
      2.2320714859    1.0000000000
    10   1   1   1   1
      6.2833667184    1.0000000000
    11   1   1   1   1
     27.5992244058    1.0000000000
    12   1   1   1   1
    113.0164103075    1.0000000000
    13   2   2   1   1
      2.8126347756    1.0000000000
    14   3   3   1   1
      3.0726651097    1.0000000000

# RI basis set for F (all-electron) relative DI metric: 6.9e-05
F  RI_aug-SZV-MOLOPT-ae_N_RI_046_s_p_d_f_g_h_i_7_6_1_1_1_0_0_error_6.9e-05
   16
     1   0   0   1   1
      0.1869045720    1.0000000000
     2   0   0   1   1
      0.5953843079    1.0000000000
     3   0   0   1   1
      2.7262606173    1.0000000000
     4   0   0   1   1
     10.1289779799    1.0000000000
     5   0   0   1   1
     39.7367152845    1.0000000000
     6   0   0   1   1
    159.8775367650    1.0000000000
     7   0   0   1   1
    645.7792900582    1.0000000000
     8   1   1   1   1
      0.1282691475    1.0000000000
     9   1   1   1   1
      0.5027577167    1.0000000000
    10   1   1   1   1
      1.9705876232    1.0000000000
    11   1   1   1   1
      7.7238142530    1.0000000000
    12   1   1   1   1
     30.2738983157    1.0000000000
    13   1   1   1   1
    118.6601463039    1.0000000000
    14   2   2   1   1
      2.7736907456    1.0000000000
    15   3   3   1   1
      3.0723897758    1.0000000000
    16   4   4   1   1
      0.3922152109    1.0000000000

# RI basis set for F (all-electron) relative DI metric: 1.4e-05
F  RI_aug-SZV-MOLOPT-ae_N_RI_063_s_p_d_f_g_h_i_7_6_3_2_1_0_0_error_1.4e-05
   19
     1   0   0   1   1
      0.1265779537    1.0000000000
     2   0   0   1   1
      0.5251361256    1.0000000000
     3   0   0   1   1
      2.1786411482    1.0000000000
     4   0   0   1   1
      9.0385655305    1.0000000000
     5   0   0   1   1
     37.4984502837    1.0000000000
     6   0   0   1   1
    155.5704571654    1.0000000000
     7   0   0   1   1
```

```
     645.4177961781    1.0000000000
      8   1   1   1   1
       0.2091824598    1.0000000000
      9   1   1   1   1
       1.7060491582    1.0000000000
     10   1   1   1   1
       4.5892205336    1.0000000000
     11   1   1   1   1
      13.5952358811    1.0000000000
     12   1   1   1   1
      40.0448606759    1.0000000000
     13   1   1   1   1
     118.0078814356    1.0000000000
     14   2   2   1   1
       0.2102478424    1.0000000000
     15   2   2   1   1
       2.6056265131    1.0000000000
     16   2   2   1   1
      27.3328854445    1.0000000000
     17   3   3   1   1
       2.8062216486    1.0000000000
     18   3   3   1   1
      15.5201615747    1.0000000000
     19   4   4   1   1
       2.6607386482    1.0000000000

# RI basis set for F (all-electron) relative DI metric: 5.8e-06
F  RI_aug-SZV-MOLOPT-ae_N_RI_068_s_p_d_f_g_h_i_7_6_4_2_1_0_0_error_5.8e-06
   20
      1   0   0   1   1
       0.1273461299    1.0000000000
      2   0   0   1   1
       0.5277846691    1.0000000000
      3   0   0   1   1
       2.1873976355    1.0000000000
      4   0   0   1   1
       9.0656451716    1.0000000000
      5   0   0   1   1
      37.5724653542    1.0000000000
      6   0   0   1   1
     155.7186629476    1.0000000000
      7   0   0   1   1
     645.3742588073    1.0000000000
      8   1   1   1   1
       0.1733687741    1.0000000000
      9   1   1   1   1
       1.8020781250    1.0000000000
     10   1   1   1   1
       4.5697814534    1.0000000000
     11   1   1   1   1
      13.6080961367    1.0000000000
     12   1   1   1   1
      40.0348599916    1.0000000000
     13   1   1   1   1
     117.9902489619    1.0000000000
     14   2   2   1   1
       0.2020930960    1.0000000000
     15   2   2   1   1
       1.8948302696    1.0000000000
     16   2   2   1   1
       5.2882739943    1.0000000000
     17   2   2   1   1
      27.2509249323    1.0000000000
     18   3   3   1   1
       2.7382996183    1.0000000000
     19   3   3   1   1
      12.7873935108    1.0000000000
     20   4   4   1   1
       2.7452017599    1.0000000000

# RI basis set for F (all-electron) relative DI metric: 1.3e-06
F  RI_aug-SZV-MOLOPT-ae_N_RI_075_s_p_d_f_g_h_i_7_6_4_3_1_0_0_error_1.3e-06
   21
      1   0   0   1   1
       0.1076819874    1.0000000000
      2   0   0   1   1
       0.4589294939    1.0000000000
      3   0   0   1   1
       1.9559100414    1.0000000000
      4   0   0   1   1
       8.3358862975    1.0000000000
      5   0   0   1   1
      35.5266852627    1.0000000000
      6   0   0   1   1
     151.4110582950    1.0000000000
      7   0   0   1   1
     645.2982709970    1.0000000000
      8   1   1   1   1
       0.1539364329    1.0000000000
      9   1   1   1   1
       1.6972930281    1.0000000000
     10   1   1   1   1
       4.0924579602    1.0000000000
     11   1   1   1   1
      13.1240621862    1.0000000000
     12   1   1   1   1
      39.5840900898    1.0000000000
     13   1   1   1   1
     117.5863446097    1.0000000000
     14   2   2   1   1
       0.1946960151    1.0000000000
     15   2   2   1   1
       1.9520296194    1.0000000000
     16   2   2   1   1
       4.5282338068    1.0000000000
     17   2   2   1   1
      26.1967893294    1.0000000000
     18   3   3   1   1
       1.7491811726    1.0000000000
     19   3   3   1   1
       4.0555527857    1.0000000000
```

```
    20   3   3   1   1
     16.5459588973    1.0000000000
    21   4   4   1   1
      2.8362867058    1.0000000000

# RI basis set for F (all-electron) relative DI metric: 4.0e-07
F  RI_aug-SZV-MOLOPT-ae_N_RI_119_s_p_d_f_g_h_i_7_6_4_3_2_2_1_error_4.0e-07
   25
     1   0   0   1   1
      0.0999352621    1.0000000000
     2   0   0   1   1
      0.4312563940    1.0000000000
     3   0   0   1   1
      1.8610255419    1.0000000000
     4   0   0   1   1
      8.0309906794    1.0000000000
     5   0   0   1   1
     34.6565964643    1.0000000000
     6   0   0   1   1
    149.5556059902    1.0000000000
     7   0   0   1   1
    645.3859167967    1.0000000000
     8   1   1   1   1
      0.1254507653    1.0000000000
     9   1   1   1   1
      0.4935945977    1.0000000000
    10   1   1   1   1
      1.9420816346    1.0000000000
    11   1   1   1   1
      7.6412526276    1.0000000000
    12   1   1   1   1
     30.0650298305    1.0000000000
    13   1   1   1   1
    118.2929113780    1.0000000000
    14   2   2   1   1
      0.1617308436    1.0000000000
    15   2   2   1   1
      0.9081656026    1.0000000000
    16   2   2   1   1
      2.9715009758    1.0000000000
    17   2   2   1   1
     24.9201422799    1.0000000000
    18   3   3   1   1
      1.6245382294    1.0000000000
    19   3   3   1   1
      3.8443374904    1.0000000000
    20   3   3   1   1
     17.8171763280    1.0000000000
    21   4   4   1   1
      2.1558144487    1.0000000000
    22   4   4   1   1
      8.6849765440    1.0000000000
    23   5   5   1   1
      1.8465706996    1.0000000000
    24   5   5   1   1
      7.0302112039    1.0000000000
    25   6   6   1   1
      3.2327123206    1.0000000000

# RI basis set for F (all-electron) relative DI metric: 1.9e-02
F  RI_aug-DZVP-MOLOPT-ae_N_RI_023_s_p_d_f_g_h_i_5_2_1_1_0_0_0_error_1.9e-02
   9
     1   0   0   1   1
      0.1546848844    1.0000000000
     2   0   0   1   1
      1.2541172930    1.0000000000
     3   0   0   1   1
      9.6258760820    1.0000000000
     4   0   0   1   1
     79.2528777700    1.0000000000
     5   0   0   1   1
    644.6015124074    1.0000000000
     6   1   1   1   1
      0.5369775865    1.0000000000
     7   1   1   1   1
      2.5756787251    1.0000000000
     8   2   2   1   1
      1.5633010807    1.0000000000
     9   3   3   1   1
      3.7676917266    1.0000000000

# RI basis set for F (all-electron) relative DI metric: 5.6e-03
F  RI_aug-DZVP-MOLOPT-ae_N_RI_033_s_p_d_f_g_h_i_6_2_1_1_1_0_0_error_5.6e-03
   11
     1   0   0   1   1
      0.4109703543    1.0000000000
     2   0   0   1   1
      1.9260955779    1.0000000000
     3   0   0   1   1
      9.9674671030    1.0000000000
     4   0   0   1   1
     42.5346836657    1.0000000000
     5   0   0   1   1
    166.9640714979    1.0000000000
     6   0   0   1   1
    643.1999962503    1.0000000000
     7   1   1   1   1
      0.8499117327    1.0000000000
     8   1   1   1   1
      2.3454546434    1.0000000000
     9   2   2   1   1
      2.1615578696    1.0000000000
    10   3   3   1   1
      1.5817105756    1.0000000000
    11   4   4   1   1
      0.7757779476    1.0000000000

# RI basis set for F (all-electron) relative DI metric: 2.2e-04
F  RI_aug-DZVP-MOLOPT-ae_N_RI_047_s_p_d_f_g_h_i_7_3_3_1_1_0_0_error_2.2e-04
   15
     1   0   0   1   1
```

```
      0.1226175883    1.0000000000
     2   0   0   1   1
      0.5108338046    1.0000000000
     3   0   0   1   1
      2.1281659468    1.0000000000
     4   0   0   1   1
      8.8660937228    1.0000000000
     5   0   0   1   1
     36.9367665273    1.0000000000
     6   0   0   1   1
    153.8811580829    1.0000000000
     7   0   0   1   1
    641.0796838069    1.0000000000
     8   1   1   1   1
      0.8296489154    1.0000000000
     9   1   1   1   1
      1.9070313845    1.0000000000
    10   1   1   1   1
     18.2438595579    1.0000000000
    11   2   2   1   1
      0.8230937079    1.0000000000
    12   2   2   1   1
      1.8915176327    1.0000000000
    13   2   2   1   1
     10.9206168359    1.0000000000
    14   3   3   1   1
      1.5360633264    1.0000000000
    15   4   4   1   1
      1.1943211072    1.0000000000

# RI basis set for F (all-electron) relative DI metric: 8.1e-05
F  RI_aug-DZVP-MOLOPT-ae_N_RI_061_s_p_d_f_g_h_i_7_3_3_3_1_0_0_error_8.1e-05
   17
     1   0   0   1   1
      0.1438459015    1.0000000000
     2   0   0   1   1
      0.5837038778    1.0000000000
     3   0   0   1   1
      2.3685848869    1.0000000000
     4   0   0   1   1
      9.6114127798    1.0000000000
     5   0   0   1   1
     39.0017535550    1.0000000000
     6   0   0   1   1
    158.2635637820    1.0000000000
     7   0   0   1   1
    642.2109774982    1.0000000000
     8   1   1   1   1
      0.6193724014    1.0000000000
     9   1   1   1   1
      2.1105205779    1.0000000000
    10   1   1   1   1
     42.2243789251    1.0000000000
    11   2   2   1   1
      0.8040448193    1.0000000000
    12   2   2   1   1
      2.3949360699    1.0000000000
    13   2   2   1   1
     15.9800470118    1.0000000000
    14   3   3   1   1
      0.8263266054    1.0000000000
    15   3   3   1   1
      2.5770848054    1.0000000000
    16   3   3   1   1
     14.8227094815    1.0000000000
    17   4   4   1   1
      1.2312585162    1.0000000000

# RI basis set for F (all-electron) relative DI metric: 3.8e-05
F  RI_aug-DZVP-MOLOPT-ae_N_RI_085_s_p_d_f_g_h_i_7_5_3_3_3_0_0_error_3.8e-05
   21
     1   0   0   1   1
      0.5524583813    1.0000000000
     2   0   0   1   1
      0.9263584076    1.0000000000
     3   0   0   1   1
      3.7536498831    1.0000000000
     4   0   0   1   1
     13.8696363383    1.0000000000
     5   0   0   1   1
     49.9651424297    1.0000000000
     6   0   0   1   1
    179.6341779215    1.0000000000
     7   0   0   1   1
    645.4317311778    1.0000000000
     8   1   1   1   1
      0.5663310069    1.0000000000
     9   1   1   1   1
      1.9096335923    1.0000000000
    10   1   1   1   1
      7.9373702022    1.0000000000
    11   1   1   1   1
     30.6738425675    1.0000000000
    12   1   1   1   1
    117.8378902033    1.0000000000
    13   2   2   1   1
      0.7277870184    1.0000000000
    14   2   2   1   1
      2.2749287956    1.0000000000
    15   2   2   1   1
     18.0804414265    1.0000000000
    16   3   3   1   1
      0.8552730141    1.0000000000
    17   3   3   1   1
      2.9579747598    1.0000000000
    18   3   3   1   1
     18.2771614526    1.0000000000
    19   4   4   1   1
      0.9760164692    1.0000000000
    20   4   4   1   1
      3.2508921749    1.0000000000
```

```
    21   4   4   1   1
      8.8641110940    1.0000000000

# RI basis set for F (all-electron) relative DI metric: 8.6e-06
F  RI_aug-DZVP-MOLOPT-ae_N_RI_093_s_p_d_f_g_h_i_7_6_4_3_3_0_0_error_8.6e-06
   23
     1   0   0   1   1
      0.4183635401    1.0000000000
     2   0   0   1   1
      0.7880773919    1.0000000000
     3   0   0   1   1
      3.4724805828    1.0000000000
     4   0   0   1   1
     13.7222021893    1.0000000000
     5   0   0   1   1
     49.8316661864    1.0000000000
     6   0   0   1   1
    179.4244621688    1.0000000000
     7   0   0   1   1
    645.2009340832    1.0000000000
     8   1   1   1   1
      0.4643989338    1.0000000000
     9   1   1   1   1
      1.1283761006    1.0000000000
    10   1   1   1   1
      4.2101445049    1.0000000000
    11   1   1   1   1
     12.9597536034    1.0000000000
    12   1   1   1   1
     39.0908703453    1.0000000000
    13   1   1   1   1
    117.7340873733    1.0000000000
    14   2   2   1   1
      0.5769420601    1.0000000000
    15   2   2   1   1
      1.4888738391    1.0000000000
    16   2   2   1   1
      5.3127375291    1.0000000000
    17   2   2   1   1
     27.9781305297    1.0000000000
    18   3   3   1   1
      0.8741840982    1.0000000000
    19   3   3   1   1
      2.7494310713    1.0000000000
    20   3   3   1   1
     18.1923285565    1.0000000000
    21   4   4   1   1
      1.0268396436    1.0000000000
    22   4   4   1   1
      2.8684446433    1.0000000000
    23   4   4   1   1
      8.7621378759    1.0000000000

# RI basis set for F (all-electron) relative DI metric: 2.8e-06
F  RI_aug-DZVP-MOLOPT-ae_N_RI_105_s_p_d_f_g_h_i_7_6_5_4_3_0_0_error_2.8e-06
   25
     1   0   0   1   1
      0.3295678595    1.0000000000
     2   0   0   1   1
      0.7409205501    1.0000000000
     3   0   0   1   1
      2.8392424897    1.0000000000
     4   0   0   1   1
     13.2708394753    1.0000000000
     5   0   0   1   1
     49.3914335416    1.0000000000
     6   0   0   1   1
    178.7905615507    1.0000000000
     7   0   0   1   1
    644.4925982230    1.0000000000
     8   1   1   1   1
      0.3363577921    1.0000000000
     9   1   1   1   1
      0.9280791570    1.0000000000
    10   1   1   1   1
      3.2177943742    1.0000000000
    11   1   1   1   1
     10.9576189577    1.0000000000
    12   1   1   1   1
     35.7099363336    1.0000000000
    13   1   1   1   1
    116.3930308876    1.0000000000
    14   2   2   1   1
      0.4115051921    1.0000000000
    15   2   2   1   1
      0.9070990533    1.0000000000
    16   2   2   1   1
      2.9144130953    1.0000000000
    17   2   2   1   1
      8.9806924875    1.0000000000
    18   2   2   1   1
     27.7586310253    1.0000000000
    19   3   3   1   1
      0.8019124675    1.0000000000
    20   3   3   1   1
      2.1235315495    1.0000000000
    21   3   3   1   1
      6.3265861866    1.0000000000
    22   3   3   1   1
     18.4503214145    1.0000000000
    23   4   4   1   1
      1.0454999788    1.0000000000
    24   4   4   1   1
      2.6364131258    1.0000000000
    25   4   4   1   1
      8.2125135635    1.0000000000


# RI basis set for F (all-electron) relative DI metric: 4.5e-02
F  RI_aug-TZVP-MOLOPT-ae_N_RI_025_s_p_d_f_g_h_i_4_3_1_1_0_0_0_error_4.5e-02
```

```
   9
     1   0   0   1   1
       0.6043351338    1.0000000000
     2   0   0   1   1
       4.1263259368    1.0000000000
     3   0   0   1   1
      28.1854824009    1.0000000000
     4   0   0   1   1
     192.5063246533    1.0000000000
     5   1   1   1   1
       1.5794244059    1.0000000000
     6   1   1   1   1
       3.2236124028    1.0000000000
     7   1   1   1   1
      17.3100315128    1.0000000000
     8   2   2   1   1
       2.4457386745    1.0000000000
     9   3   3   1   1
       2.9286689087    1.0000000000

# RI basis set for F (all-electron) relative DI metric: 2.0e-02
F  RI_aug-TZVP-MOLOPT-ae_N_RI_030_s_p_d_f_g_h_i_4_3_2_1_0_0_0_error_2.0e-02
   10
     1   0   0   1   1
       0.5805940289    1.0000000000
     2   0   0   1   1
       3.1675558546    1.0000000000
     3   0   0   1   1
      25.3348413614    1.0000000000
     4   0   0   1   1
     169.1123811744    1.0000000000
     5   1   1   1   1
       1.1548184263    1.0000000000
     6   1   1   1   1
       2.7581469510    1.0000000000
     7   1   1   1   1
      20.3883108629    1.0000000000
     8   2   2   1   1
       1.2007819906    1.0000000000
     9   2   2   1   1
       4.7129243457    1.0000000000
    10   3   3   1   1
       2.6218953220    1.0000000000

# RI basis set for F (all-electron) relative DI metric: 7.7e-03
F  RI_aug-TZVP-MOLOPT-ae_N_RI_039_s_p_d_f_g_h_i_4_3_2_1_1_0_0_error_7.7e-03
   11
     1   0   0   1   1
       1.1039745150    1.0000000000
     2   0   0   1   1
       1.7043847320    1.0000000000
     3   0   0   1   1
      15.0659123396    1.0000000000
     4   0   0   1   1
     188.4702782241    1.0000000000
     5   1   1   1   1
       1.2484239075    1.0000000000
     6   1   1   1   1
       2.5447504426    1.0000000000
     7   1   1   1   1
      20.2620411338    1.0000000000
     8   2   2   1   1
       1.4211381486    1.0000000000
     9   2   2   1   1
       4.5795761940    1.0000000000
    10   3   3   1   1
       2.1571863826    1.0000000000
    11   4   4   1   1
       2.1941646423    1.0000000000

# RI basis set for F (all-electron) relative DI metric: 3.7e-03
F  RI_aug-TZVP-MOLOPT-ae_N_RI_048_s_p_d_f_g_h_i_6_3_2_2_1_0_0_error_3.7e-03
   14
     1   0   0   1   1
       0.1699094514    1.0000000000
     2   0   0   1   1
       0.9250819525    1.0000000000
     3   0   0   1   1
       3.0470359004    1.0000000000
     4   0   0   1   1
      10.9984245580    1.0000000000
     5   0   0   1   1
      48.7436795027    1.0000000000
     6   0   0   1   1
     213.0718272028    1.0000000000
     7   1   1   1   1
       1.0456873731    1.0000000000
     8   1   1   1   1
       3.3240423482    1.0000000000
     9   1   1   1   1
      27.7645190576    1.0000000000
    10   2   2   1   1
       1.3791686743    1.0000000000
    11   2   2   1   1
       4.5293843080    1.0000000000
    12   3   3   1   1
       1.4705804445    1.0000000000
    13   3   3   1   1
       6.0711901327    1.0000000000
    14   4   4   1   1
       1.9577130841    1.0000000000

# RI basis set for F (all-electron) relative DI metric: 1.5e-03
F  RI_aug-TZVP-MOLOPT-ae_N_RI_062_s_p_d_f_g_h_i_6_4_2_2_1_1_0_error_1.5e-03
   16
     1   0   0   1   1
       0.1516497867    1.0000000000
     2   0   0   1   1
       0.6726916282    1.0000000000
     3   0   0   1   1
       2.7551155299    1.0000000000
```

```
     4   0   0   1   1
     11.6094589634    1.0000000000
     5   0   0   1   1
     49.7967003038    1.0000000000
     6   0   0   1   1
    213.2229211837    1.0000000000
     7   1   1   1   1
      0.9321967540    1.0000000000
     8   1   1   1   1
      2.3033896482    1.0000000000
     9   1   1   1   1
      7.6339448602    1.0000000000
    10   1   1   1   1
     29.2179291885    1.0000000000
    11   2   2   1   1
      1.4032339390    1.0000000000
    12   2   2   1   1
      4.4822635826    1.0000000000
    13   3   3   1   1
      1.5922590442    1.0000000000
    14   3   3   1   1
      5.8686148438    1.0000000000
    15   4   4   1   1
      2.0282465168    1.0000000000
    16   5   5   1   1
      2.3914561801    1.0000000000

# RI basis set for F (all-electron) relative DI metric: 3.1e-04
F  RI_aug-TZVP-MOLOPT-ae_N_RI_070_s_p_d_f_g_h_i_6_5_3_2_1_1_0_error_3.1e-04
   18
     1   0   0   1   1
      0.4341918002    1.0000000000
     2   0   0   1   1
      0.9966189845    1.0000000000
     3   0   0   1   1
      3.3822900838    1.0000000000
     4   0   0   1   1
     14.7048413680    1.0000000000
     5   0   0   1   1
     57.0881677000    1.0000000000
     6   0   0   1   1
    214.4386786799    1.0000000000
     7   1   1   1   1
      0.4990199534    1.0000000000
     8   1   1   1   1
      1.2539682430    1.0000000000
     9   1   1   1   1
      3.9619371622    1.0000000000
    10   1   1   1   1
     13.0010465652    1.0000000000
    11   1   1   1   1
     39.0148213887    1.0000000000
    12   2   2   1   1
      0.8099087384    1.0000000000
    13   2   2   1   1
      1.5908235805    1.0000000000
    14   2   2   1   1
      6.7955081986    1.0000000000
    15   3   3   1   1
      1.6926574641    1.0000000000
    16   3   3   1   1
      5.9420593159    1.0000000000
    17   4   4   1   1
      2.2251822678    1.0000000000
    18   5   5   1   1
      2.2858043998    1.0000000000

# RI basis set for F (all-electron) relative DI metric: 1.4e-04
F  RI_aug-TZVP-MOLOPT-ae_N_RI_085_s_p_d_f_g_h_i_7_5_4_2_2_1_0_error_1.4e-04
   21
     1   0   0   1   1
      0.4344649413    1.0000000000
     2   0   0   1   1
      0.9770593536    1.0000000000
     3   0   0   1   1
      2.5531559995    1.0000000000
     4   0   0   1   1
      7.5266100049    1.0000000000
     5   0   0   1   1
     23.3467627371    1.0000000000
     6   0   0   1   1
     71.1405872436    1.0000000000
     7   0   0   1   1
    214.3494585792    1.0000000000
     8   1   1   1   1
      0.5538129231    1.0000000000
     9   1   1   1   1
      1.1336818302    1.0000000000
    10   1   1   1   1
      3.5900260340    1.0000000000
    11   1   1   1   1
     12.5875654827    1.0000000000
    12   1   1   1   1
     38.6371199804    1.0000000000
    13   2   2   1   1
      0.5418656345    1.0000000000
    14   2   2   1   1
      1.2961294033    1.0000000000
    15   2   2   1   1
      2.7852916243    1.0000000000
    16   2   2   1   1
      8.4185126285    1.0000000000
    17   3   3   1   1
      1.6931406115    1.0000000000
    18   3   3   1   1
      5.9141035128    1.0000000000
    19   4   4   1   1
      1.2392533637    1.0000000000
    20   4   4   1   1
      3.1751830516    1.0000000000
    21   5   5   1   1
```

```
      2.2769874868    1.0000000000

# RI basis set for F (all-electron) relative DI metric: 2.5e-05
F  RI_aug-TZVP-MOLOPT-ae_N_RI_092_s_p_d_f_g_h_i_7_5_4_3_2_1_0_error_2.5e-05
   22
     1   0   0   1   1
      0.4010198869    1.0000000000
     2   0   0   1   1
      1.1232886397    1.0000000000
     3   0   0   1   1
      2.3230418851    1.0000000000
     4   0   0   1   1
      6.1700821511    1.0000000000
     5   0   0   1   1
     20.6749546189    1.0000000000
     6   0   0   1   1
     67.9058735280    1.0000000000
     7   0   0   1   1
    211.0314644356    1.0000000000
     8   1   1   1   1
      0.4473513341    1.0000000000
     9   1   1   1   1
      1.3719406999    1.0000000000
    10   1   1   1   1
      3.9235700582    1.0000000000
    11   1   1   1   1
     11.9783104583    1.0000000000
    12   1   1   1   1
     37.0993227808    1.0000000000
    13   2   2   1   1
      0.6380579475    1.0000000000
    14   2   2   1   1
      1.3752713525    1.0000000000
    15   2   2   1   1
      3.0305908393    1.0000000000
    16   2   2   1   1
      8.8951684001    1.0000000000
    17   3   3   1   1
      0.6704623423    1.0000000000
    18   3   3   1   1
      1.9384185814    1.0000000000
    19   3   3   1   1
      7.2881693204    1.0000000000
    20   4   4   1   1
      0.7346886504    1.0000000000
    21   4   4   1   1
      2.3343617433    1.0000000000
    22   5   5   1   1
      2.5990780387    1.0000000000

# RI basis set for F (all-electron) relative DI metric: 1.0e-05
F  RI_aug-TZVP-MOLOPT-ae_N_RI_109_s_p_d_f_g_h_i_7_6_5_3_3_1_0_error_1.0e-05
   25
     1   0   0   1   1
      0.3993525300    1.0000000000
     2   0   0   1   1
      1.0750833316    1.0000000000
     3   0   0   1   1
      2.0646674590    1.0000000000
     4   0   0   1   1
      6.0422927442    1.0000000000
     5   0   0   1   1
     20.2512555222    1.0000000000
     6   0   0   1   1
     67.3386263306    1.0000000000
     7   0   0   1   1
    210.4491507864    1.0000000000
     8   1   1   1   1
      0.4189286013    1.0000000000
     9   1   1   1   1
      1.4928588773    1.0000000000
    10   1   1   1   1
      2.4584046203    1.0000000000
    11   1   1   1   1
      6.0373979321    1.0000000000
    12   1   1   1   1
     15.2424059187    1.0000000000
    13   1   1   1   1
     37.9629445325    1.0000000000
    14   2   2   1   1
      0.4324477958    1.0000000000
    15   2   2   1   1
      0.8518020478    1.0000000000
    16   2   2   1   1
      1.9570861292    1.0000000000
    17   2   2   1   1
      4.8360420376    1.0000000000
    18   2   2   1   1
     11.2723144799    1.0000000000
    19   3   3   1   1
      0.6447238850    1.0000000000
    20   3   3   1   1
      2.0307889627    1.0000000000
    21   3   3   1   1
      7.8231635966    1.0000000000
    22   4   4   1   1
      0.9094668469    1.0000000000
    23   4   4   1   1
      1.8491013286    1.0000000000
    24   4   4   1   1
      3.6688259104    1.0000000000
    25   5   5   1   1
      2.6911348806    1.0000000000

# RI basis set for Ne (all-electron) relative DI metric: 4.6e-02
Ne  RI_aug-SZV-MOLOPT-ae-mini_N_RI_005_s_p_d_f_g_h_i_2_1_0_0_0_0_0_error_4.6e-02
   3
     1   0   0   1   1
      0.4396430870    1.0000000000
     2   0   0   1   1
      2.6397826698    1.0000000000
```

```
     3   1   1   1   1
      7.7663691038    1.0000000000

# RI basis set for Ne (all-electron) relative DI metric: 1.2e-02
Ne  RI_aug-SZV-MOLOPT-ae-mini_N_RI_010_s_p_d_f_g_h_i_2_1_1_0_0_0_0_error_1.2e-02
   4
     1   0   0   1   1
      0.6470235460    1.0000000000
     2   0   0   1   1
      4.9119856195    1.0000000000
     3   1   1   1   1
      7.8247632026    1.0000000000
     4   2   2   1   1
     10.6951494256    1.0000000000

# RI basis set for Ne (all-electron) relative DI metric: 5.5e-03
Ne  RI_aug-SZV-MOLOPT-ae-mini_N_RI_014_s_p_d_f_g_h_i_3_2_1_0_0_0_0_error_5.5e-03
   6
     1   0   0   1   1
      0.7202455931    1.0000000000
     2   0   0   1   1
      1.6316110087    1.0000000000
     3   0   0   1   1
      5.6562968091    1.0000000000
     4   1   1   1   1
      1.4836758343    1.0000000000
     5   1   1   1   1
      3.1235290951    1.0000000000
     6   2   2   1   1
     10.6900477387    1.0000000000

# RI basis set for Ne (all-electron) relative DI metric: 2.9e-04
Ne  RI_aug-SZV-MOLOPT-ae-mini_N_RI_019_s_p_d_f_g_h_i_3_2_2_0_0_0_0_error_2.9e-04
   7
     1   0   0   1   1
      1.1074553342    1.0000000000
     2   0   0   1   1
      3.2945011426    1.0000000000
     3   0   0   1   1
      7.2844895175    1.0000000000
     4   1   1   1   1
      0.8946013757    1.0000000000
     5   1   1   1   1
      4.4490962921    1.0000000000
     6   2   2   1   1
      1.4396420776    1.0000000000
     7   2   2   1   1
      6.4182032895    1.0000000000

# RI basis set for Ne (all-electron) relative DI metric: 8.5e-05
Ne  RI_aug-SZV-MOLOPT-ae-mini_N_RI_022_s_p_d_f_g_h_i_6_2_2_0_0_0_0_error_8.5e-05
   10
     1   0   0   1   1
      0.4922546259    1.0000000000
     2   0   0   1   1
      0.9617512476    1.0000000000
     3   0   0   1   1
      1.8842364269    1.0000000000
     4   0   0   1   1
      3.6627483787    1.0000000000
     5   0   0   1   1
      7.0732518963    1.0000000000
     6   0   0   1   1
     13.6954045742    1.0000000000
     7   1   1   1   1
      1.5239328532    1.0000000000
     8   1   1   1   1
      3.1079871667    1.0000000000
     9   2   2   1   1
      1.3856095335    1.0000000000
    10   2   2   1   1
      6.9552605104    1.0000000000

# RI basis set for Ne (all-electron) relative DI metric: 5.6e-06
Ne  RI_aug-SZV-MOLOPT-ae-mini_N_RI_025_s_p_d_f_g_h_i_6_3_2_0_0_0_0_error_5.6e-06
   11
     1   0   0   1   1
      0.4615951456    1.0000000000
     2   0   0   1   1
      0.8519527537    1.0000000000
     3   0   0   1   1
      1.8240149790    1.0000000000
     4   0   0   1   1
      3.8621946903    1.0000000000
     5   0   0   1   1
      6.9804997151    1.0000000000
     6   0   0   1   1
     12.9753826580    1.0000000000
     7   1   1   1   1
      0.8739951430    1.0000000000
     8   1   1   1   1
      1.9452087650    1.0000000000
     9   1   1   1   1
      4.3293574365    1.0000000000
    10   2   2   1   1
      1.3929514592    1.0000000000
    11   2   2   1   1
      6.9716018890    1.0000000000

# RI basis set for Ne (all-electron) relative DI metric: 1.3e-06
Ne  RI_aug-SZV-MOLOPT-ae-mini_N_RI_030_s_p_d_f_g_h_i_6_3_3_0_0_0_0_error_1.3e-06
   12
     1   0   0   1   1
      0.4741939914    1.0000000000
     2   0   0   1   1
      0.8824751469    1.0000000000
     3   0   0   1   1
      1.8394572601    1.0000000000
     4   0   0   1   1
      3.8108846344    1.0000000000
     5   0   0   1   1
```

```
      7.0210828182    1.0000000000
     6   0   0   1   1
     13.1978915379    1.0000000000
     7   1   1   1   1
      0.9266870276    1.0000000000
     8   1   1   1   1
      1.4423233229    1.0000000000
     9   1   1   1   1
      4.2651945220    1.0000000000
    10   2   2   1   1
      1.5145489782    1.0000000000
    11   2   2   1   1
      3.2176975373    1.0000000000
    12   2   2   1   1
      8.4938858380    1.0000000000

# RI basis set for Ne (all-electron) relative DI metric: 6.5e-07
Ne  RI_aug-SZV-MOLOPT-ae-mini_N_RI_037_s_p_d_f_g_h_i_7_5_3_0_0_0_0_error_6.5e-07
   15
     1   0   0   1   1
      0.4997512165    1.0000000000
     2   0   0   1   1
      0.8685208982    1.0000000000
     3   0   0   1   1
      1.5080317711    1.0000000000
     4   0   0   1   1
      2.6154691323    1.0000000000
     5   0   0   1   1
      4.5462249578    1.0000000000
     6   0   0   1   1
      7.8868272336    1.0000000000
     7   0   0   1   1
     13.6690873913    1.0000000000
     8   1   1   1   1
      0.5024152010    1.0000000000
     9   1   1   1   1
      1.1443132358    1.0000000000
    10   1   1   1   1
      2.6123894548    1.0000000000
    11   1   1   1   1
      6.0004446902    1.0000000000
    12   1   1   1   1
     13.7385350501    1.0000000000
    13   2   2   1   1
      1.6530669972    1.0000000000
    14   2   2   1   1
      3.0330711708    1.0000000000
    15   2   2   1   1
      8.1910779146    1.0000000000

# RI basis set for Ne (all-electron) relative DI metric: 1.4e-08
Ne  RI_aug-SZV-MOLOPT-ae-mini_N_RI_042_s_p_d_f_g_h_i_7_5_4_0_0_0_0_error_1.4e-08
   16
     1   0   0   1   1
      0.5000000000    1.0000000000
     2   0   0   1   1
      0.8686132474    1.0000000000
     3   0   0   1   1
      1.5089779472    1.0000000000
     4   0   0   1   1
      2.6214364701    1.0000000000
     5   0   0   1   1
      4.5540288904    1.0000000000
     6   0   0   1   1
      7.9113796468    1.0000000000
     7   0   0   1   1
     13.7438583334    1.0000000000
     8   1   1   1   1
      0.5000000005    1.0000000000
     9   1   1   1   1
      1.1448660336    1.0000000000
    10   1   1   1   1
      2.6214364683    1.0000000000
    11   1   1   1   1
      6.0023871476    1.0000000000
    12   1   1   1   1
     13.7438583323    1.0000000000
    13   2   2   1   1
      0.5000000000    1.0000000000
    14   2   2   1   1
      1.5089779476    1.0000000000
    15   2   2   1   1
      4.5540288920    1.0000000000
    16   2   2   1   1
     13.7438583291    1.0000000000

# RI basis set for Ne (all-electron) relative DI metric: 1.7e-09
Ne  RI_aug-SZV-MOLOPT-ae-mini_N_RI_045_s_p_d_f_g_h_i_7_6_4_0_0_0_0_error_1.7e-09
   17
     1   0   0   1   1
      0.5000000000    1.0000000000
     2   0   0   1   1
      0.8686132474    1.0000000000
     3   0   0   1   1
      1.5089779472    1.0000000000
     4   0   0   1   1
      2.6214364701    1.0000000000
     5   0   0   1   1
      4.5540288904    1.0000000000
     6   0   0   1   1
      7.9113796468    1.0000000000
     7   0   0   1   1
     13.7438583334    1.0000000000
     8   1   1   1   1
      0.5000000000    1.0000000000
     9   1   1   1   1
      0.9700580801    1.0000000000
    10   1   1   1   1
      1.8820253574    1.0000000000
    11   1   1   1   1
      3.6513478097    1.0000000000
```

```
    12   1   1   1   1
      7.0840388920    1.0000000000
    13   1   1   1   1
     13.7438583333    1.0000000000
    14   2   2   1   1
      0.5000000000    1.0000000000
    15   2   2   1   1
      1.5089779476    1.0000000000
    16   2   2   1   1
      4.5540288920    1.0000000000
    17   2   2   1   1
     13.7438583291    1.0000000000

# RI basis set for Ne (all-electron) relative DI metric: 4.6e-02
Ne  RI_aug-SZV-MOLOPT-ae_N_RI_013_s_p_d_f_g_h_i_4_3_0_0_0_0_0_error_4.6e-02
   7
     1   0   0   1   1
      0.4540627374    1.0000000000
     2   0   0   1   1
      3.4508094502    1.0000000000
     3   0   0   1   1
     12.0238601291    1.0000000000
     4   0   0   1   1
    248.1712426934    1.0000000000
     5   1   1   1   1
      0.5030588809    1.0000000000
     6   1   1   1   1
      1.5898694713    1.0000000000
     7   1   1   1   1
      4.7178484985    1.0000000000

# RI basis set for Ne (all-electron) relative DI metric: 5.4e-03
Ne  RI_aug-SZV-MOLOPT-ae_N_RI_023_s_p_d_f_g_h_i_4_3_2_0_0_0_0_error_5.4e-03
   9
     1   0   0   1   1
      1.1145936046    1.0000000000
     2   0   0   1   1
      4.0188134116    1.0000000000
     3   0   0   1   1
     32.5557548284    1.0000000000
     4   0   0   1   1
    248.8228593829    1.0000000000
     5   1   1   1   1
      1.5706780221    1.0000000000
     6   1   1   1   1
      3.6813352420    1.0000000000
     7   1   1   1   1
     44.5237683165    1.0000000000
     8   2   2   1   1
      1.3675624022    1.0000000000
     9   2   2   1   1
      7.9367141797    1.0000000000

# RI basis set for Ne (all-electron) relative DI metric: 4.7e-04
Ne  RI_aug-SZV-MOLOPT-ae_N_RI_032_s_p_d_f_g_h_i_6_3_2_1_0_0_0_error_4.7e-04
   12
     1   0   0   1   1
      0.7666056024    1.0000000000
     2   0   0   1   1
      1.2854170316    1.0000000000
     3   0   0   1   1
      3.6886329222    1.0000000000
     4   0   0   1   1
     17.0760387666    1.0000000000
     5   0   0   1   1
     70.9275776754    1.0000000000
     6   0   0   1   1
    269.4630211098    1.0000000000
     7   1   1   1   1
      0.9026747802    1.0000000000
     8   1   1   1   1
      4.9007131058    1.0000000000
     9   1   1   1   1
     29.3209579190    1.0000000000
    10   2   2   1   1
      1.4036427268    1.0000000000
    11   2   2   1   1
      8.6385981645    1.0000000000
    12   3   3   1   1
      1.3996533273    1.0000000000

# RI basis set for Ne (all-electron) relative DI metric: 2.2e-04
Ne  RI_aug-SZV-MOLOPT-ae_N_RI_035_s_p_d_f_g_h_i_6_4_2_1_0_0_0_error_2.2e-04
   13
     1   0   0   1   1
      0.6442025605    1.0000000000
     2   0   0   1   1
      1.2354169696    1.0000000000
     3   0   0   1   1
      3.9651382454    1.0000000000
     4   0   0   1   1
     18.0643184694    1.0000000000
     5   0   0   1   1
     71.5281229650    1.0000000000
     6   0   0   1   1
    269.9284827237    1.0000000000
     7   1   1   1   1
      1.0026561086    1.0000000000
     8   1   1   1   1
      3.5962096683    1.0000000000
     9   1   1   1   1
     12.2121702891    1.0000000000
    10   1   1   1   1
     39.8518908918    1.0000000000
    11   2   2   1   1
      1.4218008058    1.0000000000
    12   2   2   1   1
      8.7349231287    1.0000000000
    13   3   3   1   1
      1.4750730154    1.0000000000
```

```
# RI basis set for Ne (all-electron) relative DI metric: 6.4e-05
Ne  RI_aug-SZV-MOLOPT-ae_N_RI_039_s_p_d_f_g_h_i_7_5_2_1_0_0_0_error_6.4e-05
   15
    1   0   0   1   1
      0.3517191181    1.0000000000
    2   0   0   1   1
      1.0032685659    1.0000000000
    3   0   0   1   1
      3.0005862499    1.0000000000
    4   0   0   1   1
      9.9828612436    1.0000000000
    5   0   0   1   1
     30.1588493449    1.0000000000
    6   0   0   1   1
     90.2858368531    1.0000000000
    7   0   0   1   1
    270.9531472332    1.0000000000
    8   1   1   1   1
      0.9292162129    1.0000000000
    9   1   1   1   1
      1.5753619232    1.0000000000
   10   1   1   1   1
      4.5694253686    1.0000000000
   11   1   1   1   1
     16.3851427408    1.0000000000
   12   1   1   1   1
     49.1629139374    1.0000000000
   13   2   2   1   1
      1.4544881490    1.0000000000
   14   2   2   1   1
      8.8893425686    1.0000000000
   15   3   3   1   1
      1.3228713604    1.0000000000

# RI basis set for Ne (all-electron) relative DI metric: 2.4e-05
Ne  RI_aug-SZV-MOLOPT-ae_N_RI_044_s_p_d_f_g_h_i_7_5_3_1_0_0_0_error_2.4e-05
   16
    1   0   0   1   1
      0.4728115665    1.0000000000
    2   0   0   1   1
      0.9909011671    1.0000000000
    3   0   0   1   1
      2.8374191907    1.0000000000
    4   0   0   1   1
      9.8663550345    1.0000000000
    5   0   0   1   1
     30.1404072672    1.0000000000
    6   0   0   1   1
     90.2394481398    1.0000000000
    7   0   0   1   1
    270.8865117863    1.0000000000
    8   1   1   1   1
      0.7464916145    1.0000000000
    9   1   1   1   1
      1.4393031805    1.0000000000
   10   1   1   1   1
      4.2520972844    1.0000000000
   11   1   1   1   1
     16.0555654645    1.0000000000
   12   1   1   1   1
     48.9264656153    1.0000000000
   13   2   2   1   1
      1.0494944895    1.0000000000
   14   2   2   1   1
      2.3951395854    1.0000000000
   15   2   2   1   1
      6.3360734649    1.0000000000
   16   3   3   1   1
      1.3704206901    1.0000000000

# RI basis set for Ne (all-electron) relative DI metric: 1.2e-05
Ne  RI_aug-SZV-MOLOPT-ae_N_RI_052_s_p_d_f_g_h_i_7_6_4_1_0_0_0_error_1.2e-05
   18
    1   0   0   1   1
      0.4369762147    1.0000000000
    2   0   0   1   1
      1.0948867866    1.0000000000
    3   0   0   1   1
      3.1767659013    1.0000000000
    4   0   0   1   1
      9.9653088337    1.0000000000
    5   0   0   1   1
     30.0894190021    1.0000000000
    6   0   0   1   1
     90.2374412034    1.0000000000
    7   0   0   1   1
    270.9018327202    1.0000000000
    8   1   1   1   1
      0.2812122414    1.0000000000
    9   1   1   1   1
      0.9251692692    1.0000000000
   10   1   1   1   1
      2.0262847979    1.0000000000
   11   1   1   1   1
      4.5858712127    1.0000000000
   12   1   1   1   1
     16.9499778041    1.0000000000
   13   1   1   1   1
     49.5404497714    1.0000000000
   14   2   2   1   1
      0.1306624678    1.0000000000
   15   2   2   1   1
      0.4930571299    1.0000000000
   16   2   2   1   1
      1.8605587384    1.0000000000
   17   2   2   1   1
      7.0208546765    1.0000000000
   18   3   3   1   1
      1.3842971113    1.0000000000

# RI basis set for Ne (all-electron) relative DI metric: 3.5e-06
```

```
Ne  RI_aug-SZV-MOLOPT-ae_N_RI_059_s_p_d_f_g_h_i_7_6_4_2_0_0_0_error_3.5e-06
   19
     1   0   0   1   1
      0.6755892288    1.0000000000
     2   0   0   1   1
      1.1846505119    1.0000000000
     3   0   0   1   1
      2.8655028492    1.0000000000
     4   0   0   1   1
      9.6664574627    1.0000000000
     5   0   0   1   1
     29.9290700239    1.0000000000
     6   0   0   1   1
     90.0069345091    1.0000000000
     7   0   0   1   1
    270.6513437481    1.0000000000
     8   1   1   1   1
      1.0080454395    1.0000000000
     9   1   1   1   1
      1.2585667998    1.0000000000
    10   1   1   1   1
      3.1923992245    1.0000000000
    11   1   1   1   1
      8.8040205177    1.0000000000
    12   1   1   1   1
     20.7571384030    1.0000000000
    13   1   1   1   1
     49.1716634163    1.0000000000
    14   2   2   1   1
      0.7055125536    1.0000000000
    15   2   2   1   1
      1.5944903875    1.0000000000
    16   2   2   1   1
      4.1924538998    1.0000000000
    17   2   2   1   1
     10.3401603487    1.0000000000
    18   3   3   1   1
      1.1874047171    1.0000000000
    19   3   3   1   1
      7.1577113915    1.0000000000

# RI basis set for Ne (all-electron) relative DI metric: 9.4e-07
Ne  RI_aug-SZV-MOLOPT-ae_N_RI_064_s_p_d_f_g_h_i_7_6_5_2_0_0_0_error_9.4e-07
   20
     1   0   0   1   1
      0.6609948708    1.0000000000
     2   0   0   1   1
      1.1586669103    1.0000000000
     3   0   0   1   1
      3.0690972062    1.0000000000
     4   0   0   1   1
      9.6957425652    1.0000000000
     5   0   0   1   1
     29.8174288884    1.0000000000
     6   0   0   1   1
     89.9741894853    1.0000000000
     7   0   0   1   1
    270.6095368855    1.0000000000
     8   1   1   1   1
      0.7129930635    1.0000000000
     9   1   1   1   1
      1.2195701554    1.0000000000
    10   1   1   1   1
      3.1876365832    1.0000000000
    11   1   1   1   1
      8.6716048834    1.0000000000
    12   1   1   1   1
     20.5623456675    1.0000000000
    13   1   1   1   1
     49.1034189653    1.0000000000
    14   2   2   1   1
      0.4590190245    1.0000000000
    15   2   2   1   1
      1.0526751450    1.0000000000
    16   2   2   1   1
      2.1517352975    1.0000000000
    17   2   2   1   1
      4.7751549264    1.0000000000
    18   2   2   1   1
     12.2227525783    1.0000000000
    19   3   3   1   1
      1.1305666988    1.0000000000
    20   3   3   1   1
      4.8368320762    1.0000000000

# RI basis set for Ne (all-electron) relative DI metric: 3.7e-07
Ne  RI_aug-SZV-MOLOPT-ae_N_RI_078_s_p_d_f_g_h_i_7_6_5_4_0_0_0_error_3.7e-07
   22
     1   0   0   1   1
      0.5026298359    1.0000000000
     2   0   0   1   1
      1.1688355406    1.0000000000
     3   0   0   1   1
      3.3869599965    1.0000000000
     4   0   0   1   1
      9.9405994859    1.0000000000
     5   0   0   1   1
     29.9773413125    1.0000000000
     6   0   0   1   1
     90.1673653221    1.0000000000
     7   0   0   1   1
    270.8229540199    1.0000000000
     8   1   1   1   1
      0.6363648950    1.0000000000
     9   1   1   1   1
      1.5523515755    1.0000000000
    10   1   1   1   1
      3.6944925545    1.0000000000
    11   1   1   1   1
      8.8486509354    1.0000000000
    12   1   1   1   1
```

```
     20.9052834365    1.0000000000
    13   1   1   1   1
     49.4337699409    1.0000000000
    14   2   2   1   1
      0.4517495690    1.0000000000
    15   2   2   1   1
      1.0150381481    1.0000000000
    16   2   2   1   1
      2.2576725711    1.0000000000
    17   2   2   1   1
      5.0682245437    1.0000000000
    18   2   2   1   1
     11.5877595443    1.0000000000
    19   3   3   1   1
      0.9571282230    1.0000000000
    20   3   3   1   1
      2.0372817062    1.0000000000
    21   3   3   1   1
      4.0318374206    1.0000000000
    22   3   3   1   1
      8.0136192364    1.0000000000

# RI basis set for Ne (all-electron) relative DI metric: 2.5e-02
Ne  RI_aug-DZVP-MOLOPT-ae_N_RI_020_s_p_d_f_g_h_i_4_2_2_0_0_0_0_error_2.5e-02
   8
     1   0   0   1   1
      0.7946571730    1.0000000000
     2   0   0   1   1
      3.7813309102    1.0000000000
     3   0   0   1   1
     22.6099094942    1.0000000000
     4   0   0   1   1
    120.5404341233    1.0000000000
     5   1   1   1   1
      0.8197061664    1.0000000000
     6   1   1   1   1
      7.5286844839    1.0000000000
     7   2   2   1   1
      1.2621129891    1.0000000000
     8   2   2   1   1
      8.5613192368    1.0000000000

# RI basis set for Ne (all-electron) relative DI metric: 2.1e-03
Ne  RI_aug-DZVP-MOLOPT-ae_N_RI_029_s_p_d_f_g_h_i_6_2_2_1_0_0_0_error_2.1e-03
   11
     1   0   0   1   1
      0.1818778048    1.0000000000
     2   0   0   1   1
      0.7626334785    1.0000000000
     3   0   0   1   1
      3.1977341727    1.0000000000
     4   0   0   1   1
     13.4078188337    1.0000000000
     5   0   0   1   1
     56.2193774590    1.0000000000
     6   0   0   1   1
    235.7293034077    1.0000000000
     7   1   1   1   1
      1.2831535621    1.0000000000
     8   1   1   1   1
      4.9983907726    1.0000000000
     9   2   2   1   1
      1.7956420224    1.0000000000
    10   2   2   1   1
      7.0601713055    1.0000000000
    11   3   3   1   1
      1.9172489113    1.0000000000

# RI basis set for Ne (all-electron) relative DI metric: 9.2e-04
Ne  RI_aug-DZVP-MOLOPT-ae_N_RI_035_s_p_d_f_g_h_i_6_4_2_1_0_0_0_error_9.2e-04
   13
     1   0   0   1   1
      0.1843398200    1.0000000000
     2   0   0   1   1
      0.7897250611    1.0000000000
     3   0   0   1   1
      3.3831444608    1.0000000000
     4   0   0   1   1
     14.4930602914    1.0000000000
     5   0   0   1   1
     62.0885704379    1.0000000000
     6   0   0   1   1
    265.9876329545    1.0000000000
     7   1   1   1   1
      0.8503666325    1.0000000000
     8   1   1   1   1
      1.9256375040    1.0000000000
     9   1   1   1   1
      8.2382918692    1.0000000000
    10   1   1   1   1
     45.4813975727    1.0000000000
    11   2   2   1   1
      1.7866349016    1.0000000000
    12   2   2   1   1
      7.3226593049    1.0000000000
    13   3   3   1   1
      1.9468669080    1.0000000000

# RI basis set for Ne (all-electron) relative DI metric: 2.8e-04
Ne  RI_aug-DZVP-MOLOPT-ae_N_RI_040_s_p_d_f_g_h_i_6_4_3_1_0_0_0_error_2.8e-04
   14
     1   0   0   1   1
      0.5520052279    1.0000000000
     2   0   0   1   1
      1.4954573529    1.0000000000
     3   0   0   1   1
      5.4064121132    1.0000000000
     4   0   0   1   1
     19.1783079475    1.0000000000
     5   0   0   1   1
     72.1811210814    1.0000000000
```

```
      6   0   0   1   1
    270.7185862421    1.0000000000
      7   1   1   1   1
      0.9424987478    1.0000000000
      8   1   1   1   1
      2.3334430124    1.0000000000
      9   1   1   1   1
      7.9526482931    1.0000000000
     10   1   1   1   1
     26.9217125612    1.0000000000
     11   2   2   1   1
      0.8418415987    1.0000000000
     12   2   2   1   1
      2.0636381596    1.0000000000
     13   2   2   1   1
     10.7481957881    1.0000000000
     14   3   3   1   1
      1.6711478410    1.0000000000

# RI basis set for Ne (all-electron) relative DI metric: 7.3e-05
Ne  RI_aug-DZVP-MOLOPT-ae_N_RI_043_s_p_d_f_g_h_i_6_5_3_1_0_0_0_error_7.3e-05
   15
      1   0   0   1   1
      0.5030684302    1.0000000000
      2   0   0   1   1
      1.2335651213    1.0000000000
      3   0   0   1   1
      4.2702949056    1.0000000000
      4   0   0   1   1
     18.4050512939    1.0000000000
      5   0   0   1   1
     71.7530571959    1.0000000000
      6   0   0   1   1
    270.2053159208    1.0000000000
      7   1   1   1   1
      0.6382315084    1.0000000000
      8   1   1   1   1
      1.2298412518    1.0000000000
      9   1   1   1   1
      4.3860294350    1.0000000000
     10   1   1   1   1
     13.4891310461    1.0000000000
     11   1   1   1   1
     46.7950398653    1.0000000000
     12   2   2   1   1
      0.7213821990    1.0000000000
     13   2   2   1   1
      1.9304649556    1.0000000000
     14   2   2   1   1
     10.4160573710    1.0000000000
     15   3   3   1   1
      1.9214450206    1.0000000000

# RI basis set for Ne (all-electron) relative DI metric: 2.3e-05
Ne  RI_aug-DZVP-MOLOPT-ae_N_RI_054_s_p_d_f_g_h_i_7_6_3_2_0_0_0_error_2.3e-05
   18
      1   0   0   1   1
      0.4695435949    1.0000000000
      2   0   0   1   1
      1.1404303096    1.0000000000
      3   0   0   1   1
      3.2966769928    1.0000000000
      4   0   0   1   1
      9.8962311494    1.0000000000
      5   0   0   1   1
     29.9839831081    1.0000000000
      6   0   0   1   1
     90.1608271127    1.0000000000
      7   0   0   1   1
    270.8062408705    1.0000000000
      8   1   1   1   1
      0.4887997525    1.0000000000
      9   1   1   1   1
      1.2538346110    1.0000000000
     10   1   1   1   1
      3.4293442223    1.0000000000
     11   1   1   1   1
      8.3316723744    1.0000000000
     12   1   1   1   1
     20.4585827317    1.0000000000
     13   1   1   1   1
     49.0357896851    1.0000000000
     14   2   2   1   1
      0.6201554542    1.0000000000
     15   2   2   1   1
      2.0430375461    1.0000000000
     16   2   2   1   1
     11.0808712388    1.0000000000
     17   3   3   1   1
      0.8262580108    1.0000000000
     18   3   3   1   1
      2.7739076724    1.0000000000

# RI basis set for Ne (all-electron) relative DI metric: 1.1e-05
Ne  RI_aug-DZVP-MOLOPT-ae_N_RI_063_s_p_d_f_g_h_i_7_6_3_2_1_0_0_error_1.1e-05
   19
      1   0   0   1   1
      0.4640993045    1.0000000000
      2   0   0   1   1
      1.1423423224    1.0000000000
      3   0   0   1   1
      3.2820685577    1.0000000000
      4   0   0   1   1
      9.9345847941    1.0000000000
      5   0   0   1   1
     30.0105626896    1.0000000000
      6   0   0   1   1
     90.1709949774    1.0000000000
      7   0   0   1   1
    270.8270591458    1.0000000000
      8   1   1   1   1
```

```
      0.5283259145    1.0000000000
     9   1   1   1   1
      1.2557236228    1.0000000000
    10   1   1   1   1
      3.4232747943    1.0000000000
    11   1   1   1   1
      8.3539712034    1.0000000000
    12   1   1   1   1
     20.4689959272    1.0000000000
    13   1   1   1   1
     49.0424456261    1.0000000000
    14   2   2   1   1
      0.6087514080    1.0000000000
    15   2   2   1   1
      2.0342104293    1.0000000000
    16   2   2   1   1
     10.7624080111    1.0000000000
    17   3   3   1   1
      0.9399108136    1.0000000000
    18   3   3   1   1
      2.9208573775    1.0000000000
    19   4   4   1   1
      2.6885512306    1.0000000000

# RI basis set for Ne (all-electron) relative DI metric: 1.9e-06
Ne  RI_aug-DZVP-MOLOPT-ae_N_RI_075_s_p_d_f_g_h_i_7_6_4_3_1_0_0_error_1.9e-06
   21
     1   0   0   1   1
      0.4202772000    1.0000000000
     2   0   0   1   1
      1.1896827660    1.0000000000
     3   0   0   1   1
      3.2708109825    1.0000000000
     4   0   0   1   1
      9.2818145563    1.0000000000
     5   0   0   1   1
     29.5144374926    1.0000000000
     6   0   0   1   1
     89.7658224694    1.0000000000
     7   0   0   1   1
    270.2921635114    1.0000000000
     8   1   1   1   1
      0.3681264522    1.0000000000
     9   1   1   1   1
      1.2988783152    1.0000000000
    10   1   1   1   1
      3.2671159309    1.0000000000
    11   1   1   1   1
      7.9992310551    1.0000000000
    12   1   1   1   1
     20.3965536002    1.0000000000
    13   1   1   1   1
     49.1101992213    1.0000000000
    14   2   2   1   1
      0.5536955735    1.0000000000
    15   2   2   1   1
      1.6440303283    1.0000000000
    16   2   2   1   1
      3.9215473771    1.0000000000
    17   2   2   1   1
     12.5470935556    1.0000000000
    18   3   3   1   1
      0.4907125176    1.0000000000
    19   3   3   1   1
      1.8429770236    1.0000000000
    20   3   3   1   1
      5.9896552346    1.0000000000
    21   4   4   1   1
      0.9492710411    1.0000000000

# RI basis set for Ne (all-electron) relative DI metric: 9.0e-07
Ne  RI_aug-DZVP-MOLOPT-ae_N_RI_087_s_p_d_f_g_h_i_7_6_5_4_1_0_0_error_9.0e-07
   23
     1   0   0   1   1
      0.4442980251    1.0000000000
     2   0   0   1   1
      1.2366018221    1.0000000000
     3   0   0   1   1
      3.3100825195    1.0000000000
     4   0   0   1   1
      8.2129947535    1.0000000000
     5   0   0   1   1
     28.4100123214    1.0000000000
     6   0   0   1   1
     88.9347957964    1.0000000000
     7   0   0   1   1
    269.2984672186    1.0000000000
     8   1   1   1   1
      0.3480172209    1.0000000000
     9   1   1   1   1
      0.9481582464    1.0000000000
    10   1   1   1   1
      1.7381779139    1.0000000000
    11   1   1   1   1
      4.3875649500    1.0000000000
    12   1   1   1   1
     11.2111330391    1.0000000000
    13   1   1   1   1
     45.7300632666    1.0000000000
    14   2   2   1   1
      0.5362266126    1.0000000000
    15   2   2   1   1
      1.0008106068    1.0000000000
    16   2   2   1   1
      2.2779034543    1.0000000000
    17   2   2   1   1
      4.8121575794    1.0000000000
    18   2   2   1   1
     14.0453005934    1.0000000000
    19   3   3   1   1
      0.4719734430    1.0000000000
```

```
    20   3   3   1   1
      1.3736723829    1.0000000000
    21   3   3   1   1
      3.3016974290    1.0000000000
    22   3   3   1   1
      7.7985832589    1.0000000000
    23   4   4   1   1
      1.1401915323    1.0000000000

# RI basis set for Ne (all-electron) relative DI metric: 3.3e-07
Ne  RI_aug-DZVP-MOLOPT-ae_N_RI_115_s_p_d_f_g_h_i_7_6_6_6_2_0_0_error_3.3e-07
   27
     1   0   0   1   1
      0.3933140805    1.0000000000
     2   0   0   1   1
      1.2570823851    1.0000000000
     3   0   0   1   1
      2.8007638992    1.0000000000
     4   0   0   1   1
      7.6748782256    1.0000000000
     5   0   0   1   1
     27.7561466144    1.0000000000
     6   0   0   1   1
     88.3396082750    1.0000000000
     7   0   0   1   1
    268.5817092000    1.0000000000
     8   1   1   1   1
      0.3701625588    1.0000000000
     9   1   1   1   1
      1.2187377276    1.0000000000
    10   1   1   1   1
      1.8602058581    1.0000000000
    11   1   1   1   1
      4.7128998466    1.0000000000
    12   1   1   1   1
     15.8946091097    1.0000000000
    13   1   1   1   1
     48.8654570141    1.0000000000
    14   2   2   1   1
      0.4266648268    1.0000000000
    15   2   2   1   1
      0.8566308763    1.0000000000
    16   2   2   1   1
      1.7832048114    1.0000000000
    17   2   2   1   1
      3.4655614332    1.0000000000
    18   2   2   1   1
      6.6094290402    1.0000000000
    19   2   2   1   1
     16.7296963984    1.0000000000
    20   3   3   1   1
      0.4162255575    1.0000000000
    21   3   3   1   1
      1.2394359884    1.0000000000
    22   3   3   1   1
      2.0946195182    1.0000000000
    23   3   3   1   1
      5.1173724886    1.0000000000
    24   3   3   1   1
     10.7909257787    1.0000000000
    25   3   3   1   1
     23.0934338936    1.0000000000
    26   4   4   1   1
      0.7767254350    1.0000000000
    27   4   4   1   1
      3.4191739506    1.0000000000

# RI basis set for Ne (all-electron) relative DI metric: 2.0e-02
Ne  RI_aug-TZVP-MOLOPT-ae_N_RI_029_s_p_d_f_g_h_i_6_2_2_1_0_0_0_error_2.0e-02
   11
     1   0   0   1   1
      0.7437104806    1.0000000000
     2   0   0   1   1
      1.6377060400    1.0000000000
     3   0   0   1   1
      5.4216491061    1.0000000000
     4   0   0   1   1
     17.8648897168    1.0000000000
     5   0   0   1   1
     70.7470901890    1.0000000000
     6   0   0   1   1
    269.2650634749    1.0000000000
     7   1   1   1   1
      1.6899358353    1.0000000000
     8   1   1   1   1
      3.4679880125    1.0000000000
     9   2   2   1   1
      1.9856723653    1.0000000000
    10   2   2   1   1
      8.3152129005    1.0000000000
    11   3   3   1   1
      4.2793240118    1.0000000000

# RI basis set for Ne (all-electron) relative DI metric: 1.0e-02
Ne  RI_aug-TZVP-MOLOPT-ae_N_RI_039_s_p_d_f_g_h_i_6_3_2_2_0_0_0_error_1.0e-02
   13
     1   0   0   1   1
      0.2047592637    1.0000000000
     2   0   0   1   1
      0.9189252249    1.0000000000
     3   0   0   1   1
      3.2732950110    1.0000000000
     4   0   0   1   1
     13.5623763957    1.0000000000
     5   0   0   1   1
     60.6886467147    1.0000000000
     6   0   0   1   1
    264.5752683689    1.0000000000
     7   1   1   1   1
      1.0371611983    1.0000000000
     8   1   1   1   1
```

```
      2.8585249484    1.0000000000
     9   1   1   1   1
      6.1196896520    1.0000000000
    10   2   2   1   1
      1.8584783718    1.0000000000
    11   2   2   1   1
      7.3935965758    1.0000000000
    12   3   3   1   1
      1.6283490419    1.0000000000
    13   3   3   1   1
      5.4450970581    1.0000000000

# RI basis set for Ne (all-electron) relative DI metric: 3.5e-03
Ne  RI_aug-TZVP-MOLOPT-ae_N_RI_048_s_p_d_f_g_h_i_6_3_2_2_1_0_0_error_3.5e-03
   14
     1   0   0   1   1
      0.2019428740    1.0000000000
     2   0   0   1   1
      1.0869212167    1.0000000000
     3   0   0   1   1
      2.2354579893    1.0000000000
     4   0   0   1   1
     10.4956014537    1.0000000000
     5   0   0   1   1
     56.7884250848    1.0000000000
     6   0   0   1   1
    262.9848789341    1.0000000000
     7   1   1   1   1
      1.0625440891    1.0000000000
     8   1   1   1   1
      2.5500061729    1.0000000000
     9   1   1   1   1
      6.0758145865    1.0000000000
    10   2   2   1   1
      2.1320718184    1.0000000000
    11   2   2   1   1
      5.5012688025    1.0000000000
    12   3   3   1   1
      1.6329303585    1.0000000000
    13   3   3   1   1
      5.5664604624    1.0000000000
    14   4   4   1   1
      4.4844153752    1.0000000000

# RI basis set for Ne (all-electron) relative DI metric: 1.2e-03
Ne  RI_aug-TZVP-MOLOPT-ae_N_RI_056_s_p_d_f_g_h_i_6_4_3_2_1_0_0_error_1.2e-03
   16
     1   0   0   1   1
      0.5774757984    1.0000000000
     2   0   0   1   1
      1.6232168850    1.0000000000
     3   0   0   1   1
      4.8531429808    1.0000000000
     4   0   0   1   1
     18.2906288657    1.0000000000
     5   0   0   1   1
     71.2839471132    1.0000000000
     6   0   0   1   1
    269.6697310247    1.0000000000
     7   1   1   1   1
      0.7892217820    1.0000000000
     8   1   1   1   1
      2.0904110637    1.0000000000
     9   1   1   1   1
      5.9476450215    1.0000000000
    10   1   1   1   1
     17.3897120467    1.0000000000
    11   2   2   1   1
      0.9056956890    1.0000000000
    12   2   2   1   1
      3.0000298453    1.0000000000
    13   2   2   1   1
      9.1214424485    1.0000000000
    14   3   3   1   1
      1.6689077535    1.0000000000
    15   3   3   1   1
      5.6300671642    1.0000000000
    16   4   4   1   1
      4.4288823901    1.0000000000

# RI basis set for Ne (all-electron) relative DI metric: 5.7e-04
Ne  RI_aug-TZVP-MOLOPT-ae_N_RI_068_s_p_d_f_g_h_i_6_4_4_3_1_0_0_error_5.7e-04
   18
     1   0   0   1   1
      0.5008202856    1.0000000000
     2   0   0   1   1
      1.7990155030    1.0000000000
     3   0   0   1   1
      4.4833053934    1.0000000000
     4   0   0   1   1
     14.9533551009    1.0000000000
     5   0   0   1   1
     61.1963522452    1.0000000000
     6   0   0   1   1
    215.3367946816    1.0000000000
     7   1   1   1   1
      0.6279885109    1.0000000000
     8   1   1   1   1
      1.7703368411    1.0000000000
     9   1   1   1   1
      4.9302435583    1.0000000000
    10   1   1   1   1
     17.0629121258    1.0000000000
    11   2   2   1   1
      1.0121532973    1.0000000000
    12   2   2   1   1
      2.1317063778    1.0000000000
    13   2   2   1   1
      4.6769297800    1.0000000000
    14   2   2   1   1
     11.9698619229    1.0000000000
```

```
    15   3   3   1   1
      0.5722661388    1.0000000000
    16   3   3   1   1
      2.6426363347    1.0000000000
    17   3   3   1   1
      7.5004736342    1.0000000000
    18   4   4   1   1
      4.9723209504    1.0000000000

# RI basis set for Ne (all-electron) relative DI metric: 2.0e-04
Ne  RI_aug-TZVP-MOLOPT-ae_N_RI_077_s_p_d_f_g_h_i_6_4_4_3_2_0_0_error_2.0e-04
   19
     1   0   0   1   1
      0.4200060039    1.0000000000
     2   0   0   1   1
      1.6821224879    1.0000000000
     3   0   0   1   1
      3.1436680192    1.0000000000
     4   0   0   1   1
     13.1430733814    1.0000000000
     5   0   0   1   1
     62.5308213321    1.0000000000
     6   0   0   1   1
    246.9821494380    1.0000000000
     7   1   1   1   1
      0.6622853755    1.0000000000
     8   1   1   1   1
      1.7718182915    1.0000000000
     9   1   1   1   1
      4.4324214066    1.0000000000
    10   1   1   1   1
     15.1700542902    1.0000000000
    11   2   2   1   1
      0.7574931484    1.0000000000
    12   2   2   1   1
      1.8797810666    1.0000000000
    13   2   2   1   1
      4.8196678366    1.0000000000
    14   2   2   1   1
     17.1488382983    1.0000000000
    15   3   3   1   1
      1.1090219380    1.0000000000
    16   3   3   1   1
      2.7972741283    1.0000000000
    17   3   3   1   1
      6.9701605606    1.0000000000
    18   4   4   1   1
      1.4659311782    1.0000000000
    19   4   4   1   1
      6.4304735805    1.0000000000

# RI basis set for Ne (all-electron) relative DI metric: 2.7e-05
Ne  RI_aug-TZVP-MOLOPT-ae_N_RI_089_s_p_d_f_g_h_i_7_4_4_3_2_1_0_error_2.7e-05
   21
     1   0   0   1   1
      0.5481066716    1.0000000000
     2   0   0   1   1
      1.1683434151    1.0000000000
     3   0   0   1   1
      3.3620204772    1.0000000000
     4   0   0   1   1
      9.3802881658    1.0000000000
     5   0   0   1   1
     28.9475036702    1.0000000000
     6   0   0   1   1
     89.0446728689    1.0000000000
     7   0   0   1   1
    269.6646490396    1.0000000000
     8   1   1   1   1
      0.6730967245    1.0000000000
     9   1   1   1   1
      1.6435699346    1.0000000000
    10   1   1   1   1
      4.9441731652    1.0000000000
    11   1   1   1   1
     15.1262545305    1.0000000000
    12   2   2   1   1
      0.7530573837    1.0000000000
    13   2   2   1   1
      1.6242852285    1.0000000000
    14   2   2   1   1
      4.9032590009    1.0000000000
    15   2   2   1   1
     14.3085546627    1.0000000000
    16   3   3   1   1
      0.8799470996    1.0000000000
    17   3   3   1   1
      2.3187179365    1.0000000000
    18   3   3   1   1
      6.8446857727    1.0000000000
    19   4   4   1   1
      0.9745477703    1.0000000000
    20   4   4   1   1
      6.4590455777    1.0000000000
    21   5   5   1   1
      1.1056232814    1.0000000000

# RI basis set for Ne (all-electron) relative DI metric: 1.2e-05
Ne  RI_aug-TZVP-MOLOPT-ae_N_RI_104_s_p_d_f_g_h_i_7_5_5_4_2_1_0_error_1.2e-05
   24
     1   0   0   1   1
      0.4947522140    1.0000000000
     2   0   0   1   1
      1.1858743704    1.0000000000
     3   0   0   1   1
      3.1388769674    1.0000000000
     4   0   0   1   1
      8.9673455294    1.0000000000
     5   0   0   1   1
     28.4884749352    1.0000000000
     6   0   0   1   1
```

```
     88.5926356863    1.0000000000
     7   0   0   1   1
    269.2047099616    1.0000000000
     8   1   1   1   1
      0.5339110432    1.0000000000
     9   1   1   1   1
      1.5863650404    1.0000000000
    10   1   1   1   1
      4.4864470391    1.0000000000
    11   1   1   1   1
     15.8407442275    1.0000000000
    12   1   1   1   1
     48.9080541734    1.0000000000
    13   2   2   1   1
      0.5175284694    1.0000000000
    14   2   2   1   1
      1.0544813270    1.0000000000
    15   2   2   1   1
      2.4451233528    1.0000000000
    16   2   2   1   1
      5.1398440243    1.0000000000
    17   2   2   1   1
     13.5667918627    1.0000000000
    18   3   3   1   1
      0.6583302963    1.0000000000
    19   3   3   1   1
      1.8551253167    1.0000000000
    20   3   3   1   1
      3.9135169117    1.0000000000
    21   3   3   1   1
      7.7581322367    1.0000000000
    22   4   4   1   1
      0.8736452274    1.0000000000
    23   4   4   1   1
      6.0975268781    1.0000000000
    24   5   5   1   1
      0.8608216383    1.0000000000

# RI basis set for Ne (all-electron) relative DI metric: 5.8e-06
Ne  RI_aug-TZVP-MOLOPT-ae_N_RI_113_s_p_d_f_g_h_i_7_5_5_4_3_1_0_error_5.8e-06
   25
     1   0   0   1   1
      0.4771586132    1.0000000000
     2   0   0   1   1
      0.9652333488    1.0000000000
     3   0   0   1   1
      2.6328005066    1.0000000000
     4   0   0   1   1
      7.7067536043    1.0000000000
     5   0   0   1   1
     25.3045908851    1.0000000000
     6   0   0   1   1
     84.7214858405    1.0000000000
     7   0   0   1   1
    265.2366652186    1.0000000000
     8   1   1   1   1
      0.6393714377    1.0000000000
     9   1   1   1   1
      1.5238600431    1.0000000000
    10   1   1   1   1
      4.3467837123    1.0000000000
    11   1   1   1   1
     11.2727254773    1.0000000000
    12   1   1   1   1
     39.9299202236    1.0000000000
    13   2   2   1   1
      0.6344284299    1.0000000000
    14   2   2   1   1
      1.1777101782    1.0000000000
    15   2   2   1   1
      2.6909365640    1.0000000000
    16   2   2   1   1
      5.2012563563    1.0000000000
    17   2   2   1   1
     14.1326326914    1.0000000000
    18   3   3   1   1
      0.7901432335    1.0000000000
    19   3   3   1   1
      1.7565103850    1.0000000000
    20   3   3   1   1
      3.8818287140    1.0000000000
    21   3   3   1   1
      8.0447446335    1.0000000000
    22   4   4   1   1
      0.8334394184    1.0000000000
    23   4   4   1   1
      3.2105697574    1.0000000000
    24   4   4   1   1
      7.1054882942    1.0000000000
    25   5   5   1   1
      1.0193582738    1.0000000000

# RI basis set for Na (all-electron) relative DI metric: 3.8e-02
Na  RI_aug-SZV-MOLOPT-ae-mini_N_RI_005_s_p_d_f_g_h_i_2_1_0_0_0_0_0_error_3.8e-02
   3
     1   0   0   1   1
      0.1659950329    1.0000000000
     2   0   0   1   1
      3.4988769034    1.0000000000
     3   1   1   1   1
      9.4222426315    1.0000000000

# RI basis set for Na (all-electron) relative DI metric: 1.9e-03
Na  RI_aug-SZV-MOLOPT-ae-mini_N_RI_014_s_p_d_f_g_h_i_3_2_1_0_0_0_0_error_1.9e-03
   6
     1   0   0   1   1
      0.3305171263    1.0000000000
     2   0   0   1   1
      0.8697493974    1.0000000000
     3   0   0   1   1
      1.9321219344    1.0000000000
```

```
    4   1   1   1   1
     0.6188680630    1.0000000000
    5   1   1   1   1
     6.2243787904    1.0000000000
    6   2   2   1   1
     9.4317708295    1.0000000000

# RI basis set for Na (all-electron) relative DI metric: 7.2e-04
Na  RI_aug-SZV-MOLOPT-ae-mini_N_RI_019_s_p_d_f_g_h_i_3_2_2_0_0_0_0_error_7.2e-04
   7
    1   0   0   1   1
     0.3328919834    1.0000000000
    2   0   0   1   1
     0.9475613956    1.0000000000
    3   0   0   1   1
     2.3707872534    1.0000000000
    4   1   1   1   1
     0.6326303538    1.0000000000
    5   1   1   1   1
     6.0255216980    1.0000000000
    6   2   2   1   1
     0.2327810196    1.0000000000
    7   2   2   1   1
     9.0861714161    1.0000000000

# RI basis set for Na (all-electron) relative DI metric: 1.7e-04
Na  RI_aug-SZV-MOLOPT-ae-mini_N_RI_020_s_p_d_f_g_h_i_4_2_2_0_0_0_0_error_1.7e-04
   8
    1   0   0   1   1
     0.1956740974    1.0000000000
    2   0   0   1   1
     0.7280004203    1.0000000000
    3   0   0   1   1
     3.0101112539    1.0000000000
    4   0   0   1   1
    12.9907838033    1.0000000000
    5   1   1   1   1
     0.6442344598    1.0000000000
    6   1   1   1   1
     6.0090195654    1.0000000000
    7   2   2   1   1
     0.8182234386    1.0000000000
    8   2   2   1   1
     7.0230466319    1.0000000000

# RI basis set for Na (all-electron) relative DI metric: 3.9e-05
Na  RI_aug-SZV-MOLOPT-ae-mini_N_RI_028_s_p_d_f_g_h_i_4_3_3_0_0_0_0_error_3.9e-05
   10
    1   0   0   1   1
     0.1987987891    1.0000000000
    2   0   0   1   1
     0.9271273975    1.0000000000
    3   0   0   1   1
     2.4885559769    1.0000000000
    4   0   0   1   1
    11.8887685082    1.0000000000
    5   1   1   1   1
     0.3136972153    1.0000000000
    6   1   1   1   1
     0.8152318450    1.0000000000
    7   1   1   1   1
     4.7310207450    1.0000000000
    8   2   2   1   1
     0.2073404573    1.0000000000
    9   2   2   1   1
     0.7874476838    1.0000000000
   10   2   2   1   1
     7.4339450521    1.0000000000

# RI basis set for Na (all-electron) relative DI metric: 8.2e-07
Na  RI_aug-SZV-MOLOPT-ae-mini_N_RI_042_s_p_d_f_g_h_i_7_5_4_0_0_0_0_error_8.2e-07
   16
    1   0   0   1   1
     0.1243510767    1.0000000000
    2   0   0   1   1
     0.2896492398    1.0000000000
    3   0   0   1   1
     0.6819272224    1.0000000000
    4   0   0   1   1
     1.5848630386    1.0000000000
    5   0   0   1   1
     3.6827639027    1.0000000000
    6   0   0   1   1
     8.6094873363    1.0000000000
    7   0   0   1   1
    20.0672084093    1.0000000000
    8   1   1   1   1
     0.0920321819    1.0000000000
    9   1   1   1   1
     0.4244053257    1.0000000000
   10   1   1   1   1
     1.1119206412    1.0000000000
   11   1   1   1   1
     4.6534389842    1.0000000000
   12   1   1   1   1
    18.6668676400    1.0000000000
   13   2   2   1   1
     0.1337113219    1.0000000000
   14   2   2   1   1
     0.8782885904    1.0000000000
   15   2   2   1   1
     5.0672330221    1.0000000000
   16   2   2   1   1
    15.1367531967    1.0000000000

# RI basis set for Na (all-electron) relative DI metric: 2.4e-07
Na  RI_aug-SZV-MOLOPT-ae-mini_N_RI_055_s_p_d_f_g_h_i_7_6_6_0_0_0_0_error_2.4e-07
   19
    1   0   0   1   1
     0.1249999995    1.0000000000
    2   0   0   1   1
```

```
      0.2914106205    1.0000000000
     3   0   0   1   1
      0.6793612018    1.0000000000
     4   0   0   1   1
      1.5837845625    1.0000000000
     5   0   0   1   1
      3.6922531511    1.0000000000
     6   0   0   1   1
      8.6076942845    1.0000000000
     7   0   0   1   1
     20.0669883318    1.0000000000
     8   1   1   1   1
      0.1249999978    1.0000000000
     9   1   1   1   1
      0.3451631665    1.0000000000
    10   1   1   1   1
      0.9531009150    1.0000000000
    11   1   1   1   1
      2.6318026849    1.0000000000
    12   1   1   1   1
      7.2672108188    1.0000000000
    13   1   1   1   1
     20.0669883265    1.0000000000
    14   2   2   1   1
      0.1250000000    1.0000000000
    15   2   2   1   1
      0.3451631709    1.0000000000
    16   2   2   1   1
      0.9531009202    1.0000000000
    17   2   2   1   1
      2.6318026818    1.0000000000
    18   2   2   1   1
      7.2672107973    1.0000000000
    19   2   2   1   1
     20.0669883688    1.0000000000

# RI basis set for Na (all-electron) relative DI metric: 2.4e-02
Na  RI_aug-SZV-MOLOPT-ae_N_RI_009_s_p_d_f_g_h_i_3_2_0_0_0_0_0_error_2.4e-02
   5
     1   0   0   1   1
      0.4798297284    1.0000000000
     2   0   0   1   1
      2.0550990198    1.0000000000
     3   0   0   1   1
    130.3357741540    1.0000000000
     4   1   1   1   1
      0.5532086748    1.0000000000
     5   1   1   1   1
      7.7007017924    1.0000000000

# RI basis set for Na (all-electron) relative DI metric: 1.4e-03
Na  RI_aug-SZV-MOLOPT-ae_N_RI_023_s_p_d_f_g_h_i_4_3_2_0_0_0_0_error_1.4e-03
   9
     1   0   0   1   1
      0.5062487345    1.0000000000
     2   0   0   1   1
      2.7166170610    1.0000000000
     3   0   0   1   1
     14.9314761310    1.0000000000
     4   0   0   1   1
    121.9203963807    1.0000000000
     5   1   1   1   1
      0.6645949346    1.0000000000
     6   1   1   1   1
      5.1969137824    1.0000000000
     7   1   1   1   1
     15.2242234832    1.0000000000
     8   2   2   1   1
      0.2048028426    1.0000000000
     9   2   2   1   1
     10.6234344169    1.0000000000

# RI basis set for Na (all-electron) relative DI metric: 7.2e-05
Na  RI_aug-SZV-MOLOPT-ae_N_RI_028_s_p_d_f_g_h_i_6_4_2_0_0_0_0_error_7.2e-05
   12
     1   0   0   1   1
      0.0972240400    1.0000000000
     2   0   0   1   1
      0.5810912743    1.0000000000
     3   0   0   1   1
      2.2325909807    1.0000000000
     4   0   0   1   1
      3.9634813260    1.0000000000
     5   0   0   1   1
     26.2099468993    1.0000000000
     6   0   0   1   1
    131.7709777641    1.0000000000
     7   1   1   1   1
      0.3465160744    1.0000000000
     8   1   1   1   1
      0.9266454584    1.0000000000
     9   1   1   1   1
      4.2826126749    1.0000000000
    10   1   1   1   1
     24.0544975966    1.0000000000
    11   2   2   1   1
      0.7434881497    1.0000000000
    12   2   2   1   1
      7.4085082238    1.0000000000

# RI basis set for Na (all-electron) relative DI metric: 2.8e-05
Na  RI_aug-SZV-MOLOPT-ae_N_RI_033_s_p_d_f_g_h_i_6_4_3_0_0_0_0_error_2.8e-05
   13
     1   0   0   1   1
      0.0938891993    1.0000000000
     2   0   0   1   1
      0.5685845659    1.0000000000
     3   0   0   1   1
      2.1761013692    1.0000000000
     4   0   0   1   1
      3.8315733004    1.0000000000
```

```
      5   0   0   1   1
     25.7818840726    1.0000000000
      6   0   0   1   1
    131.6818782923    1.0000000000
      7   1   1   1   1
      0.3231018255    1.0000000000
      8   1   1   1   1
      0.8336905909    1.0000000000
      9   1   1   1   1
      4.6182603847    1.0000000000
     10   1   1   1   1
     24.4319205136    1.0000000000
     11   2   2   1   1
      0.1004302377    1.0000000000
     12   2   2   1   1
      0.7471696705    1.0000000000
     13   2   2   1   1
      7.4776468823    1.0000000000

# RI basis set for Na (all-electron) relative DI metric: 7.8e-06
Na  RI_aug-SZV-MOLOPT-ae_N_RI_050_s_p_d_f_g_h_i_6_5_3_2_0_0_0_error_7.8e-06
   16
      1   0   0   1   1
      0.1248701287    1.0000000000
      2   0   0   1   1
      0.5817364102    1.0000000000
      3   0   0   1   1
      1.6466585962    1.0000000000
      4   0   0   1   1
      4.0996485737    1.0000000000
      5   0   0   1   1
     23.8473181963    1.0000000000
      6   0   0   1   1
    106.5236860087    1.0000000000
      7   1   1   1   1
      0.1155081480    1.0000000000
      8   1   1   1   1
      0.3031130024    1.0000000000
      9   1   1   1   1
      1.0217320632    1.0000000000
     10   1   1   1   1
      4.5009918036    1.0000000000
     11   1   1   1   1
     30.0863272264    1.0000000000
     12   2   2   1   1
      0.0943312359    1.0000000000
     13   2   2   1   1
      0.8513108167    1.0000000000
     14   2   2   1   1
      7.2827116986    1.0000000000
     15   3   3   1   1
      0.3198801961    1.0000000000
     16   3   3   1   1
      1.8233632511    1.0000000000

# RI basis set for Na (all-electron) relative DI metric: 1.6e-06
Na  RI_aug-SZV-MOLOPT-ae_N_RI_063_s_p_d_f_g_h_i_7_6_3_2_1_0_0_error_1.6e-06
   19
      1   0   0   1   1
      0.0962155837    1.0000000000
      2   0   0   1   1
      0.2059399214    1.0000000000
      3   0   0   1   1
      0.7800942577    1.0000000000
      4   0   0   1   1
      2.7618605248    1.0000000000
      5   0   0   1   1
      9.7015520106    1.0000000000
      6   0   0   1   1
     36.4487264835    1.0000000000
      7   0   0   1   1
    138.9793070848    1.0000000000
      8   1   1   1   1
      0.1099208247    1.0000000000
      9   1   1   1   1
      0.2185840195    1.0000000000
     10   1   1   1   1
      0.8906016355    1.0000000000
     11   1   1   1   1
      2.9681717035    1.0000000000
     12   1   1   1   1
      5.9749488285    1.0000000000
     13   1   1   1   1
     33.7427160018    1.0000000000
     14   2   2   1   1
      0.0930101091    1.0000000000
     15   2   2   1   1
      0.7859260246    1.0000000000
     16   2   2   1   1
      7.5628083544    1.0000000000
     17   3   3   1   1
      0.4728549272    1.0000000000
     18   3   3   1   1
      1.8377351608    1.0000000000
     19   4   4   1   1
      0.4464535805    1.0000000000

# RI basis set for Na (all-electron) relative DI metric: 2.8e-02
Na  RI_aug-DZVP-MOLOPT-ae_N_RI_013_s_p_d_f_g_h_i_4_3_0_0_0_0_0_error_2.8e-02
   7
      1   0   0   1   1
      0.3548782787    1.0000000000
      2   0   0   1   1
      0.7528039463    1.0000000000
      3   0   0   1   1
     11.2823438496    1.0000000000
      4   0   0   1   1
     41.3926914307    1.0000000000
      5   1   1   1   1
      0.6910738743    1.0000000000
      6   1   1   1   1
```

```
      2.5421382962    1.0000000000
     7   1   1   1   1
      9.7702122514    1.0000000000

# RI basis set for Na (all-electron) relative DI metric: 1.4e-02
Na  RI_aug-DZVP-MOLOPT-ae_N_RI_023_s_p_d_f_g_h_i_4_3_2_0_0_0_0_error_1.4e-02
   9
     1   0   0   1   1
      0.6569658913    1.0000000000
     2   0   0   1   1
      2.2944863700    1.0000000000
     3   0   0   1   1
      7.5863584352    1.0000000000
     4   0   0   1   1
     28.9603368176    1.0000000000
     5   1   1   1   1
      0.8521499089    1.0000000000
     6   1   1   1   1
      1.9201916382    1.0000000000
     7   1   1   1   1
     10.3095645549    1.0000000000
     8   2   2   1   1
      4.0592321049    1.0000000000
     9   2   2   1   1
      8.2100385770    1.0000000000

# RI basis set for Na (all-electron) relative DI metric: 5.4e-03
Na  RI_aug-DZVP-MOLOPT-ae_N_RI_027_s_p_d_f_g_h_i_5_4_2_0_0_0_0_error_5.4e-03
   11
     1   0   0   1   1
      0.4078941031    1.0000000000
     2   0   0   1   1
      1.5413888754    1.0000000000
     3   0   0   1   1
      5.7371141558    1.0000000000
     4   0   0   1   1
     21.7252774020    1.0000000000
     5   0   0   1   1
     83.4817913728    1.0000000000
     6   1   1   1   1
      0.2737733419    1.0000000000
     7   1   1   1   1
      0.8704299307    1.0000000000
     8   1   1   1   1
      2.4492897507    1.0000000000
     9   1   1   1   1
      9.8191827499    1.0000000000
    10   2   2   1   1
      0.5944873286    1.0000000000
    11   2   2   1   1
      4.2528661252    1.0000000000

# RI basis set for Na (all-electron) relative DI metric: 9.7e-04
Na  RI_aug-DZVP-MOLOPT-ae_N_RI_039_s_p_d_f_g_h_i_5_4_3_1_0_0_0_error_9.7e-04
   13
     1   0   0   1   1
      0.5146403839    1.0000000000
     2   0   0   1   1
      1.9307149051    1.0000000000
     3   0   0   1   1
      6.5790309246    1.0000000000
     4   0   0   1   1
     21.3519120992    1.0000000000
     5   0   0   1   1
     81.0060520322    1.0000000000
     6   1   1   1   1
      0.2360573389    1.0000000000
     7   1   1   1   1
      0.7877452114    1.0000000000
     8   1   1   1   1
      2.7046165793    1.0000000000
     9   1   1   1   1
      9.3938319467    1.0000000000
    10   2   2   1   1
      0.1445260351    1.0000000000
    11   2   2   1   1
      0.6006678723    1.0000000000
    12   2   2   1   1
      4.5738096863    1.0000000000
    13   3   3   1   1
      2.4004644175    1.0000000000

# RI basis set for Na (all-electron) relative DI metric: 1.5e-04
Na  RI_aug-DZVP-MOLOPT-ae_N_RI_069_s_p_d_f_g_h_i_7_5_4_1_1_1_0_error_1.5e-04
   19
     1   0   0   1   1
      0.1198096619    1.0000000000
     2   0   0   1   1
      0.5048768535    1.0000000000
     3   0   0   1   1
      1.8006252956    1.0000000000
     4   0   0   1   1
      5.5015818959    1.0000000000
     5   0   0   1   1
     13.3426666348    1.0000000000
     6   0   0   1   1
     47.9204411554    1.0000000000
     7   0   0   1   1
    166.7035532803    1.0000000000
     8   1   1   1   1
      0.3263538067    1.0000000000
     9   1   1   1   1
      0.9542279643    1.0000000000
    10   1   1   1   1
      2.7893378159    1.0000000000
    11   1   1   1   1
      8.6465093952    1.0000000000
    12   1   1   1   1
     25.8148071677    1.0000000000
    13   2   2   1   1
      0.1967863522    1.0000000000
```

```
    14   2   2   1   1
      0.7163693288    1.0000000000
    15   2   2   1   1
      2.1623188339    1.0000000000
    16   2   2   1   1
      8.1586922343    1.0000000000
    17   3   3   1   1
      2.3732917676    1.0000000000
    18   4   4   1   1
      0.5154958653    1.0000000000
    19   5   5   1   1
      0.3097677618    1.0000000000

# RI basis set for Na (all-electron) relative DI metric: 1.6e-02
Na  RI_aug-TZVP-MOLOPT-ae_N_RI_025_s_p_d_f_g_h_i_4_3_1_1_0_0_0_error_1.6e-02
   9
     1   0   0   1   1
      0.5737786902    1.0000000000
     2   0   0   1   1
      1.6353724535    1.0000000000
     3   0   0   1   1
      9.9844651012    1.0000000000
     4   0   0   1   1
     54.0164854180    1.0000000000
     5   1   1   1   1
      0.8985118325    1.0000000000
     6   1   1   1   1
      2.5368786001    1.0000000000
     7   1   1   1   1
      7.2717100350    1.0000000000
     8   2   2   1   1
      2.7931882419    1.0000000000
     9   3   3   1   1
      3.0052673142    1.0000000000

# RI basis set for Na (all-electron) relative DI metric: 2.2e-03
Na  RI_aug-TZVP-MOLOPT-ae_N_RI_046_s_p_d_f_g_h_i_5_4_3_2_0_0_0_error_2.2e-03
   14
     1   0   0   1   1
      0.6150511570    1.0000000000
     2   0   0   1   1
      1.7051493610    1.0000000000
     3   0   0   1   1
      2.6980278999    1.0000000000
     4   0   0   1   1
      6.7090559481    1.0000000000
     5   0   0   1   1
     23.9020220499    1.0000000000
     6   1   1   1   1
      0.6683729754    1.0000000000
     7   1   1   1   1
      1.5760465006    1.0000000000
     8   1   1   1   1
      3.3831892886    1.0000000000
     9   1   1   1   1
      7.8874128807    1.0000000000
    10   2   2   1   1
      0.8462179544    1.0000000000
    11   2   2   1   1
      3.7273769663    1.0000000000
    12   2   2   1   1
     17.4318610851    1.0000000000
    13   3   3   1   1
      0.7671792391    1.0000000000
    14   3   3   1   1
      3.5522045146    1.0000000000

# RI basis set for Na (all-electron) relative DI metric: 2.7e-04
Na  RI_aug-TZVP-MOLOPT-ae_N_RI_059_s_p_d_f_g_h_i_6_5_3_2_1_0_0_error_2.7e-04
   17
     1   0   0   1   1
      0.1523521569    1.0000000000
     2   0   0   1   1
      0.7041579422    1.0000000000
     3   0   0   1   1
      2.6770632121    1.0000000000
     4   0   0   1   1
      7.6571946289    1.0000000000
     5   0   0   1   1
     29.2315972045    1.0000000000
     6   0   0   1   1
    139.6262280750    1.0000000000
     7   1   1   1   1
      0.1378190007    1.0000000000
     8   1   1   1   1
      0.6248620026    1.0000000000
     9   1   1   1   1
      2.3286291883    1.0000000000
    10   1   1   1   1
      5.9901571103    1.0000000000
    11   1   1   1   1
     13.8870914073    1.0000000000
    12   2   2   1   1
      1.1183492925    1.0000000000
    13   2   2   1   1
      2.8343798526    1.0000000000
    14   2   2   1   1
     16.4309632249    1.0000000000
    15   3   3   1   1
      0.8052075150    1.0000000000
    16   3   3   1   1
      3.2816004192    1.0000000000
    17   4   4   1   1
      2.4742737549    1.0000000000

# RI basis set for Na (all-electron) relative DI metric: 7.6e-05
Na  RI_aug-TZVP-MOLOPT-ae_N_RI_065_s_p_d_f_g_h_i_7_5_4_2_1_0_0_error_7.6e-05
   19
     1   0   0   1   1
      0.1087928572    1.0000000000
     2   0   0   1   1
```

```
      0.3702483176    1.0000000000
     3   0   0   1   1
      1.1728002416    1.0000000000
     4   0   0   1   1
      3.0260594117    1.0000000000
     5   0   0   1   1
      8.9109960421    1.0000000000
     6   0   0   1   1
     34.8512132354    1.0000000000
     7   0   0   1   1
    121.0420291629    1.0000000000
     8   1   1   1   1
      0.1630462475    1.0000000000
     9   1   1   1   1
      0.6729612470    1.0000000000
    10   1   1   1   1
      2.4600880770    1.0000000000
    11   1   1   1   1
      5.3591345043    1.0000000000
    12   1   1   1   1
     12.4191925111    1.0000000000
    13   2   2   1   1
      0.3969772490    1.0000000000
    14   2   2   1   1
      1.5326220073    1.0000000000
    15   2   2   1   1
      2.9796556406    1.0000000000
    16   2   2   1   1
     17.2999718852    1.0000000000
    17   3   3   1   1
      0.8869935434    1.0000000000
    18   3   3   1   1
      3.3891014037    1.0000000000
    19   4   4   1   1
      2.8759816146    1.0000000000

# RI basis set for Na (all-electron) relative DI metric: 3.3e-05
Na  RI_aug-TZVP-MOLOPT-ae_N_RI_087_s_p_d_f_g_h_i_7_6_5_4_1_0_0_error_3.3e-05
   23
     1   0   0   1   1
      0.1734105201    1.0000000000
     2   0   0   1   1
      0.5026019846    1.0000000000
     3   0   0   1   1
      1.4226085299    1.0000000000
     4   0   0   1   1
      3.8558945550    1.0000000000
     5   0   0   1   1
     10.7313926665    1.0000000000
     6   0   0   1   1
     31.8832405582    1.0000000000
     7   0   0   1   1
     92.4804961821    1.0000000000
     8   1   1   1   1
      0.1136645112    1.0000000000
     9   1   1   1   1
      0.2482343553    1.0000000000
    10   1   1   1   1
      0.8839750082    1.0000000000
    11   1   1   1   1
      3.1352388181    1.0000000000
    12   1   1   1   1
      5.9503271712    1.0000000000
    13   1   1   1   1
     20.7280878085    1.0000000000
    14   2   2   1   1
      0.1403314941    1.0000000000
    15   2   2   1   1
      0.4935536254    1.0000000000
    16   2   2   1   1
      1.7229945583    1.0000000000
    17   2   2   1   1
      3.1318695565    1.0000000000
    18   2   2   1   1
     14.7996786130    1.0000000000
    19   3   3   1   1
      0.1010979923    1.0000000000
    20   3   3   1   1
      0.3403085869    1.0000000000
    21   3   3   1   1
      1.1722446661    1.0000000000
    22   3   3   1   1
      3.7319016120    1.0000000000
    23   4   4   1   1
      2.7475340858    1.0000000000

# RI basis set for Na (all-electron) relative DI metric: 1.6e-05
Na  RI_aug-TZVP-MOLOPT-ae_N_RI_094_s_p_d_f_g_h_i_7_6_5_5_1_0_0_error_1.6e-05
   24
     1   0   0   1   1
      0.1379690051    1.0000000000
     2   0   0   1   1
      0.4757434633    1.0000000000
     3   0   0   1   1
      1.4412409818    1.0000000000
     4   0   0   1   1
      2.2408095829    1.0000000000
     5   0   0   1   1
      6.1824913434    1.0000000000
     6   0   0   1   1
     25.0040677343    1.0000000000
     7   0   0   1   1
     96.3640608050    1.0000000000
     8   1   1   1   1
      0.1087521954    1.0000000000
     9   1   1   1   1
      0.2421708135    1.0000000000
    10   1   1   1   1
      0.8677995946    1.0000000000
    11   1   1   1   1
      2.5211878692    1.0000000000
```

```
    12   1   1   1   1
      6.4419165865    1.0000000000
    13   1   1   1   1
     21.3600094650    1.0000000000
    14   2   2   1   1
      0.1245043641    1.0000000000
    15   2   2   1   1
      0.4724014835    1.0000000000
    16   2   2   1   1
      1.6699788102    1.0000000000
    17   2   2   1   1
      3.1446228990    1.0000000000
    18   2   2   1   1
     15.4096000597    1.0000000000
    19   3   3   1   1
      0.1046485725    1.0000000000
    20   3   3   1   1
      0.2570209082    1.0000000000
    21   3   3   1   1
      0.6378435612    1.0000000000
    22   3   3   1   1
      1.4835143337    1.0000000000
    23   3   3   1   1
      3.8558478308    1.0000000000
    24   4   4   1   1
      2.7157180157    1.0000000000

# RI basis set for Na (all-electron) relative DI metric: 4.3e-06
Na  RI_aug-TZVP-MOLOPT-ae_N_RI_117_s_p_d_f_g_h_i_7_6_6_5_3_0_0_error_4.3e-06
   27
     1   0   0   1   1
      0.1327196587    1.0000000000
     2   0   0   1   1
      0.4268266540    1.0000000000
     3   0   0   1   1
      1.4631424244    1.0000000000
     4   0   0   1   1
      2.3104509070    1.0000000000
     5   0   0   1   1
      5.6378490564    1.0000000000
     6   0   0   1   1
     20.0138246547    1.0000000000
     7   0   0   1   1
     83.7751346728    1.0000000000
     8   1   1   1   1
      0.1302483561    1.0000000000
     9   1   1   1   1
      0.4475250354    1.0000000000
    10   1   1   1   1
      1.2995001929    1.0000000000
    11   1   1   1   1
      2.6546297739    1.0000000000
    12   1   1   1   1
      8.3845279054    1.0000000000
    13   1   1   1   1
     20.5189335471    1.0000000000
    14   2   2   1   1
      0.1242322855    1.0000000000
    15   2   2   1   1
      0.3346059322    1.0000000000
    16   2   2   1   1
      0.9230487970    1.0000000000
    17   2   2   1   1
      2.5266980924    1.0000000000
    18   2   2   1   1
      5.5784521229    1.0000000000
    19   2   2   1   1
     15.0405073734    1.0000000000
    20   3   3   1   1
      0.1052549304    1.0000000000
    21   3   3   1   1
      0.2795957125    1.0000000000
    22   3   3   1   1
      0.7580076292    1.0000000000
    23   3   3   1   1
      1.8992066818    1.0000000000
    24   3   3   1   1
      4.4570017204    1.0000000000
    25   4   4   1   1
      0.0914638893    1.0000000000
    26   4   4   1   1
      0.6403793239    1.0000000000
    27   4   4   1   1
      2.8181169662    1.0000000000

# RI basis set for Mg (all-electron) relative DI metric: 3.4e-02
Mg  RI_aug-SZV-MOLOPT-ae-mini_N_RI_009_s_p_d_f_g_h_i_3_2_0_0_0_0_0_error_3.4e-02
   5
     1   0   0   1   1
      0.4206501406    1.0000000000
     2   0   0   1   1
      1.3859240259    1.0000000000
     3   0   0   1   1
     11.7020853632    1.0000000000
     4   1   1   1   1
      0.1591635569    1.0000000000
     5   1   1   1   1
     12.7576069267    1.0000000000

# RI basis set for Mg (all-electron) relative DI metric: 1.5e-02
Mg  RI_aug-SZV-MOLOPT-ae-mini_N_RI_015_s_p_d_f_g_h_i_6_3_0_0_0_0_0_error_1.5e-02
   9
     1   0   0   1   1
      0.0989860728    1.0000000000
     2   0   0   1   1
      0.2807687108    1.0000000000
     3   0   0   1   1
      0.6301160668    1.0000000000
     4   0   0   1   1
      1.7602737682    1.0000000000
     5   0   0   1   1
```

```
      11.9965250989    1.0000000000
     6   0   0   1   1
      32.1888849681    1.0000000000
     7   1   1   1   1
       0.1015634563    1.0000000000
     8   1   1   1   1
       0.2713061943    1.0000000000
     9   1   1   1   1
       7.0692745964    1.0000000000

# RI basis set for Mg (all-electron) relative DI metric: 1.1e-03
Mg  RI_aug-SZV-MOLOPT-ae-mini_N_RI_024_s_p_d_f_g_h_i_7_4_1_0_0_0_0_error_1.1e-03
   12
     1   0   0   1   1
       0.0995477887    1.0000000000
     2   0   0   1   1
       0.2591895416    1.0000000000
     3   0   0   1   1
       0.6858331831    1.0000000000
     4   0   0   1   1
       1.7619287537    1.0000000000
     5   0   0   1   1
       4.8731195849    1.0000000000
     6   0   0   1   1
      10.1532501366    1.0000000000
     7   0   0   1   1
      23.1858030745    1.0000000000
     8   1   1   1   1
       0.1178332773    1.0000000000
     9   1   1   1   1
       0.2972925399    1.0000000000
    10   1   1   1   1
       1.3761453597    1.0000000000
    11   1   1   1   1
       6.0795374773    1.0000000000
    12   2   2   1   1
      11.7369785276    1.0000000000

# RI basis set for Mg (all-electron) relative DI metric: 3.9e-06
Mg  RI_aug-SZV-MOLOPT-ae-mini_N_RI_034_s_p_d_f_g_h_i_7_4_3_0_0_0_0_error_3.9e-06
   14
     1   0   0   1   1
       0.0949380541    1.0000000000
     2   0   0   1   1
       0.2494633503    1.0000000000
     3   0   0   1   1
       0.7039653384    1.0000000000
     4   0   0   1   1
       1.7723919038    1.0000000000
     5   0   0   1   1
       4.4222552582    1.0000000000
     6   0   0   1   1
      10.3423231831    1.0000000000
     7   0   0   1   1
      24.7625976405    1.0000000000
     8   1   1   1   1
       0.1058009809    1.0000000000
     9   1   1   1   1
       0.3692706196    1.0000000000
    10   1   1   1   1
       1.0607496860    1.0000000000
    11   1   1   1   1
       6.5518515786    1.0000000000
    12   2   2   1   1
       0.2432142270    1.0000000000
    13   2   2   1   1
       1.2580402050    1.0000000000
    14   2   2   1   1
       8.5016927123    1.0000000000

# RI basis set for Mg (all-electron) relative DI metric: 1.6e-06
Mg  RI_aug-SZV-MOLOPT-ae-mini_N_RI_037_s_p_d_f_g_h_i_7_5_3_0_0_0_0_error_1.6e-06
   15
     1   0   0   1   1
       0.0985221237    1.0000000000
     2   0   0   1   1
       0.2577014524    1.0000000000
     3   0   0   1   1
       0.7021029601    1.0000000000
     4   0   0   1   1
       1.7700270827    1.0000000000
     5   0   0   1   1
       4.4299168539    1.0000000000
     6   0   0   1   1
      10.3414234164    1.0000000000
     7   0   0   1   1
      24.7687749004    1.0000000000
     8   1   1   1   1
       0.1053783012    1.0000000000
     9   1   1   1   1
       0.3559938100    1.0000000000
    10   1   1   1   1
       1.3186187982    1.0000000000
    11   1   1   1   1
       5.6209843447    1.0000000000
    12   1   1   1   1
      24.3289735305    1.0000000000
    13   2   2   1   1
       0.2645579050    1.0000000000
    14   2   2   1   1
       0.9777913902    1.0000000000
    15   2   2   1   1
       9.3237396067    1.0000000000

# RI basis set for Mg (all-electron) relative DI metric: 2.9e-07
Mg  RI_aug-SZV-MOLOPT-ae-mini_N_RI_045_s_p_d_f_g_h_i_7_6_4_0_0_0_0_error_2.9e-07
   17
     1   0   0   1   1
       0.0963879662    1.0000000000
     2   0   0   1   1
       0.2536080657    1.0000000000
```

```
     3   0   0   1   1
      0.7040003066    1.0000000000
     4   0   0   1   1
      1.7676155648    1.0000000000
     5   0   0   1   1
      4.3053256701    1.0000000000
     6   0   0   1   1
     10.3829981799    1.0000000000
     7   0   0   1   1
     25.1423612667    1.0000000000
     8   1   1   1   1
      0.1021068101    1.0000000000
     9   1   1   1   1
      0.3354598715    1.0000000000
    10   1   1   1   1
      1.0579337882    1.0000000000
    11   1   1   1   1
      3.0536115007    1.0000000000
    12   1   1   1   1
      8.4165490950    1.0000000000
    13   1   1   1   1
     25.1445541017    1.0000000000
    14   2   2   1   1
      0.1308743527    1.0000000000
    15   2   2   1   1
      1.0412861910    1.0000000000
    16   2   2   1   1
      6.3866393058    1.0000000000
    17   2   2   1   1
     15.8676238442    1.0000000000

# RI basis set for Mg (all-electron) relative DI metric: 1.3e-02
Mg  RI_aug-SZV-MOLOPT-ae_N_RI_020_s_p_d_f_g_h_i_6_3_1_0_0_0_0_error_1.3e-02
   10
     1   0   0   1   1
      0.0935169191    1.0000000000
     2   0   0   1   1
      0.2582763859    1.0000000000
     3   0   0   1   1
      1.4536999722    1.0000000000
     4   0   0   1   1
     10.1114146566    1.0000000000
     5   0   0   1   1
     43.0502525213    1.0000000000
     6   0   0   1   1
    186.3152522419    1.0000000000
     7   1   1   1   1
      0.1772203472    1.0000000000
     8   1   1   1   1
      0.6122203844    1.0000000000
     9   1   1   1   1
      7.3760092471    1.0000000000
    10   2   2   1   1
      0.1678555406    1.0000000000

# RI basis set for Mg (all-electron) relative DI metric: 4.3e-03
Mg  RI_aug-SZV-MOLOPT-ae_N_RI_025_s_p_d_f_g_h_i_6_3_2_0_0_0_0_error_4.3e-03
   11
     1   0   0   1   1
      0.1012791075    1.0000000000
     2   0   0   1   1
      0.3096646188    1.0000000000
     3   0   0   1   1
      1.5094073031    1.0000000000
     4   0   0   1   1
      7.5839993976    1.0000000000
     5   0   0   1   1
     36.6261931303    1.0000000000
     6   0   0   1   1
    179.4004518358    1.0000000000
     7   1   1   1   1
      0.0939384635    1.0000000000
     8   1   1   1   1
      0.5257141015    1.0000000000
     9   1   1   1   1
      4.1101923551    1.0000000000
    10   2   2   1   1
      0.1946556418    1.0000000000
    11   2   2   1   1
     12.1555511383    1.0000000000

# RI basis set for Mg (all-electron) relative DI metric: 7.7e-04
Mg  RI_aug-SZV-MOLOPT-ae_N_RI_028_s_p_d_f_g_h_i_6_4_2_0_0_0_0_error_7.7e-04
   12
     1   0   0   1   1
      0.1004487933    1.0000000000
     2   0   0   1   1
      0.3744840272    1.0000000000
     3   0   0   1   1
      2.0748269278    1.0000000000
     4   0   0   1   1
      7.4338102455    1.0000000000
     5   0   0   1   1
     39.8045117870    1.0000000000
     6   0   0   1   1
    174.4518111622    1.0000000000
     7   1   1   1   1
      0.1614575159    1.0000000000
     8   1   1   1   1
      0.4172997669    1.0000000000
     9   1   1   1   1
      7.0512881106    1.0000000000
    10   1   1   1   1
     34.2432819087    1.0000000000
    11   2   2   1   1
      0.2036911786    1.0000000000
    12   2   2   1   1
     12.0275842468    1.0000000000

# RI basis set for Mg (all-electron) relative DI metric: 3.6e-04
Mg  RI_aug-SZV-MOLOPT-ae_N_RI_033_s_p_d_f_g_h_i_6_4_3_0_0_0_0_error_3.6e-04
```

```
   13
    1   0   0   1   1
      0.0992065991    1.0000000000
    2   0   0   1   1
      0.3928761741    1.0000000000
    3   0   0   1   1
      2.2386393024    1.0000000000
    4   0   0   1   1
      6.4925744784    1.0000000000
    5   0   0   1   1
     37.8351098925    1.0000000000
    6   0   0   1   1
    172.0316414718    1.0000000000
    7   1   1   1   1
      0.2003304428    1.0000000000
    8   1   1   1   1
      0.3824897523    1.0000000000
    9   1   1   1   1
      5.1786169658    1.0000000000
   10   1   1   1   1
     20.3348287774    1.0000000000
   11   2   2   1   1
      0.1507903626    1.0000000000
   12   2   2   1   1
      0.8954827636    1.0000000000
   13   2   2   1   1
      9.1854836847    1.0000000000

# RI basis set for Mg (all-electron) relative DI metric: 1.2e-05
Mg  RI_aug-SZV-MOLOPT-ae_N_RI_037_s_p_d_f_g_h_i_7_5_3_0_0_0_0_error_1.2e-05
   15
    1   0   0   1   1
      0.1281101492    1.0000000000
    2   0   0   1   1
      0.3865424577    1.0000000000
    3   0   0   1   1
      1.4544494842    1.0000000000
    4   0   0   1   1
      5.2049127188    1.0000000000
    5   0   0   1   1
     16.9312483596    1.0000000000
    6   0   0   1   1
     55.1478962316    1.0000000000
    7   0   0   1   1
    179.3034513557    1.0000000000
    8   1   1   1   1
      0.1638039648    1.0000000000
    9   1   1   1   1
      0.3196976759    1.0000000000
   10   1   1   1   1
      1.6211938390    1.0000000000
   11   1   1   1   1
      5.6997794500    1.0000000000
   12   1   1   1   1
     32.9079665694    1.0000000000
   13   2   2   1   1
      0.1852188514    1.0000000000
   14   2   2   1   1
      1.1019882687    1.0000000000
   15   2   2   1   1
      9.3363931765    1.0000000000

# RI basis set for Mg (all-electron) relative DI metric: 1.8e-06
Mg  RI_aug-SZV-MOLOPT-ae_N_RI_044_s_p_d_f_g_h_i_7_5_3_1_0_0_0_error_1.8e-06
   16
    1   0   0   1   1
      0.1460585692    1.0000000000
    2   0   0   1   1
      0.3496283230    1.0000000000
    3   0   0   1   1
      1.2122062334    1.0000000000
    4   0   0   1   1
      4.4737213171    1.0000000000
    5   0   0   1   1
     16.5736853767    1.0000000000
    6   0   0   1   1
     54.9926172174    1.0000000000
    7   0   0   1   1
    179.5915948772    1.0000000000
    8   1   1   1   1
      0.1560520526    1.0000000000
    9   1   1   1   1
      0.3415925326    1.0000000000
   10   1   1   1   1
      1.3225434816    1.0000000000
   11   1   1   1   1
      5.8255423540    1.0000000000
   12   1   1   1   1
     40.0555013896    1.0000000000
   13   2   2   1   1
      0.1845178658    1.0000000000
   14   2   2   1   1
      1.0229170574    1.0000000000
   15   2   2   1   1
      9.2743041849    1.0000000000
   16   3   3   1   1
      0.6047690570    1.0000000000

# RI basis set for Mg (all-electron) relative DI metric: 6.7e-07
Mg  RI_aug-SZV-MOLOPT-ae_N_RI_071_s_p_d_f_g_h_i_7_6_6_1_1_0_0_error_6.7e-07
   21
    1   0   0   1   1
      0.1470993640    1.0000000000
    2   0   0   1   1
      0.3919839257    1.0000000000
    3   0   0   1   1
      1.2397457406    1.0000000000
    4   0   0   1   1
      4.1261745156    1.0000000000
    5   0   0   1   1
     16.4831001493    1.0000000000
```

```
    6   0   0   1   1
    54.9228246121    1.0000000000
    7   0   0   1   1
   179.1648333435    1.0000000000
    8   1   1   1   1
     0.1620360469    1.0000000000
    9   1   1   1   1
     0.3348497582    1.0000000000
   10   1   1   1   1
     1.3491537401    1.0000000000
   11   1   1   1   1
     4.6343508467    1.0000000000
   12   1   1   1   1
    13.4452288646    1.0000000000
   13   1   1   1   1
    43.7772490950    1.0000000000
   14   2   2   1   1
     0.1055202596    1.0000000000
   15   2   2   1   1
     0.2047547223    1.0000000000
   16   2   2   1   1
     0.5704710369    1.0000000000
   17   2   2   1   1
     1.4756227479    1.0000000000
   18   2   2   1   1
     3.7038459301    1.0000000000
   19   2   2   1   1
    11.0178951491    1.0000000000
   20   3   3   1   1
     0.6627048108    1.0000000000
   21   4   4   1   1
     0.5536024977    1.0000000000

# RI basis set for Mg (all-electron) relative DI metric: 3.2e-07
Mg  RI_aug-SZV-MOLOPT-ae_N_RI_085_s_p_d_f_g_h_i_7_6_6_3_1_0_0_error_3.2e-07
  23
    1   0   0   1   1
     0.1430990131    1.0000000000
    2   0   0   1   1
     0.3506238762    1.0000000000
    3   0   0   1   1
     1.1119615396    1.0000000000
    4   0   0   1   1
     3.7356512607    1.0000000000
    5   0   0   1   1
    16.2735292823    1.0000000000
    6   0   0   1   1
    54.8664564841    1.0000000000
    7   0   0   1   1
   179.1541902964    1.0000000000
    8   1   1   1   1
     0.1773520167    1.0000000000
    9   1   1   1   1
     0.2932002740    1.0000000000
   10   1   1   1   1
     1.2181544646    1.0000000000
   11   1   1   1   1
     4.4157559951    1.0000000000
   12   1   1   1   1
    13.4361936675    1.0000000000
   13   1   1   1   1
    43.5765772557    1.0000000000
   14   2   2   1   1
     0.1047461753    1.0000000000
   15   2   2   1   1
     0.2064365520    1.0000000000
   16   2   2   1   1
     0.5709921959    1.0000000000
   17   2   2   1   1
     1.4781757622    1.0000000000
   18   2   2   1   1
     3.7117492770    1.0000000000
   19   2   2   1   1
    10.9125899811    1.0000000000
   20   3   3   1   1
     0.0996024935    1.0000000000
   21   3   3   1   1
     0.5243711476    1.0000000000
   22   3   3   1   1
     2.2799526161    1.0000000000
   23   4   4   1   1
     0.5530610059    1.0000000000

# RI basis set for Mg (all-electron) relative DI metric: 1.5e-07
Mg  RI_aug-SZV-MOLOPT-ae_N_RI_110_s_p_d_f_g_h_i_7_6_6_4_3_0_0_error_1.5e-07
  26
    1   0   0   1   1
     0.1480731673    1.0000000000
    2   0   0   1   1
     0.3014458777    1.0000000000
    3   0   0   1   1
     0.9998156051    1.0000000000
    4   0   0   1   1
     3.6861447325    1.0000000000
    5   0   0   1   1
    13.0321292333    1.0000000000
    6   0   0   1   1
    48.1472500386    1.0000000000
    7   0   0   1   1
   179.6978603612    1.0000000000
    8   1   1   1   1
     0.1614848718    1.0000000000
    9   1   1   1   1
     0.3342061551    1.0000000000
   10   1   1   1   1
     1.3256343409    1.0000000000
   11   1   1   1   1
     4.5985613356    1.0000000000
   12   1   1   1   1
    14.6672611658    1.0000000000
   13   1   1   1   1
```

```
    44.0679644901    1.0000000000
   14   2   2   1   1
     0.1097911670    1.0000000000
   15   2   2   1   1
     0.1970059818    1.0000000000
   16   2   2   1   1
     0.5645391969    1.0000000000
   17   2   2   1   1
     1.4730506145    1.0000000000
   18   2   2   1   1
     3.7155301232    1.0000000000
   19   2   2   1   1
    11.3527053960    1.0000000000
   20   3   3   1   1
     0.1309384823    1.0000000000
   21   3   3   1   1
     0.3299111137    1.0000000000
   22   3   3   1   1
     0.8583701791    1.0000000000
   23   3   3   1   1
     2.2715126479    1.0000000000
   24   4   4   1   1
     0.1045898353    1.0000000000
   25   4   4   1   1
     0.3337529588    1.0000000000
   26   4   4   1   1
     0.8175883887    1.0000000000

# RI basis set for Mg (all-electron) relative DI metric: 1.9e-02
Mg  RI_aug-DZVP-MOLOPT-ae_N_RI_023_s_p_d_f_g_h_i_4_3_2_0_0_0_0_error_1.9e-02
   9
    1   0   0   1   1
     0.7603394419    1.0000000000
    2   0   0   1   1
     5.3374659424    1.0000000000
    3   0   0   1   1
    18.6955198677    1.0000000000
    4   0   0   1   1
   130.4593963702    1.0000000000
    5   1   1   1   1
     0.2039056354    1.0000000000
    6   1   1   1   1
     0.5177019228    1.0000000000
    7   1   1   1   1
     9.9848935127    1.0000000000
    8   2   2   1   1
     0.2793855126    1.0000000000
    9   2   2   1   1
    13.4574256888    1.0000000000

# RI basis set for Mg (all-electron) relative DI metric: 7.0e-03
Mg  RI_aug-DZVP-MOLOPT-ae_N_RI_034_s_p_d_f_g_h_i_7_4_3_0_0_0_0_error_7.0e-03
   14
    1   0   0   1   1
     0.0800509203    1.0000000000
    2   0   0   1   1
     0.2572866795    1.0000000000
    3   0   0   1   1
     0.8531864589    1.0000000000
    4   0   0   1   1
     3.0704266537    1.0000000000
    5   0   0   1   1
    12.5087798545    1.0000000000
    6   0   0   1   1
    47.1590962299    1.0000000000
    7   0   0   1   1
   178.3930072706    1.0000000000
    8   1   1   1   1
     0.1074254648    1.0000000000
    9   1   1   1   1
     0.3744458606    1.0000000000
   10   1   1   1   1
     1.9888841272    1.0000000000
   11   1   1   1   1
     5.6240012994    1.0000000000
   12   2   2   1   1
     0.1943351809    1.0000000000
   13   2   2   1   1
     0.8300856231    1.0000000000
   14   2   2   1   1
    10.0746342527    1.0000000000

# RI basis set for Mg (all-electron) relative DI metric: 2.9e-03
Mg  RI_aug-DZVP-MOLOPT-ae_N_RI_046_s_p_d_f_g_h_i_7_4_4_1_0_0_0_error_2.9e-03
   16
    1   0   0   1   1
     0.0931563396    1.0000000000
    2   0   0   1   1
     0.2641069877    1.0000000000
    3   0   0   1   1
     0.9078628918    1.0000000000
    4   0   0   1   1
     3.2108384732    1.0000000000
    5   0   0   1   1
    11.6819633128    1.0000000000
    6   0   0   1   1
    45.7934931517    1.0000000000
    7   0   0   1   1
   177.1286129758    1.0000000000
    8   1   1   1   1
     0.1584423242    1.0000000000
    9   1   1   1   1
     0.2735407060    1.0000000000
   10   1   1   1   1
     1.0937729024    1.0000000000
   11   1   1   1   1
     8.7244543645    1.0000000000
   12   2   2   1   1
     0.1686955262    1.0000000000
   13   2   2   1   1
     0.5556460546    1.0000000000
```

```
    14   2   2   1   1
      1.3988660832    1.0000000000
    15   2   2   1   1
      8.4985631783    1.0000000000
    16   3   3   1   1
      1.7163613523    1.0000000000

# RI basis set for Mg (all-electron) relative DI metric: 2.3e-04
Mg  RI_aug-DZVP-MOLOPT-ae_N_RI_056_s_p_d_f_g_h_i_7_5_4_2_0_0_0_error_2.3e-04
   18
     1   0   0   1   1
      0.0930648348    1.0000000000
     2   0   0   1   1
      0.2612576038    1.0000000000
     3   0   0   1   1
      0.9525819054    1.0000000000
     4   0   0   1   1
      3.5659640832    1.0000000000
     5   0   0   1   1
     11.2921527489    1.0000000000
     6   0   0   1   1
     45.1627037356    1.0000000000
     7   0   0   1   1
    176.7990650641    1.0000000000
     8   1   1   1   1
      0.1353490055    1.0000000000
     9   1   1   1   1
      0.2714239068    1.0000000000
    10   1   1   1   1
      1.2937020485    1.0000000000
    11   1   1   1   1
      7.8204122556    1.0000000000
    12   1   1   1   1
     30.3270524645    1.0000000000
    13   2   2   1   1
      0.1644684766    1.0000000000
    14   2   2   1   1
      0.5864912306    1.0000000000
    15   2   2   1   1
      1.3794441451    1.0000000000
    16   2   2   1   1
      8.1530454924    1.0000000000
    17   3   3   1   1
      0.1472680895    1.0000000000
    18   3   3   1   1
      2.2268858235    1.0000000000

# RI basis set for Mg (all-electron) relative DI metric: 1.0e-04
Mg  RI_aug-DZVP-MOLOPT-ae_N_RI_059_s_p_d_f_g_h_i_7_6_4_2_0_0_0_error_1.0e-04
   19
     1   0   0   1   1
      0.0908612009    1.0000000000
     2   0   0   1   1
      0.2589084179    1.0000000000
     3   0   0   1   1
      0.8956736272    1.0000000000
     4   0   0   1   1
      3.1514926145    1.0000000000
     5   0   0   1   1
      9.8281055195    1.0000000000
     6   0   0   1   1
     40.7550922275    1.0000000000
     7   0   0   1   1
    171.6940869748    1.0000000000
     8   1   1   1   1
      0.1193131991    1.0000000000
     9   1   1   1   1
      0.2782673222    1.0000000000
    10   1   1   1   1
      1.0710878502    1.0000000000
    11   1   1   1   1
      3.5284953493    1.0000000000
    12   1   1   1   1
     10.6298838808    1.0000000000
    13   1   1   1   1
     37.4676499204    1.0000000000
    14   2   2   1   1
      0.1896385913    1.0000000000
    15   2   2   1   1
      0.5984033887    1.0000000000
    16   2   2   1   1
      1.0888888804    1.0000000000
    17   2   2   1   1
      8.5628882855    1.0000000000
    18   3   3   1   1
      0.1267635174    1.0000000000
    19   3   3   1   1
      2.2998506615    1.0000000000

# RI basis set for Mg (all-electron) relative DI metric: 4.2e-05
Mg  RI_aug-DZVP-MOLOPT-ae_N_RI_066_s_p_d_f_g_h_i_7_6_4_3_0_0_0_error_4.2e-05
   20
     1   0   0   1   1
      0.0917306398    1.0000000000
     2   0   0   1   1
      0.2595362241    1.0000000000
     3   0   0   1   1
      0.8699318295    1.0000000000
     4   0   0   1   1
      2.9242679432    1.0000000000
     5   0   0   1   1
      9.5444273515    1.0000000000
     6   0   0   1   1
     38.9209654676    1.0000000000
     7   0   0   1   1
    168.8633232212    1.0000000000
     8   1   1   1   1
      0.1134538180    1.0000000000
     9   1   1   1   1
      0.2410529392    1.0000000000
    10   1   1   1   1
```

```
      0.9624711619    1.0000000000
    11   1   1   1   1
      2.2921937815    1.0000000000
    12   1   1   1   1
      5.9021175247    1.0000000000
    13   1   1   1   1
     30.2643630423    1.0000000000
    14   2   2   1   1
      0.1857058861    1.0000000000
    15   2   2   1   1
      0.5689795561    1.0000000000
    16   2   2   1   1
      1.2503384786    1.0000000000
    17   2   2   1   1
      8.1639263990    1.0000000000
    18   3   3   1   1
      0.1558660930    1.0000000000
    19   3   3   1   1
      0.6789715720    1.0000000000
    20   3   3   1   1
      2.9173155221    1.0000000000

# RI basis set for Mg (all-electron) relative DI metric: 1.2e-05
Mg  RI_aug-DZVP-MOLOPT-ae_N_RI_078_s_p_d_f_g_h_i_7_6_5_4_0_0_0_error_1.2e-05
   22
     1   0   0   1   1
      0.0921598034    1.0000000000
     2   0   0   1   1
      0.2549440642    1.0000000000
     3   0   0   1   1
      1.1168644756    1.0000000000
     4   0   0   1   1
      3.0521659816    1.0000000000
     5   0   0   1   1
      9.0107234064    1.0000000000
     6   0   0   1   1
     35.1532849666    1.0000000000
     7   0   0   1   1
    159.3312262806    1.0000000000
     8   1   1   1   1
      0.1074788166    1.0000000000
     9   1   1   1   1
      0.2778720506    1.0000000000
    10   1   1   1   1
      0.7227311939    1.0000000000
    11   1   1   1   1
      2.1988035487    1.0000000000
    12   1   1   1   1
      6.2219867834    1.0000000000
    13   1   1   1   1
     40.1040437401    1.0000000000
    14   2   2   1   1
      0.1884075885    1.0000000000
    15   2   2   1   1
      0.5298643845    1.0000000000
    16   2   2   1   1
      1.4887968574    1.0000000000
    17   2   2   1   1
      4.0984201700    1.0000000000
    18   2   2   1   1
     15.3142525744    1.0000000000
    19   3   3   1   1
      0.1243841909    1.0000000000
    20   3   3   1   1
      0.2672994767    1.0000000000
    21   3   3   1   1
      0.7121912126    1.0000000000
    22   3   3   1   1
      2.8995781052    1.0000000000

# RI basis set for Mg (all-electron) relative DI metric: 5.2e-06
Mg  RI_aug-DZVP-MOLOPT-ae_N_RI_112_s_p_d_f_g_h_i_7_6_5_5_3_0_0_error_5.2e-06
   26
     1   0   0   1   1
      0.0915334733    1.0000000000
     2   0   0   1   1
      0.3088832963    1.0000000000
     3   0   0   1   1
      0.9828741222    1.0000000000
     4   0   0   1   1
      2.6900845553    1.0000000000
     5   0   0   1   1
      8.6598496317    1.0000000000
     6   0   0   1   1
     32.2052584150    1.0000000000
     7   0   0   1   1
    129.9084981333    1.0000000000
     8   1   1   1   1
      0.1153545866    1.0000000000
     9   1   1   1   1
      0.2508597405    1.0000000000
    10   1   1   1   1
      0.7181941383    1.0000000000
    11   1   1   1   1
      2.0539463997    1.0000000000
    12   1   1   1   1
      6.1476632948    1.0000000000
    13   1   1   1   1
     41.9201386813    1.0000000000
    14   2   2   1   1
      0.1966578143    1.0000000000
    15   2   2   1   1
      0.4585503822    1.0000000000
    16   2   2   1   1
      1.7500878482    1.0000000000
    17   2   2   1   1
      4.0782194048    1.0000000000
    18   2   2   1   1
     18.2299210175    1.0000000000
    19   3   3   1   1
      0.1338459290    1.0000000000
```

```
    20   3   3   1   1
      0.3082687404    1.0000000000
    21   3   3   1   1
      0.7131538177    1.0000000000
    22   3   3   1   1
      1.5922483256    1.0000000000
    23   3   3   1   1
      4.0819812836    1.0000000000
    24   4   4   1   1
      0.1580287875    1.0000000000
    25   4   4   1   1
      0.3898881352    1.0000000000
    26   4   4   1   1
      0.9376332299    1.0000000000

# RI basis set for Mg (all-electron) relative DI metric: 4.3e-02
Mg  RI_aug-TZVP-MOLOPT-ae_N_RI_025_s_p_d_f_g_h_i_6_3_2_0_0_0_0_error_4.3e-02
   11
     1   0   0   1   1
      0.0900463612    1.0000000000
     2   0   0   1   1
      0.3215677672    1.0000000000
     3   0   0   1   1
      1.1465293418    1.0000000000
     4   0   0   1   1
      5.3198961236    1.0000000000
     5   0   0   1   1
     37.0111871045    1.0000000000
     6   0   0   1   1
    181.1258800064    1.0000000000
     7   1   1   1   1
      0.2039372996    1.0000000000
     8   1   1   1   1
      1.2009675039    1.0000000000
     9   1   1   1   1
      8.5538835478    1.0000000000
    10   2   2   1   1
      0.3810609557    1.0000000000
    11   2   2   1   1
      6.7865536540    1.0000000000

# RI basis set for Mg (all-electron) relative DI metric: 9.2e-03
Mg  RI_aug-TZVP-MOLOPT-ae_N_RI_044_s_p_d_f_g_h_i_6_3_3_2_0_0_0_error_9.2e-03
   14
     1   0   0   1   1
      0.0945633007    1.0000000000
     2   0   0   1   1
      0.3505014859    1.0000000000
     3   0   0   1   1
      1.4059808254    1.0000000000
     4   0   0   1   1
      5.8459986429    1.0000000000
     5   0   0   1   1
     34.4121975385    1.0000000000
     6   0   0   1   1
    177.1057440086    1.0000000000
     7   1   1   1   1
      0.2582398392    1.0000000000
     8   1   1   1   1
      1.0938484137    1.0000000000
     9   1   1   1   1
      4.6280827446    1.0000000000
    10   2   2   1   1
      0.2549053785    1.0000000000
    11   2   2   1   1
      2.8208884451    1.0000000000
    12   2   2   1   1
     10.5220420116    1.0000000000
    13   3   3   1   1
      0.1231694970    1.0000000000
    14   3   3   1   1
      5.6905416713    1.0000000000

# RI basis set for Mg (all-electron) relative DI metric: 3.4e-03
Mg  RI_aug-TZVP-MOLOPT-ae_N_RI_047_s_p_d_f_g_h_i_6_4_3_2_0_0_0_error_3.4e-03
   15
     1   0   0   1   1
      0.0807318273    1.0000000000
     2   0   0   1   1
      0.3607080557    1.0000000000
     3   0   0   1   1
      1.7726657987    1.0000000000
     4   0   0   1   1
      6.3543059728    1.0000000000
     5   0   0   1   1
     33.3080906915    1.0000000000
     6   0   0   1   1
    175.4152751927    1.0000000000
     7   1   1   1   1
      0.1762582677    1.0000000000
     8   1   1   1   1
      0.5898857582    1.0000000000
     9   1   1   1   1
      2.1623019015    1.0000000000
    10   1   1   1   1
      6.6132134070    1.0000000000
    11   2   2   1   1
      0.2631207100    1.0000000000
    12   2   2   1   1
      3.1471670963    1.0000000000
    13   2   2   1   1
     11.3381027406    1.0000000000
    14   3   3   1   1
      0.1972434468    1.0000000000
    15   3   3   1   1
      4.1486947963    1.0000000000

# RI basis set for Mg (all-electron) relative DI metric: 1.3e-03
Mg  RI_aug-TZVP-MOLOPT-ae_N_RI_052_s_p_d_f_g_h_i_6_4_4_2_0_0_0_error_1.3e-03
   16
     1   0   0   1   1
```

```
      0.1049394101    1.0000000000
     2   0   0   1   1
      0.3697770525    1.0000000000
     3   0   0   1   1
      1.8020051942    1.0000000000
     4   0   0   1   1
      6.9065842203    1.0000000000
     5   0   0   1   1
     33.1808900405    1.0000000000
     6   0   0   1   1
    136.4209046167    1.0000000000
     7   1   1   1   1
      0.1607319451    1.0000000000
     8   1   1   1   1
      0.5766075538    1.0000000000
     9   1   1   1   1
      3.1378317419    1.0000000000
    10   1   1   1   1
      7.7737029546    1.0000000000
    11   2   2   1   1
      0.1927367938    1.0000000000
    12   2   2   1   1
      0.6271928592    1.0000000000
    13   2   2   1   1
      3.4786445912    1.0000000000
    14   2   2   1   1
     13.4453629175    1.0000000000
    15   3   3   1   1
      0.2256699148    1.0000000000
    16   3   3   1   1
      4.6711872048    1.0000000000

# RI basis set for Mg (all-electron) relative DI metric: 5.3e-04
Mg  RI_aug-TZVP-MOLOPT-ae_N_RI_059_s_p_d_f_g_h_i_7_6_4_2_0_0_0_error_5.3e-04
   19
     1   0   0   1   1
      0.1008712609    1.0000000000
     2   0   0   1   1
      0.2616218517    1.0000000000
     3   0   0   1   1
      0.9234600155    1.0000000000
     4   0   0   1   1
      2.6004089264    1.0000000000
     5   0   0   1   1
      9.1431015549    1.0000000000
     6   0   0   1   1
     38.9230934289    1.0000000000
     7   0   0   1   1
    169.2347259835    1.0000000000
     8   1   1   1   1
      0.1456856908    1.0000000000
     9   1   1   1   1
      0.2590353369    1.0000000000
    10   1   1   1   1
      0.8235460980    1.0000000000
    11   1   1   1   1
      3.1254892851    1.0000000000
    12   1   1   1   1
      9.3105248797    1.0000000000
    13   1   1   1   1
     37.7415552162    1.0000000000
    14   2   2   1   1
      0.1893556836    1.0000000000
    15   2   2   1   1
      0.4984471141    1.0000000000
    16   2   2   1   1
      3.8521323155    1.0000000000
    17   2   2   1   1
     13.0285006636    1.0000000000
    18   3   3   1   1
      0.1862912535    1.0000000000
    19   3   3   1   1
      4.9147198489    1.0000000000

# RI basis set for Mg (all-electron) relative DI metric: 2.0e-04
Mg  RI_aug-TZVP-MOLOPT-ae_N_RI_066_s_p_d_f_g_h_i_7_6_4_3_0_0_0_error_2.0e-04
   20
     1   0   0   1   1
      0.0924411082    1.0000000000
     2   0   0   1   1
      0.3019863237    1.0000000000
     3   0   0   1   1
      1.0517562816    1.0000000000
     4   0   0   1   1
      2.2986925386    1.0000000000
     5   0   0   1   1
      6.8688442140    1.0000000000
     6   0   0   1   1
     31.9630629038    1.0000000000
     7   0   0   1   1
    135.4299514969    1.0000000000
     8   1   1   1   1
      0.1437338299    1.0000000000
     9   1   1   1   1
      0.2348304171    1.0000000000
    10   1   1   1   1
      0.7553032479    1.0000000000
    11   1   1   1   1
      3.3180086275    1.0000000000
    12   1   1   1   1
      8.4047930569    1.0000000000
    13   1   1   1   1
     33.4834208541    1.0000000000
    14   2   2   1   1
      0.1944828850    1.0000000000
    15   2   2   1   1
      0.5742485726    1.0000000000
    16   2   2   1   1
      3.9301985996    1.0000000000
    17   2   2   1   1
     14.4974381540    1.0000000000
```

```
    18   3   3   1   1
      0.1664793604    1.0000000000
    19   3   3   1   1
      1.2700364129    1.0000000000
    20   3   3   1   1
      5.1945360810    1.0000000000

# RI basis set for Mg (all-electron) relative DI metric: 9.0e-05
Mg  RI_aug-TZVP-MOLOPT-ae_N_RI_071_s_p_d_f_g_h_i_7_6_5_3_0_0_0_error_9.0e-05
   21
     1   0   0   1   1
      0.0950484821    1.0000000000
     2   0   0   1   1
      0.3157392173    1.0000000000
     3   0   0   1   1
      1.0589223461    1.0000000000
     4   0   0   1   1
      2.2569676311    1.0000000000
     5   0   0   1   1
      6.5045804595    1.0000000000
     6   0   0   1   1
     29.7639527205    1.0000000000
     7   0   0   1   1
    122.0503179560    1.0000000000
     8   1   1   1   1
      0.1449412227    1.0000000000
     9   1   1   1   1
      0.2307484695    1.0000000000
    10   1   1   1   1
      0.7629427145    1.0000000000
    11   1   1   1   1
      3.3374827761    1.0000000000
    12   1   1   1   1
      8.3701172600    1.0000000000
    13   1   1   1   1
     32.7062070210    1.0000000000
    14   2   2   1   1
      0.1659327766    1.0000000000
    15   2   2   1   1
      0.2193811957    1.0000000000
    16   2   2   1   1
      0.6757946683    1.0000000000
    17   2   2   1   1
      3.7789512949    1.0000000000
    18   2   2   1   1
     15.4577080233    1.0000000000
    19   3   3   1   1
      0.1661257849    1.0000000000
    20   3   3   1   1
      1.3158907160    1.0000000000
    21   3   3   1   1
      5.8040188033    1.0000000000

# RI basis set for Mg (all-electron) relative DI metric: 2.6e-05
Mg  RI_aug-TZVP-MOLOPT-ae_N_RI_090_s_p_d_f_g_h_i_7_6_6_5_0_0_0_error_2.6e-05
   24
     1   0   0   1   1
      0.0910873725    1.0000000000
     2   0   0   1   1
      0.2059114342    1.0000000000
     3   0   0   1   1
      0.5882445179    1.0000000000
     4   0   0   1   1
      2.2281449146    1.0000000000
     5   0   0   1   1
      6.5241541795    1.0000000000
     6   0   0   1   1
     20.2128503623    1.0000000000
     7   0   0   1   1
     86.8546542699    1.0000000000
     8   1   1   1   1
      0.1322023321    1.0000000000
     9   1   1   1   1
      0.2948928829    1.0000000000
    10   1   1   1   1
      0.9346882293    1.0000000000
    11   1   1   1   1
      2.9717978859    1.0000000000
    12   1   1   1   1
      8.8661705071    1.0000000000
    13   1   1   1   1
     30.2675321255    1.0000000000
    14   2   2   1   1
      0.0944317472    1.0000000000
    15   2   2   1   1
      0.2144536826    1.0000000000
    16   2   2   1   1
      0.6316990879    1.0000000000
    17   2   2   1   1
      2.4115603333    1.0000000000
    18   2   2   1   1
      3.7617507575    1.0000000000
    19   2   2   1   1
     17.9916478363    1.0000000000
    20   3   3   1   1
      0.1174856034    1.0000000000
    21   3   3   1   1
      0.2491566148    1.0000000000
    22   3   3   1   1
      0.7326217199    1.0000000000
    23   3   3   1   1
      1.6605571684    1.0000000000
    24   3   3   1   1
      5.5136554193    1.0000000000

# RI basis set for Al (all-electron) relative DI metric: 3.5e-07
Al  RI_aug-SZV-MOLOPT-ae-mini_N_RI_009_s_p_d_f_g_h_i_3_2_0_0_0_0_0_error_3.5e-07
    5
     1   0   0   1   1
      0.1221492443    1.0000000000
     2   0   0   1   1
```

```
      0.3830916447    1.0000000000
     3   0   0   1   1
      1.8151792082    1.0000000000
     4   1   1   1   1
      0.1891407366    1.0000000000
     5   1   1   1   1
      0.3908787346    1.0000000000

# RI basis set for Al (all-electron) relative DI metric: 2.0e-09
Al  RI_aug-SZV-MOLOPT-ae-mini_N_RI_021_s_p_d_f_g_h_i_6_5_0_0_0_0_0_error_2.0e-09
   11
     1   0   0   1   1
      0.1500000000    1.0000000000
     2   0   0   1   1
      0.2595287582    1.0000000000
     3   0   0   1   1
      0.4490345088    1.0000000000
     4   0   0   1   1
      0.7769157897    1.0000000000
     5   0   0   1   1
      1.3442132673    1.0000000000
     6   0   0   1   1
      2.3257466667    1.0000000000
     7   1   1   1   1
      0.1500000000    1.0000000000
     8   1   1   1   1
      0.2976521652    1.0000000000
     9   1   1   1   1
      0.5906454097    1.0000000000
    10   1   1   1   1
      1.1720459004    1.0000000000
    11   1   1   1   1
      2.3257466667    1.0000000000

# RI basis set for Al (all-electron) relative DI metric: 5.0e-11
Al  RI_aug-SZV-MOLOPT-ae-mini_N_RI_025_s_p_d_f_g_h_i_7_6_0_0_0_0_0_error_5.0e-11
   13
     1   0   0   1   1
      0.1500000000    1.0000000000
     2   0   0   1   1
      0.2368662132    1.0000000000
     3   0   0   1   1
      0.3740373532    1.0000000000
     4   0   0   1   1
      0.5906454097    1.0000000000
     5   0   0   1   1
      0.9326929438    1.0000000000
     6   0   0   1   1
      1.4728229714    1.0000000000
     7   0   0   1   1
      2.3257466667    1.0000000000
     8   1   1   1   1
      0.1500000000    1.0000000000
     9   1   1   1   1
      0.2595287582    1.0000000000
    10   1   1   1   1
      0.4490345088    1.0000000000
    11   1   1   1   1
      0.7769157897    1.0000000000
    12   1   1   1   1
      1.3442132673    1.0000000000
    13   1   1   1   1
      2.3257466667    1.0000000000

# RI basis set for Al (all-electron) relative DI metric: 1.8e-02
Al  RI_aug-SZV-MOLOPT-ae-SR_N_RI_019_s_p_d_f_g_h_i_5_3_1_0_0_0_0_error_1.8e-02
   9
     1   0   0   1   1
      0.2354377070    1.0000000000
     2   0   0   1   1
      0.6702615016    1.0000000000
     3   0   0   1   1
      4.3280076412    1.0000000000
     4   0   0   1   1
     30.2388576387    1.0000000000
     5   0   0   1   1
    207.3210767741    1.0000000000
     6   1   1   1   1
      0.2065353692    1.0000000000
     7   1   1   1   1
      2.7339008535    1.0000000000
     8   1   1   1   1
     38.5567197290    1.0000000000
     9   2   2   1   1
      0.3187332304    1.0000000000

# RI basis set for Al (all-electron) relative DI metric: 3.2e-03
Al  RI_aug-SZV-MOLOPT-ae-SR_N_RI_030_s_p_d_f_g_h_i_6_4_1_1_0_0_0_error_3.2e-03
   12
     1   0   0   1   1
      0.1720518340    1.0000000000
     2   0   0   1   1
      0.4591546019    1.0000000000
     3   0   0   1   1
      2.0568073867    1.0000000000
     4   0   0   1   1
      9.5872325965    1.0000000000
     5   0   0   1   1
     44.6477527392    1.0000000000
     6   0   0   1   1
    207.5070013708    1.0000000000
     7   1   1   1   1
      0.2028190370    1.0000000000
     8   1   1   1   1
      1.1159010943    1.0000000000
     9   1   1   1   1
      8.3481944208    1.0000000000
    10   1   1   1   1
     52.8549801739    1.0000000000
    11   2   2   1   1
      0.3631853836    1.0000000000
```

```
    12   3   3   1   1
      0.7818307706    1.0000000000

# RI basis set for Al (all-electron) relative DI metric: 2.3e-04
Al  RI_aug-SZV-MOLOPT-ae-SR_N_RI_031_s_p_d_f_g_h_i_7_4_1_1_0_0_0_error_2.3e-04
   13
     1   0   0   1   1
      0.1555244457    1.0000000000
     2   0   0   1   1
      0.3585786588    1.0000000000
     3   0   0   1   1
      1.2384859781    1.0000000000
     4   0   0   1   1
      4.4497291466    1.0000000000
     5   0   0   1   1
     16.0368740334    1.0000000000
     6   0   0   1   1
     57.7054709464    1.0000000000
     7   0   0   1   1
    207.5330554143    1.0000000000
     8   1   1   1   1
      0.1888730932    1.0000000000
     9   1   1   1   1
      0.5899838420    1.0000000000
    10   1   1   1   1
      8.2479600572    1.0000000000
    11   1   1   1   1
     68.5432475405    1.0000000000
    12   2   2   1   1
      0.3096171362    1.0000000000
    13   3   3   1   1
      0.7011450216    1.0000000000

# RI basis set for Al (all-electron) relative DI metric: 1.4e-05
Al  RI_aug-SZV-MOLOPT-ae-SR_N_RI_044_s_p_d_f_g_h_i_7_5_3_1_0_0_0_error_1.4e-05
   16
     1   0   0   1   1
      0.1521404407    1.0000000000
     2   0   0   1   1
      0.4415583501    1.0000000000
     3   0   0   1   1
      1.6203906010    1.0000000000
     4   0   0   1   1
      6.1644090841    1.0000000000
     5   0   0   1   1
     20.3815476330    1.0000000000
     6   0   0   1   1
     65.1540777830    1.0000000000
     7   0   0   1   1
    206.6569607810    1.0000000000
     8   1   1   1   1
      0.1818964967    1.0000000000
     9   1   1   1   1
      0.4675517310    1.0000000000
    10   1   1   1   1
      2.4228615807    1.0000000000
    11   1   1   1   1
     12.6221916724    1.0000000000
    12   1   1   1   1
     59.4315110768    1.0000000000
    13   2   2   1   1
      0.2808847185    1.0000000000
    14   2   2   1   1
      1.0794659306    1.0000000000
    15   2   2   1   1
     14.2103902629    1.0000000000
    16   3   3   1   1
      0.5292616794    1.0000000000

# RI basis set for Al (all-electron) relative DI metric: 3.2e-06
Al  RI_aug-SZV-MOLOPT-ae-SR_N_RI_049_s_p_d_f_g_h_i_7_5_4_1_0_0_0_error_3.2e-06
   17
     1   0   0   1   1
      0.1804115198    1.0000000000
     2   0   0   1   1
      0.4545458601    1.0000000000
     3   0   0   1   1
      1.4866027893    1.0000000000
     4   0   0   1   1
      6.0159611358    1.0000000000
     5   0   0   1   1
     20.2758710488    1.0000000000
     6   0   0   1   1
     65.0589087391    1.0000000000
     7   0   0   1   1
    206.6247843837    1.0000000000
     8   1   1   1   1
      0.2416293264    1.0000000000
     9   1   1   1   1
      0.3802763631    1.0000000000
    10   1   1   1   1
      2.2345412377    1.0000000000
    11   1   1   1   1
     11.9745628307    1.0000000000
    12   1   1   1   1
     60.4376412542    1.0000000000
    13   2   2   1   1
      0.2723545661    1.0000000000
    14   2   2   1   1
      0.9893373874    1.0000000000
    15   2   2   1   1
      3.9677705912    1.0000000000
    16   2   2   1   1
     14.2293304533    1.0000000000
    17   3   3   1   1
      0.3670211484    1.0000000000

# RI basis set for Al (all-electron) relative DI metric: 5.9e-07
Al  RI_aug-SZV-MOLOPT-ae-SR_N_RI_056_s_p_d_f_g_h_i_7_5_4_2_0_0_0_error_5.9e-07
   18
     1   0   0   1   1
```

```
      0.2032650008    1.0000000000
     2   0   0   1   1
      0.3941295881    1.0000000000
     3   0   0   1   1
      1.2136086846    1.0000000000
     4   0   0   1   1
      4.3605351856    1.0000000000
     5   0   0   1   1
     15.9336201008    1.0000000000
     6   0   0   1   1
     57.5965689536    1.0000000000
     7   0   0   1   1
    207.4169307033    1.0000000000
     8   1   1   1   1
      0.2090775754    1.0000000000
     9   1   1   1   1
      0.4222981189    1.0000000000
    10   1   1   1   1
      2.3670698446    1.0000000000
    11   1   1   1   1
     12.0823231554    1.0000000000
    12   1   1   1   1
     60.5245554030    1.0000000000
    13   2   2   1   1
      0.2519879090    1.0000000000
    14   2   2   1   1
      0.5177509558    1.0000000000
    15   2   2   1   1
      2.8897992713    1.0000000000
    16   2   2   1   1
     14.3254031724    1.0000000000
    17   3   3   1   1
      0.3182662517    1.0000000000
    18   3   3   1   1
      3.0152123180    1.0000000000

# RI basis set for Al (all-electron) relative DI metric: 2.4e-07
Al  RI_aug-SZV-MOLOPT-ae-SR_N_RI_103_s_p_d_f_g_h_i_7_5_5_2_2_1_1_error_2.4e-07
   23
     1   0   0   1   1
      0.1758134011    1.0000000000
     2   0   0   1   1
      0.3647349325    1.0000000000
     3   0   0   1   1
      1.2322719535    1.0000000000
     4   0   0   1   1
      4.4241731142    1.0000000000
     5   0   0   1   1
     16.0061134692    1.0000000000
     6   0   0   1   1
     57.6740514680    1.0000000000
     7   0   0   1   1
    207.4998394453    1.0000000000
     8   1   1   1   1
      0.2016433208    1.0000000000
     9   1   1   1   1
      0.4150270060    1.0000000000
    10   1   1   1   1
      2.3724636041    1.0000000000
    11   1   1   1   1
     12.0985399123    1.0000000000
    12   1   1   1   1
     60.5394264052    1.0000000000
    13   2   2   1   1
      0.2261776318    1.0000000000
    14   2   2   1   1
      0.3572058661    1.0000000000
    15   2   2   1   1
      1.3083693960    1.0000000000
    16   2   2   1   1
      4.3391784218    1.0000000000
    17   2   2   1   1
     14.3357558397    1.0000000000
    18   3   3   1   1
      0.2813743345    1.0000000000
    19   3   3   1   1
      2.9945209450    1.0000000000
    20   4   4   1   1
      0.1599539537    1.0000000000
    21   4   4   1   1
      1.0739288668    1.0000000000
    22   5   5   1   1
      0.5901435493    1.0000000000
    23   6   6   1   1
      0.5835397040    1.0000000000

# RI basis set for Al (all-electron) relative DI metric: 8.9e-04
Al  RI_aug-SZV-MOLOPT-ae_N_RI_027_s_p_d_f_g_h_i_6_3_1_1_0_0_0_error_8.9e-04
   11
     1   0   0   1   1
      0.2067172443    1.0000000000
     2   0   0   1   1
      0.4520040703    1.0000000000
     3   0   0   1   1
      1.9827618281    1.0000000000
     4   0   0   1   1
      9.5266136040    1.0000000000
     5   0   0   1   1
     44.6197145485    1.0000000000
     6   0   0   1   1
    207.4811068801    1.0000000000
     7   1   1   1   1
      0.1491943392    1.0000000000
     8   1   1   1   1
      0.2804925881    1.0000000000
     9   1   1   1   1
      8.3491768740    1.0000000000
    10   2   2   1   1
      0.3417349607    1.0000000000
    11   3   3   1   1
      1.3863267342    1.0000000000
```

```
# RI basis set for Al (all-electron) relative DI metric: 7.3e-05
Al  RI_aug-SZV-MOLOPT-ae_N_RI_039_s_p_d_f_g_h_i_6_4_1_1_1_0_0_error_7.3e-05
   13
    1   0   0   1   1
      0.1613373956    1.0000000000
    2   0   0   1   1
      0.4554362209    1.0000000000
    3   0   0   1   1
      2.0827781878    1.0000000000
    4   0   0   1   1
      9.6302478776    1.0000000000
    5   0   0   1   1
     44.6965269772    1.0000000000
    6   0   0   1   1
    207.5610354002    1.0000000000
    7   1   1   1   1
      0.2009954304    1.0000000000
    8   1   1   1   1
      0.3748572902    1.0000000000
    9   1   1   1   1
      6.2553122689    1.0000000000
   10   1   1   1   1
     53.5402695283    1.0000000000
   11   2   2   1   1
      0.3636266559    1.0000000000
   12   3   3   1   1
      0.7426753919    1.0000000000
   13   4   4   1   1
      0.1595857001    1.0000000000

# RI basis set for Al (all-electron) relative DI metric: 2.2e-05
Al  RI_aug-SZV-MOLOPT-ae_N_RI_048_s_p_d_f_g_h_i_7_5_2_1_1_0_0_error_2.2e-05
   16
    1   0   0   1   1
      0.1354684428    1.0000000000
    2   0   0   1   1
      0.3369389848    1.0000000000
    3   0   0   1   1
      1.1970775210    1.0000000000
    4   0   0   1   1
      4.3781437167    1.0000000000
    5   0   0   1   1
     15.9416275263    1.0000000000
    6   0   0   1   1
     57.6047755554    1.0000000000
    7   0   0   1   1
    207.4265569339    1.0000000000
    8   1   1   1   1
      0.1445237399    1.0000000000
    9   1   1   1   1
      0.4630589022    1.0000000000
   10   1   1   1   1
      1.8892298644    1.0000000000
   11   1   1   1   1
     11.7544456007    1.0000000000
   12   1   1   1   1
     60.4182055599    1.0000000000
   13   2   2   1   1
      0.3266844876    1.0000000000
   14   2   2   1   1
     12.0084519592    1.0000000000
   15   3   3   1   1
      0.6088857260    1.0000000000
   16   4   4   1   1
      1.1215509910    1.0000000000

# RI basis set for Al (all-electron) relative DI metric: 1.0e-05
Al  RI_aug-SZV-MOLOPT-ae_N_RI_053_s_p_d_f_g_h_i_7_5_3_1_1_0_0_error_1.0e-05
   17
    1   0   0   1   1
      0.1258978784    1.0000000000
    2   0   0   1   1
      0.3353967231    1.0000000000
    3   0   0   1   1
      1.2012338661    1.0000000000
    4   0   0   1   1
      4.4021199682    1.0000000000
    5   0   0   1   1
     15.9788203809    1.0000000000
    6   0   0   1   1
     57.6434408248    1.0000000000
    7   0   0   1   1
    207.4672992066    1.0000000000
    8   1   1   1   1
      0.1516158471    1.0000000000
    9   1   1   1   1
      0.4491888332    1.0000000000
   10   1   1   1   1
      2.1712602597    1.0000000000
   11   1   1   1   1
     11.9594453014    1.0000000000
   12   1   1   1   1
     60.4801331394    1.0000000000
   13   2   2   1   1
      0.3236660642    1.0000000000
   14   2   2   1   1
      1.8110650057    1.0000000000
   15   2   2   1   1
     13.3945970021    1.0000000000
   16   3   3   1   1
      0.5221554384    1.0000000000
   17   4   4   1   1
      0.6472513927    1.0000000000

# RI basis set for Al (all-electron) relative DI metric: 6.1e-07
Al  RI_aug-SZV-MOLOPT-ae_N_RI_058_s_p_d_f_g_h_i_7_5_4_1_1_0_0_error_6.1e-07
   18
    1   0   0   1   1
      0.1251650853    1.0000000000
    2   0   0   1   1
```

```
      0.3427276409    1.0000000000
     3   0   0   1   1
      1.2186344428    1.0000000000
     4   0   0   1   1
      4.4275058677    1.0000000000
     5   0   0   1   1
     16.0095335344    1.0000000000
     6   0   0   1   1
     57.6763204233    1.0000000000
     7   0   0   1   1
    207.5022466723    1.0000000000
     8   1   1   1   1
      0.1413371374    1.0000000000
     9   1   1   1   1
      0.4337492136    1.0000000000
    10   1   1   1   1
      2.0873062168    1.0000000000
    11   1   1   1   1
     11.8811300201    1.0000000000
    12   1   1   1   1
     60.4320566075    1.0000000000
    13   2   2   1   1
      0.2105841760    1.0000000000
    14   2   2   1   1
      0.4847770774    1.0000000000
    15   2   2   1   1
      2.8669374326    1.0000000000
    16   2   2   1   1
     14.3348035017    1.0000000000
    17   3   3   1   1
      0.3694067709    1.0000000000
    18   4   4   1   1
      0.6467670730    1.0000000000

# RI basis set for Al (all-electron) relative DI metric: 1.9e-07
Al  RI_aug-SZV-MOLOPT-ae_N_RI_095_s_p_d_f_g_h_i_7_5_5_4_1_1_0_error_1.9e-07
   23
     1   0   0   1   1
      0.1200523045    1.0000000000
     2   0   0   1   1
      0.3495372299    1.0000000000
     3   0   0   1   1
      1.2334951165    1.0000000000
     4   0   0   1   1
      4.4271489949    1.0000000000
     5   0   0   1   1
     15.9965391709    1.0000000000
     6   0   0   1   1
     57.6634065177    1.0000000000
     7   0   0   1   1
    207.4895034330    1.0000000000
     8   1   1   1   1
      0.1383983567    1.0000000000
     9   1   1   1   1
      0.4163670520    1.0000000000
    10   1   1   1   1
      2.2044535242    1.0000000000
    11   1   1   1   1
     11.8925780155    1.0000000000
    12   1   1   1   1
     60.4210520743    1.0000000000
    13   2   2   1   1
      0.1232504214    1.0000000000
    14   2   2   1   1
      0.3707000221    1.0000000000
    15   2   2   1   1
      1.2979796111    1.0000000000
    16   2   2   1   1
      4.3228464572    1.0000000000
    17   2   2   1   1
     14.3227472321    1.0000000000
    18   3   3   1   1
      0.2143479080    1.0000000000
    19   3   3   1   1
      0.4259150937    1.0000000000
    20   3   3   1   1
      1.1696812916    1.0000000000
    21   3   3   1   1
      3.0352430407    1.0000000000
    22   4   4   1   1
      0.6506989097    1.0000000000
    23   5   5   1   1
      0.5968196068    1.0000000000

# RI basis set for Al (all-electron) relative DI metric: 2.9e-02
Al  RI_aug-DZVP-MOLOPT-ae-SR_N_RI_023_s_p_d_f_g_h_i_6_4_1_0_0_0_0_error_2.9e-02
   11
     1   0   0   1   1
      0.1746910009    1.0000000000
     2   0   0   1   1
      0.3900658588    1.0000000000
     3   0   0   1   1
      1.7454687308    1.0000000000
     4   0   0   1   1
      9.0535801312    1.0000000000
     5   0   0   1   1
     44.0347083769    1.0000000000
     6   0   0   1   1
    206.8332063309    1.0000000000
     7   1   1   1   1
      0.2715571718    1.0000000000
     8   1   1   1   1
      1.5254479792    1.0000000000
     9   1   1   1   1
      8.9489539514    1.0000000000
    10   1   1   1   1
     48.5233360681    1.0000000000
    11   2   2   1   1
      0.4467425568    1.0000000000

# RI basis set for Al (all-electron) relative DI metric: 1.4e-02
```

```
Al  RI_aug-DZVP-MOLOPT-ae-SR_N_RI_045_s_p_d_f_g_h_i_6_4_4_1_0_0_0_error_1.4e-02
   15
    1   0   0   1   1
      0.1419489359    1.0000000000
    2   0   0   1   1
      0.5133638430    1.0000000000
    3   0   0   1   1
      2.5528037983    1.0000000000
    4   0   0   1   1
     12.7846860873    1.0000000000
    5   0   0   1   1
     58.1060952235    1.0000000000
    6   0   0   1   1
    255.8858902352    1.0000000000
    7   1   1   1   1
      0.3810469475    1.0000000000
    8   1   1   1   1
      2.1575979502    1.0000000000
    9   1   1   1   1
     12.2174151206    1.0000000000
   10   1   1   1   1
     69.1770967747    1.0000000000
   11   2   2   1   1
      0.1698458967    1.0000000000
   12   2   2   1   1
      0.6182586179    1.0000000000
   13   2   2   1   1
      2.7640245922    1.0000000000
   14   2   2   1   1
     11.8299755771    1.0000000000
   15   3   3   1   1
      0.1250839652    1.0000000000

# RI basis set for Al (all-electron) relative DI metric: 3.6e-04
Al  RI_aug-DZVP-MOLOPT-ae-SR_N_RI_049_s_p_d_f_g_h_i_7_5_4_1_0_0_0_error_3.6e-04
   17
    1   0   0   1   1
      0.1516615421    1.0000000000
    2   0   0   1   1
      0.3650036450    1.0000000000
    3   0   0   1   1
      1.2531823002    1.0000000000
    4   0   0   1   1
      4.4601469176    1.0000000000
    5   0   0   1   1
     16.0524042880    1.0000000000
    6   0   0   1   1
     57.7258420993    1.0000000000
    7   0   0   1   1
    207.5552924362    1.0000000000
    8   1   1   1   1
      0.1409394607    1.0000000000
    9   1   1   1   1
      0.3313781379    1.0000000000
   10   1   1   1   1
      2.0970357559    1.0000000000
   11   1   1   1   1
     10.8986881885    1.0000000000
   12   1   1   1   1
     48.8299414558    1.0000000000
   13   2   2   1   1
      0.1417007428    1.0000000000
   14   2   2   1   1
      0.4376792950    1.0000000000
   15   2   2   1   1
      2.7175527856    1.0000000000
   16   2   2   1   1
     14.1981333628    1.0000000000
   17   3   3   1   1
      0.2124515418    1.0000000000

# RI basis set for Al (all-electron) relative DI metric: 3.6e-05
Al  RI_aug-DZVP-MOLOPT-ae-SR_N_RI_061_s_p_d_f_g_h_i_7_6_4_1_1_0_0_error_3.6e-05
   19
    1   0   0   1   1
      0.1216894413    1.0000000000
    2   0   0   1   1
      0.3190961912    1.0000000000
    3   0   0   1   1
      1.0422646709    1.0000000000
    4   0   0   1   1
      4.0425181488    1.0000000000
    5   0   0   1   1
     15.5665162838    1.0000000000
    6   0   0   1   1
     57.2071058653    1.0000000000
    7   0   0   1   1
    207.0011037160    1.0000000000
    8   1   1   1   1
      0.1696366126    1.0000000000
    9   1   1   1   1
      0.3759102268    1.0000000000
   10   1   1   1   1
      1.0946565208    1.0000000000
   11   1   1   1   1
      4.2780209975    1.0000000000
   12   1   1   1   1
     16.3634756695    1.0000000000
   13   1   1   1   1
     60.2218011393    1.0000000000
   14   2   2   1   1
      0.1682886668    1.0000000000
   15   2   2   1   1
      0.4327167241    1.0000000000
   16   2   2   1   1
      1.9693959626    1.0000000000
   17   2   2   1   1
      9.7389882627    1.0000000000
   18   3   3   1   1
      0.1898088006    1.0000000000
   19   4   4   1   1
```

```
      0.7918944001    1.0000000000

# RI basis set for Al (all-electron) relative DI metric: 1.3e-05
Al  RI_aug-DZVP-MOLOPT-ae-SR_N_RI_082_s_p_d_f_g_h_i_7_6_5_2_2_0_0_error_1.3e-05
   22
     1   0   0   1   1
      0.1735791779    1.0000000000
     2   0   0   1   1
      0.2918912396    1.0000000000
     3   0   0   1   1
      0.9044668717    1.0000000000
     4   0   0   1   1
      3.6189891663    1.0000000000
     5   0   0   1   1
     14.9712913211    1.0000000000
     6   0   0   1   1
     56.5608323280    1.0000000000
     7   0   0   1   1
    206.3084577042    1.0000000000
     8   1   1   1   1
      0.1563921638    1.0000000000
     9   1   1   1   1
      0.4328423232    1.0000000000
    10   1   1   1   1
      0.9010722342    1.0000000000
    11   1   1   1   1
      3.5823013629    1.0000000000
    12   1   1   1   1
     15.3943574994    1.0000000000
    13   1   1   1   1
     59.2820251265    1.0000000000
    14   2   2   1   1
      0.1706543980    1.0000000000
    15   2   2   1   1
      0.4491207819    1.0000000000
    16   2   2   1   1
      1.2264744276    1.0000000000
    17   2   2   1   1
      4.2643415572    1.0000000000
    18   2   2   1   1
     14.2749270029    1.0000000000
    19   3   3   1   1
      0.1892059641    1.0000000000
    20   3   3   1   1
      2.1285327629    1.0000000000
    21   4   4   1   1
      0.5801088489    1.0000000000
    22   4   4   1   1
      1.1799200029    1.0000000000

# RI basis set for Al (all-electron) relative DI metric: 5.8e-06
Al  RI_aug-DZVP-MOLOPT-ae-SR_N_RI_100_s_p_d_f_g_h_i_7_6_5_3_2_1_0_error_5.8e-06
   24
     1   0   0   1   1
      0.1345155601    1.0000000000
     2   0   0   1   1
      0.3015925901    1.0000000000
     3   0   0   1   1
      0.9683844009    1.0000000000
     4   0   0   1   1
      3.8376132110    1.0000000000
     5   0   0   1   1
     15.3084322184    1.0000000000
     6   0   0   1   1
     56.9364216837    1.0000000000
     7   0   0   1   1
    206.7129154929    1.0000000000
     8   1   1   1   1
      0.1492304576    1.0000000000
     9   1   1   1   1
      0.3998954403    1.0000000000
    10   1   1   1   1
      1.0703823083    1.0000000000
    11   1   1   1   1
      4.1523206394    1.0000000000
    12   1   1   1   1
     16.1861109650    1.0000000000
    13   1   1   1   1
     60.0460364583    1.0000000000
    14   2   2   1   1
      0.1606100939    1.0000000000
    15   2   2   1   1
      0.4299145884    1.0000000000
    16   2   2   1   1
      1.2803867090    1.0000000000
    17   2   2   1   1
      4.3117275893    1.0000000000
    18   2   2   1   1
     14.3148775297    1.0000000000
    19   3   3   1   1
      0.1837883436    1.0000000000
    20   3   3   1   1
      0.7097293671    1.0000000000
    21   3   3   1   1
      3.0010296871    1.0000000000
    22   4   4   1   1
      0.2989001234    1.0000000000
    23   4   4   1   1
      1.5225873966    1.0000000000
    24   5   5   1   1
      0.5655098133    1.0000000000

# RI basis set for Al (all-electron) relative DI metric: 2.8e-06
Al  RI_aug-DZVP-MOLOPT-ae-SR_N_RI_111_s_p_d_f_g_h_i_7_6_5_3_2_2_0_error_2.8e-06
   25
     1   0   0   1   1
      0.1615768296    1.0000000000
     2   0   0   1   1
      0.2769309759    1.0000000000
     3   0   0   1   1
      0.9132210260    1.0000000000
```

```
    4   0   0   1   1
     3.6440605468    1.0000000000
    5   0   0   1   1
    15.0190039575    1.0000000000
    6   0   0   1   1
    56.6260505900    1.0000000000
    7   0   0   1   1
   206.3818651031    1.0000000000
    8   1   1   1   1
     0.1445022569    1.0000000000
    9   1   1   1   1
     0.3840193643    1.0000000000
   10   1   1   1   1
     1.0074717499    1.0000000000
   11   1   1   1   1
     3.9544767180    1.0000000000
   12   1   1   1   1
    15.9374812163    1.0000000000
   13   1   1   1   1
    59.8077477746    1.0000000000
   14   2   2   1   1
     0.1615384614    1.0000000000
   15   2   2   1   1
     0.4339744315    1.0000000000
   16   2   2   1   1
     1.2706604384    1.0000000000
   17   2   2   1   1
     4.3033579846    1.0000000000
   18   2   2   1   1
    14.3087228299    1.0000000000
   19   3   3   1   1
     0.1756745155    1.0000000000
   20   3   3   1   1
     0.6903218771    1.0000000000
   21   3   3   1   1
     3.0091975583    1.0000000000
   22   4   4   1   1
     0.2464186778    1.0000000000
   23   4   4   1   1
     1.1660823083    1.0000000000
   24   5   5   1   1
     0.4082664324    1.0000000000
   25   5   5   1   1
     0.6324307990    1.0000000000

# RI basis set for Al (all-electron) relative DI metric: 3.6e-02
Al  RI_aug-DZVP-MOLOPT-ae_N_RI_019_s_p_d_f_g_h_i_4_1_1_1_0_0_0_error_3.6e-02
  7
    1   0   0   1   1
     0.8475010084    1.0000000000
    2   0   0   1   1
     5.5752436714    1.0000000000
    3   0   0   1   1
    33.9490040805    1.0000000000
    4   0   0   1   1
   190.4521119670    1.0000000000
    5   1   1   1   1
     0.2471682915    1.0000000000
    6   2   2   1   1
     0.3759042603    1.0000000000
    7   3   3   1   1
     1.3042395869    1.0000000000

# RI basis set for Al (all-electron) relative DI metric: 1.4e-02
Al  RI_aug-DZVP-MOLOPT-ae_N_RI_030_s_p_d_f_g_h_i_6_4_1_1_0_0_0_error_1.4e-02
  12
    1   0   0   1   1
     0.1929692757    1.0000000000
    2   0   0   1   1
     0.4163925401    1.0000000000
    3   0   0   1   1
     1.7557810321    1.0000000000
    4   0   0   1   1
     9.0386746781    1.0000000000
    5   0   0   1   1
    44.0157338146    1.0000000000
    6   0   0   1   1
   206.8125929026    1.0000000000
    7   1   1   1   1
     0.1376178970    1.0000000000
    8   1   1   1   1
     0.3850881721    1.0000000000
    9   1   1   1   1
     4.6126043221    1.0000000000
   10   1   1   1   1
    43.2144591487    1.0000000000
   11   2   2   1   1
     0.3686849906    1.0000000000
   12   3   3   1   1
     0.5952661157    1.0000000000

# RI basis set for Al (all-electron) relative DI metric: 5.4e-03
Al  RI_aug-DZVP-MOLOPT-ae_N_RI_039_s_p_d_f_g_h_i_6_4_1_1_1_0_0_error_5.4e-03
  13
    1   0   0   1   1
     0.1825682714    1.0000000000
    2   0   0   1   1
     0.4947302583    1.0000000000
    3   0   0   1   1
     2.0774882473    1.0000000000
    4   0   0   1   1
     9.5430170147    1.0000000000
    5   0   0   1   1
    44.5876299883    1.0000000000
    6   0   0   1   1
   207.4407159930    1.0000000000
    7   1   1   1   1
     0.1313219111    1.0000000000
    8   1   1   1   1
     0.3453740175    1.0000000000
    9   1   1   1   1
```

```
      4.7214964430    1.0000000000
    10   1   1   1   1
     39.9277800217    1.0000000000
    11   2   2   1   1
      0.4335064728    1.0000000000
    12   3   3   1   1
      0.5354421590    1.0000000000
    13   4   4   1   1
      0.1071744059    1.0000000000

# RI basis set for Al (all-electron) relative DI metric: 4.8e-04
Al  RI_aug-DZVP-MOLOPT-ae_N_RI_053_s_p_d_f_g_h_i_7_5_3_1_1_0_0_error_4.8e-04
   17
     1   0   0   1   1
      0.1779944504    1.0000000000
     2   0   0   1   1
      0.3327316042    1.0000000000
     3   0   0   1   1
      1.0859394175    1.0000000000
     4   0   0   1   1
      4.1936321428    1.0000000000
     5   0   0   1   1
     15.7644953461    1.0000000000
     6   0   0   1   1
     57.4205182486    1.0000000000
     7   0   0   1   1
    207.2292165671    1.0000000000
     8   1   1   1   1
      0.1608058689    1.0000000000
     9   1   1   1   1
      0.3007202010    1.0000000000
    10   1   1   1   1
      2.1057529700    1.0000000000
    11   1   1   1   1
     11.2532907860    1.0000000000
    12   1   1   1   1
     51.8721874189    1.0000000000
    13   2   2   1   1
      0.1820173676    1.0000000000
    14   2   2   1   1
      0.4899885569    1.0000000000
    15   2   2   1   1
      7.2895422711    1.0000000000
    16   3   3   1   1
      0.1564622539    1.0000000000
    17   4   4   1   1
      0.5788995771    1.0000000000

# RI basis set for Al (all-electron) relative DI metric: 5.9e-05
Al  RI_aug-DZVP-MOLOPT-ae_N_RI_056_s_p_d_f_g_h_i_7_6_3_1_1_0_0_error_5.9e-05
   18
     1   0   0   1   1
      0.1239966836    1.0000000000
     2   0   0   1   1
      0.2875079033    1.0000000000
     3   0   0   1   1
      0.9861627938    1.0000000000
     4   0   0   1   1
      3.9937483983    1.0000000000
     5   0   0   1   1
     15.5501410688    1.0000000000
     6   0   0   1   1
     57.1983668199    1.0000000000
     7   0   0   1   1
    206.9934307525    1.0000000000
     8   1   1   1   1
      0.1236618443    1.0000000000
     9   1   1   1   1
      0.3136727823    1.0000000000
    10   1   1   1   1
      0.9681545850    1.0000000000
    11   1   1   1   1
      4.0566404716    1.0000000000
    12   1   1   1   1
     16.1891189841    1.0000000000
    13   1   1   1   1
     60.0725159176    1.0000000000
    14   2   2   1   1
      0.1618360260    1.0000000000
    15   2   2   1   1
      0.4839219226    1.0000000000
    16   2   2   1   1
      3.0884033261    1.0000000000
    17   3   3   1   1
      0.1661307643    1.0000000000
    18   4   4   1   1
      0.6737137230    1.0000000000

# RI basis set for Al (all-electron) relative DI metric: 2.9e-05
Al  RI_aug-DZVP-MOLOPT-ae_N_RI_091_s_p_d_f_g_h_i_7_6_5_3_1_1_0_error_2.9e-05
   23
     1   0   0   1   1
      0.1228561948    1.0000000000
     2   0   0   1   1
      0.3547980858    1.0000000000
     3   0   0   1   1
      0.8697181359    1.0000000000
     4   0   0   1   1
      3.7425804603    1.0000000000
     5   0   0   1   1
     15.3047905302    1.0000000000
     6   0   0   1   1
     56.7541238979    1.0000000000
     7   0   0   1   1
    204.3484011601    1.0000000000
     8   1   1   1   1
      0.1159258272    1.0000000000
     9   1   1   1   1
      0.2980924018    1.0000000000
    10   1   1   1   1
      0.8827308321    1.0000000000
```

```
    11   1   1   1   1
      3.7827990223    1.0000000000
    12   1   1   1   1
     15.8615229081    1.0000000000
    13   1   1   1   1
     59.7555157906    1.0000000000
    14   2   2   1   1
      0.1429118724    1.0000000000
    15   2   2   1   1
      0.4344036631    1.0000000000
    16   2   2   1   1
      1.2352454296    1.0000000000
    17   2   2   1   1
      4.2710408979    1.0000000000
    18   2   2   1   1
     14.2834774729    1.0000000000
    19   3   3   1   1
      0.1719501546    1.0000000000
    20   3   3   1   1
      0.7562169338    1.0000000000
    21   3   3   1   1
      2.8698671041    1.0000000000
    22   4   4   1   1
      0.4847766919    1.0000000000
    23   5   5   1   1
      0.4529777996    1.0000000000

# RI basis set for Al (all-electron) relative DI metric: 4.0e-02
Al  RI_aug-TZVP-MOLOPT-ae_N_RI_030_s_p_d_f_g_h_i_6_4_1_1_0_0_0_error_4.0e-02
   12
     1   0   0   1   1
      0.1759853938    1.0000000000
     2   0   0   1   1
      0.4151753323    1.0000000000
     3   0   0   1   1
      1.9094559446    1.0000000000
     4   0   0   1   1
      9.3760502881    1.0000000000
     5   0   0   1   1
     44.4035061117    1.0000000000
     6   0   0   1   1
    207.2358713011    1.0000000000
     7   1   1   1   1
      0.3005892967    1.0000000000
     8   1   1   1   1
      0.9695435351    1.0000000000
     9   1   1   1   1
      8.4713493621    1.0000000000
    10   1   1   1   1
     56.7933004516    1.0000000000
    11   2   2   1   1
      0.3152249653    1.0000000000
    12   3   3   1   1
      0.5352041202    1.0000000000

# RI basis set for Al (all-electron) relative DI metric: 1.9e-02
Al  RI_aug-TZVP-MOLOPT-ae_N_RI_043_s_p_d_f_g_h_i_7_5_1_1_1_0_0_error_1.9e-02
   15
     1   0   0   1   1
      0.1800310300    1.0000000000
     2   0   0   1   1
      0.2889794459    1.0000000000
     3   0   0   1   1
      0.9267483447    1.0000000000
     4   0   0   1   1
      3.7399131803    1.0000000000
     5   0   0   1   1
     15.1309271475    1.0000000000
     6   0   0   1   1
     56.7217684649    1.0000000000
     7   0   0   1   1
    206.4815589622    1.0000000000
     8   1   1   1   1
      0.2398031858    1.0000000000
     9   1   1   1   1
      0.5207701192    1.0000000000
    10   1   1   1   1
      2.8985430802    1.0000000000
    11   1   1   1   1
     13.8129115097    1.0000000000
    12   1   1   1   1
     56.5629935713    1.0000000000
    13   2   2   1   1
      0.4239655696    1.0000000000
    14   3   3   1   1
      0.4758273178    1.0000000000
    15   4   4   1   1
      0.2592360415    1.0000000000

# RI basis set for Al (all-electron) relative DI metric: 5.5e-03
Al  RI_aug-TZVP-MOLOPT-ae_N_RI_056_s_p_d_f_g_h_i_7_6_3_1_1_0_0_error_5.5e-03
   18
     1   0   0   1   1
      0.1460205173    1.0000000000
     2   0   0   1   1
      0.2965821351    1.0000000000
     3   0   0   1   1
      1.0184602937    1.0000000000
     4   0   0   1   1
      4.0488614894    1.0000000000
     5   0   0   1   1
     15.5663551458    1.0000000000
     6   0   0   1   1
     57.1988500055    1.0000000000
     7   0   0   1   1
    206.9916731706    1.0000000000
     8   1   1   1   1
      0.1895788248    1.0000000000
     9   1   1   1   1
      0.3434053999    1.0000000000
    10   1   1   1   1
```

```
      1.0307304275    1.0000000000
    11   1   1   1   1
      4.1257992766    1.0000000000
    12   1   1   1   1
     16.2173455112    1.0000000000
    13   1   1   1   1
     60.0981277487    1.0000000000
    14   2   2   1   1
      0.2304738903    1.0000000000
    15   2   2   1   1
      0.8343954452    1.0000000000
    16   2   2   1   1
      6.5516211020    1.0000000000
    17   3   3   1   1
      0.3608321534    1.0000000000
    18   4   4   1   1
      0.4406243897    1.0000000000

# RI basis set for Al (all-electron) relative DI metric: 1.2e-03
Al  RI_aug-TZVP-MOLOPT-ae_N_RI_070_s_p_d_f_g_h_i_7_6_3_3_1_0_0_error_1.2e-03
   20
     1   0   0   1   1
      0.1582173800    1.0000000000
     2   0   0   1   1
      0.2636924522    1.0000000000
     3   0   0   1   1
      0.9098387045    1.0000000000
     4   0   0   1   1
      3.7734399575    1.0000000000
     5   0   0   1   1
     15.2269037374    1.0000000000
     6   0   0   1   1
     56.8214487057    1.0000000000
     7   0   0   1   1
    206.5847288483    1.0000000000
     8   1   1   1   1
      0.2012424973    1.0000000000
     9   1   1   1   1
      0.3635367493    1.0000000000
    10   1   1   1   1
      0.9675291571    1.0000000000
    11   1   1   1   1
      4.0458254031    1.0000000000
    12   1   1   1   1
     16.1347904753    1.0000000000
    13   1   1   1   1
     60.0116742880    1.0000000000
    14   2   2   1   1
      0.2247281375    1.0000000000
    15   2   2   1   1
      0.4441406718    1.0000000000
    16   2   2   1   1
      1.2356901303    1.0000000000
    17   3   3   1   1
      0.2853439862    1.0000000000
    18   3   3   1   1
      0.5945264259    1.0000000000
    19   3   3   1   1
      2.7936996353    1.0000000000
    20   4   4   1   1
      0.2932748019    1.0000000000

# RI basis set for Al (all-electron) relative DI metric: 5.6e-04
Al  RI_aug-TZVP-MOLOPT-ae_N_RI_088_s_p_d_f_g_h_i_7_6_3_3_3_0_0_error_5.6e-04
   22
     1   0   0   1   1
      0.1424915876    1.0000000000
     2   0   0   1   1
      0.3261832636    1.0000000000
     3   0   0   1   1
      1.1832309448    1.0000000000
     4   0   0   1   1
      4.3694067400    1.0000000000
     5   0   0   1   1
     15.9405090753    1.0000000000
     6   0   0   1   1
     57.6008661511    1.0000000000
     7   0   0   1   1
    207.4208883088    1.0000000000
     8   1   1   1   1
      0.1907706641    1.0000000000
     9   1   1   1   1
      0.2940060252    1.0000000000
    10   1   1   1   1
      0.8805919884    1.0000000000
    11   1   1   1   1
      3.3031295093    1.0000000000
    12   1   1   1   1
     14.3699879808    1.0000000000
    13   1   1   1   1
     58.3636155772    1.0000000000
    14   2   2   1   1
      0.1815309041    1.0000000000
    15   2   2   1   1
      0.4822201858    1.0000000000
    16   2   2   1   1
      1.7728218181    1.0000000000
    17   3   3   1   1
      0.2101426840    1.0000000000
    18   3   3   1   1
      0.4800532807    1.0000000000
    19   3   3   1   1
      2.3721319851    1.0000000000
    20   4   4   1   1
      0.1672097062    1.0000000000
    21   4   4   1   1
      0.4442049020    1.0000000000
    22   4   4   1   1
      0.8720677539    1.0000000000

# RI basis set for Al (all-electron) relative DI metric: 2.1e-04
```

```
Al  RI_aug-TZVP-MOLOPT-ae_N_RI_103_s_p_d_f_g_h_i_7_6_6_3_3_0_0_error_2.1e-04
   25
    1   0   0   1   1
      0.1602495938    1.0000000000
    2   0   0   1   1
      0.3708575497    1.0000000000
    3   0   0   1   1
      1.2379795993    1.0000000000
    4   0   0   1   1
      4.3935776691    1.0000000000
    5   0   0   1   1
     15.9556941496    1.0000000000
    6   0   0   1   1
     57.6156924460    1.0000000000
    7   0   0   1   1
    207.4362638927    1.0000000000
    8   1   1   1   1
      0.1757753585    1.0000000000
    9   1   1   1   1
      0.3154970784    1.0000000000
   10   1   1   1   1
      0.9351213545    1.0000000000
   11   1   1   1   1
      3.3470320007    1.0000000000
   12   1   1   1   1
     11.5416219101    1.0000000000
   13   1   1   1   1
     36.7084306335    1.0000000000
   14   2   2   1   1
      0.1855991902    1.0000000000
   15   2   2   1   1
      0.3626555453    1.0000000000
   16   2   2   1   1
      0.8216604961    1.0000000000
   17   2   2   1   1
      2.0454856416    1.0000000000
   18   2   2   1   1
      5.4412772230    1.0000000000
   19   2   2   1   1
     14.2815698861    1.0000000000
   20   3   3   1   1
      0.1405545020    1.0000000000
   21   3   3   1   1
      0.3070644358    1.0000000000
   22   3   3   1   1
      0.6239947467    1.0000000000
   23   4   4   1   1
      0.1980738748    1.0000000000
   24   4   4   1   1
      0.3849988706    1.0000000000
   25   4   4   1   1
      1.0675377716    1.0000000000

# RI basis set for Al (all-electron) relative DI metric: 9.4e-05
Al  RI_aug-TZVP-MOLOPT-ae_N_RI_117_s_p_d_f_g_h_i_7_6_6_5_3_0_0_error_9.4e-05
   27
    1   0   0   1   1
      0.1533697652    1.0000000000
    2   0   0   1   1
      0.3567686493    1.0000000000
    3   0   0   1   1
      1.1371332505    1.0000000000
    4   0   0   1   1
      4.1412636573    1.0000000000
    5   0   0   1   1
     15.6366262569    1.0000000000
    6   0   0   1   1
     57.2720680563    1.0000000000
    7   0   0   1   1
    207.0691524920    1.0000000000
    8   1   1   1   1
      0.1695023584    1.0000000000
    9   1   1   1   1
      0.2640729309    1.0000000000
   10   1   1   1   1
      0.6890440486    1.0000000000
   11   1   1   1   1
      2.3862673114    1.0000000000
   12   1   1   1   1
      8.7323376862    1.0000000000
   13   1   1   1   1
     28.0415787419    1.0000000000
   14   2   2   1   1
      0.1724224470    1.0000000000
   15   2   2   1   1
      0.3627986148    1.0000000000
   16   2   2   1   1
      0.8051010334    1.0000000000
   17   2   2   1   1
      1.9769804707    1.0000000000
   18   2   2   1   1
      5.3779339048    1.0000000000
   19   2   2   1   1
     14.2321938414    1.0000000000
   20   3   3   1   1
      0.1235040418    1.0000000000
   21   3   3   1   1
      0.3331293393    1.0000000000
   22   3   3   1   1
      0.7958932262    1.0000000000
   23   3   3   1   1
      1.4270938277    1.0000000000
   24   3   3   1   1
      2.5059035190    1.0000000000
   25   4   4   1   1
      0.1906813095    1.0000000000
   26   4   4   1   1
      0.3740899909    1.0000000000
   27   4   4   1   1
      1.0466606305    1.0000000000
```

```
# RI basis set for Si (all-electron) relative DI metric: 3.9e-02
Si  RI_aug-SZV-MOLOPT-ae-mini_N_RI_009_s_p_d_f_g_h_i_3_2_0_0_0_0_0_error_3.9e-02
   5
     1   0   0   1   1
      0.2646431963    1.0000000000
     2   0   0   1   1
      0.5763701985    1.0000000000
     3   0   0   1   1
      1.1065458541    1.0000000000
     4   1   1   1   1
      0.2723303396    1.0000000000
     5   1   1   1   1
      0.4922516821    1.0000000000

# RI basis set for Si (all-electron) relative DI metric: 2.4e-03
Si  RI_aug-SZV-MOLOPT-ae-mini_N_RI_014_s_p_d_f_g_h_i_3_2_1_0_0_0_0_error_2.4e-03
   6
     1   0   0   1   1
      0.2342917848    1.0000000000
     2   0   0   1   1
      0.5274621214    1.0000000000
     3   0   0   1   1
      1.4899502774    1.0000000000
     4   1   1   1   1
      0.1648281349    1.0000000000
     5   1   1   1   1
      0.5364102357    1.0000000000
     6   2   2   1   1
      0.3865257808    1.0000000000

# RI basis set for Si (all-electron) relative DI metric: 7.4e-05
Si  RI_aug-SZV-MOLOPT-ae-mini_N_RI_020_s_p_d_f_g_h_i_4_2_2_0_0_0_0_error_7.4e-05
   8
     1   0   0   1   1
      0.1870880444    1.0000000000
     2   0   0   1   1
      0.4280364717    1.0000000000
     3   0   0   1   1
      0.9265276410    1.0000000000
     4   0   0   1   1
      2.1259615747    1.0000000000
     5   1   1   1   1
      0.1619599640    1.0000000000
     6   1   1   1   1
      0.4822186340    1.0000000000
     7   2   2   1   1
      0.2046478549    1.0000000000
     8   2   2   1   1
      0.6536273161    1.0000000000

# RI basis set for Si (all-electron) relative DI metric: 5.7e-07
Si  RI_aug-SZV-MOLOPT-ae-mini_N_RI_021_s_p_d_f_g_h_i_5_2_2_0_0_0_0_error_5.7e-07
   9
     1   0   0   1   1
      0.1960034633    1.0000000000
     2   0   0   1   1
      0.3807565845    1.0000000000
     3   0   0   1   1
      0.7330966318    1.0000000000
     4   0   0   1   1
      1.3512761937    1.0000000000
     5   0   0   1   1
      2.4540927727    1.0000000000
     6   1   1   1   1
      0.2618934571    1.0000000000
     7   1   1   1   1
      0.4646386425    1.0000000000
     8   2   2   1   1
      0.2204279916    1.0000000000
     9   2   2   1   1
      0.5718799572    1.0000000000

# RI basis set for Si (all-electron) relative DI metric: 2.5e-07
Si  RI_aug-SZV-MOLOPT-ae-mini_N_RI_033_s_p_d_f_g_h_i_6_4_3_0_0_0_0_error_2.5e-07
   13
     1   0   0   1   1
      0.2250793212    1.0000000000
     2   0   0   1   1
      0.3632431600    1.0000000000
     3   0   0   1   1
      0.5862172476    1.0000000000
     4   0   0   1   1
      0.9461024868    1.0000000000
     5   0   0   1   1
      1.5269874132    1.0000000000
     6   0   0   1   1
      2.4645868515    1.0000000000
     7   1   1   1   1
      0.2264471398    1.0000000000
     8   1   1   1   1
      0.5002911258    1.0000000000
     9   1   1   1   1
      1.1089267037    1.0000000000
    10   1   1   1   1
      2.4638698398    1.0000000000
    11   2   2   1   1
      0.2221866093    1.0000000000
    12   2   2   1   1
      0.6654385237    1.0000000000
    13   2   2   1   1
      2.4473404638    1.0000000000

# RI basis set for Si (all-electron) relative DI metric: 4.8e-08
Si  RI_aug-SZV-MOLOPT-ae-mini_N_RI_039_s_p_d_f_g_h_i_7_4_4_0_0_0_0_error_4.8e-08
   15
     1   0   0   1   1
      0.2250000000    1.0000000000
     2   0   0   1   1
      0.3353067085    1.0000000000
     3   0   0   1   1
      0.4996915055    1.0000000000
```

```
     4   0   0   1   1
      0.7446662843    1.0000000000
     5   0   0   1   1
      1.1097404476    1.0000000000
     6   0   0   1   1
      1.6537929632    1.0000000000
     7   0   0   1   1
      2.4645683333    1.0000000000
     8   1   1   1   1
      0.2250000014    1.0000000000
     9   1   1   1   1
      0.4996915059    1.0000000000
    10   1   1   1   1
      1.1097404468    1.0000000000
    11   1   1   1   1
      2.4645683327    1.0000000000
    12   2   2   1   1
      0.2250000000    1.0000000000
    13   2   2   1   1
      0.4996915055    1.0000000000
    14   2   2   1   1
      1.1097404476    1.0000000000
    15   2   2   1   1
      2.4645683333    1.0000000000

# RI basis set for Si (all-electron) relative DI metric: 7.9e-09
Si  RI_aug-SZV-MOLOPT-ae-mini_N_RI_042_s_p_d_f_g_h_i_7_5_4_0_0_0_0_error_7.9e-09
   16
     1   0   0   1   1
      0.2250000000    1.0000000000
     2   0   0   1   1
      0.3353067085    1.0000000000
     3   0   0   1   1
      0.4996915055    1.0000000000
     4   0   0   1   1
      0.7446662843    1.0000000000
     5   0   0   1   1
      1.1097404476    1.0000000000
     6   0   0   1   1
      1.6537929632    1.0000000000
     7   0   0   1   1
      2.4645683333    1.0000000000
     8   1   1   1   1
      0.2250000000    1.0000000000
     9   1   1   1   1
      0.4093286137    1.0000000000
    10   1   1   1   1
      0.7446662843    1.0000000000
    11   1   1   1   1
      1.3547254125    1.0000000000
    12   1   1   1   1
      2.4645683333    1.0000000000
    13   2   2   1   1
      0.2250000000    1.0000000000
    14   2   2   1   1
      0.4996915055    1.0000000000
    15   2   2   1   1
      1.1097404476    1.0000000000
    16   2   2   1   1
      2.4645683333    1.0000000000

# RI basis set for Si (all-electron) relative DI metric: 2.7e-02
Si  RI_aug-SZV-MOLOPT-ae-SR_N_RI_019_s_p_d_f_g_h_i_5_3_1_0_0_0_0_error_2.7e-02
   9
     1   0   0   1   1
      0.2668401934    1.0000000000
     2   0   0   1   1
      0.7290121777    1.0000000000
     3   0   0   1   1
      4.8169304251    1.0000000000
     4   0   0   1   1
     34.9688885642    1.0000000000
     5   0   0   1   1
    243.1643203396    1.0000000000
     6   1   1   1   1
      0.3392174348    1.0000000000
     7   1   1   1   1
      3.7162842654    1.0000000000
     8   1   1   1   1
     38.5929458711    1.0000000000
     9   2   2   1   1
      0.4824179975    1.0000000000

# RI basis set for Si (all-electron) relative DI metric: 1.1e-03
Si  RI_aug-SZV-MOLOPT-ae-SR_N_RI_034_s_p_d_f_g_h_i_7_5_1_1_0_0_0_error_1.1e-03
   14
     1   0   0   1   1
      0.2242605179    1.0000000000
     2   0   0   1   1
      0.5818150370    1.0000000000
     3   0   0   1   1
      1.9868210375    1.0000000000
     4   0   0   1   1
      7.3289544039    1.0000000000
     5   0   0   1   1
     23.9952432630    1.0000000000
     6   0   0   1   1
     76.5634505393    1.0000000000
     7   0   0   1   1
    243.3076814010    1.0000000000
     8   1   1   1   1
      0.2480835833    1.0000000000
     9   1   1   1   1
      0.7529647539    1.0000000000
    10   1   1   1   1
      2.6923177670    1.0000000000
    11   1   1   1   1
      9.5613535147    1.0000000000
    12   1   1   1   1
     33.7399673920    1.0000000000
    13   2   2   1   1
```

```
      0.3832332989    1.0000000000
    14   3   3   1   1
      0.3342848887    1.0000000000

# RI basis set for Si (all-electron) relative DI metric: 1.0e-04
Si  RI_aug-SZV-MOLOPT-ae-SR_N_RI_053_s_p_d_f_g_h_i_7_5_3_1_1_0_0_error_1.0e-04
   17
     1   0   0   1   1
      0.2269571353    1.0000000000
     2   0   0   1   1
      0.4789108490    1.0000000000
     3   0   0   1   1
      1.5066946215    1.0000000000
     4   0   0   1   1
      5.1901385874    1.0000000000
     5   0   0   1   1
     18.7041457908    1.0000000000
     6   0   0   1   1
     67.5303169257    1.0000000000
     7   0   0   1   1
    243.8295019860    1.0000000000
     8   1   1   1   1
      0.2219894472    1.0000000000
     9   1   1   1   1
      0.5167637810    1.0000000000
    10   1   1   1   1
      2.8155220035    1.0000000000
    11   1   1   1   1
     14.1105785999    1.0000000000
    12   1   1   1   1
     67.8732342262    1.0000000000
    13   2   2   1   1
      0.2969348431    1.0000000000
    14   2   2   1   1
      1.1208101564    1.0000000000
    15   2   2   1   1
     15.7815285571    1.0000000000
    16   3   3   1   1
      0.2800775652    1.0000000000
    17   4   4   1   1
      0.9877232823    1.0000000000

# RI basis set for Si (all-electron) relative DI metric: 6.2e-06
Si  RI_aug-SZV-MOLOPT-ae-SR_N_RI_069_s_p_d_f_g_h_i_7_5_4_1_1_1_0_error_6.2e-06
   19
     1   0   0   1   1
      0.2249942678    1.0000000000
     2   0   0   1   1
      0.2843145141    1.0000000000
     3   0   0   1   1
      1.1704741093    1.0000000000
     4   0   0   1   1
      4.8841824395    1.0000000000
     5   0   0   1   1
     18.3518861431    1.0000000000
     6   0   0   1   1
     67.1529453534    1.0000000000
     7   0   0   1   1
    243.4279389842    1.0000000000
     8   1   1   1   1
      0.2481690816    1.0000000000
     9   1   1   1   1
      0.4048639640    1.0000000000
    10   1   1   1   1
      1.9532771314    1.0000000000
    11   1   1   1   1
      8.7985352357    1.0000000000
    12   1   1   1   1
     35.4533120788    1.0000000000
    13   2   2   1   1
      0.2358278082    1.0000000000
    14   2   2   1   1
      0.6198309539    1.0000000000
    15   2   2   1   1
      3.9033520510    1.0000000000
    16   2   2   1   1
     18.3280994095    1.0000000000
    17   3   3   1   1
      0.3756020976    1.0000000000
    18   4   4   1   1
      0.8517710399    1.0000000000
    19   5   5   1   1
      1.5446348872    1.0000000000

# RI basis set for Si (all-electron) relative DI metric: 9.6e-07
Si  RI_aug-SZV-MOLOPT-ae-SR_N_RI_105_s_p_d_f_g_h_i_7_5_4_3_2_1_1_error_9.6e-07
   23
     1   0   0   1   1
      0.2124442712    1.0000000000
     2   0   0   1   1
      0.4271498770    1.0000000000
     3   0   0   1   1
      1.4409673671    1.0000000000
     4   0   0   1   1
      5.1718302861    1.0000000000
     5   0   0   1   1
     18.6914899238    1.0000000000
     6   0   0   1   1
     67.5159378304    1.0000000000
     7   0   0   1   1
    243.8139731961    1.0000000000
     8   1   1   1   1
      0.2179933351    1.0000000000
     9   1   1   1   1
      0.5166364870    1.0000000000
    10   1   1   1   1
      2.8803242373    1.0000000000
    11   1   1   1   1
     14.1858701668    1.0000000000
    12   1   1   1   1
     67.9428262631    1.0000000000
```

```
    13   2   2   1   1
      0.2484924267    1.0000000000
    14   2   2   1   1
      0.6819562054    1.0000000000
    15   2   2   1   1
      3.7367585290    1.0000000000
    16   2   2   1   1
     18.9025612962    1.0000000000
    17   3   3   1   1
      0.2783825698    1.0000000000
    18   3   3   1   1
      0.9669591531    1.0000000000
    19   3   3   1   1
      4.3186564292    1.0000000000
    20   4   4   1   1
      0.2097066609    1.0000000000
    21   4   4   1   1
      1.2975358516    1.0000000000
    22   5   5   1   1
      0.7974852271    1.0000000000
    23   6   6   1   1
      0.7609785222    1.0000000000

# RI basis set for Si (all-electron) relative DI metric: 1.3e-02
Si  RI_aug-SZV-MOLOPT-ae_N_RI_021_s_p_d_f_g_h_i_7_3_1_0_0_0_error_1.3e-02
   11
     1   0   0   1   1
      0.2247079981    1.0000000000
     2   0   0   1   1
      0.4704859104    1.0000000000
     3   0   0   1   1
      1.5028821939    1.0000000000
     4   0   0   1   1
      5.2193543489    1.0000000000
     5   0   0   1   1
     18.7418543706    1.0000000000
     6   0   0   1   1
     67.5707696130    1.0000000000
     7   0   0   1   1
    243.8727424005    1.0000000000
     8   1   1   1   1
      0.3097250172    1.0000000000
     9   1   1   1   1
      1.7269609387    1.0000000000
    10   1   1   1   1
     50.3917798237    1.0000000000
    11   2   2   1   1
      0.4401352998    1.0000000000

# RI basis set for Si (all-electron) relative DI metric: 5.1e-03
Si  RI_aug-SZV-MOLOPT-ae_N_RI_041_s_p_d_f_g_h_i_7_4_3_1_0_0_0_error_5.1e-03
   15
     1   0   0   1   1
      0.2294233533    1.0000000000
     2   0   0   1   1
      0.4729457920    1.0000000000
     3   0   0   1   1
      1.5035820303    1.0000000000
     4   0   0   1   1
      5.2074920672    1.0000000000
     5   0   0   1   1
     18.7221198220    1.0000000000
     6   0   0   1   1
     67.5502197548    1.0000000000
     7   0   0   1   1
    243.8511511495    1.0000000000
     8   1   1   1   1
      0.2622001317    1.0000000000
     9   1   1   1   1
      1.4289049217    1.0000000000
    10   1   1   1   1
      8.7593361140    1.0000000000
    11   1   1   1   1
     45.1988021254    1.0000000000
    12   2   2   1   1
      0.4698603421    1.0000000000
    13   2   2   1   1
      1.0161836168    1.0000000000
    14   2   2   1   1
     18.4106454400    1.0000000000
    15   3   3   1   1
      0.8119225063    1.0000000000

# RI basis set for Si (all-electron) relative DI metric: 5.1e-04
Si  RI_aug-SZV-MOLOPT-ae_N_RI_044_s_p_d_f_g_h_i_7_5_3_1_0_0_0_error_5.1e-04
   16
     1   0   0   1   1
      0.1652651257    1.0000000000
     2   0   0   1   1
      0.4078700943    1.0000000000
     3   0   0   1   1
      1.4425935929    1.0000000000
     4   0   0   1   1
      5.1836933961    1.0000000000
     5   0   0   1   1
     18.7053520824    1.0000000000
     6   0   0   1   1
     67.5310986237    1.0000000000
     7   0   0   1   1
    243.8301232616    1.0000000000
     8   1   1   1   1
      0.2071376150    1.0000000000
     9   1   1   1   1
      0.4803734648    1.0000000000
    10   1   1   1   1
      2.8532674466    1.0000000000
    11   1   1   1   1
     14.1587767318    1.0000000000
    12   1   1   1   1
     67.9141710279    1.0000000000
    13   2   2   1   1
```

```
      0.4042843159    1.0000000000
    14   2   2   1   1
      1.9937010870    1.0000000000
    15   2   2   1   1
     18.0906973637    1.0000000000
    16   3   3   1   1
      0.4383282583    1.0000000000

# RI basis set for Si (all-electron) relative DI metric: 1.8e-04
Si  RI_aug-SZV-MOLOPT-ae_N_RI_051_s_p_d_f_g_h_i_7_5_3_2_0_0_0_error_1.8e-04
   17
     1   0   0   1   1
      0.2087637618    1.0000000000
     2   0   0   1   1
      0.4045052402    1.0000000000
     3   0   0   1   1
      1.4274445226    1.0000000000
     4   0   0   1   1
      5.1696092823    1.0000000000
     5   0   0   1   1
     18.6907606858    1.0000000000
     6   0   0   1   1
     67.5152658192    1.0000000000
     7   0   0   1   1
    243.8131200907    1.0000000000
     8   1   1   1   1
      0.2742518198    1.0000000000
     9   1   1   1   1
      0.4494911856    1.0000000000
    10   1   1   1   1
      2.8080193638    1.0000000000
    11   1   1   1   1
     14.1090990929    1.0000000000
    12   1   1   1   1
     67.8684386318    1.0000000000
    13   2   2   1   1
      0.3002919645    1.0000000000
    14   2   2   1   1
      0.8304579224    1.0000000000
    15   2   2   1   1
     16.9043160026    1.0000000000
    16   3   3   1   1
      0.4197878303    1.0000000000
    17   3   3   1   1
      4.1567115861    1.0000000000

# RI basis set for Si (all-electron) relative DI metric: 7.9e-05
Si  RI_aug-SZV-MOLOPT-ae_N_RI_081_s_p_d_f_g_h_i_7_6_3_3_1_1_0_error_7.9e-05
   21
     1   0   0   1   1
      0.1796843985    1.0000000000
     2   0   0   1   1
      0.3425238110    1.0000000000
     3   0   0   1   1
      1.3772654852    1.0000000000
     4   0   0   1   1
      5.1398558926    1.0000000000
     5   0   0   1   1
     18.6603830739    1.0000000000
     6   0   0   1   1
     67.4822298538    1.0000000000
     7   0   0   1   1
    243.7776731804    1.0000000000
     8   1   1   1   1
      0.2098726365    1.0000000000
     9   1   1   1   1
      0.4792057601    1.0000000000
    10   1   1   1   1
      1.5731133647    1.0000000000
    11   1   1   1   1
      5.5512751730    1.0000000000
    12   1   1   1   1
     19.4462302552    1.0000000000
    13   1   1   1   1
     67.9610913225    1.0000000000
    14   2   2   1   1
      0.2599241803    1.0000000000
    15   2   2   1   1
      0.8295508477    1.0000000000
    16   2   2   1   1
     15.4027659484    1.0000000000
    17   3   3   1   1
      0.3178592704    1.0000000000
    18   3   3   1   1
      0.9557109840    1.0000000000
    19   3   3   1   1
      4.2992041178    1.0000000000
    20   4   4   1   1
      0.9998431863    1.0000000000
    21   5   5   1   1
      0.8432105206    1.0000000000

# RI basis set for Si (all-electron) relative DI metric: 5.0e-03
Si  RI_aug-DZVP-MOLOPT-ae-SR_N_RI_043_s_p_d_f_g_h_i_6_5_3_1_0_0_0_error_5.0e-03
   15
     1   0   0   1   1
      0.1843011473    1.0000000000
     2   0   0   1   1
      0.5065166369    1.0000000000
     3   0   0   1   1
      2.0969693993    1.0000000000
     4   0   0   1   1
     10.5636397625    1.0000000000
     5   0   0   1   1
     51.4872028398    1.0000000000
     6   0   0   1   1
    242.9987707061    1.0000000000
     7   1   1   1   1
      0.2986051483    1.0000000000
     8   1   1   1   1
      0.6218638942    1.0000000000
```

```
    9   1   1   1   1
     2.6099729704    1.0000000000
   10   1   1   1   1
    13.6575352484    1.0000000000
   11   1   1   1   1
    67.4303473069    1.0000000000
   12   2   2   1   1
     0.3094232740    1.0000000000
   13   2   2   1   1
     1.5055132848    1.0000000000
   14   2   2   1   1
    12.7241483286    1.0000000000
   15   3   3   1   1
     0.5302519482    1.0000000000

# RI basis set for Si (all-electron) relative DI metric: 1.1e-04
Si  RI_aug-DZVP-MOLOPT-ae-SR_N_RI_065_s_p_d_f_g_h_i_7_5_4_2_1_0_0_error_1.1e-04
   19
    1   0   0   1   1
     0.2062138331    1.0000000000
    2   0   0   1   1
     0.3671467361    1.0000000000
    3   0   0   1   1
     1.2834394708    1.0000000000
    4   0   0   1   1
     5.0222127811    1.0000000000
    5   0   0   1   1
    18.5774517926    1.0000000000
    6   0   0   1   1
    67.4030502593    1.0000000000
    7   0   0   1   1
   243.6922751643    1.0000000000
    8   1   1   1   1
     0.2701146257    1.0000000000
    9   1   1   1   1
     0.4362312684    1.0000000000
   10   1   1   1   1
     1.9837326164    1.0000000000
   11   1   1   1   1
    10.8114198983    1.0000000000
   12   1   1   1   1
    64.1028586622    1.0000000000
   13   2   2   1   1
     0.3001440778    1.0000000000
   14   2   2   1   1
     0.5478853346    1.0000000000
   15   2   2   1   1
     3.1630732933    1.0000000000
   16   2   2   1   1
    18.3983279143    1.0000000000
   17   3   3   1   1
     0.3183947450    1.0000000000
   18   3   3   1   1
     1.0419806461    1.0000000000
   19   4   4   1   1
     0.4041851474    1.0000000000

# RI basis set for Si (all-electron) relative DI metric: 4.5e-05
Si  RI_aug-DZVP-MOLOPT-ae-SR_N_RI_068_s_p_d_f_g_h_i_7_6_4_2_1_0_0_error_4.5e-05
   20
    1   0   0   1   1
     0.2153518647    1.0000000000
    2   0   0   1   1
     0.3694716660    1.0000000000
    3   0   0   1   1
     1.2150382776    1.0000000000
    4   0   0   1   1
     4.7958281713    1.0000000000
    5   0   0   1   1
    18.2833596352    1.0000000000
    6   0   0   1   1
    67.0875145108    1.0000000000
    7   0   0   1   1
   243.3572275543    1.0000000000
    8   1   1   1   1
     0.2639754096    1.0000000000
    9   1   1   1   1
     0.4601564575    1.0000000000
   10   1   1   1   1
     1.4963716433    1.0000000000
   11   1   1   1   1
     5.4101466978    1.0000000000
   12   1   1   1   1
    19.3038897956    1.0000000000
   13   1   1   1   1
    67.8277590293    1.0000000000
   14   2   2   1   1
     0.2639784300    1.0000000000
   15   2   2   1   1
     0.5520111573    1.0000000000
   16   2   2   1   1
     3.0966631520    1.0000000000
   17   2   2   1   1
    18.2940243116    1.0000000000
   18   3   3   1   1
     0.3274581904    1.0000000000
   19   3   3   1   1
     0.8414769720    1.0000000000
   20   4   4   1   1
     0.3489091707    1.0000000000

# RI basis set for Si (all-electron) relative DI metric: 1.8e-05
Si  RI_aug-DZVP-MOLOPT-ae-SR_N_RI_080_s_p_d_f_g_h_i_7_6_5_3_1_0_0_error_1.8e-05
   22
    1   0   0   1   1
     0.2191207275    1.0000000000
    2   0   0   1   1
     0.3292964898    1.0000000000
    3   0   0   1   1
     1.0576706158    1.0000000000
    4   0   0   1   1
```

```
      4.2418571022    1.0000000000
     5   0   0   1   1
     17.5338505100    1.0000000000
     6   0   0   1   1
     66.2970662752    1.0000000000
     7   0   0   1   1
    242.5203402395    1.0000000000
     8   1   1   1   1
      0.2002048116    1.0000000000
     9   1   1   1   1
      0.5381907986    1.0000000000
    10   1   1   1   1
      1.3849307477    1.0000000000
    11   1   1   1   1
      5.2303465145    1.0000000000
    12   1   1   1   1
     19.1200651460    1.0000000000
    13   1   1   1   1
     67.6457438401    1.0000000000
    14   2   2   1   1
      0.2384399860    1.0000000000
    15   2   2   1   1
      0.5500900068    1.0000000000
    16   2   2   1   1
      1.5864493931    1.0000000000
    17   2   2   1   1
      5.5706392360    1.0000000000
    18   2   2   1   1
     18.8791068881    1.0000000000
    19   3   3   1   1
      0.2634741496    1.0000000000
    20   3   3   1   1
      0.6934743347    1.0000000000
    21   3   3   1   1
      4.1317352771    1.0000000000
    22   4   4   1   1
      0.3219403731    1.0000000000

# RI basis set for Si (all-electron) relative DI metric: 8.8e-06
Si  RI_aug-DZVP-MOLOPT-ae-SR_N_RI_094_s_p_d_f_g_h_i_7_6_5_5_1_0_0_error_8.8e-06
   24
     1   0   0   1   1
      0.2262003190    1.0000000000
     2   0   0   1   1
      0.3185014807    1.0000000000
     3   0   0   1   1
      1.0563751670    1.0000000000
     4   0   0   1   1
      4.2652345926    1.0000000000
     5   0   0   1   1
     17.5673821505    1.0000000000
     6   0   0   1   1
     66.3293470328    1.0000000000
     7   0   0   1   1
    242.5521014592    1.0000000000
     8   1   1   1   1
      0.1986128917    1.0000000000
     9   1   1   1   1
      0.5326163428    1.0000000000
    10   1   1   1   1
      1.3735214282    1.0000000000
    11   1   1   1   1
      5.0100564211    1.0000000000
    12   1   1   1   1
     18.9207423670    1.0000000000
    13   1   1   1   1
     67.4809041759    1.0000000000
    14   2   2   1   1
      0.2399887651    1.0000000000
    15   2   2   1   1
      0.5454024997    1.0000000000
    16   2   2   1   1
      1.5623656334    1.0000000000
    17   2   2   1   1
      5.5434690654    1.0000000000
    18   2   2   1   1
     18.8561519335    1.0000000000
    19   3   3   1   1
      0.1865709494    1.0000000000
    20   3   3   1   1
      0.4133695912    1.0000000000
    21   3   3   1   1
      0.9074010757    1.0000000000
    22   3   3   1   1
      1.9965059387    1.0000000000
    23   3   3   1   1
      4.2673343047    1.0000000000
    24   4   4   1   1
      0.3257400060    1.0000000000

# RI basis set for Si (all-electron) relative DI metric: 7.3e-03
Si  RI_aug-DZVP-MOLOPT-ae_N_RI_043_s_p_d_f_g_h_i_6_5_3_1_0_0_0_error_7.3e-03
   15
     1   0   0   1   1
      0.1930690656    1.0000000000
     2   0   0   1   1
      0.4463672377    1.0000000000
     3   0   0   1   1
      1.9678373575    1.0000000000
     4   0   0   1   1
     10.3883652787    1.0000000000
     5   0   0   1   1
     51.2985861681    1.0000000000
     6   0   0   1   1
    242.7928871781    1.0000000000
     7   1   1   1   1
      0.2873930029    1.0000000000
     8   1   1   1   1
      0.5918895462    1.0000000000
     9   1   1   1   1
      2.7011307313    1.0000000000
```

```
    10   1   1   1   1
     13.8907941322    1.0000000000
    11   1   1   1   1
     67.6599353183    1.0000000000
    12   2   2   1   1
      0.3158663255    1.0000000000
    13   2   2   1   1
      1.8471101071    1.0000000000
    14   2   2   1   1
     12.9478762654    1.0000000000
    15   3   3   1   1
      0.5175002308    1.0000000000

# RI basis set for Si (all-electron) relative DI metric: 2.3e-03
Si  RI_aug-DZVP-MOLOPT-ae_N_RI_057_s_p_d_f_g_h_i_6_5_3_3_0_0_0_error_2.3e-03
   17
     1   0   0   1   1
      0.1702539565    1.0000000000
     2   0   0   1   1
      0.4191114255    1.0000000000
     3   0   0   1   1
      1.8570845493    1.0000000000
     4   0   0   1   1
     10.0837926364    1.0000000000
     5   0   0   1   1
     50.9394136466    1.0000000000
     6   0   0   1   1
    242.4014951589    1.0000000000
     7   1   1   1   1
      0.2505969412    1.0000000000
     8   1   1   1   1
      0.5116563383    1.0000000000
     9   1   1   1   1
      2.6198883248    1.0000000000
    10   1   1   1   1
     13.8082195968    1.0000000000
    11   1   1   1   1
     67.5862974513    1.0000000000
    12   2   2   1   1
      0.1974840745    1.0000000000
    13   2   2   1   1
      0.8720597790    1.0000000000
    14   2   2   1   1
      8.2839421653    1.0000000000
    15   3   3   1   1
      0.3788140024    1.0000000000
    16   3   3   1   1
      0.8689519206    1.0000000000
    17   3   3   1   1
      4.3238280850    1.0000000000

# RI basis set for Si (all-electron) relative DI metric: 9.3e-04
Si  RI_aug-DZVP-MOLOPT-ae_N_RI_066_s_p_d_f_g_h_i_6_5_3_3_1_0_0_error_9.3e-04
   18
     1   0   0   1   1
      0.1847897772    1.0000000000
     2   0   0   1   1
      0.4184307016    1.0000000000
     3   0   0   1   1
      1.7439995985    1.0000000000
     4   0   0   1   1
      9.6030727451    1.0000000000
     5   0   0   1   1
     50.3171191267    1.0000000000
     6   0   0   1   1
    241.7209007444    1.0000000000
     7   1   1   1   1
      0.1936926415    1.0000000000
     8   1   1   1   1
      0.5503347542    1.0000000000
     9   1   1   1   1
      3.3983859827    1.0000000000
    10   1   1   1   1
     15.3235509373    1.0000000000
    11   1   1   1   1
     65.0272392796    1.0000000000
    12   2   2   1   1
      0.2144788893    1.0000000000
    13   2   2   1   1
      0.7764121241    1.0000000000
    14   2   2   1   1
      4.6697149112    1.0000000000
    15   3   3   1   1
      0.3470142575    1.0000000000
    16   3   3   1   1
      1.1653946941    1.0000000000
    17   3   3   1   1
      3.0630346774    1.0000000000
    18   4   4   1   1
      0.7358443877    1.0000000000

# RI basis set for Si (all-electron) relative DI metric: 9.4e-05
Si  RI_aug-DZVP-MOLOPT-ae_N_RI_070_s_p_d_f_g_h_i_7_6_3_3_1_0_0_error_9.4e-05
   20
     1   0   0   1   1
      0.1651235610    1.0000000000
     2   0   0   1   1
      0.3357758073    1.0000000000
     3   0   0   1   1
      1.1322659891    1.0000000000
     4   0   0   1   1
      4.5899049645    1.0000000000
     5   0   0   1   1
     18.0366580221    1.0000000000
     6   0   0   1   1
     66.8250038103    1.0000000000
     7   0   0   1   1
    243.0788552456    1.0000000000
     8   1   1   1   1
      0.2096215919    1.0000000000
     9   1   1   1   1
```

```
      0.3947346544    1.0000000000
    10   1   1   1   1
      1.3539767796    1.0000000000
    11   1   1   1   1
      5.2994958673    1.0000000000
    12   1   1   1   1
     19.2344491747    1.0000000000
    13   1   1   1   1
     67.7694972092    1.0000000000
    14   2   2   1   1
      0.2746520146    1.0000000000
    15   2   2   1   1
      0.6050154581    1.0000000000
    16   2   2   1   1
      6.2064276494    1.0000000000
    17   3   3   1   1
      0.2699008266    1.0000000000
    18   3   3   1   1
      0.7514565138    1.0000000000
    19   3   3   1   1
      4.2866224629    1.0000000000
    20   4   4   1   1
      0.3067086688    1.0000000000

# RI basis set for Si (all-electron) relative DI metric: 4.1e-05
Si  RI_aug-DZVP-MOLOPT-ae_N_RI_080_s_p_d_f_g_h_i_7_6_5_3_1_0_0_error_4.1e-05
   22
     1   0   0   1   1
      0.1717522483    1.0000000000
     2   0   0   1   1
      0.2799643418    1.0000000000
     3   0   0   1   1
      0.9925499448    1.0000000000
     4   0   0   1   1
      3.8729776242    1.0000000000
     5   0   0   1   1
     16.8279204306    1.0000000000
     6   0   0   1   1
     65.5326368556    1.0000000000
     7   0   0   1   1
    241.7133383578    1.0000000000
     8   1   1   1   1
      0.1991653386    1.0000000000
     9   1   1   1   1
      0.3449777697    1.0000000000
    10   1   1   1   1
      1.1565432149    1.0000000000
    11   1   1   1   1
      4.8691280187    1.0000000000
    12   1   1   1   1
     18.8745661972    1.0000000000
    13   1   1   1   1
     67.4484410080    1.0000000000
    14   2   2   1   1
      0.2108208287    1.0000000000
    15   2   2   1   1
      0.5450631359    1.0000000000
    16   2   2   1   1
      1.5487587346    1.0000000000
    17   2   2   1   1
      5.5175440359    1.0000000000
    18   2   2   1   1
     18.8391849945    1.0000000000
    19   3   3   1   1
      0.2487861066    1.0000000000
    20   3   3   1   1
      0.7059755144    1.0000000000
    21   3   3   1   1
      4.1893593308    1.0000000000
    22   4   4   1   1
      0.2904248227    1.0000000000

# RI basis set for Si (all-electron) relative DI metric: 7.3e-06
Si  RI_aug-DZVP-MOLOPT-ae_N_RI_115_s_p_d_f_g_h_i_7_6_6_6_2_0_0_error_7.3e-06
   27
     1   0   0   1   1
      0.1911233297    1.0000000000
     2   0   0   1   1
      0.3823226970    1.0000000000
     3   0   0   1   1
      1.1631236406    1.0000000000
     4   0   0   1   1
      4.7840753082    1.0000000000
     5   0   0   1   1
     19.5907693305    1.0000000000
     6   0   0   1   1
     69.5600688748    1.0000000000
     7   0   0   1   1
    234.5150521703    1.0000000000
     8   1   1   1   1
      0.1739570716    1.0000000000
     9   1   1   1   1
      0.3924703999    1.0000000000
    10   1   1   1   1
      1.1413646845    1.0000000000
    11   1   1   1   1
      4.4481695162    1.0000000000
    12   1   1   1   1
     18.4067619042    1.0000000000
    13   1   1   1   1
     67.0583509382    1.0000000000
    14   2   2   1   1
      0.2363899936    1.0000000000
    15   2   2   1   1
      0.5655550326    1.0000000000
    16   2   2   1   1
      1.3812538781    1.0000000000
    17   2   2   1   1
      3.3190206053    1.0000000000
    18   2   2   1   1
      7.9190725685    1.0000000000
```

```
    19   2   2   1   1
     18.8416153714    1.0000000000
    20   3   3   1   1
      0.1765822739    1.0000000000
    21   3   3   1   1
      0.3789836334    1.0000000000
    22   3   3   1   1
      0.6991423946    1.0000000000
    23   3   3   1   1
      1.2972663712    1.0000000000
    24   3   3   1   1
      2.3746684505    1.0000000000
    25   3   3   1   1
      4.3230414800    1.0000000000
    26   4   4   1   1
      0.1735351971    1.0000000000
    27   4   4   1   1
      0.3847349515    1.0000000000

# RI basis set for Si (all-electron) relative DI metric: 6.1e-03
Si  RI_aug-TZVP-MOLOPT-ae_N_RI_044_s_p_d_f_g_h_i_7_5_3_1_0_0_0_error_6.1e-03
   16
     1   0   0   1   1
      0.2015296193    1.0000000000
     2   0   0   1   1
      0.4239793584    1.0000000000
     3   0   0   1   1
      1.3684850156    1.0000000000
     4   0   0   1   1
      4.9907392402    1.0000000000
     5   0   0   1   1
     18.4635679366    1.0000000000
     6   0   0   1   1
     67.2669861233    1.0000000000
     7   0   0   1   1
    243.5469115781    1.0000000000
     8   1   1   1   1
      0.3280341368    1.0000000000
     9   1   1   1   1
      0.4641562017    1.0000000000
    10   1   1   1   1
      2.1096553774    1.0000000000
    11   1   1   1   1
     12.5794298637    1.0000000000
    12   1   1   1   1
     66.5085798313    1.0000000000
    13   2   2   1   1
      0.3741018363    1.0000000000
    14   2   2   1   1
      0.6205099588    1.0000000000
    15   2   2   1   1
      7.7699359926    1.0000000000
    16   3   3   1   1
      0.4771794519    1.0000000000

# RI basis set for Si (all-electron) relative DI metric: 2.2e-03
Si  RI_aug-TZVP-MOLOPT-ae_N_RI_061_s_p_d_f_g_h_i_7_6_3_3_0_0_0_error_2.2e-03
   19
     1   0   0   1   1
      0.1816287217    1.0000000000
     2   0   0   1   1
      0.3386401775    1.0000000000
     3   0   0   1   1
      0.8791600470    1.0000000000
     4   0   0   1   1
      3.4097198428    1.0000000000
     5   0   0   1   1
     16.0220284192    1.0000000000
     6   0   0   1   1
     64.1203929435    1.0000000000
     7   0   0   1   1
    232.2062420927    1.0000000000
     8   1   1   1   1
      0.1832805565    1.0000000000
     9   1   1   1   1
      0.4164490574    1.0000000000
    10   1   1   1   1
      1.1760138988    1.0000000000
    11   1   1   1   1
      3.7606921222    1.0000000000
    12   1   1   1   1
     11.3673509998    1.0000000000
    13   1   1   1   1
     32.2288504012    1.0000000000
    14   2   2   1   1
      0.2800986434    1.0000000000
    15   2   2   1   1
      0.6324730857    1.0000000000
    16   2   2   1   1
      0.9581585746    1.0000000000
    17   3   3   1   1
      0.2576791647    1.0000000000
    18   3   3   1   1
      0.7151917455    1.0000000000
    19   3   3   1   1
      2.3399734109    1.0000000000

# RI basis set for Si (all-electron) relative DI metric: 1.0e-03
Si  RI_aug-TZVP-MOLOPT-ae_N_RI_081_s_p_d_f_g_h_i_7_6_3_3_1_1_0_error_1.0e-03
   21
     1   0   0   1   1
      0.1932446338    1.0000000000
     2   0   0   1   1
      0.3684863996    1.0000000000
     3   0   0   1   1
      1.1137758765    1.0000000000
     4   0   0   1   1
      4.3850536711    1.0000000000
     5   0   0   1   1
     17.6970692766    1.0000000000
     6   0   0   1   1
```

```
      66.4513374220    1.0000000000
      7   0   0   1   1
     242.6803413878    1.0000000000
      8   1   1   1   1
       0.2527956505    1.0000000000
      9   1   1   1   1
       0.4796783292    1.0000000000
     10   1   1   1   1
       1.1944309781    1.0000000000
     11   1   1   1   1
       4.4321715998    1.0000000000
     12   1   1   1   1
      18.0687414209    1.0000000000
     13   1   1   1   1
      66.6978884716    1.0000000000
     14   2   2   1   1
       0.2457896778    1.0000000000
     15   2   2   1   1
       0.5298644340    1.0000000000
     16   2   2   1   1
       1.2017704059    1.0000000000
     17   3   3   1   1
       0.2780543489    1.0000000000
     18   3   3   1   1
       0.7519949750    1.0000000000
     19   3   3   1   1
       3.8030536207    1.0000000000
     20   4   4   1   1
       0.9320262362    1.0000000000
     21   5   5   1   1
       0.7274774545    1.0000000000

# RI basis set for Si (all-electron) relative DI metric: 3.1e-04
Si  RI_aug-TZVP-MOLOPT-ae_N_RI_104_s_p_d_f_g_h_i_7_6_4_3_3_1_0_error_3.1e-04
   24
      1   0   0   1   1
       0.2019093195    1.0000000000
      2   0   0   1   1
       0.4076886133    1.0000000000
      3   0   0   1   1
       1.4099385723    1.0000000000
      4   0   0   1   1
       5.1034489304    1.0000000000
      5   0   0   1   1
      18.6099609911    1.0000000000
      6   0   0   1   1
      67.4302692876    1.0000000000
      7   0   0   1   1
     243.7230568055    1.0000000000
      8   1   1   1   1
       0.2265633716    1.0000000000
      9   1   1   1   1
       0.4999277990    1.0000000000
     10   1   1   1   1
       1.1096085599    1.0000000000
     11   1   1   1   1
       3.8971138669    1.0000000000
     12   1   1   1   1
      15.2326505103    1.0000000000
     13   1   1   1   1
      63.3400730891    1.0000000000
     14   2   2   1   1
       0.2840735491    1.0000000000
     15   2   2   1   1
       0.6347530395    1.0000000000
     16   2   2   1   1
       1.9557735736    1.0000000000
     17   2   2   1   1
       6.3014338454    1.0000000000
     18   3   3   1   1
       0.2505542359    1.0000000000
     19   3   3   1   1
       0.6726078078    1.0000000000
     20   3   3   1   1
       3.1602977319    1.0000000000
     21   4   4   1   1
       0.2050115993    1.0000000000
     22   4   4   1   1
       0.5336829465    1.0000000000
     23   4   4   1   1
       1.2132637505    1.0000000000
     24   5   5   1   1
       0.6001819968    1.0000000000

# RI basis set for P (all-electron) relative DI metric: 3.5e-02
P  RI_aug-SZV-MOLOPT-ae-mini_N_RI_006_s_p_d_f_g_h_i_3_1_0_0_0_0_0_error_3.5e-02
   4
      1   0   0   1   1
       0.4306640332    1.0000000000
      2   0   0   1   1
       1.2270337739    1.0000000000
      3   0   0   1   1
       4.4471937308    1.0000000000
      4   1   1   1   1
       0.6333235789    1.0000000000

# RI basis set for P (all-electron) relative DI metric: 1.7e-03
P  RI_aug-SZV-MOLOPT-ae-mini_N_RI_010_s_p_d_f_g_h_i_4_2_0_0_0_0_0_error_1.7e-03
   6
      1   0   0   1   1
       0.3524703383    1.0000000000
      2   0   0   1   1
       1.2412335881    1.0000000000
      3   0   0   1   1
       4.3709951081    1.0000000000
      4   0   0   1   1
      15.3924263565    1.0000000000
      5   1   1   1   1
       0.2632239484    1.0000000000
      6   1   1   1   1
       0.4890657101    1.0000000000
```

```
# RI basis set for P (all-electron) relative DI metric: 1.5e-05
P  RI_aug-SZV-MOLOPT-ae-mini_N_RI_015_s_p_d_f_g_h_i_4_2_1_0_0_0_0_error_1.5e-05
   7
     1   0   0   1   1
      0.4164748724    1.0000000000
     2   0   0   1   1
      0.8203116026    1.0000000000
     3   0   0   1   1
      1.6213264475    1.0000000000
     4   0   0   1   1
      3.2540156631    1.0000000000
     5   1   1   1   1
      0.2979755017    1.0000000000
     6   1   1   1   1
      0.5322486009    1.0000000000
     7   2   2   1   1
      1.6246175929    1.0000000000

# RI basis set for P (all-electron) relative DI metric: 1.6e-06
P  RI_aug-SZV-MOLOPT-ae-mini_N_RI_018_s_p_d_f_g_h_i_4_3_1_0_0_0_0_error_1.6e-06
   8
     1   0   0   1   1
      0.4089055951    1.0000000000
     2   0   0   1   1
      0.8212934363    1.0000000000
     3   0   0   1   1
      1.6455370130    1.0000000000
     4   0   0   1   1
      3.3053487165    1.0000000000
     5   1   1   1   1
      0.2826116606    1.0000000000
     6   1   1   1   1
      0.6279290881    1.0000000000
     7   1   1   1   1
      2.5951581292    1.0000000000
     8   2   2   1   1
      1.0780338463    1.0000000000

# RI basis set for P (all-electron) relative DI metric: 4.6e-07
P  RI_aug-SZV-MOLOPT-ae-mini_N_RI_029_s_p_d_f_g_h_i_5_3_3_0_0_0_0_error_4.6e-07
   11
     1   0   0   1   1
      0.2205299109    1.0000000000
     2   0   0   1   1
      0.4220977289    1.0000000000
     3   0   0   1   1
      0.8063816705    1.0000000000
     4   0   0   1   1
      1.5315248794    1.0000000000
     5   0   0   1   1
      2.9036118138    1.0000000000
     6   1   1   1   1
      0.3101107622    1.0000000000
     7   1   1   1   1
      0.6050255236    1.0000000000
     8   1   1   1   1
      2.4289279027    1.0000000000
     9   2   2   1   1
      0.2388184010    1.0000000000
    10   2   2   1   1
      0.8372292850    1.0000000000
    11   2   2   1   1
      2.9046195532    1.0000000000

# RI basis set for P (all-electron) relative DI metric: 4.1e-08
P  RI_aug-SZV-MOLOPT-ae-mini_N_RI_036_s_p_d_f_g_h_i_6_5_3_0_0_0_0_error_4.1e-08
   14
     1   0   0   1   1
      0.2249999997    1.0000000000
     2   0   0   1   1
      0.3753015640    1.0000000000
     3   0   0   1   1
      0.6260056180    1.0000000000
     4   0   0   1   1
      1.0441817229    1.0000000000
     5   0   0   1   1
      1.7417023730    1.0000000000
     6   0   0   1   1
      2.9051716665    1.0000000000
     7   1   1   1   1
      0.2250000000    1.0000000000
     8   1   1   1   1
      0.4265106093    1.0000000000
     9   1   1   1   1
      0.8084946659    1.0000000000
    10   1   1   1   1
      1.5325846783    1.0000000000
    11   1   1   1   1
      2.9051716667    1.0000000000
    12   2   2   1   1
      0.2250000032    1.0000000000
    13   2   2   1   1
      0.8084946721    1.0000000000
    14   2   2   1   1
      2.9051716666    1.0000000000

# RI basis set for P (all-electron) relative DI metric: 1.4e-08
P  RI_aug-SZV-MOLOPT-ae-mini_N_RI_042_s_p_d_f_g_h_i_7_5_4_0_0_0_0_error_1.4e-08
   16
     1   0   0   1   1
      0.2250000000    1.0000000000
     2   0   0   1   1
      0.3446254914    1.0000000000
     3   0   0   1   1
      0.5278521304    1.0000000000
     4   0   0   1   1
      0.8084946660    1.0000000000
     5   0   0   1   1
      1.2383460960    1.0000000000
     6   0   0   1   1
```

```
      1.8967361418    1.0000000000
     7   0   0   1   1
      2.9051716667    1.0000000000
     8   1   1   1   1
      0.2250000000    1.0000000000
     9   1   1   1   1
      0.4265106093    1.0000000000
    10   1   1   1   1
      0.8084946659    1.0000000000
    11   1   1   1   1
      1.5325846783    1.0000000000
    12   1   1   1   1
      2.9051716667    1.0000000000
    13   2   2   1   1
      0.2250000000    1.0000000000
    14   2   2   1   1
      0.5278521304    1.0000000000
    15   2   2   1   1
      1.2383460962    1.0000000000
    16   2   2   1   1
      2.9051716667    1.0000000000

# RI basis set for P (all-electron) relative DI metric: 2.5e-09
P  RI_aug-SZV-MOLOPT-ae-mini_N_RI_045_s_p_d_f_g_h_i_7_6_4_0_0_0_0_error_2.5e-09
   17
     1   0   0   1   1
      0.2250000000    1.0000000000
     2   0   0   1   1
      0.3446254914    1.0000000000
     3   0   0   1   1
      0.5278521304    1.0000000000
     4   0   0   1   1
      0.8084946660    1.0000000000
     5   0   0   1   1
      1.2383460960    1.0000000000
     6   0   0   1   1
      1.8967361418    1.0000000000
     7   0   0   1   1
      2.9051716667    1.0000000000
     8   1   1   1   1
      0.2250000000    1.0000000000
     9   1   1   1   1
      0.3753015642    1.0000000000
    10   1   1   1   1
      0.6260056182    1.0000000000
    11   1   1   1   1
      1.0441817230    1.0000000000
    12   1   1   1   1
      1.7417023732    1.0000000000
    13   1   1   1   1
      2.9051716667    1.0000000000
    14   2   2   1   1
      0.2250000000    1.0000000000
    15   2   2   1   1
      0.5278521304    1.0000000000
    16   2   2   1   1
      1.2383460962    1.0000000000
    17   2   2   1   1
      2.9051716667    1.0000000000

# RI basis set for P (all-electron) relative DI metric: 1.1e-02
P  RI_aug-SZV-MOLOPT-ae_N_RI_022_s_p_d_f_g_h_i_7_1_1_1_0_0_0_error_1.1e-02
   10
     1   0   0   1   1
      0.3102846221    1.0000000000
     2   0   0   1   1
      0.9675410747    1.0000000000
     3   0   0   1   1
      2.9475660172    1.0000000000
     4   0   0   1   1
      9.3026367371    1.0000000000
     5   0   0   1   1
     29.5859283114    1.0000000000
     6   0   0   1   1
     94.2680229490    1.0000000000
     7   0   0   1   1
    300.6106628184    1.0000000000
     8   1   1   1   1
      0.5077267489    1.0000000000
     9   2   2   1   1
      0.7099333158    1.0000000000
    10   3   3   1   1
      1.2789537381    1.0000000000

# RI basis set for P (all-electron) relative DI metric: 5.4e-04
P  RI_aug-SZV-MOLOPT-ae_N_RI_037_s_p_d_f_g_h_i_7_6_1_1_0_0_0_error_5.4e-04
   15
     1   0   0   1   1
      0.3371263690    1.0000000000
     2   0   0   1   1
      0.7577066360    1.0000000000
     3   0   0   1   1
      2.0166803737    1.0000000000
     4   0   0   1   1
      8.8851947761    1.0000000000
     5   0   0   1   1
     29.6614514765    1.0000000000
     6   0   0   1   1
     94.4262494857    1.0000000000
     7   0   0   1   1
    300.7677618649    1.0000000000
     8   1   1   1   1
      0.3636288512    1.0000000000
     9   1   1   1   1
      0.7207198899    1.0000000000
    10   1   1   1   1
      2.7498054758    1.0000000000
    11   1   1   1   1
      8.8425156467    1.0000000000
    12   1   1   1   1
     27.1070518100    1.0000000000
```

```
    13   1   1   1   1
     82.3519581211    1.0000000000
    14   2   2   1   1
      0.5430634091    1.0000000000
    15   3   3   1   1
      0.5399090909    1.0000000000

# RI basis set for P (all-electron) relative DI metric: 2.2e-04
P  RI_aug-SZV-MOLOPT-ae_N_RI_051_s_p_d_f_g_h_i_7_6_2_1_1_0_0_error_2.2e-04
   17
     1   0   0   1   1
      0.2962352317    1.0000000000
     2   0   0   1   1
      0.6994557900    1.0000000000
     3   0   0   1   1
      2.3674234334    1.0000000000
     4   0   0   1   1
      9.0061396313    1.0000000000
     5   0   0   1   1
     29.5056851693    1.0000000000
     6   0   0   1   1
     94.2391953185    1.0000000000
     7   0   0   1   1
    300.5337040869    1.0000000000
     8   1   1   1   1
      0.3148833105    1.0000000000
     9   1   1   1   1
      0.5931565946    1.0000000000
    10   1   1   1   1
      1.8044537155    1.0000000000
    11   1   1   1   1
      6.5654281595    1.0000000000
    12   1   1   1   1
     23.3974488380    1.0000000000
    13   1   1   1   1
     82.5065747441    1.0000000000
    14   2   2   1   1
      0.4954813083    1.0000000000
    15   2   2   1   1
     21.2079502266    1.0000000000
    16   3   3   1   1
      0.5722434219    1.0000000000
    17   4   4   1   1
      1.7206756859    1.0000000000

# RI basis set for P (all-electron) relative DI metric: 6.8e-05
P  RI_aug-SZV-MOLOPT-ae_N_RI_067_s_p_d_f_g_h_i_7_6_3_1_1_1_0_error_6.8e-05
   19
     1   0   0   1   1
      0.2197408869    1.0000000000
     2   0   0   1   1
      0.7440245829    1.0000000000
     3   0   0   1   1
      2.7171255840    1.0000000000
     4   0   0   1   1
      9.0890470561    1.0000000000
     5   0   0   1   1
     29.3666457485    1.0000000000
     6   0   0   1   1
     94.0251019769    1.0000000000
     7   0   0   1   1
    300.3596530679    1.0000000000
     8   1   1   1   1
      0.3002370034    1.0000000000
     9   1   1   1   1
      0.9082387333    1.0000000000
    10   1   1   1   1
      2.9182626764    1.0000000000
    11   1   1   1   1
      8.9505541889    1.0000000000
    12   1   1   1   1
     27.1986848528    1.0000000000
    13   1   1   1   1
     82.5119915559    1.0000000000
    14   2   2   1   1
      0.4317496326    1.0000000000
    15   2   2   1   1
      1.2400854978    1.0000000000
    16   2   2   1   1
     22.3913339292    1.0000000000
    17   3   3   1   1
      0.5154582526    1.0000000000
    18   4   4   1   1
      1.0713072883    1.0000000000
    19   5   5   1   1
      0.8078459226    1.0000000000

# RI basis set for P (all-electron) relative DI metric: 2.7e-05
P  RI_aug-SZV-MOLOPT-ae_N_RI_079_s_p_d_f_g_h_i_7_6_4_2_1_1_0_error_2.7e-05
   21
     1   0   0   1   1
      0.2722064555    1.0000000000
     2   0   0   1   1
      0.5330315885    1.0000000000
     3   0   0   1   1
      1.7624898454    1.0000000000
     4   0   0   1   1
      6.1725109615    1.0000000000
     5   0   0   1   1
     22.6863439704    1.0000000000
     6   0   0   1   1
     82.7589980581    1.0000000000
     7   0   0   1   1
    300.3229779979    1.0000000000
     8   1   1   1   1
      0.2467145205    1.0000000000
     9   1   1   1   1
      0.7964917146    1.0000000000
    10   1   1   1   1
      2.8491072865    1.0000000000
    11   1   1   1   1
```

```
      8.8964133181    1.0000000000
    12   1   1   1   1
     27.1164637044    1.0000000000
    13   1   1   1   1
     82.3138801135    1.0000000000
    14   2   2   1   1
      0.4334086448    1.0000000000
    15   2   2   1   1
      1.0820513620    1.0000000000
    16   2   2   1   1
      5.1626304606    1.0000000000
    17   2   2   1   1
     24.2358941350    1.0000000000
    18   3   3   1   1
      0.4510023851    1.0000000000
    19   3   3   1   1
      3.2723676238    1.0000000000
    20   4   4   1   1
      1.0951498170    1.0000000000
    21   5   5   1   1
      1.3418205458    1.0000000000

# RI basis set for P (all-electron) relative DI metric: 5.9e-06
P  RI_aug-SZV-MOLOPT-ae_N_RI_091_s_p_d_f_g_h_i_7_6_5_3_1_1_0_error_5.9e-06
   23
     1   0   0   1   1
      0.2708480810    1.0000000000
     2   0   0   1   1
      0.3673400320    1.0000000000
     3   0   0   1   1
      1.4165168675    1.0000000000
     4   0   0   1   1
      6.3254704755    1.0000000000
     5   0   0   1   1
     24.2562734210    1.0000000000
     6   0   0   1   1
     85.9946334657    1.0000000000
     7   0   0   1   1
    297.2473311173    1.0000000000
     8   1   1   1   1
      0.2577333413    1.0000000000
     9   1   1   1   1
      0.5734554388    1.0000000000
    10   1   1   1   1
      1.8720767480    1.0000000000
    11   1   1   1   1
      6.5295281435    1.0000000000
    12   1   1   1   1
     23.3648118815    1.0000000000
    13   1   1   1   1
     82.4840618238    1.0000000000
    14   2   2   1   1
      0.3483808696    1.0000000000
    15   2   2   1   1
      0.7562074793    1.0000000000
    16   2   2   1   1
      2.8381768055    1.0000000000
    17   2   2   1   1
      8.4467792286    1.0000000000
    18   2   2   1   1
     24.6073427387    1.0000000000
    19   3   3   1   1
      0.3901840019    1.0000000000
    20   3   3   1   1
      1.1697220174    1.0000000000
    21   3   3   1   1
      5.2125652387    1.0000000000
    22   4   4   1   1
      1.4194706962    1.0000000000
    23   5   5   1   1
      1.1074917370    1.0000000000

# RI basis set for P (all-electron) relative DI metric: 4.5e-02
P  RI_aug-DZVP-MOLOPT-ae_N_RI_018_s_p_d_f_g_h_i_3_1_1_1_0_0_0_error_4.5e-02
   6
     1   0   0   1   1
      1.9700052162    1.0000000000
     2   0   0   1   1
      3.8905251822    1.0000000000
     3   0   0   1   1
    290.5841706215    1.0000000000
     4   1   1   1   1
      0.4900580707    1.0000000000
     5   2   2   1   1
      0.6873684449    1.0000000000
     6   3   3   1   1
      0.7614069144    1.0000000000

# RI basis set for P (all-electron) relative DI metric: 1.5e-02
P  RI_aug-DZVP-MOLOPT-ae_N_RI_029_s_p_d_f_g_h_i_5_4_1_1_0_0_0_error_1.5e-02
   11
     1   0   0   1   1
      0.4492199289    1.0000000000
     2   0   0   1   1
      0.5861969845    1.0000000000
     3   0   0   1   1
      4.1488976967    1.0000000000
     4   0   0   1   1
     38.6233757642    1.0000000000
     5   0   0   1   1
    294.5713524550    1.0000000000
     6   1   1   1   1
      0.3289647724    1.0000000000
     7   1   1   1   1
      1.4936551495    1.0000000000
     8   1   1   1   1
     12.4151127152    1.0000000000
     9   1   1   1   1
     73.3698288365    1.0000000000
    10   2   2   1   1
      0.5898613108    1.0000000000
```

```
    11   3   3   1   1
      0.7459846397    1.0000000000

# RI basis set for P (all-electron) relative DI metric: 5.3e-03
P  RI_aug-DZVP-MOLOPT-ae_N_RI_050_s_p_d_f_g_h_i_6_5_3_2_0_0_0_error_5.3e-03
   16
     1   0   0   1   1
      0.3064438112    1.0000000000
     2   0   0   1   1
      0.6192778964    1.0000000000
     3   0   0   1   1
      2.4571678873    1.0000000000
     4   0   0   1   1
     12.8086519282    1.0000000000
     5   0   0   1   1
     63.1282009605    1.0000000000
     6   0   0   1   1
    299.4311408744    1.0000000000
     7   1   1   1   1
      0.3194813769    1.0000000000
     8   1   1   1   1
      1.0092042832    1.0000000000
     9   1   1   1   1
      5.1030245013    1.0000000000
    10   1   1   1   1
     20.6569909490    1.0000000000
    11   1   1   1   1
     79.5498182708    1.0000000000
    12   2   2   1   1
      0.7502124259    1.0000000000
    13   2   2   1   1
      1.2272801009    1.0000000000
    14   2   2   1   1
     23.8344248027    1.0000000000
    15   3   3   1   1
      0.5535225931    1.0000000000
    16   3   3   1   1
      4.0410788412    1.0000000000

# RI basis set for P (all-electron) relative DI metric: 1.2e-03
P  RI_aug-DZVP-MOLOPT-ae_N_RI_064_s_p_d_f_g_h_i_6_5_4_2_1_0_0_error_1.2e-03
   18
     1   0   0   1   1
      0.2759097431    1.0000000000
     2   0   0   1   1
      0.5933609998    1.0000000000
     3   0   0   1   1
      2.4803665668    1.0000000000
     4   0   0   1   1
     12.9230061786    1.0000000000
     5   0   0   1   1
     63.2713782271    1.0000000000
     6   0   0   1   1
    299.5899260175    1.0000000000
     7   1   1   1   1
      0.2738676172    1.0000000000
     8   1   1   1   1
      0.9148520528    1.0000000000
     9   1   1   1   1
      4.9359409442    1.0000000000
    10   1   1   1   1
     20.3914825302    1.0000000000
    11   1   1   1   1
     78.8915576114    1.0000000000
    12   2   2   1   1
      0.4245049967    1.0000000000
    13   2   2   1   1
      0.8544460219    1.0000000000
    14   2   2   1   1
      4.6055029616    1.0000000000
    15   2   2   1   1
     24.7162347519    1.0000000000
    16   3   3   1   1
      0.5112589718    1.0000000000
    17   3   3   1   1
      3.4844136453    1.0000000000
    18   4   4   1   1
      0.7921155406    1.0000000000

# RI basis set for P (all-electron) relative DI metric: 5.5e-04
P  RI_aug-DZVP-MOLOPT-ae_N_RI_068_s_p_d_f_g_h_i_7_6_4_2_1_0_0_error_5.5e-04
   20
     1   0   0   1   1
      0.2109943231    1.0000000000
     2   0   0   1   1
      0.5472524153    1.0000000000
     3   0   0   1   1
      1.2702086517    1.0000000000
     4   0   0   1   1
      6.3055407364    1.0000000000
     5   0   0   1   1
     23.2608212559    1.0000000000
     6   0   0   1   1
     83.1798562644    1.0000000000
     7   0   0   1   1
    295.4461943475    1.0000000000
     8   1   1   1   1
      0.4390017339    1.0000000000
     9   1   1   1   1
      0.6126873028    1.0000000000
    10   1   1   1   1
      2.9128857884    1.0000000000
    11   1   1   1   1
      9.1687181817    1.0000000000
    12   1   1   1   1
     27.1930276327    1.0000000000
    13   1   1   1   1
     82.0967480722    1.0000000000
    14   2   2   1   1
      0.4713902313    1.0000000000
    15   2   2   1   1
```

```
      0.5775175693    1.0000000000
    16   2   2   1   1
      4.4330595639    1.0000000000
    17   2   2   1   1
     24.6236275721    1.0000000000
    18   3   3   1   1
      0.6860747132    1.0000000000
    19   3   3   1   1
      1.4022379761    1.0000000000
    20   4   4   1   1
      0.5343303195    1.0000000000

# RI basis set for P (all-electron) relative DI metric: 1.2e-04
P  RI_aug-DZVP-MOLOPT-ae_N_RI_075_s_p_d_f_g_h_i_7_6_4_3_1_0_0_error_1.2e-04
   21
     1   0   0   1   1
      0.2045841841    1.0000000000
     2   0   0   1   1
      0.3607074005    1.0000000000
     3   0   0   1   1
      1.2496291842    1.0000000000
     4   0   0   1   1
      5.0605571846    1.0000000000
     5   0   0   1   1
     21.3288206662    1.0000000000
     6   0   0   1   1
     81.3220945954    1.0000000000
     7   0   0   1   1
    298.7966763406    1.0000000000
     8   1   1   1   1
      0.2807689202    1.0000000000
     9   1   1   1   1
      0.4306877397    1.0000000000
    10   1   1   1   1
      1.2384627039    1.0000000000
    11   1   1   1   1
      5.7901285130    1.0000000000
    12   1   1   1   1
     23.0480858949    1.0000000000
    13   1   1   1   1
     80.4557418551    1.0000000000
    14   2   2   1   1
      0.2367858329    1.0000000000
    15   2   2   1   1
      0.5870215294    1.0000000000
    16   2   2   1   1
      1.7967891250    1.0000000000
    17   2   2   1   1
     18.0105296761    1.0000000000
    18   3   3   1   1
      0.3118925812    1.0000000000
    19   3   3   1   1
      0.8590097519    1.0000000000
    20   3   3   1   1
      4.9943100858    1.0000000000
    21   4   4   1   1
      0.6369322866    1.0000000000

# RI basis set for P (all-electron) relative DI metric: 5.3e-05
P  RI_aug-DZVP-MOLOPT-ae_N_RI_095_s_p_d_f_g_h_i_7_6_4_3_2_1_0_error_5.3e-05
   23
     1   0   0   1   1
      0.2713398591    1.0000000000
     2   0   0   1   1
      0.3362011366    1.0000000000
     3   0   0   1   1
      1.2426321264    1.0000000000
     4   0   0   1   1
      5.2994240979    1.0000000000
     5   0   0   1   1
     21.7109324551    1.0000000000
     6   0   0   1   1
     81.7189649755    1.0000000000
     7   0   0   1   1
    299.2130197582    1.0000000000
     8   1   1   1   1
      0.3183959762    1.0000000000
     9   1   1   1   1
      0.5419932500    1.0000000000
    10   1   1   1   1
      1.3485324863    1.0000000000
    11   1   1   1   1
      5.7391268760    1.0000000000
    12   1   1   1   1
     22.6972977247    1.0000000000
    13   1   1   1   1
     81.8804154698    1.0000000000
    14   2   2   1   1
      0.3143426826    1.0000000000
    15   2   2   1   1
      0.7411445883    1.0000000000
    16   2   2   1   1
      4.4447548765    1.0000000000
    17   2   2   1   1
     24.6186440571    1.0000000000
    18   3   3   1   1
      0.4174138969    1.0000000000
    19   3   3   1   1
      0.8680622566    1.0000000000
    20   3   3   1   1
      4.7839181596    1.0000000000
    21   4   4   1   1
      0.4478030052    1.0000000000
    22   4   4   1   1
      1.4387705264    1.0000000000
    23   5   5   1   1
      1.0634773833    1.0000000000

# RI basis set for P (all-electron) relative DI metric: 2.4e-05
P  RI_aug-DZVP-MOLOPT-ae_N_RI_105_s_p_d_f_g_h_i_7_6_6_3_2_1_0_error_2.4e-05
   25
```

```
    1   0   0   1   1
      0.2240734895    1.0000000000
    2   0   0   1   1
      0.3417346478    1.0000000000
    3   0   0   1   1
      0.9983353719    1.0000000000
    4   0   0   1   1
      3.8197603752    1.0000000000
    5   0   0   1   1
     18.9185207440    1.0000000000
    6   0   0   1   1
     78.5400726494    1.0000000000
    7   0   0   1   1
    288.5616095378    1.0000000000
    8   1   1   1   1
      0.2932232786    1.0000000000
    9   1   1   1   1
      0.6724744461    1.0000000000
   10   1   1   1   1
      2.7069957050    1.0000000000
   11   1   1   1   1
      8.6010203193    1.0000000000
   12   1   1   1   1
     25.1355642925    1.0000000000
   13   1   1   1   1
     72.4287215340    1.0000000000
   14   2   2   1   1
      0.2999984979    1.0000000000
   15   2   2   1   1
      0.5584581345    1.0000000000
   16   2   2   1   1
      1.4738684921    1.0000000000
   17   2   2   1   1
      4.0019090776    1.0000000000
   18   2   2   1   1
     10.0312331167    1.0000000000
   19   2   2   1   1
     24.6091721752    1.0000000000
   20   3   3   1   1
      0.2784184036    1.0000000000
   21   3   3   1   1
      0.7364388245    1.0000000000
   22   3   3   1   1
      2.4068123809    1.0000000000
   23   4   4   1   1
      0.4577659823    1.0000000000
   24   4   4   1   1
      1.0837024305    1.0000000000
   25   5   5   1   1
      0.1921287791    1.0000000000

# RI basis set for P (all-electron) relative DI metric: 2.0e-02
P  RI_aug-TZVP-MOLOPT-ae_N_RI_034_s_p_d_f_g_h_i_5_4_2_1_0_0_0_error_2.0e-02
   12
    1   0   0   1   1
      0.2598650628    1.0000000000
    2   0   0   1   1
      0.6782697320    1.0000000000
    3   0   0   1   1
      4.8439243576    1.0000000000
    4   0   0   1   1
     41.2012633846    1.0000000000
    5   0   0   1   1
    297.7836280086    1.0000000000
    6   1   1   1   1
      0.2647900236    1.0000000000
    7   1   1   1   1
      0.4753481845    1.0000000000
    8   1   1   1   1
      2.8242299398    1.0000000000
    9   1   1   1   1
     39.6229818582    1.0000000000
   10   2   2   1   1
      0.5380295627    1.0000000000
   11   2   2   1   1
      1.4657155422    1.0000000000
   12   3   3   1   1
      0.8459724215    1.0000000000

# RI basis set for P (all-electron) relative DI metric: 7.8e-03
P  RI_aug-TZVP-MOLOPT-ae_N_RI_055_s_p_d_f_g_h_i_7_4_4_1_1_0_0_error_7.8e-03
   17
    1   0   0   1   1
      0.2670533635    1.0000000000
    2   0   0   1   1
      0.5214880505    1.0000000000
    3   0   0   1   1
      1.5808089782    1.0000000000
    4   0   0   1   1
      5.9046548409    1.0000000000
    5   0   0   1   1
     22.3867497296    1.0000000000
    6   0   0   1   1
     82.4262896442    1.0000000000
    7   0   0   1   1
    299.9653225287    1.0000000000
    8   1   1   1   1
      0.2600207516    1.0000000000
    9   1   1   1   1
      0.9252664964    1.0000000000
   10   1   1   1   1
      6.4277784675    1.0000000000
   11   1   1   1   1
     37.8961398399    1.0000000000
   12   2   2   1   1
      0.4716003478    1.0000000000
   13   2   2   1   1
      0.9235522093    1.0000000000
   14   2   2   1   1
      3.7537662470    1.0000000000
   15   2   2   1   1
```

```
     24.0042072060    1.0000000000
    16   3   3   1   1
      0.6971855550    1.0000000000
    17   4   4   1   1
      0.7964839133    1.0000000000

# RI basis set for P (all-electron) relative DI metric: 3.7e-03
P  RI_aug-TZVP-MOLOPT-ae_N_RI_061_s_p_d_f_g_h_i_7_6_4_1_1_0_0_error_3.7e-03
   19
     1   0   0   1   1
      0.2827429487    1.0000000000
     2   0   0   1   1
      0.5430582866    1.0000000000
     3   0   0   1   1
      1.3351722654    1.0000000000
     4   0   0   1   1
      5.2105073450    1.0000000000
     5   0   0   1   1
     21.4024463759    1.0000000000
     6   0   0   1   1
     81.3702644399    1.0000000000
     7   0   0   1   1
    298.8453598462    1.0000000000
     8   1   1   1   1
      0.3100044190    1.0000000000
     9   1   1   1   1
      0.4573387737    1.0000000000
    10   1   1   1   1
      1.4889004356    1.0000000000
    11   1   1   1   1
      5.2900178899    1.0000000000
    12   1   1   1   1
     21.4979377558    1.0000000000
    13   1   1   1   1
     80.7122890876    1.0000000000
    14   2   2   1   1
      0.4544214286    1.0000000000
    15   2   2   1   1
      1.0521660577    1.0000000000
    16   2   2   1   1
      4.0855934258    1.0000000000
    17   2   2   1   1
     14.6001978907    1.0000000000
    18   3   3   1   1
      0.6916691453    1.0000000000
    19   4   4   1   1
      0.5225410505    1.0000000000

# RI basis set for P (all-electron) relative DI metric: 1.5e-03
P  RI_aug-TZVP-MOLOPT-ae_N_RI_075_s_p_d_f_g_h_i_7_6_4_3_1_0_0_error_1.5e-03
   21
     1   0   0   1   1
      0.2839389172    1.0000000000
     2   0   0   1   1
      0.5754192689    1.0000000000
     3   0   0   1   1
      1.3305535986    1.0000000000
     4   0   0   1   1
      4.9931140468    1.0000000000
     5   0   0   1   1
     20.8870866541    1.0000000000
     6   0   0   1   1
     80.8144928998    1.0000000000
     7   0   0   1   1
    298.2643600152    1.0000000000
     8   1   1   1   1
      0.3729048865    1.0000000000
     9   1   1   1   1
      0.5274378927    1.0000000000
    10   1   1   1   1
      1.6814181073    1.0000000000
    11   1   1   1   1
      6.6247653148    1.0000000000
    12   1   1   1   1
     23.2392700529    1.0000000000
    13   1   1   1   1
     75.1113874965    1.0000000000
    14   2   2   1   1
      0.3850336407    1.0000000000
    15   2   2   1   1
      0.8555628157    1.0000000000
    16   2   2   1   1
      2.5763840810    1.0000000000
    17   2   2   1   1
      8.0494517743    1.0000000000
    18   3   3   1   1
      0.6287451160    1.0000000000
    19   3   3   1   1
      0.9641593911    1.0000000000
    20   3   3   1   1
      4.3066450604    1.0000000000
    21   4   4   1   1
      0.7145775777    1.0000000000

# RI basis set for P (all-electron) relative DI metric: 6.9e-04
P  RI_aug-TZVP-MOLOPT-ae_N_RI_086_s_p_d_f_g_h_i_7_6_4_3_1_1_0_error_6.9e-04
   22
     1   0   0   1   1
      0.2823726969    1.0000000000
     2   0   0   1   1
      0.5182483798    1.0000000000
     3   0   0   1   1
      1.2736836026    1.0000000000
     4   0   0   1   1
      4.9179278284    1.0000000000
     5   0   0   1   1
     20.7877672967    1.0000000000
     6   0   0   1   1
     80.7053122926    1.0000000000
     7   0   0   1   1
    298.1476102107    1.0000000000
```

```
      8   1   1   1   1
       0.3206395323    1.0000000000
      9   1   1   1   1
       0.6443270811    1.0000000000
     10   1   1   1   1
       1.3769178248    1.0000000000
     11   1   1   1   1
       4.9671890581    1.0000000000
     12   1   1   1   1
      20.7972274054    1.0000000000
     13   1   1   1   1
      80.0600532776    1.0000000000
     14   2   2   1   1
       0.3917133195    1.0000000000
     15   2   2   1   1
       0.7879215520    1.0000000000
     16   2   2   1   1
       2.5166765118    1.0000000000
     17   2   2   1   1
       9.0692756826    1.0000000000
     18   3   3   1   1
       0.4785837383    1.0000000000
     19   3   3   1   1
       0.8560155663    1.0000000000
     20   3   3   1   1
       3.3648477779    1.0000000000
     21   4   4   1   1
       0.6463871661    1.0000000000
     22   5   5   1   1
       0.7900005992    1.0000000000

# RI basis set for P (all-electron) relative DI metric: 3.3e-04
P  RI_aug-TZVP-MOLOPT-ae_N_RI_111_s_p_d_f_g_h_i_7_6_4_4_3_1_0_error_3.3e-04
   25
      1   0   0   1   1
       0.2940933105    1.0000000000
      2   0   0   1   1
       0.6891077327    1.0000000000
      3   0   0   1   1
       2.2258757634    1.0000000000
      4   0   0   1   1
       8.2784063698    1.0000000000
      5   0   0   1   1
      28.4765575526    1.0000000000
      6   0   0   1   1
      93.0843242341    1.0000000000
      7   0   0   1   1
     299.4055772662    1.0000000000
      8   1   1   1   1
       0.3916659249    1.0000000000
      9   1   1   1   1
       0.6199324998    1.0000000000
     10   1   1   1   1
       2.0405579260    1.0000000000
     11   1   1   1   1
       7.6097067319    1.0000000000
     12   1   1   1   1
      24.9298423220    1.0000000000
     13   1   1   1   1
      77.1074085948    1.0000000000
     14   2   2   1   1
       0.4368143967    1.0000000000
     15   2   2   1   1
       1.0533348969    1.0000000000
     16   2   2   1   1
       2.5101614373    1.0000000000
     17   2   2   1   1
      10.4918561970    1.0000000000
     18   3   3   1   1
       0.2934535509    1.0000000000
     19   3   3   1   1
       0.6223531171    1.0000000000
     20   3   3   1   1
       1.6013692899    1.0000000000
     21   3   3   1   1
       4.1306007499    1.0000000000
     22   4   4   1   1
       0.2305991012    1.0000000000
     23   4   4   1   1
       0.6068749792    1.0000000000
     24   4   4   1   1
       1.3361460073    1.0000000000
     25   5   5   1   1
       0.3576840752    1.0000000000

# RI basis set for S (all-electron) relative DI metric: 3.1e-02
S  RI_aug-SZV-MOLOPT-ae-mini_N_RI_013_s_p_d_f_g_h_i_4_3_0_0_0_0_0_error_3.1e-02
   7
      1   0   0   1   1
       0.3917585998    1.0000000000
      2   0   0   1   1
       1.0414841652    1.0000000000
      3   0   0   1   1
       2.7687691639    1.0000000000
      4   0   0   1   1
       7.3607288363    1.0000000000
      5   1   1   1   1
       0.4109529966    1.0000000000
      6   1   1   1   1
       0.7522178552    1.0000000000
      7   1   1   1   1
       2.1455282027    1.0000000000

# RI basis set for S (all-electron) relative DI metric: 4.3e-03
S  RI_aug-SZV-MOLOPT-ae-mini_N_RI_018_s_p_d_f_g_h_i_4_3_1_0_0_0_0_error_4.3e-03
   8
      1   0   0   1   1
       0.4446608996    1.0000000000
      2   0   0   1   1
       0.7234808544    1.0000000000
      3   0   0   1   1
```

```
      1.2112135158    1.0000000000
     4   0   0   1   1
      2.0984873770    1.0000000000
     5   1   1   1   1
      0.3594012410    1.0000000000
     6   1   1   1   1
      0.8166996637    1.0000000000
     7   1   1   1   1
      2.8410978191    1.0000000000
     8   2   2   1   1
      1.8558999609    1.0000000000

# RI basis set for S (all-electron) relative DI metric: 3.2e-06
S  RI_aug-SZV-MOLOPT-ae-mini_N_RI_024_s_p_d_f_g_h_i_5_3_2_0_0_0_0_error_3.2e-06
   10
     1   0   0   1   1
      0.2239052376    1.0000000000
     2   0   0   1   1
      0.4589446100    1.0000000000
     3   0   0   1   1
      0.9203474234    1.0000000000
     4   0   0   1   1
      1.7672851701    1.0000000000
     5   0   0   1   1
      3.3751787111    1.0000000000
     6   1   1   1   1
      0.3961473500    1.0000000000
     7   1   1   1   1
      0.9697219465    1.0000000000
     8   1   1   1   1
      1.9533554439    1.0000000000
     9   2   2   1   1
      0.2430103510    1.0000000000
    10   2   2   1   1
      0.9718065294    1.0000000000

# RI basis set for S (all-electron) relative DI metric: 1.6e-06
S  RI_aug-SZV-MOLOPT-ae-mini_N_RI_031_s_p_d_f_g_h_i_7_3_3_0_0_0_0_error_1.6e-06
   13
     1   0   0   1   1
      0.2483652725    1.0000000000
     2   0   0   1   1
      0.3849180524    1.0000000000
     3   0   0   1   1
      0.5947003460    1.0000000000
     4   0   0   1   1
      0.9180989442    1.0000000000
     5   0   0   1   1
      1.4184616314    1.0000000000
     6   0   0   1   1
      2.1912041719    1.0000000000
     7   0   0   1   1
      3.3825850011    1.0000000000
     8   1   1   1   1
      0.4455959843    1.0000000000
     9   1   1   1   1
      0.7608778638    1.0000000000
    10   1   1   1   1
      2.7005672217    1.0000000000
    11   2   2   1   1
      0.2323356313    1.0000000000
    12   2   2   1   1
      0.9538986362    1.0000000000
    13   2   2   1   1
      3.3587056559    1.0000000000

# RI basis set for S (all-electron) relative DI metric: 1.5e-07
S  RI_aug-SZV-MOLOPT-ae-mini_N_RI_037_s_p_d_f_g_h_i_7_5_3_0_0_0_0_error_1.5e-07
   15
     1   0   0   1   1
      0.2499999999    1.0000000000
     2   0   0   1   1
      0.3859035085    1.0000000000
     3   0   0   1   1
      0.5956860715    1.0000000000
     4   0   0   1   1
      0.9195093800    1.0000000000
     5   0   0   1   1
      1.4193675835    1.0000000000
     6   0   0   1   1
      2.1909557215    1.0000000000
     7   0   0   1   1
      3.3819900000    1.0000000000
     8   1   1   1   1
      0.2500000000    1.0000000000
     9   1   1   1   1
      0.4794552586    1.0000000000
    10   1   1   1   1
      0.9195093800    1.0000000000
    11   1   1   1   1
      1.7634544304    1.0000000000
    12   1   1   1   1
      3.3819900000    1.0000000000
    13   2   2   1   1
      0.2499999913    1.0000000000
    14   2   2   1   1
      0.9195093664    1.0000000000
    15   2   2   1   1
      3.3819900000    1.0000000000

# RI basis set for S (all-electron) relative DI metric: 5.1e-08
S  RI_aug-SZV-MOLOPT-ae-mini_N_RI_042_s_p_d_f_g_h_i_7_5_4_0_0_0_0_error_5.1e-08
   16
     1   0   0   1   1
      0.2499999999    1.0000000000
     2   0   0   1   1
      0.3859035085    1.0000000000
     3   0   0   1   1
      0.5956860715    1.0000000000
     4   0   0   1   1
      0.9195093800    1.0000000000
```

```
     5   0   0   1   1
      1.4193675835    1.0000000000
     6   0   0   1   1
      2.1909557215    1.0000000000
     7   0   0   1   1
      3.3819899999    1.0000000000
     8   1   1   1   1
      0.2500000000    1.0000000000
     9   1   1   1   1
      0.4794552586    1.0000000000
    10   1   1   1   1
      0.9195093800    1.0000000000
    11   1   1   1   1
      1.7634544304    1.0000000000
    12   1   1   1   1
      3.3819900000    1.0000000000
    13   2   2   1   1
      0.2499999998    1.0000000000
    14   2   2   1   1
      0.5956860718    1.0000000000
    15   2   2   1   1
      1.4193675828    1.0000000000
    16   2   2   1   1
      3.3819899997    1.0000000000

# RI basis set for S (all-electron) relative DI metric: 1.5e-02
S  RI_aug-SZV-MOLOPT-ae_N_RI_021_s_p_d_f_g_h_i_6_1_1_1_0_0_0_error_1.5e-02
   9
     1   0   0   1   1
      0.2502961293    1.0000000000
     2   0   0   1   1
      0.8294520829    1.0000000000
     3   0   0   1   1
      4.3967791552    1.0000000000
     4   0   0   1   1
     21.5942027240    1.0000000000
     5   0   0   1   1
     89.0567750777    1.0000000000
     6   0   0   1   1
    349.8069835052    1.0000000000
     7   1   1   1   1
      0.5547921887    1.0000000000
     8   2   2   1   1
      0.7535914967    1.0000000000
     9   3   3   1   1
      0.4410414853    1.0000000000

# RI basis set for S (all-electron) relative DI metric: 2.6e-03
S  RI_aug-SZV-MOLOPT-ae_N_RI_031_s_p_d_f_g_h_i_7_4_1_1_0_0_0_error_2.6e-03
   13
     1   0   0   1   1
      0.3184052430    1.0000000000
     2   0   0   1   1
      0.6227890368    1.0000000000
     3   0   0   1   1
      2.0507484034    1.0000000000
     4   0   0   1   1
      7.4322997590    1.0000000000
     5   0   0   1   1
     26.9864329998    1.0000000000
     6   0   0   1   1
     97.7234521039    1.0000000000
     7   0   0   1   1
    353.6745443749    1.0000000000
     8   1   1   1   1
      0.2552186435    1.0000000000
     9   1   1   1   1
      0.8097592942    1.0000000000
    10   1   1   1   1
     10.8828523927    1.0000000000
    11   1   1   1   1
     92.1212069644    1.0000000000
    12   2   2   1   1
      0.6025217860    1.0000000000
    13   3   3   1   1
      0.7022586239    1.0000000000

# RI basis set for S (all-electron) relative DI metric: 8.0e-04
S  RI_aug-SZV-MOLOPT-ae_N_RI_041_s_p_d_f_g_h_i_7_4_3_1_0_0_0_error_8.0e-04
   15
     1   0   0   1   1
      0.2827137142    1.0000000000
     2   0   0   1   1
      0.5249871744    1.0000000000
     3   0   0   1   1
      1.9858035689    1.0000000000
     4   0   0   1   1
      7.4141807883    1.0000000000
     5   0   0   1   1
     26.9650379678    1.0000000000
     6   0   0   1   1
     97.7005835647    1.0000000000
     7   0   0   1   1
    353.6502553840    1.0000000000
     8   1   1   1   1
      0.3260435784    1.0000000000
     9   1   1   1   1
      0.5242741843    1.0000000000
    10   1   1   1   1
      9.3458727712    1.0000000000
    11   1   1   1   1
     84.4291955793    1.0000000000
    12   2   2   1   1
      0.4962844892    1.0000000000
    13   2   2   1   1
      1.8772207334    1.0000000000
    14   2   2   1   1
     23.5845458051    1.0000000000
    15   3   3   1   1
      0.6832682757    1.0000000000
```

```
# RI basis set for S (all-electron) relative DI metric: 1.9e-04
S  RI_aug-SZV-MOLOPT-ae_N_RI_072_s_p_d_f_g_h_i_7_6_3_2_2_0_0_error_1.9e-04
   20
     1   0   0   1   1
      0.3416781572    1.0000000000
     2   0   0   1   1
      1.0505565040    1.0000000000
     3   0   0   1   1
      3.3986434923    1.0000000000
     4   0   0   1   1
     10.9290937876    1.0000000000
     5   0   0   1   1
     34.8794487702    1.0000000000
     6   0   0   1   1
    111.0919432647    1.0000000000
     7   0   0   1   1
    353.6420598725    1.0000000000
     8   1   1   1   1
      0.2943149269    1.0000000000
     9   1   1   1   1
      0.6887347875    1.0000000000
    10   1   1   1   1
      2.6900825634    1.0000000000
    11   1   1   1   1
      9.1025474373    1.0000000000
    12   1   1   1   1
     30.3383645624    1.0000000000
    13   1   1   1   1
    100.6331721843    1.0000000000
    14   2   2   1   1
      0.6369979122    1.0000000000
    15   2   2   1   1
      1.7195276810    1.0000000000
    16   2   2   1   1
     29.2342584121    1.0000000000
    17   3   3   1   1
      0.6446630421    1.0000000000
    18   3   3   1   1
      4.0797892747    1.0000000000
    19   4   4   1   1
      0.2759523441    1.0000000000
    20   4   4   1   1
      1.4474892985    1.0000000000

# RI basis set for S (all-electron) relative DI metric: 1.2e-02
S  RI_aug-DZVP-MOLOPT-ae_N_RI_039_s_p_d_f_g_h_i_5_4_3_1_0_0_0_error_1.2e-02
   13
     1   0   0   1   1
      0.2640581978    1.0000000000
     2   0   0   1   1
      0.7173465764    1.0000000000
     3   0   0   1   1
      5.5123278063    1.0000000000
     4   0   0   1   1
     48.3194153216    1.0000000000
     5   0   0   1   1
    350.1511357028    1.0000000000
     6   1   1   1   1
      0.3510461524    1.0000000000
     7   1   1   1   1
      0.5972893439    1.0000000000
     8   1   1   1   1
      9.6239831403    1.0000000000
     9   1   1   1   1
     97.1901975049    1.0000000000
    10   2   2   1   1
      0.7196272264    1.0000000000
    11   2   2   1   1
      4.5552803058    1.0000000000
    12   2   2   1   1
     28.4581367599    1.0000000000
    13   3   3   1   1
      0.8570773350    1.0000000000

# RI basis set for S (all-electron) relative DI metric: 5.2e-03
S  RI_aug-DZVP-MOLOPT-ae_N_RI_048_s_p_d_f_g_h_i_7_4_3_2_0_0_0_error_5.2e-03
   16
     1   0   0   1   1
      0.3020012495    1.0000000000
     2   0   0   1   1
      0.6111320981    1.0000000000
     3   0   0   1   1
      1.9736797107    1.0000000000
     4   0   0   1   1
      7.2451176709    1.0000000000
     5   0   0   1   1
     26.7479903083    1.0000000000
     6   0   0   1   1
     97.4674569940    1.0000000000
     7   0   0   1   1
    353.4008327994    1.0000000000
     8   1   1   1   1
      0.3283740756    1.0000000000
     9   1   1   1   1
      0.5668002359    1.0000000000
    10   1   1   1   1
      3.9767152195    1.0000000000
    11   1   1   1   1
     77.2346736413    1.0000000000
    12   2   2   1   1
      0.4988625787    1.0000000000
    13   2   2   1   1
      0.7647992711    1.0000000000
    14   2   2   1   1
      8.9845227182    1.0000000000
    15   3   3   1   1
      0.2693390233    1.0000000000
    16   3   3   1   1
      0.8676881869    1.0000000000

# RI basis set for S (all-electron) relative DI metric: 2.1e-03
```

```
S  RI_aug-DZVP-MOLOPT-ae_N_RI_065_s_p_d_f_g_h_i_7_5_4_2_1_0_0_error_2.1e-03
   19
     1   0   0   1   1
      0.2283137225    1.0000000000
     2   0   0   1   1
      0.6255889741    1.0000000000
     3   0   0   1   1
      2.1864182647    1.0000000000
     4   0   0   1   1
      8.7702241282    1.0000000000
     5   0   0   1   1
     32.4225304721    1.0000000000
     6   0   0   1   1
    108.3250475088    1.0000000000
     7   0   0   1   1
    349.6795017942    1.0000000000
     8   1   1   1   1
      0.3492099367    1.0000000000
     9   1   1   1   1
      1.3867673523    1.0000000000
    10   1   1   1   1
      5.5088948564    1.0000000000
    11   1   1   1   1
     21.8814356746    1.0000000000
    12   1   1   1   1
     86.9104068621    1.0000000000
    13   2   2   1   1
      0.3890590862    1.0000000000
    14   2   2   1   1
      0.8534851999    1.0000000000
    15   2   2   1   1
      4.6792369028    1.0000000000
    16   2   2   1   1
     25.9492411385    1.0000000000
    17   3   3   1   1
      0.4244930847    1.0000000000
    18   3   3   1   1
      1.2985823690    1.0000000000
    19   4   4   1   1
      0.7646178679    1.0000000000

# RI basis set for S (all-electron) relative DI metric: 1.8e-04
S  RI_aug-DZVP-MOLOPT-ae_N_RI_070_s_p_d_f_g_h_i_7_5_5_2_1_0_0_error_1.8e-04
   20
     1   0   0   1   1
      0.2901700939    1.0000000000
     2   0   0   1   1
      0.4752257357    1.0000000000
     3   0   0   1   1
      1.5884381939    1.0000000000
     4   0   0   1   1
      6.4907592281    1.0000000000
     5   0   0   1   1
     25.8526023777    1.0000000000
     6   0   0   1   1
     96.5079814877    1.0000000000
     7   0   0   1   1
    352.3772137793    1.0000000000
     8   1   1   1   1
      0.3146301567    1.0000000000
     9   1   1   1   1
      0.6575037643    1.0000000000
    10   1   1   1   1
      3.0827576458    1.0000000000
    11   1   1   1   1
     17.0845598869    1.0000000000
    12   1   1   1   1
     97.3693932404    1.0000000000
    13   2   2   1   1
      0.3847443439    1.0000000000
    14   2   2   1   1
      0.7374942444    1.0000000000
    15   2   2   1   1
      2.3925473350    1.0000000000
    16   2   2   1   1
      8.8547057817    1.0000000000
    17   2   2   1   1
     31.8457574816    1.0000000000
    18   3   3   1   1
      0.4108575481    1.0000000000
    19   3   3   1   1
      1.0692078390    1.0000000000
    20   4   4   1   1
      0.5614499318    1.0000000000

# RI basis set for S (all-electron) relative DI metric: 8.5e-05
S  RI_aug-DZVP-MOLOPT-ae_N_RI_073_s_p_d_f_g_h_i_7_6_5_2_1_0_0_error_8.5e-05
   21
     1   0   0   1   1
      0.2903921013    1.0000000000
     2   0   0   1   1
      0.4634786360    1.0000000000
     3   0   0   1   1
      1.5391648984    1.0000000000
     4   0   0   1   1
      6.2832808374    1.0000000000
     5   0   0   1   1
     25.5782923426    1.0000000000
     6   0   0   1   1
     96.2180926702    1.0000000000
     7   0   0   1   1
    352.0707936979    1.0000000000
     8   1   1   1   1
      0.3171056269    1.0000000000
     9   1   1   1   1
      0.5616827344    1.0000000000
    10   1   1   1   1
      1.8595799025    1.0000000000
    11   1   1   1   1
      7.4839051904    1.0000000000
    12   1   1   1   1
```

```
     28.5597942522    1.0000000000
    13   1   1   1   1
    100.9878228978    1.0000000000
    14   2   2   1   1
      0.4099180911    1.0000000000
    15   2   2   1   1
      0.7126948360    1.0000000000
    16   2   2   1   1
      2.3810439763    1.0000000000
    17   2   2   1   1
      8.8407579838    1.0000000000
    18   2   2   1   1
     31.8340184805    1.0000000000
    19   3   3   1   1
      0.3615010288    1.0000000000
    20   3   3   1   1
      0.9509529515    1.0000000000
    21   4   4   1   1
      0.5497230114    1.0000000000

# RI basis set for S (all-electron) relative DI metric: 3.0e-05
S  RI_aug-DZVP-MOLOPT-ae_N_RI_098_s_p_d_f_g_h_i_7_6_5_3_3_0_0_error_3.0e-05
   24
     1   0   0   1   1
      0.3346844148    1.0000000000
     2   0   0   1   1
      0.5381981321    1.0000000000
     3   0   0   1   1
      1.8545458393    1.0000000000
     4   0   0   1   1
      7.0482315264    1.0000000000
     5   0   0   1   1
     26.4919693543    1.0000000000
     6   0   0   1   1
     97.1799091720    1.0000000000
     7   0   0   1   1
    353.0917014359    1.0000000000
     8   1   1   1   1
      0.2621175202    1.0000000000
     9   1   1   1   1
      0.6284073108    1.0000000000
    10   1   1   1   1
      1.7285377280    1.0000000000
    11   1   1   1   1
      6.8209392732    1.0000000000
    12   1   1   1   1
     27.8366880419    1.0000000000
    13   1   1   1   1
    100.3434885260    1.0000000000
    14   2   2   1   1
      0.3929766104    1.0000000000
    15   2   2   1   1
      0.6760840257    1.0000000000
    16   2   2   1   1
      2.3809663304    1.0000000000
    17   2   2   1   1
      8.8493011289    1.0000000000
    18   2   2   1   1
     31.8481833122    1.0000000000
    19   3   3   1   1
      0.3555966732    1.0000000000
    20   3   3   1   1
      0.9371212043    1.0000000000
    21   3   3   1   1
      5.4777727495    1.0000000000
    22   4   4   1   1
      0.2934469255    1.0000000000
    23   4   4   1   1
      0.6961915044    1.0000000000
    24   4   4   1   1
      1.7365900772    1.0000000000

# RI basis set for S (all-electron) relative DI metric: 1.3e-05
S  RI_aug-DZVP-MOLOPT-ae_N_RI_112_s_p_d_f_g_h_i_7_6_5_5_3_0_0_error_1.3e-05
   26
     1   0   0   1   1
      0.2747308493    1.0000000000
     2   0   0   1   1
      0.4501924376    1.0000000000
     3   0   0   1   1
      1.5766362952    1.0000000000
     4   0   0   1   1
      6.1210136425    1.0000000000
     5   0   0   1   1
     24.9780892087    1.0000000000
     6   0   0   1   1
     95.4781304763    1.0000000000
     7   0   0   1   1
    351.2620329951    1.0000000000
     8   1   1   1   1
      0.2974099958    1.0000000000
     9   1   1   1   1
      0.5566607944    1.0000000000
    10   1   1   1   1
      1.6611037448    1.0000000000
    11   1   1   1   1
      6.3680763125    1.0000000000
    12   1   1   1   1
     21.7584926669    1.0000000000
    13   1   1   1   1
     70.3580587160    1.0000000000
    14   2   2   1   1
      0.3420117384    1.0000000000
    15   2   2   1   1
      0.8689039626    1.0000000000
    16   2   2   1   1
      1.7905251475    1.0000000000
    17   2   2   1   1
      8.0866464375    1.0000000000
    18   2   2   1   1
     31.3431623861    1.0000000000
```

```
    19   3   3   1   1
      0.1962228454    1.0000000000
    20   3   3   1   1
      0.4752074227    1.0000000000
    21   3   3   1   1
      1.1105638184    1.0000000000
    22   3   3   1   1
      2.4814503856    1.0000000000
    23   3   3   1   1
      5.8594677161    1.0000000000
    24   4   4   1   1
      0.3914745320    1.0000000000
    25   4   4   1   1
      0.7987067810    1.0000000000
    26   4   4   1   1
      1.6995491395    1.0000000000

# RI basis set for S (all-electron) relative DI metric: 3.4e-02
S  RI_aug-TZVP-MOLOPT-ae_N_RI_040_s_p_d_f_g_h_i_6_4_3_1_0_0_0_error_3.4e-02
   14
     1   0   0   1   1
      0.1727895936    1.0000000000
     2   0   0   1   1
      0.6828360568    1.0000000000
     3   0   0   1   1
      3.0607476237    1.0000000000
     4   0   0   1   1
     15.6065346900    1.0000000000
     5   0   0   1   1
     74.9916619788    1.0000000000
     6   0   0   1   1
    353.0552394372    1.0000000000
     7   1   1   1   1
      0.2568381153    1.0000000000
     8   1   1   1   1
      0.6392553882    1.0000000000
     9   1   1   1   1
      8.1127022036    1.0000000000
    10   1   1   1   1
     73.7920476487    1.0000000000
    11   2   2   1   1
      0.8480897610    1.0000000000
    12   2   2   1   1
      3.9671906305    1.0000000000
    13   2   2   1   1
     16.4650407781    1.0000000000
    14   3   3   1   1
      1.0878420221    1.0000000000

# RI basis set for S (all-electron) relative DI metric: 1.5e-02
S  RI_aug-TZVP-MOLOPT-ae_N_RI_055_s_p_d_f_g_h_i_7_4_4_1_1_0_0_error_1.5e-02
   17
     1   0   0   1   1
      0.2950364312    1.0000000000
     2   0   0   1   1
      0.5733224975    1.0000000000
     3   0   0   1   1
      1.7581699749    1.0000000000
     4   0   0   1   1
      6.8206937911    1.0000000000
     5   0   0   1   1
     26.2336784133    1.0000000000
     6   0   0   1   1
     96.8977188814    1.0000000000
     7   0   0   1   1
    352.7881659991    1.0000000000
     8   1   1   1   1
      0.4648099241    1.0000000000
     9   1   1   1   1
      1.7519773325    1.0000000000
    10   1   1   1   1
      8.8547151398    1.0000000000
    11   1   1   1   1
     40.9977024441    1.0000000000
    12   2   2   1   1
      0.5846590502    1.0000000000
    13   2   2   1   1
      1.2891719515    1.0000000000
    14   2   2   1   1
      6.6298970141    1.0000000000
    15   2   2   1   1
     30.4247329077    1.0000000000
    16   3   3   1   1
      0.6216081803    1.0000000000
    17   4   4   1   1
      0.9879880714    1.0000000000

# RI basis set for S (all-electron) relative DI metric: 7.0e-03
S  RI_aug-TZVP-MOLOPT-ae_N_RI_061_s_p_d_f_g_h_i_7_6_4_1_1_0_0_error_7.0e-03
   19
     1   0   0   1   1
      0.3193433489    1.0000000000
     2   0   0   1   1
      0.4956876232    1.0000000000
     3   0   0   1   1
      1.4878494921    1.0000000000
     4   0   0   1   1
      5.9361831390    1.0000000000
     5   0   0   1   1
     24.8269313311    1.0000000000
     6   0   0   1   1
     95.3440464099    1.0000000000
     7   0   0   1   1
    351.1329302506    1.0000000000
     8   1   1   1   1
      0.4087707076    1.0000000000
     9   1   1   1   1
      0.5088848973    1.0000000000
    10   1   1   1   1
      1.8187438010    1.0000000000
    11   1   1   1   1
```

```
      6.8060545856    1.0000000000
    12   1   1   1   1
     27.2842753864    1.0000000000
    13   1   1   1   1
     99.7858245095    1.0000000000
    14   2   2   1   1
      0.5435237587    1.0000000000
    15   2   2   1   1
      1.3094498200    1.0000000000
    16   2   2   1   1
      5.7234923533    1.0000000000
    17   2   2   1   1
     22.4329184009    1.0000000000
    18   3   3   1   1
      0.8064003431    1.0000000000
    19   4   4   1   1
      0.7404516823    1.0000000000

# RI basis set for S (all-electron) relative DI metric: 3.4e-03
S  RI_aug-TZVP-MOLOPT-ae_N_RI_068_s_p_d_f_g_h_i_7_6_4_2_1_0_0_error_3.4e-03
   20
     1   0   0   1   1
      0.3201316182    1.0000000000
     2   0   0   1   1
      0.5185853180    1.0000000000
     3   0   0   1   1
      1.5116275581    1.0000000000
     4   0   0   1   1
      5.8847630152    1.0000000000
     5   0   0   1   1
     24.6615248256    1.0000000000
     6   0   0   1   1
     95.1605748317    1.0000000000
     7   0   0   1   1
    350.9420154965    1.0000000000
     8   1   1   1   1
      0.3929266753    1.0000000000
     9   1   1   1   1
      0.6116877583    1.0000000000
    10   1   1   1   1
      1.7860536661    1.0000000000
    11   1   1   1   1
      6.4150645017    1.0000000000
    12   1   1   1   1
     26.3147395459    1.0000000000
    13   1   1   1   1
     98.8708032004    1.0000000000
    14   2   2   1   1
      0.3835305532    1.0000000000
    15   2   2   1   1
      1.1158416531    1.0000000000
    16   2   2   1   1
      4.2473840098    1.0000000000
    17   2   2   1   1
     14.7159176277    1.0000000000
    18   3   3   1   1
      0.5745635687    1.0000000000
    19   3   3   1   1
      1.0689484485    1.0000000000
    20   4   4   1   1
      0.9288692530    1.0000000000

# RI basis set for S (all-electron) relative DI metric: 1.7e-03
S  RI_aug-TZVP-MOLOPT-ae_N_RI_079_s_p_d_f_g_h_i_7_6_4_2_1_1_0_error_1.7e-03
   21
     1   0   0   1   1
      0.3112139767    1.0000000000
     2   0   0   1   1
      0.5158932785    1.0000000000
     3   0   0   1   1
      1.5592776225    1.0000000000
     4   0   0   1   1
      6.1529848242    1.0000000000
     5   0   0   1   1
     25.2309692181    1.0000000000
     6   0   0   1   1
     95.8044546187    1.0000000000
     7   0   0   1   1
    351.6262770198    1.0000000000
     8   1   1   1   1
      0.3815041799    1.0000000000
     9   1   1   1   1
      0.6045297671    1.0000000000
    10   1   1   1   1
      1.8321090509    1.0000000000
    11   1   1   1   1
      6.8473449096    1.0000000000
    12   1   1   1   1
     27.4753415717    1.0000000000
    13   1   1   1   1
     99.9822840263    1.0000000000
    14   2   2   1   1
      0.5628011532    1.0000000000
    15   2   2   1   1
      0.7720564870    1.0000000000
    16   2   2   1   1
      3.1506457122    1.0000000000
    17   2   2   1   1
     18.0514430915    1.0000000000
    18   3   3   1   1
      0.4450769100    1.0000000000
    19   3   3   1   1
      1.1109376341    1.0000000000
    20   4   4   1   1
      0.9347185779    1.0000000000
    21   5   5   1   1
      1.3667021350    1.0000000000

# RI basis set for S (all-electron) relative DI metric: 4.9e-04
S  RI_aug-TZVP-MOLOPT-ae_N_RI_107_s_p_d_f_g_h_i_7_6_5_4_2_1_0_error_4.9e-04
   25
```

```
      1   0   0   1   1
       0.2572111090    1.0000000000
      2   0   0   1   1
       0.4304379135    1.0000000000
      3   0   0   1   1
       1.5884307013    1.0000000000
      4   0   0   1   1
       6.1857145714    1.0000000000
      5   0   0   1   1
      25.0970959122    1.0000000000
      6   0   0   1   1
      95.6176647735    1.0000000000
      7   0   0   1   1
     351.4221327033    1.0000000000
      8   1   1   1   1
       0.3303637640    1.0000000000
      9   1   1   1   1
       0.5726771550    1.0000000000
     10   1   1   1   1
       1.7355331333    1.0000000000
     11   1   1   1   1
       6.1418594219    1.0000000000
     12   1   1   1   1
      24.7686789465    1.0000000000
     13   1   1   1   1
      97.1501992861    1.0000000000
     14   2   2   1   1
       0.3965241856    1.0000000000
     15   2   2   1   1
       0.6060124557    1.0000000000
     16   2   2   1   1
       1.8846751425    1.0000000000
     17   2   2   1   1
       6.3749587464    1.0000000000
     18   2   2   1   1
      20.1306827476    1.0000000000
     19   3   3   1   1
       0.3240904223    1.0000000000
     20   3   3   1   1
       0.7067032417    1.0000000000
     21   3   3   1   1
       1.8022436973    1.0000000000
     22   3   3   1   1
       4.6286672632    1.0000000000
     23   4   4   1   1
       0.8104079161    1.0000000000
     24   4   4   1   1
       1.6309399089    1.0000000000
     25   5   5   1   1
       0.9858921275    1.0000000000

# RI basis set for Cl (all-electron) relative DI metric: 2.9e-02
Cl  RI_aug-SZV-MOLOPT-ae-mini_N_RI_011_s_p_d_f_g_h_i_3_1_1_0_0_0_0_error_2.9e-02
   5
      1   0   0   1   1
       0.2753200298    1.0000000000
      2   0   0   1   1
       0.4929012904    1.0000000000
      3   0   0   1   1
       0.8849019267    1.0000000000
      4   1   1   1   1
       2.3246274012    1.0000000000
      5   2   2   1   1
       1.7800077813    1.0000000000

# RI basis set for Cl (all-electron) relative DI metric: 4.1e-03
Cl  RI_aug-SZV-MOLOPT-ae-mini_N_RI_014_s_p_d_f_g_h_i_3_2_1_0_0_0_0_error_4.1e-03
   6
      1   0   0   1   1
       0.3346343565    1.0000000000
      2   0   0   1   1
       0.7463421181    1.0000000000
      3   0   0   1   1
       1.6347955261    1.0000000000
      4   1   1   1   1
       0.4967832479    1.0000000000
      5   1   1   1   1
       0.8509423063    1.0000000000
      6   2   2   1   1
       1.8137182743    1.0000000000

# RI basis set for Cl (all-electron) relative DI metric: 3.1e-04
Cl  RI_aug-SZV-MOLOPT-ae-mini_N_RI_019_s_p_d_f_g_h_i_3_2_2_0_0_0_0_error_3.1e-04
   7
      1   0   0   1   1
       0.2653531384    1.0000000000
      2   0   0   1   1
       0.6314021229    1.0000000000
      3   0   0   1   1
       1.0939054908    1.0000000000
      4   1   1   1   1
       0.3715518348    1.0000000000
      5   1   1   1   1
       1.1759537978    1.0000000000
      6   2   2   1   1
       0.2075466466    1.0000000000
      7   2   2   1   1
       1.0993237991    1.0000000000

# RI basis set for Cl (all-electron) relative DI metric: 7.8e-05
Cl  RI_aug-SZV-MOLOPT-ae-mini_N_RI_021_s_p_d_f_g_h_i_5_2_2_0_0_0_0_error_7.8e-05
   9
      1   0   0   1   1
       0.1938497056    1.0000000000
      2   0   0   1   1
       0.4214534460    1.0000000000
      3   0   0   1   1
       0.8717593146    1.0000000000
      4   0   0   1   1
       1.7373929847    1.0000000000
      5   0   0   1   1
```

```
      3.5311544267    1.0000000000
     6   1   1   1   1
      0.6120433429    1.0000000000
     7   1   1   1   1
      0.7746948978    1.0000000000
     8   2   2   1   1
      0.2042879670    1.0000000000
     9   2   2   1   1
      1.1337598088    1.0000000000

# RI basis set for Cl (all-electron) relative DI metric: 1.1e-05
Cl  RI_aug-SZV-MOLOPT-ae-mini_N_RI_024_s_p_d_f_g_h_i_5_3_2_0_0_0_0_error_1.1e-05
   10
     1   0   0   1   1
      0.1908666034    1.0000000000
     2   0   0   1   1
      0.4133557109    1.0000000000
     3   0   0   1   1
      0.8619072538    1.0000000000
     4   0   0   1   1
      1.7380654938    1.0000000000
     5   0   0   1   1
      3.5416093342    1.0000000000
     6   1   1   1   1
      0.2177576092    1.0000000000
     7   1   1   1   1
      0.5783272425    1.0000000000
     8   1   1   1   1
      1.2488078757    1.0000000000
     9   2   2   1   1
      0.2386832122    1.0000000000
    10   2   2   1   1
      1.0189456200    1.0000000000

# RI basis set for Cl (all-electron) relative DI metric: 2.1e-06
Cl  RI_aug-SZV-MOLOPT-ae-mini_N_RI_025_s_p_d_f_g_h_i_6_3_2_0_0_0_0_error_2.1e-06
   11
     1   0   0   1   1
      0.1949166613    1.0000000000
     2   0   0   1   1
      0.3572067352    1.0000000000
     3   0   0   1   1
      0.6305728647    1.0000000000
     4   0   0   1   1
      1.1056915220    1.0000000000
     5   0   0   1   1
      1.9818057901    1.0000000000
     6   0   0   1   1
      3.5520114789    1.0000000000
     7   1   1   1   1
      0.2924242644    1.0000000000
     8   1   1   1   1
      0.6341743475    1.0000000000
     9   1   1   1   1
      1.1787393037    1.0000000000
    10   2   2   1   1
      0.2509290439    1.0000000000
    11   2   2   1   1
      1.1367750250    1.0000000000

# RI basis set for Cl (all-electron) relative DI metric: 9.5e-07
Cl  RI_aug-SZV-MOLOPT-ae-mini_N_RI_030_s_p_d_f_g_h_i_6_3_3_0_0_0_0_error_9.5e-07
   12
     1   0   0   1   1
      0.2001963688    1.0000000000
     2   0   0   1   1
      0.3554372478    1.0000000000
     3   0   0   1   1
      0.6319470208    1.0000000000
     4   0   0   1   1
      1.1238923230    1.0000000000
     5   0   0   1   1
      1.9971619106    1.0000000000
     6   0   0   1   1
      3.5488989094    1.0000000000
     7   1   1   1   1
      0.3788936110    1.0000000000
     8   1   1   1   1
      0.9055623848    1.0000000000
     9   1   1   1   1
      1.7564414979    1.0000000000
    10   2   2   1   1
      0.2412583631    1.0000000000
    11   2   2   1   1
      1.0229473596    1.0000000000
    12   2   2   1   1
      3.3934830750    1.0000000000

# RI basis set for Cl (all-electron) relative DI metric: 7.1e-08
Cl  RI_aug-SZV-MOLOPT-ae-mini_N_RI_046_s_p_d_f_g_h_i_6_5_5_0_0_0_0_error_7.1e-08
   16
     1   0   0   1   1
      0.1999999998    1.0000000000
     2   0   0   1   1
      0.3555041094    1.0000000000
     3   0   0   1   1
      0.6319158592    1.0000000000
     4   0   0   1   1
      1.1232434237    1.0000000000
     5   0   0   1   1
      1.9965882653    1.0000000000
     6   0   0   1   1
      3.5489766664    1.0000000000
     7   1   1   1   1
      0.2000000000    1.0000000000
     8   1   1   1   1
      0.4104859368    1.0000000000
     9   1   1   1   1
      0.8424935212    1.0000000000
    10   1   1   1   1
      1.7291587110    1.0000000000
```

```
    11   1   1   1   1
      3.5489766666    1.0000000000
    12   2   2   1   1
      0.2000000000    1.0000000000
    13   2   2   1   1
      0.4104859367    1.0000000000
    14   2   2   1   1
      0.8424935212    1.0000000000
    15   2   2   1   1
      1.7291587113    1.0000000000
    16   2   2   1   1
      3.5489766667    1.0000000000

# RI basis set for Cl (all-electron) relative DI metric: 1.4e-08
Cl  RI_aug-SZV-MOLOPT-ae-mini_N_RI_047_s_p_d_f_g_h_i_7_5_5_0_0_0_0_error_1.4e-08
   17
     1   0   0   1   1
      0.2000000000    1.0000000000
     2   0   0   1   1
      0.3230047089    1.0000000000
     3   0   0   1   1
      0.5216602099    1.0000000000
     4   0   0   1   1
      0.8424935212    1.0000000000
     5   0   0   1   1
      1.3606468729    1.0000000000
     6   0   0   1   1
      2.1974767356    1.0000000000
     7   0   0   1   1
      3.5489766667    1.0000000000
     8   1   1   1   1
      0.2000000000    1.0000000000
     9   1   1   1   1
      0.4104859368    1.0000000000
    10   1   1   1   1
      0.8424935212    1.0000000000
    11   1   1   1   1
      1.7291587110    1.0000000000
    12   1   1   1   1
      3.5489766666    1.0000000000
    13   2   2   1   1
      0.2000000000    1.0000000000
    14   2   2   1   1
      0.4104859367    1.0000000000
    15   2   2   1   1
      0.8424935213    1.0000000000
    16   2   2   1   1
      1.7291587112    1.0000000000
    17   2   2   1   1
      3.5489766666    1.0000000000

# RI basis set for Cl (all-electron) relative DI metric: 6.9e-09
Cl  RI_aug-SZV-MOLOPT-ae-mini_N_RI_050_s_p_d_f_g_h_i_7_6_5_0_0_0_0_error_6.9e-09
   18
     1   0   0   1   1
      0.2000000000    1.0000000000
     2   0   0   1   1
      0.3230047089    1.0000000000
     3   0   0   1   1
      0.5216602099    1.0000000000
     4   0   0   1   1
      0.8424935212    1.0000000000
     5   0   0   1   1
      1.3606468729    1.0000000000
     6   0   0   1   1
      2.1974767356    1.0000000000
     7   0   0   1   1
      3.5489766667    1.0000000000
     8   1   1   1   1
      0.2000000000    1.0000000000
     9   1   1   1   1
      0.3555041095    1.0000000000
    10   1   1   1   1
      0.6319158592    1.0000000000
    11   1   1   1   1
      1.1232434239    1.0000000000
    12   1   1   1   1
      1.9965882656    1.0000000000
    13   1   1   1   1
      3.5489766667    1.0000000000
    14   2   2   1   1
      0.2000000000    1.0000000000
    15   2   2   1   1
      0.4104859367    1.0000000000
    16   2   2   1   1
      0.8424935213    1.0000000000
    17   2   2   1   1
      1.7291587112    1.0000000000
    18   2   2   1   1
      3.5489766666    1.0000000000

# RI basis set for Cl (all-electron) relative DI metric: 7.0e-03
Cl  RI_aug-SZV-MOLOPT-ae_N_RI_027_s_p_d_f_g_h_i_6_3_1_1_0_0_0_error_7.0e-03
   11
     1   0   0   1   1
      0.2448810414    1.0000000000
     2   0   0   1   1
      1.0689705418    1.0000000000
     3   0   0   1   1
      4.7614002889    1.0000000000
     4   0   0   1   1
     21.3798898754    1.0000000000
     5   0   0   1   1
     95.0610573491    1.0000000000
     6   0   0   1   1
    421.4539666580    1.0000000000
     7   1   1   1   1
      0.5326781235    1.0000000000
     8   1   1   1   1
      6.6253203880    1.0000000000
     9   1   1   1   1
```

```
     82.4813040231    1.0000000000
    10   2   2   1   1
      0.9298305952    1.0000000000
    11   3   3   1   1
      0.7501079429    1.0000000000

# RI basis set for Cl (all-electron) relative DI metric: 1.3e-03
Cl  RI_aug-SZV-MOLOPT-ae_N_RI_042_s_p_d_f_g_h_i_6_5_1_1_1_0_0_error_1.3e-03
   14
     1   0   0   1   1
      0.9722459299    1.0000000000
     2   0   0   1   1
      2.5847135575    1.0000000000
     3   0   0   1   1
      9.1897121065    1.0000000000
     4   0   0   1   1
     33.1834735350    1.0000000000
     5   0   0   1   1
    118.9618672292    1.0000000000
     6   0   0   1   1
    425.3304907559    1.0000000000
     7   1   1   1   1
      0.4794123927    1.0000000000
     8   1   1   1   1
      1.5588839623    1.0000000000
     9   1   1   1   1
      7.5715838698    1.0000000000
    10   1   1   1   1
     32.4184510010    1.0000000000
    11   1   1   1   1
    133.4378309350    1.0000000000
    12   2   2   1   1
      0.8972337822    1.0000000000
    13   3   3   1   1
      0.6843780723    1.0000000000
    14   4   4   1   1
      0.3298038429    1.0000000000

# RI basis set for Cl (all-electron) relative DI metric: 3.4e-04
Cl  RI_aug-SZV-MOLOPT-ae_N_RI_063_s_p_d_f_g_h_i_6_5_2_2_2_0_0_error_3.4e-04
   17
     1   0   0   1   1
      0.3018898096    1.0000000000
     2   0   0   1   1
      0.9228413056    1.0000000000
     3   0   0   1   1
      4.1638344789    1.0000000000
     4   0   0   1   1
     19.2684781928    1.0000000000
     5   0   0   1   1
     90.3883791372    1.0000000000
     6   0   0   1   1
    425.1773114753    1.0000000000
     7   1   1   1   1
      0.2841143814    1.0000000000
     8   1   1   1   1
      0.9496935475    1.0000000000
     9   1   1   1   1
      6.3627453129    1.0000000000
    10   1   1   1   1
     31.3383429971    1.0000000000
    11   1   1   1   1
    132.4993560674    1.0000000000
    12   2   2   1   1
      0.8276392269    1.0000000000
    13   2   2   1   1
     37.3850122334    1.0000000000
    14   3   3   1   1
      0.8436249285    1.0000000000
    15   3   3   1   1
      4.8435054150    1.0000000000
    16   4   4   1   1
      0.2435315033    1.0000000000
    17   4   4   1   1
      2.3311468165    1.0000000000

# RI basis set for Cl (all-electron) relative DI metric: 1.6e-04
Cl  RI_aug-SZV-MOLOPT-ae_N_RI_079_s_p_d_f_g_h_i_6_5_3_2_2_1_0_error_1.6e-04
   19
     1   0   0   1   1
      0.3933878227    1.0000000000
     2   0   0   1   1
      0.9454117099    1.0000000000
     3   0   0   1   1
      4.1420015946    1.0000000000
     4   0   0   1   1
     19.2397433182    1.0000000000
     5   0   0   1   1
     90.3706523323    1.0000000000
     6   0   0   1   1
    425.1594269187    1.0000000000
     7   1   1   1   1
      0.3001587979    1.0000000000
     8   1   1   1   1
      0.5969784741    1.0000000000
     9   1   1   1   1
      5.4196543673    1.0000000000
    10   1   1   1   1
     27.2246847590    1.0000000000
    11   1   1   1   1
    134.8168376223    1.0000000000
    12   2   2   1   1
      0.7330253897    1.0000000000
    13   2   2   1   1
      2.3082928767    1.0000000000
    14   2   2   1   1
     38.7255190037    1.0000000000
    15   3   3   1   1
      0.8123288781    1.0000000000
    16   3   3   1   1
      4.5384937408    1.0000000000
```

```
    17   4   4   1   1
      0.2878171564    1.0000000000
    18   4   4   1   1
      1.8342334841    1.0000000000
    19   5   5   1   1
      1.2372489257    1.0000000000

# RI basis set for Cl (all-electron) relative DI metric: 6.4e-05
Cl  RI_aug-SZV-MOLOPT-ae_N_RI_108_s_p_d_f_g_h_i_6_5_3_3_3_1_1_error_6.4e-05
   22
     1   0   0   1   1
      0.3440375331    1.0000000000
     2   0   0   1   1
      0.9015038618    1.0000000000
     3   0   0   1   1
      4.1407281194    1.0000000000
     4   0   0   1   1
     19.2483089875    1.0000000000
     5   0   0   1   1
     90.3699995645    1.0000000000
     6   0   0   1   1
    425.1582449022    1.0000000000
     7   1   1   1   1
      0.3836939269    1.0000000000
     8   1   1   1   1
      0.8683276566    1.0000000000
     9   1   1   1   1
      5.5114686366    1.0000000000
    10   1   1   1   1
     28.1208556839    1.0000000000
    11   1   1   1   1
    134.8575264426    1.0000000000
    12   2   2   1   1
      0.7053218898    1.0000000000
    13   2   2   1   1
      1.5977675319    1.0000000000
    14   2   2   1   1
     26.0899548387    1.0000000000
    15   3   3   1   1
      0.4931251285    1.0000000000
    16   3   3   1   1
      1.6851753289    1.0000000000
    17   3   3   1   1
      7.1710126576    1.0000000000
    18   4   4   1   1
      0.2697818932    1.0000000000
    19   4   4   1   1
      1.0380003963    1.0000000000
    20   4   4   1   1
      2.2129397202    1.0000000000
    21   5   5   1   1
      1.3084585494    1.0000000000
    22   6   6   1   1
      1.9290113032    1.0000000000

# RI basis set for Cl (all-electron) relative DI metric: 2.8e-05
Cl  RI_aug-SZV-MOLOPT-ae_N_RI_112_s_p_d_f_g_h_i_7_6_3_3_3_1_1_error_2.8e-05
   24
     1   0   0   1   1
      0.3397027078    1.0000000000
     2   0   0   1   1
      0.7115714784    1.0000000000
     3   0   0   1   1
      2.4502250663    1.0000000000
     4   0   0   1   1
      8.8468086811    1.0000000000
     5   0   0   1   1
     32.1494707956    1.0000000000
     6   0   0   1   1
    116.8932415011    1.0000000000
     7   0   0   1   1
    425.0722262339    1.0000000000
     8   1   1   1   1
      0.3307645420    1.0000000000
     9   1   1   1   1
      0.7286616765    1.0000000000
    10   1   1   1   1
      3.1029990953    1.0000000000
    11   1   1   1   1
     11.0894461404    1.0000000000
    12   1   1   1   1
     38.7486520758    1.0000000000
    13   1   1   1   1
    135.0328217310    1.0000000000
    14   2   2   1   1
      0.5626605150    1.0000000000
    15   2   2   1   1
      1.1491133166    1.0000000000
    16   2   2   1   1
     26.8500403379    1.0000000000
    17   3   3   1   1
      0.6711085085    1.0000000000
    18   3   3   1   1
      1.6336588580    1.0000000000
    19   3   3   1   1
      7.1240162845    1.0000000000
    20   4   4   1   1
      0.2790152691    1.0000000000
    21   4   4   1   1
      1.0662082487    1.0000000000
    22   4   4   1   1
      2.2209820056    1.0000000000
    23   5   5   1   1
      1.6662243823    1.0000000000
    24   6   6   1   1
      1.9242475757    1.0000000000

# RI basis set for Cl (all-electron) relative DI metric: 1.8e-02
Cl  RI_aug-DZVP-MOLOPT-ae_N_RI_044_s_p_d_f_g_h_i_6_4_2_1_1_0_0_error_1.8e-02
   14
     1   0   0   1   1
```

```
      0.3642851831    1.0000000000
     2   0   0   1   1
      0.9726988905    1.0000000000
     3   0   0   1   1
      4.2428295311    1.0000000000
     4   0   0   1   1
     19.2570802658    1.0000000000
     5   0   0   1   1
     90.3530029151    1.0000000000
     6   0   0   1   1
    425.1381241212    1.0000000000
     7   1   1   1   1
      0.3649604372    1.0000000000
     8   1   1   1   1
      1.2442122650    1.0000000000
     9   1   1   1   1
      7.3436354760    1.0000000000
    10   1   1   1   1
    111.4012070199    1.0000000000
    11   2   2   1   1
      2.5485958039    1.0000000000
    12   2   2   1   1
     13.1658769922    1.0000000000
    13   3   3   1   1
      1.7232506398    1.0000000000
    14   4   4   1   1
      0.6243390565    1.0000000000

# RI basis set for Cl (all-electron) relative DI metric: 2.1e-03
Cl  RI_aug-DZVP-MOLOPT-ae_N_RI_054_s_p_d_f_g_h_i_6_4_4_1_1_0_0_error_2.1e-03
   16
     1   0   0   1   1
      0.2092710578    1.0000000000
     2   0   0   1   1
      0.7344600365    1.0000000000
     3   0   0   1   1
      3.7253897356    1.0000000000
     4   0   0   1   1
     18.7010688748    1.0000000000
     5   0   0   1   1
     89.7498469947    1.0000000000
     6   0   0   1   1
    424.4713851725    1.0000000000
     7   1   1   1   1
      0.2912067248    1.0000000000
     8   1   1   1   1
      0.6934373921    1.0000000000
     9   1   1   1   1
      5.3311523635    1.0000000000
    10   1   1   1   1
    150.0975079323    1.0000000000
    11   2   2   1   1
      0.3017316540    1.0000000000
    12   2   2   1   1
      1.0036950246    1.0000000000
    13   2   2   1   1
      4.8189708702    1.0000000000
    14   2   2   1   1
     27.1075112438    1.0000000000
    15   3   3   1   1
      1.0137994372    1.0000000000
    16   4   4   1   1
      1.3484642123    1.0000000000

# RI basis set for Cl (all-electron) relative DI metric: 4.2e-04
Cl  RI_aug-DZVP-MOLOPT-ae_N_RI_068_s_p_d_f_g_h_i_7_6_4_2_1_0_0_error_4.2e-04
   20
     1   0   0   1   1
      0.2636819961    1.0000000000
     2   0   0   1   1
      0.7222242713    1.0000000000
     3   0   0   1   1
      2.6823303775    1.0000000000
     4   0   0   1   1
     11.0972130483    1.0000000000
     5   0   0   1   1
     39.5861520750    1.0000000000
     6   0   0   1   1
    130.8325838952    1.0000000000
     7   0   0   1   1
    422.6643268536    1.0000000000
     8   1   1   1   1
      0.4034013479    1.0000000000
     9   1   1   1   1
      0.6555135916    1.0000000000
    10   1   1   1   1
      3.1522713375    1.0000000000
    11   1   1   1   1
     11.3960707136    1.0000000000
    12   1   1   1   1
     39.3552950049    1.0000000000
    13   1   1   1   1
    133.7373899990    1.0000000000
    14   2   2   1   1
      0.3384505431    1.0000000000
    15   2   2   1   1
      1.0030341747    1.0000000000
    16   2   2   1   1
      6.6427120017    1.0000000000
    17   2   2   1   1
     33.0869370307    1.0000000000
    18   3   3   1   1
      0.4555405397    1.0000000000
    19   3   3   1   1
      1.5781969428    1.0000000000
    20   4   4   1   1
      1.2172980904    1.0000000000

# RI basis set for Cl (all-electron) relative DI metric: 1.3e-04
Cl  RI_aug-DZVP-MOLOPT-ae_N_RI_084_s_p_d_f_g_h_i_7_6_4_3_2_0_0_error_1.3e-04
   22
```

```
     1   0   0   1   1
      0.2085751891    1.0000000000
     2   0   0   1   1
      0.6173661565    1.0000000000
     3   0   0   1   1
      2.4525439799    1.0000000000
     4   0   0   1   1
      8.8235336377    1.0000000000
     5   0   0   1   1
     32.0972369931    1.0000000000
     6   0   0   1   1
    116.8297895678    1.0000000000
     7   0   0   1   1
    425.0031961089    1.0000000000
     8   1   1   1   1
      0.2621478963    1.0000000000
     9   1   1   1   1
      0.8154575912    1.0000000000
    10   1   1   1   1
      3.1045648272    1.0000000000
    11   1   1   1   1
     11.0273543220    1.0000000000
    12   1   1   1   1
     38.6879838208    1.0000000000
    13   1   1   1   1
    134.9786666125    1.0000000000
    14   2   2   1   1
      0.3956705209    1.0000000000
    15   2   2   1   1
      0.8920066879    1.0000000000
    16   2   2   1   1
      6.2633699855    1.0000000000
    17   2   2   1   1
     38.7639221139    1.0000000000
    18   3   3   1   1
      0.3614895010    1.0000000000
    19   3   3   1   1
      1.1892784782    1.0000000000
    20   3   3   1   1
      6.9696118284    1.0000000000
    21   4   4   1   1
      0.6995802494    1.0000000000
    22   4   4   1   1
      1.6491856286    1.0000000000

# RI basis set for Cl (all-electron) relative DI metric: 2.6e-05
Cl  RI_aug-DZVP-MOLOPT-ae_N_RI_114_s_p_d_f_g_h_i_7_6_5_4_4_0_0_error_2.6e-05
   26
     1   0   0   1   1
      0.3851988696    1.0000000000
     2   0   0   1   1
      0.7309682527    1.0000000000
     3   0   0   1   1
      2.6914752757    1.0000000000
     4   0   0   1   1
     11.0841175273    1.0000000000
     5   0   0   1   1
     39.4144157350    1.0000000000
     6   0   0   1   1
    130.5057502940    1.0000000000
     7   0   0   1   1
    422.4256934008    1.0000000000
     8   1   1   1   1
      0.2677626368    1.0000000000
     9   1   1   1   1
      0.7702719838    1.0000000000
    10   1   1   1   1
      3.0758033997    1.0000000000
    11   1   1   1   1
     10.5883473247    1.0000000000
    12   1   1   1   1
     38.2405482841    1.0000000000
    13   1   1   1   1
    134.5627967263    1.0000000000
    14   2   2   1   1
      0.4512514634    1.0000000000
    15   2   2   1   1
      0.7110871095    1.0000000000
    16   2   2   1   1
      2.6811094680    1.0000000000
    17   2   2   1   1
     10.3906581583    1.0000000000
    18   2   2   1   1
     39.0320723382    1.0000000000
    19   3   3   1   1
      0.3119070685    1.0000000000
    20   3   3   1   1
      0.8616333357    1.0000000000
    21   3   3   1   1
      2.2581745822    1.0000000000
    22   3   3   1   1
      6.2259228126    1.0000000000
    23   4   4   1   1
      0.2665538068    1.0000000000
    24   4   4   1   1
      0.5343879417    1.0000000000
    25   4   4   1   1
      1.0596130296    1.0000000000
    26   4   4   1   1
      2.1887627489    1.0000000000

# RI basis set for Cl (all-electron) relative DI metric: 2.5e-02
Cl  RI_aug-TZVP-MOLOPT-ae_N_RI_042_s_p_d_f_g_h_i_6_5_1_1_1_0_0_error_2.5e-02
   14
     1   0   0   1   1
      0.4021851921    1.0000000000
     2   0   0   1   1
      0.6553436891    1.0000000000
     3   0   0   1   1
      2.8693634148    1.0000000000
     4   0   0   1   1
```

```
     16.0549038217    1.0000000000
     5   0   0   1   1
     86.4952260942    1.0000000000
     6   0   0   1   1
    420.9162539920    1.0000000000
     7   1   1   1   1
      0.5475770899    1.0000000000
     8   1   1   1   1
      0.9626865277    1.0000000000
     9   1   1   1   1
      5.2291441403    1.0000000000
    10   1   1   1   1
     26.5772063719    1.0000000000
    11   1   1   1   1
    112.8661187079    1.0000000000
    12   2   2   1   1
      1.0767670919    1.0000000000
    13   3   3   1   1
      1.0368261691    1.0000000000
    14   4   4   1   1
      0.6070604168    1.0000000000

# RI basis set for Cl (all-electron) relative DI metric: 1.1e-02
Cl  RI_aug-TZVP-MOLOPT-ae_N_RI_057_s_p_d_f_g_h_i_6_5_4_1_1_0_0_error_1.1e-02
   17
     1   0   0   1   1
      0.4482434090    1.0000000000
     2   0   0   1   1
      0.7075182867    1.0000000000
     3   0   0   1   1
      2.8583750550    1.0000000000
     4   0   0   1   1
     15.7789475403    1.0000000000
     5   0   0   1   1
     86.1093102007    1.0000000000
     6   0   0   1   1
    420.5063083334    1.0000000000
     7   1   1   1   1
      0.3895169091    1.0000000000
     8   1   1   1   1
      1.2210520374    1.0000000000
     9   1   1   1   1
      5.6940351013    1.0000000000
    10   1   1   1   1
     25.9513103879    1.0000000000
    11   1   1   1   1
     99.3632780560    1.0000000000
    12   2   2   1   1
      0.6582056251    1.0000000000
    13   2   2   1   1
      1.1559733172    1.0000000000
    14   2   2   1   1
      5.2734333989    1.0000000000
    15   2   2   1   1
     37.8412328826    1.0000000000
    16   3   3   1   1
      0.9876572546    1.0000000000
    17   4   4   1   1
      1.1436987628    1.0000000000

# RI basis set for Cl (all-electron) relative DI metric: 4.9e-03
Cl  RI_aug-TZVP-MOLOPT-ae_N_RI_064_s_p_d_f_g_h_i_6_5_4_2_1_0_0_error_4.9e-03
   18
     1   0   0   1   1
      0.4214447605    1.0000000000
     2   0   0   1   1
      0.7479260889    1.0000000000
     3   0   0   1   1
      2.5870288856    1.0000000000
     4   0   0   1   1
     14.8084844753    1.0000000000
     5   0   0   1   1
     86.8923700916    1.0000000000
     6   0   0   1   1
    397.6518266946    1.0000000000
     7   1   1   1   1
      0.4364356372    1.0000000000
     8   1   1   1   1
      0.8979804936    1.0000000000
     9   1   1   1   1
      2.5947746342    1.0000000000
    10   1   1   1   1
     16.6114160985    1.0000000000
    11   1   1   1   1
    105.2100363529    1.0000000000
    12   2   2   1   1
      0.5159168000    1.0000000000
    13   2   2   1   1
      1.6050839072    1.0000000000
    14   2   2   1   1
      6.2812730462    1.0000000000
    15   2   2   1   1
     22.4393166495    1.0000000000
    16   3   3   1   1
      0.5549888363    1.0000000000
    17   3   3   1   1
      1.6557072870    1.0000000000
    18   4   4   1   1
      1.0746967009    1.0000000000

# RI basis set for Cl (all-electron) relative DI metric: 1.9e-03
Cl  RI_aug-TZVP-MOLOPT-ae_N_RI_079_s_p_d_f_g_h_i_7_6_4_2_1_1_0_error_1.9e-03
   21
     1   0   0   1   1
      0.3689607407    1.0000000000
     2   0   0   1   1
      0.5788974748    1.0000000000
     3   0   0   1   1
      1.7221258107    1.0000000000
     4   0   0   1   1
      6.4710827478    1.0000000000
```

```
     5   0   0   1   1
     27.5076595314    1.0000000000
     6   0   0   1   1
    111.6161257526    1.0000000000
     7   0   0   1   1
    419.4795430550    1.0000000000
     8   1   1   1   1
      0.5008483792    1.0000000000
     9   1   1   1   1
      0.6902875221    1.0000000000
    10   1   1   1   1
      2.2590199483    1.0000000000
    11   1   1   1   1
      8.3540004072    1.0000000000
    12   1   1   1   1
     34.8619879018    1.0000000000
    13   1   1   1   1
    131.4408337050    1.0000000000
    14   2   2   1   1
      0.6727640674    1.0000000000
    15   2   2   1   1
      0.8781593903    1.0000000000
    16   2   2   1   1
      3.3168702090    1.0000000000
    17   2   2   1   1
     19.1614869851    1.0000000000
    18   3   3   1   1
      0.6631555825    1.0000000000
    19   3   3   1   1
      1.2406514373    1.0000000000
    20   4   4   1   1
      0.9635802849    1.0000000000
    21   5   5   1   1
      1.9911109456    1.0000000000

# RI basis set for Cl (all-electron) relative DI metric: 8.6e-04
Cl  RI_aug-TZVP-MOLOPT-ae_N_RI_102_s_p_d_f_g_h_i_7_6_4_4_2_1_0_error_8.6e-04
   24
     1   0   0   1   1
      0.5003852186    1.0000000000
     2   0   0   1   1
      1.1334713346    1.0000000000
     3   0   0   1   1
      2.9543730613    1.0000000000
     4   0   0   1   1
     10.7024447031    1.0000000000
     5   0   0   1   1
     38.4353495638    1.0000000000
     6   0   0   1   1
    127.8254281111    1.0000000000
     7   0   0   1   1
    412.3890943772    1.0000000000
     8   1   1   1   1
      0.3628634448    1.0000000000
     9   1   1   1   1
      0.9117085616    1.0000000000
    10   1   1   1   1
      2.7868113044    1.0000000000
    11   1   1   1   1
     10.6364243877    1.0000000000
    12   1   1   1   1
     38.3777996702    1.0000000000
    13   1   1   1   1
    121.5285421955    1.0000000000
    14   2   2   1   1
      0.5117732297    1.0000000000
    15   2   2   1   1
      1.2502234383    1.0000000000
    16   2   2   1   1
      3.9020255790    1.0000000000
    17   2   2   1   1
     10.8638729890    1.0000000000
    18   3   3   1   1
      0.1879601200    1.0000000000
    19   3   3   1   1
      0.6601763089    1.0000000000
    20   3   3   1   1
      1.7480215520    1.0000000000
    21   3   3   1   1
      4.3416673187    1.0000000000
    22   4   4   1   1
      0.2740064666    1.0000000000
    23   4   4   1   1
      1.0061404098    1.0000000000
    24   5   5   1   1
      0.8845719378    1.0000000000

# RI basis set for Cl (all-electron) relative DI metric: 4.3e-04
Cl  RI_aug-TZVP-MOLOPT-ae_N_RI_116_s_p_d_f_g_h_i_7_6_5_4_3_1_0_error_4.3e-04
   26
     1   0   0   1   1
      0.3723952041    1.0000000000
     2   0   0   1   1
      0.8054492965    1.0000000000
     3   0   0   1   1
      2.3926313700    1.0000000000
     4   0   0   1   1
      7.6483762919    1.0000000000
     5   0   0   1   1
     30.1825004523    1.0000000000
     6   0   0   1   1
    114.7300482667    1.0000000000
     7   0   0   1   1
    422.7684618477    1.0000000000
     8   1   1   1   1
      0.5408463596    1.0000000000
     9   1   1   1   1
      0.7457136183    1.0000000000
    10   1   1   1   1
      2.0564702242    1.0000000000
    11   1   1   1   1
```

```
  8.1621624868    1.0000000000
12   1   1   1   1
 30.5965416866    1.0000000000
13   1   1   1   1
 99.9607329871    1.0000000000
14   2   2   1   1
  0.4056605469    1.0000000000
15   2   2   1   1
  1.0213901469    1.0000000000
16   2   2   1   1
  2.7438413567    1.0000000000
17   2   2   1   1
  7.9225262865    1.0000000000
18   2   2   1   1
 30.5175181105    1.0000000000
19   3   3   1   1
  0.3796553691    1.0000000000
20   3   3   1   1
  0.8398904713    1.0000000000
21   3   3   1   1
  1.9071036546    1.0000000000
22   3   3   1   1
  4.3349358015    1.0000000000
23   4   4   1   1
  0.3379373680    1.0000000000
24   4   4   1   1
  0.8635504287    1.0000000000
25   4   4   1   1
  1.8186751668    1.0000000000
26   5   5   1   1
  1.1855348210    1.0000000000
```

[1] L. Goerigk, A. Hansen, C. Bauer, S. Ehrlich, A. Najibi, and S. Grimme, A look at the density functional theory zoo with the advanced GMTKN55 database for general main group thermochemistry, kinetics and noncovalent interactions, Phys. Chem. Chem. Phys. **19**, 32184 (2017).

[2] A. Karton, S. Daon, and J. M. Martin, W4-11: A high-confidence benchmark dataset for computational thermochemistry derived from first-principles W4 data, Chem. Phys. Lett. **510**, 165 (2011).

[3] S. Grimme, J. Antony, S. Ehrlich, and H. Krieg, A consistent and accurate ab initio parametrization of density functional dispersion correction (DFT-D) for the 94 elements H-Pu, J. Chem. Phys. **132**, 154104 (2010).

[4] H. Yu and D. G. Truhlar, Components of the Bond Energy in Polar Diatomic Molecules, Radicals, and Ions Formed by Group-1 and Group-2 Metal Atoms, J. Chem. Theory Comput. **11**, 2968 (2015).

[5] R. A. Kendall, T. H. Dunning, and R. J. Harrison, Electron affinities of the first-row atoms revisited. Systematic basis sets and wave functions, J. Chem. Phys. **96**, 6796 (1992).

[6] T. H. Dunning, Gaussian basis sets for use in correlated molecular calculations. I. The atoms boron through neon and hydrogen, J. Chem. Phys. **90**, 1007 (1989).

[7] J. Hutter, Optimized MOLOPT basis sets for PBE DFT calculations, SVP-MOLOPT-PBE-ae and TZVPP-MOLOPT-PBE-ae – Basis sets based on the def2-SVP / def2-TZVPP / def2-QZVPP Ahlrichs sets, `https://github.com/cp2k/cp2k/blob/master/data/BASIS_MOLOPT_UZH` (accessed 02 December 2025).

[8] T. Müller, *From Benchmarking to Periodic Fock Exchange in the Auxiliary Density Matrix Method via k-Point Sampling with Gaussian Basis Sets*, Ph.D. thesis, University of Zurich, Zürich (2024).
